# Pd-catalyzed asymmetric Suzuki–Miyaura coupling reactions for the synthesis of chiral biaryl compounds with a large steric substituent at the 2-position

**DOI:** 10.3762/bjoc.16.85

**Published:** 2020-05-11

**Authors:** Yongsu Li, Bendu Pan, Xuefeng He, Wang Xia, Yaqi Zhang, Hao Liang, Chitreddy V Subba Reddy, Rihui Cao, Liqin Qiu

**Affiliations:** 1 School of Chemistry, Guangdong Key Lab of Chiral Molecules and Drug Discovery, Sun Yat-sen University, No. 135 Xingangxi Road, Guangzhou 510275, People’s Republic of China

**Keywords:** asymmetric catalysis, biaryls, monophosphine ligand, palladium catalyst, Suzuki–Miyaura couplings

## Abstract

Pd-catalyzed asymmetric Suzuki–Miyaura couplings of 3-methyl-2-bromophenylamides, 3-methyl-2-bromo-1-nitrobenzene and 1-naphthaleneboronic acids have been successfully developed and the corresponding axially chiral biaryl compounds were obtained in very high yields (up to 99%) with good enantioselectivities (up to 88% ee) under mild conditions. The chiral-bridged biphenyl monophosphine ligands developed by our group exhibit significant superiority to the naphthyl counterpart MOP in both reactivity and enantioselectivity control. The large steric hindrance from π-conjugated *ortho*-substituents of the bromobenzene substrates and the Pd···O interaction between carbonyl and palladium seem essential to achieve high enantioselectivity.

## Introduction

Axially chiral molecules have received much attention from chemists because of their widespread appearance in biologically active compounds [1–4] such as vancomycin [5] and korupensamine A [6] and as useful chiral ligands in asymmetric catalysis. Different strategies with various metals and phosphine ligands had been successfully employed for the efficient synthesis of this scaffold [7–22], like Hiyama [23–24], Negishi [25–26] or Suzuki–Miyaura couplings [27–36]. In these synthetic strategies, the reaction system of palladium with chiral phosphine ligands was studied fruitfully by Cammidge [37–38], Buchwald [13,28,39], Tang [40–43] and other groups [43–59]. Nevertheless, for the asymmetric formation of large steric systems such as sterically demanding biaryls still remain limitations [24], especially on how to obtain the large steric axially chiral biaryl with a high yield and good enantioselectivity through those coupling strategies [13]. Therefore, based on our previous research [60–65], we herein present a new method through which those large steric axially chiral biaryl compounds can be obtained in excellent yields and good enantioselectivities under mild conditions, by using brominated amides and arylboronic acids as substrates, as well as palladium and chiral-bridged biphenyl monophosphine ligands as catalysts.

## Results and Discussion

**Figure 1:**
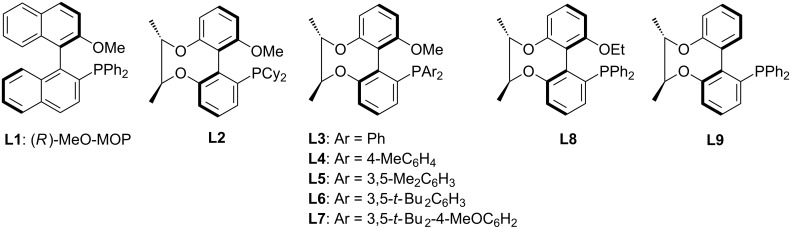
(*R*)-MeO-MOP and our ligands.

2-Bromo-3-methyl-*N*-phenylbenzamide (**1g**) and 1-naphthylboronic acid (**2a**) were utilized to synthesize axially chiral compound **3g**. This reaction was selected as model reaction for further optimization of Pd sources, ligands (Figure 1), solvents, bases and temperature. At first, various phosphine ligands were screened with 2.5 mol % Pd_2_(dba)_3_, 3.0 equivalents K_3_PO_4_ in THF at 50 °C for 72 h. The results show that the ligands have a large effect on the reaction. As listed in Table 1, the reaction was performed well and the product was obtained in 70% yield when using **L1** (*R*)-MOP as the ligand, but the enantioselectivity was unsatisfactorily low (36% ee; Table 1, entry 1). After further examination of the ligands developed by our group (**L2**–**L9**), it was found that phosphine ligands with a large steric aryl group linked to a phosphorus atom are more effective than the ligand with a cyclohexyl group both in yield and enantioselectivity (Table 1, entries 2–7). The results show that the higher the steric hindrance of the ligand, the better the yield and enantioselectivity. In order to find out whether the 1’-substituent of the ligand has any influence on the reaction result, we replaced the methoxy group (**L3**) with an ethoxy group (**L8**), or a hydrogen atom (**L9**), which all led to a significant decrease of yield and enantioselectivity (Table 1, entries 3 vs 8 and 9). Considering the yield and enantioselectivity of the product comprehensively, **L7** was chosen as the most suitable ligand for this reaction and under these conditions product **3g** was obtained in 85% yield and with 78% ee (Table 1, entry 7). In the following investigations, it was found that Pd_2_(dba)_3_ as palladium source, THF as solvent and K_3_PO_4_ as base is the most effective combination (Table 1, entries 10–18). More results suggested that the yield varied directly proportional to the temperature, while the enantioselectivity went inversely (Table 1, entries 19–21). Therefore, 50 °C was chosen as the reaction temperature.

**Table 1:** Optimization of reaction conditions^a^.

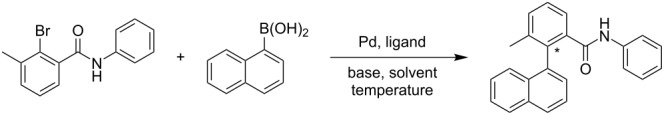							

Entry	Ligand	Pd	Solvent	Base	Temp (°C)	Yield (%)^b^	ee (%)^c^

1	**L1**	Pd_2_(dba)_3_	THF	K_3_PO_4_	50	70	36
2	**L2**	Pd_2_(dba)_3_	THF	K_3_PO_4_	50	36	16
3	**L3**	Pd_2_(dba)_3_	THF	K_3_PO_4_	50	48	54
4	**L4**	Pd_2_(dba)_3_	THF	K_3_PO_4_	50	82	54
5	**L5**	Pd_2_(dba)_3_	THF	K_3_PO_4_	50	83	63
6	**L6**	Pd_2_(dba)_3_	THF	K_3_PO_4_	50	63	76
**7**	**L7**	**Pd_2_(dba)_3_**	**THF**	**K_3_PO_4_**	**50**	**85**	**78**
8	**L8**	Pd_2_(dba)_3_	THF	K_3_PO_4_	50	85	36
9	**L9**	Pd_2_(dba)_3_	THF	K_3_PO_4_	50	64	37
10	**L7**	Pd(OAc)_2_	THF	K_3_PO_4_	50	20	60
11	**L7**	PdCl_2_	THF	K_3_PO_4_	50	78	75
12	**L7**	Pd(CF_3_COO)_2_	THF	K_3_PO_4_	50	20	74
13	**L7**	Pd_2_(dba)_3_	toluene	K_3_PO_4_	50	75	68
14	**L7**	Pd_2_(dba)_3_	DME	K_3_PO_4_	50	45	70
15	**L7**	Pd_2_(dba)_3_	DCE	K_3_PO_4_	50	57	62
16	**L7**	Pd_2_(dba)_3_	THF	Cs_2_CO_3_	50	60	52
17	**L7**	Pd_2_(dba)_3_	THF	KF	50	30	78
18	**L7**	Pd_2_(dba)_3_	THF	CsF	50	53	74
19	**L7**	Pd_2_(dba)_3_	THF	K_3_PO_4_	40	63	78
20	**L7**	Pd_2_(dba)_3_	THF	K_3_PO_4_	60	90	72
21	**L7**	Pd_2_(dba)_3_	THF	K_3_PO_4_	70	90	69

^a^Reaction conditions: 1 equiv of *N*-aryl-bromoarylamide, 2 equiv of naphthylboronic acid, 5 mol % Pd, 6 mol % of ligand, 3 equiv of base, 2 mL solvent, 50 ⁰C, 72 h. ^b^NMR Yield. ^c^Characterized by HPLC with a chiral AD-H column.

**Scheme 1:**
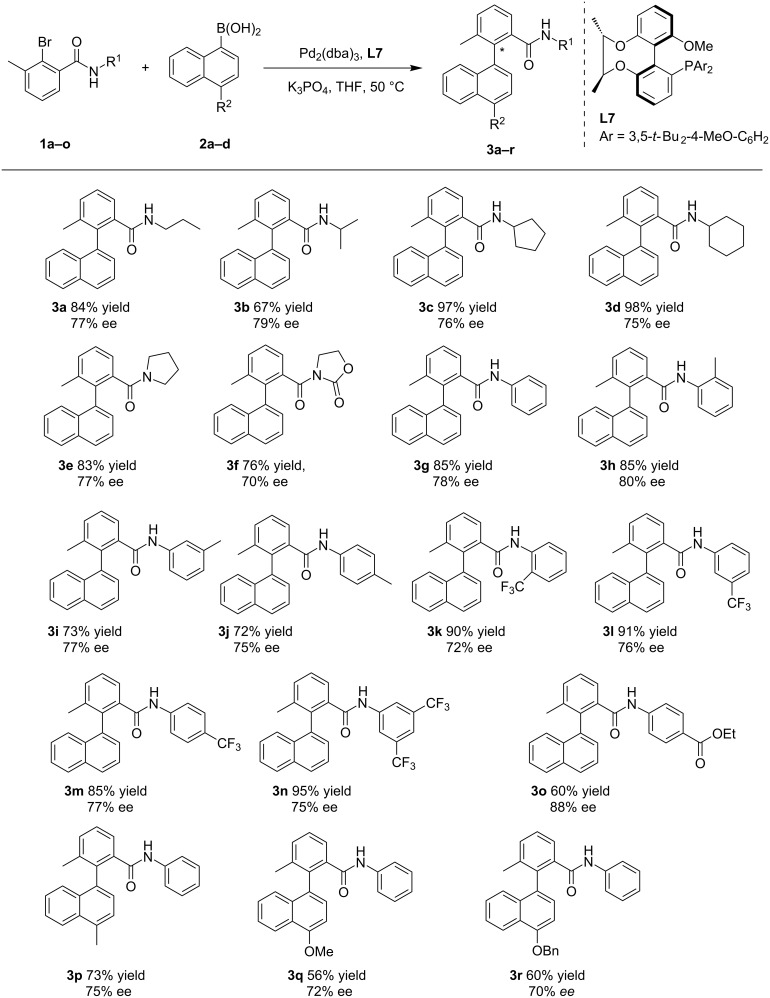
Asymmetric Suzuki–Miyaura coupling. Reaction conditions: 1 equiv *N*-aryl-bromoaryl compounds, 2 equiv arylboronic acids, 5 mol % Pd, 6 mol % ligand, 3 equiv of K_3_PO_4_, 2 mL THF, 50 ⁰C, 72 h; Yields are combined isolated values; ee values were determined by HPLC with chiral columns.

With the optimized reaction conditions in hand, we expanded the reaction with various functionalized starting materials, as shown in Scheme 1. Linear *N*-alkyl substituted amides were found to produce better yields than *N*-branched alkyl chain amides, though they performed similar ee values (**3a**, **3b**). *N*-Cycloalkyl-substituted amides enabled the reaction to achieve a quantitative conversion with good ee value (97% yield, 76% ee for **3c**; 98% yield, 75% ee for **3d**). The yield and ee value of an oxazolidinone amide were slightly lower than those of tetrahydropyrrolamide (**3e**, **3f**). Various aromatic substituted amides were investigated. The results show that electron-rich or electron-deficient substituents on the phenyl ring have no significant influence on the enantiomeric excesses of the products (**3g–n**), but the best ee value was obtained for the substrate with an electron-deficient phenyl ester (88% ee, **3o**). By changing 1-naphthaleneboronic acid to 4-substituted-1-naphthaleneboronic acids **3p**, **3q** and **3r**, the enantioselectivity and the yield of the reaction decreased with the increase of the substituent on the boronic acid. When a methyl or methoxy group were present at the *ortho*-position of 1-naphthaleneboronic acid, the reactions were hard to move on even at 70 °C. By replacing the 3-methyl group of the amide with a 3-methoxy or a 3-benzyloxy group, the yield and ee value of the reaction were still unsatisfactory though the temperature had been raised to 70 °C too (see Supporting Information File 1, Scheme S1, compounds **3s**, 65% yield, 18% ee; **3t**, 60% yield, 23% ee). In addition, phenylboric acids were also investigated, but the reaction results were not so good (see Supporting Information File 1, Scheme S1, compounds **3u**, 60% yield, 11% ee; **3v**, 90% yield, 0% ee).

More specifically, the change of the R^1^ group on the bromoamide compound has a weak influence on the coupling reaction. No matter it was a straight alkyl chain, a branched alkyl chain, a heterocyclic group, an electron-rich aromatic or an electron-deficient aromatic group, the reaction always performed well with high yield and good enantioselectivity. We guess that the large sterically hindered π-plane formed between a carbonyl group and a benzene ring is the guarantee of high ee values of the product [13,39,42,57,65] along with the Pd···O interaction [13,64] between carbonyl and palladium. The experimental results also show that the substituent of the arylboronic acid has an obvious effect on the reaction: the yield and ee value of the product decrease with the increase of the steric hindrance of the 4-substituted boronic acid.

**Scheme 2:**
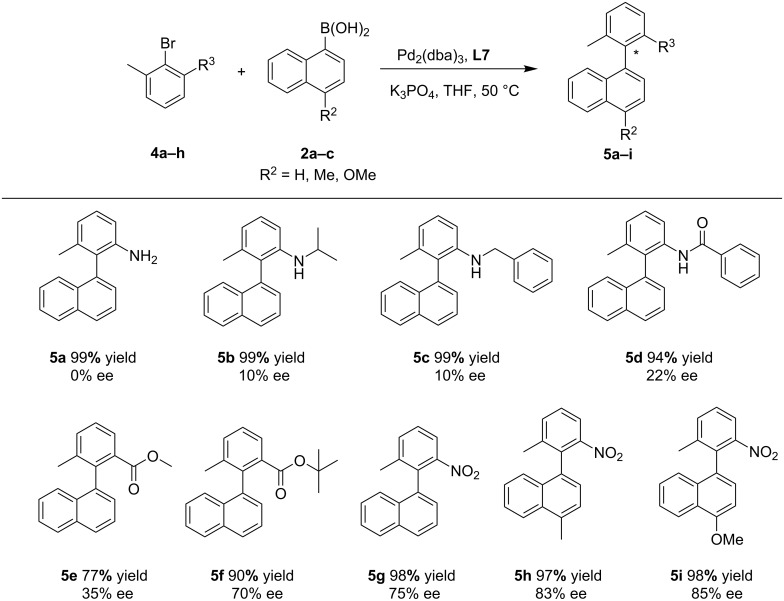
Asymmetric Suzuki–Miyaura coupling. Reaction conditions: 1 equiv of bromoaryl compounds, 2 equiv of arylboronic acids, 5 mol % Pd, 6 mol % of ligand, 3 equiv of K_3_PO_4_, 2 mL THF, 50 ⁰C, 72 h; Yields are combined isolated values; ee values were determined by HPLC.

**Scheme 3:**
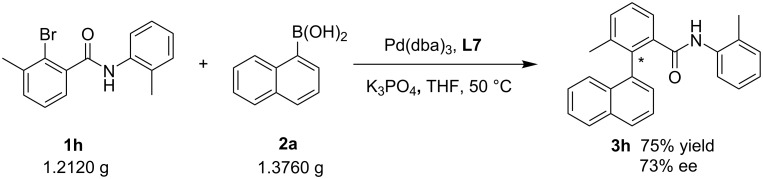
Gram-scale reaction.

Next, in order to confirm our speculation, the amide group of the aryl bromide was replaced with other functional groups, as shown in Scheme 2. When the amide group was changed into an amine, coupling product **5a** was obtained quantitatively but without any enantioselectivity. Just a slight improvement of the ee value was found along with the introduction of bulkier substituents to the amino group (**5b–d**). From the reaction results, it can be seen that the Pd···O interaction [13,64] between the carbonyl group and the palladium plays an important role for the reaction enantioselectivity. However, with the substitution of an ester group for the amino moiety, interestingly, the situation changed significantly. The greater the ester substituent, the better the reaction results including the product yield and ee value. When the functional group changed from methyl ester to *tert*-butyl ester, the reaction yield rose up from 77% to 90% and the enantioselectivity increased from 35% to 70% (**5e**, **5f**). This indicates that the O···Pd interaction between the carbonyl group and the palladium is not strong enough to determine the enantioselectivity of the reaction solely, the steric hindrance from π-conjugated *ortho*-substituents of the bromobenzenes is also important. In addition, coupling of 2-bromo-3-nitrotoluene with 4-substituted or unsubstituted 1-naphthaleneboronic acids provided the corresponding products in high yields with good ee values (**5g**, 98% yield, 75% ee; **5h**, 97% yield, 83% ee; **5i**, 98% yield, 85% ee) [39,62]. All these results show that the large steric hindrance from π-conjugated *ortho*-substituents of the bromobenzenes and the O···Pd interaction work together for the acquisition of high enantioselectivity. As stated before, the replacement of naphthaleneboronic acid with phenylboronic acid derivatives also resulted in poor enantioselectivities of the products (see Supporting Information File 1, Scheme S1, **3u**, 60% yield, 11% ee; **3v**, 99% yield, 0% ee). This further demonstrates the importance of the steric hindrance and rigidity from the other substrate arylboronic acid. A gram-scale reaction of **1h** with **2a** went well and provided **3h** with a slightly reduced yield and ee (75% yield and 73% ee, Scheme 3), compared with **3h** in Scheme 1.

**Scheme 4:**
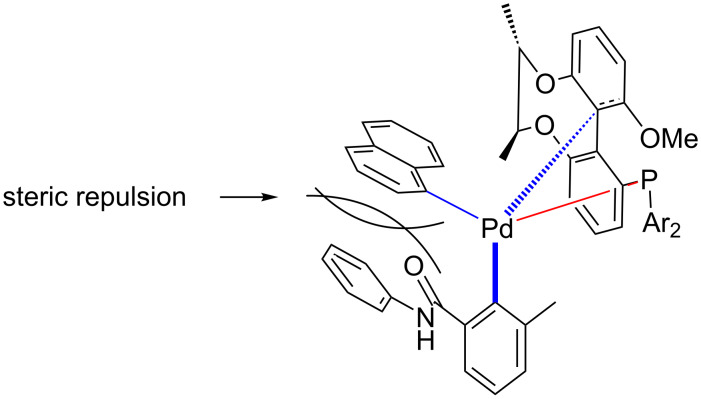
Based on our analysis and speculation, a possible intermediate structure is proposed [65–66].

According to the coordination of axial chiral phosphine ligands [65–66] and our analysis, we propose a possible intermediate structure (Scheme 4).

## Conclusion

In summary, a Pd-catalyzed asymmetric Suzuki–Miyaura coupling of 3-methyl-2-bromophenylamides or 3-methyl-2-bromo-1-nitrobenzene and 1-naphthaleneboronic acids has been successfully developed and the corresponding axially chiral biaryl compounds were obtained in very high yields (up to 99%) and with good enantioselectivities (up to 88% ee) under mild conditions. The chiral-bridged biphenyl monophosphine ligands developed by our group, especially **L7**, exhibited significant superiority to the naphthyl counterpart MOP in reactivity and enantioselectivity in the reactions. The large steric hindrance from π-conjugated *ortho*-substituents of the bromobenzene substrates and the existence of the O···Pd interaction between the carbonyl group and the palladium are beneficial to acquire high enantioselectivities.

## Experimental

### General procedure for the synthesis of amide substrates

**Scheme 5:**
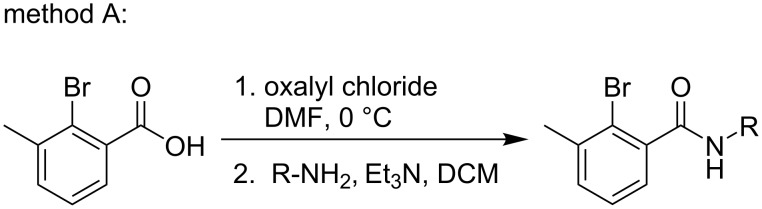
Method A for the synthesis of amide substrates.

**Method A:** To a stirred solution of 2-bromo-3-methylbenzoic acid (2.0 g, 1.0 equiv) in DMF (0.05 equiv), oxalyl chloride (1.5 equiv) was added in DCM (20 mL) at 0 °C. After the addition was completed, the reaction mixture was further stirred for 2 h at room temperature. After the disappearance of the benzoic acid (monitored by TLC), the corresponding arylamines (1.5 equiv) were added, followed by the addition of triethylamine (2.0 equiv) in DCM (10 mL). After that the resulting reaction mixture was stirred for 20 h at room temperature and checked by TLC for reaction completion. The reaction mixture was then quenched with ice-cold water and extracted with DCM. The organic layer was washed with brine, dried over sodium sulfate and concentrated under reduced pressure to obtain the crude product. The crude product was purified by column chromatography to obtain the desired amide compounds.

**Scheme 6:**
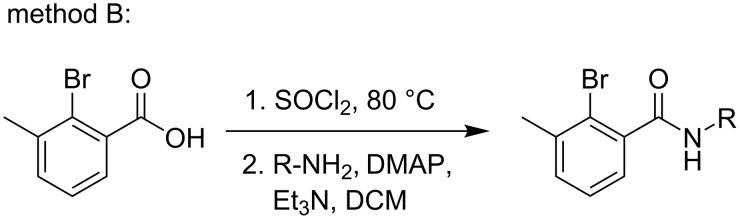
Method B for the synthesis of amide substrates.

**Method B:** A round bottom flask (100 mL) was charged with 2-bromo-3-methylbenzoic acid (2.0 g, 1.0 equiv) and SOCl_2_ (16 mL) was added at room temperature. Then, the reaction mixture was refluxed at 80 °C for 2 h. After completion of the reaction, the excess thionyl chloride was removed by evaporation using a rotary evaporator. Then, the corresponding arylamines (1.2 equiv) were added to the acyl chlorides, triethylamine (1.5 equiv) and DMAP (5 mol %) in DCM (20 mL). The resulting reaction mixture was stirred for 20 h at room temperature. The completion of the reaction was monitored by TLC. After completion of the reaction ice-cold water was added and the separated organic layer was washed with brine and saturated sodium bicarbonate solution. Then, the organic layer was dried over anhydrous sodium sulfate and concentrated under reduced pressure to obtain the crude product. The crude product was purified by column chromatography to obtain the desired products.

### General procedure for the asymmetric Suzuki–Miyaura coupling

In a glovebox, an oven-dried sealing tube (15 mL) was charged with bromoarylamides (0.2 mmol, 1.0 equiv), Pd_2_(dba)_3_ (0.005 mmol, 5 mol % Pd), ligand **L7** (0.012 mmol, 6 mol %), arylboronic acid (0.4 mmol, 2.0 equiv), K_3_PO_4_ (0.6 mmol, 3.0 equiv) and 2 mL of dry THF. S-Phos was used as ligand for the preparation of racemic products and all the reactions were carried out at 50 °C for 72 h. The completion of the reaction was checked by TLC. After completion of the reaction, the reaction mixture was cooled to room temperature and water was added. Then, the reaction mixture was extracted with ethyl acetate and the organic layer was dried over anhydrous Na_2_SO_4_ and concentrated under reduced pressure to obtain the crude product. The crude product was purified by flash chromatography using silica gel. The enantiomeric excess value of the product was determined by HPLC by using an AD-H, OD-H or IA-3 column.

## Supporting Information

File 1 Further experimental data, copies of NMR spectra and HPLC chromatograms.
