# Supplementary material for: Pd-catalyzed asymmetric Suzuki–Miyaura coupling reactions for the synthesis of chiral biaryl compounds with a large steric substituent at the 2-position
Source: Beilstein J Org Chem. 2020 May 11;16:966–73. doi: 10.3762/bjoc.16.85 (PMC7237804; doi:10.3762/bjoc.16.85)
Supplement: File 1 — Further experimental data, copies of NMR spectra and HPLC chromatograms. [file Beilstein_J_Org_Chem-16-966-s001.pdf]

BEILSTEIN JOURNAL OF ORGANIC CHEMISTRY

Supporting Information

for

Pd-catalyzed asymmetric Suzuki–Miyaura coupling reactions for the synthesis of chiral biaryl compounds with a large steric substituent at the 2-position

Yongsu Li, Bendu Pan, Xuefeng He, Wang Xia, Yaqi Zhang, Hao Liang, Chitreddy V. Subba Reddy, Rihui Cao and Liqin Qiu

Beilstein J. Org. Chem. **2020,** *16,* 966–973. doi:10.3762/bjoc.16.85

Further experimental data, copies of NMR spectra and HPLC chromatograms

License and Terms: This is a supporting information file under the terms of the Creative Commons Attribution License (http://creativecommons.org/licenses/by/4.0). Please note that the reuse, redistribution and reproduction in particular requires that the authors and source are credited.
The license is subject to the *Beilstein Journal of Organic Chemistry* terms and conditions: (https://www.beilstein-journals.org/bjoc)

Table of Contents

General consideration S2

Experimental section..S2

Copies of NMR spectra...S14

Copies of HPLC chromatograms...S49

General consideration

All reactions and controls were accomplished in a nitrogen-filled glove box or under nitrogen using standard techniques unless otherwise noted. All the commercially available chemicals were purchased from suppliers such as Titan, Aladdin etc. and were used without further purification and the reactions were monitored by TLC. All the reactions were performed using oven dried glassware. A WRS-3 melting point apparatus was used for recording the melting points in open capillaries and are uncorrected. Column chromatography was performed using silica gel (200–300 mesh). The ^{1}H NMR and ^{13}C NMR values were obtained using a Bruker Avance-400 MHz spectrometer in $CDCl_3$ or DMSO-d_6 solvent with TMS or other deuterated solvent as the internal standard. The products' enantiomeric excesses (ee values) were determined by chiral HPLC analysis using an Aglient HP 1200 instrument (*n*-hexane/2-propanol as eluent) with a Chialcel OD-H, AD-H or IA-3. Chemical shift values (δ) were expressed in parts per million (ppm). Coupling constants (*J*) are reported in Hertz (Hz). Abbreviations are as follows: s = singlet, d = doublet, t = triplet, q = quartet, m = multiplet and dd = doublet of doublets.

Experimental section

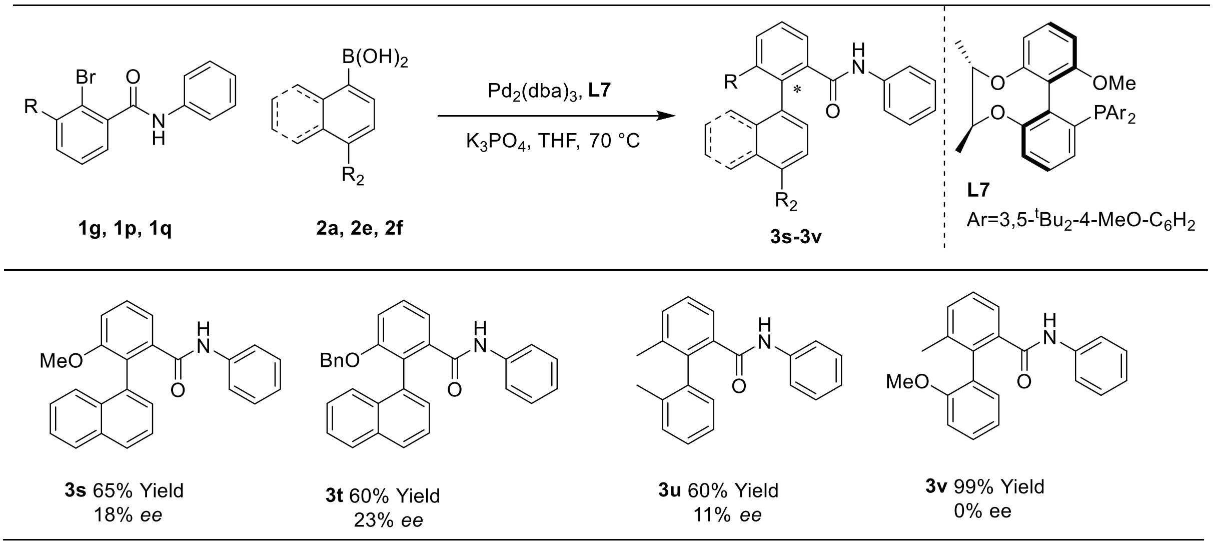

Scheme S1: Asymmetric Suzuki–Miyaura coupling. Reaction conditions: 1 equiv of aryl bromoaryl compounds, 2 equiv of arylboronic acids, 5 mol % Pd, 6 mol % of ligand, 3 equiv of K_3PO_4, 2 mL solvent, 70 °C, 72 h; Yields are combined isolated values; ee values were determined by HPLC.

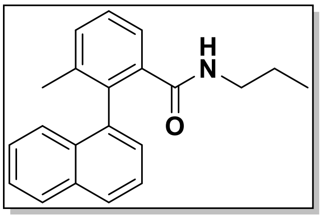

3-Methyl-2-(naphthalen-1-yl)-*N*-propylbenzamide (3a)

White solid, 84% yield, 77% ee, $[\alpha]_D^{20}$ = -4.4, M.P.: 70.8 – 71.8 °C. The enantiomeric excess was determined by HPLC analysis using a chiral stationary phase column [Daicel Chiralcel® AD-H, 254 nm, *n*-hexane/iPrOH = 95:5 as the eluent, flow rate: 1 mL/min, retention times: 9.9 min (minor isomer) and 11.6 min (major isomer)]. ^{1}H NMR (400 MHz, $CDCl_3$, ppm) δ 7.92 (d, *J* = 8.2 Hz, 1H), 7.89 (d, *J* = 8.4 Hz, 1H), 7.70 (dd, *J* = 6.2, 2.9 Hz, 1H), 7.53 (m, 1H), 7.50 (dd, *J* = 8.5, 4.3 Hz, 1H), 7.45 – 7.38 (m, 4H), 7.34 (d, *J* = 6.9 Hz, 1H), 5.10 (s, 1H), 2.83 (td, *J* = 13.4, 6.6 Hz, 1H), 2.70 (td, *J* = 12.9, 7.0 Hz, 1H), 1.95 (s, 3H), 0.71 – 0.59 (m, 1H), 0.59 – 0.46 (m, 1H), 0.28 (t, *J* = 7.4 Hz, 3H); ^{13}C NMR (101 MHz, $CDCl_3$, ppm) δ 168.83, 137.64, 137.37, 137.20, 136.58, 133.69, 131.90, 131.66, 128.66, 128.12, 127.99, 127.01, 126.90, 126.49, 126.23, 125.78, 124.91, 41.13, 21.88, 20.34, 10.81; MS(ESI): m/z 304 $[M+H]^+$

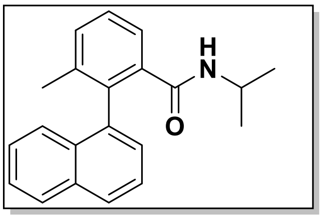

***N*-Isopropyl-3-methyl-2-(naphthalen-1-yl)benzamide (3b)**

White solid, 67% yield, 79% ee, $[\alpha]_D^{20}$ = -60.9, M.P.: 89.3 –91.1 °C. The enantiomeric excess was determined by HPLC analysis using a chiral stationary phase column [Daicel Chiralcel® OD-H, 254 nm, *n*-hexane/iPrOH = 97:3 as the eluent, flow rate: 1 mL/min, retention times: 10.1 min (minor isomer) and 11.1 min (major isomer)]. ^{1}H NMR (400 MHz, $CDCl_3$, ppm) δ 7.92 (m, 2H), 7.72 – 7.66 (m, 1H), 7.55 (dd, *J* = 6.0, 4.8 Hz, 1H), 7.53 – 7.49 (m, 1H), 7.44 (m, 3H), 7.42 (m, 1H), 7.34 (dd, *J* = 7.0, 1.0 Hz, 1H), 5.00 – 4.75 (s, 1H), 3.71 – 3.59 (m, 1H), 1.99 (s, 3H), 0.40 (d, *J* = 6.5 Hz, 3H), 0.27 (d, *J* = 6.5 Hz, 3H); ^{13}C NMR (101 MHz, $CDCl_3$, ppm) δ 167.88, 137.56, 137.52, 137.27, 136.53, 133.70, 132.08, 131.61, 128.63, 128.05, 128.01, 127.03, 126.96, 126.42, 126.24, 125.76, 124.87, 40.89, 21.69, 21.52, 20.33; MS(ESI): m/z 304 $[M+H]^+$

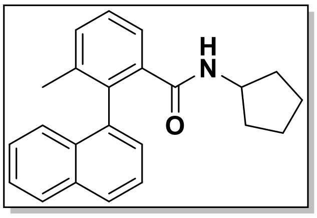

***N*-Cyclopentyl-3-methyl-2-(naphthalen-1-yl)benzamide (3c)**

White solid, 97% yield, 76% ee, $[\alpha]_D^{20}$ = -66.5, M.P.: 95.9 – 96.8 °C The enantiomeric excess was determined by HPLC analysis using a chiral stationary phase column [Daicel Chiralcel® AD-H, 254 nm, *n*-hexane/iPrOH = 95:5 as the eluent, flow rate: 1 mL/min, retention times: 8.8 min (minor isomer) and 9.8 min (major isomer)]. ^{1}H NMR (400 MHz, $CDCl_3$, ppm) δ 7.92 (m, 2H), 7.73 – 7.70 (m, 1H), 7.54 (m, 1H), 7.53 – 7.48 (m, 1H), 7.45 – 7.38 (m, 4H), 7.33 (m, 1H), 5.05 (s, 1H). 3.92 – 3.76 (m, 1H), 1.96 (s, 3H), 1.48 – 1.25 (m, 2H), 1.21 – 1.07 (m, 2H), 0.95 – 0.69 (m, 2H), 0.56 – 0.39 (m, 1H), 0.29 – 0.15 (m, 1H); ^{13}C NMR (101 MHz, $CDCl_3$, ppm) δ 168.07, 137.55, 137.40, 137.30, 136.48, 133.70, 131.95, 131.63, 128.12, 128.03, 127.10, 126.95, 126.58, 126.30, 125.85, 124.82, 50.75, 32.32, 32.27, 23.18, 23.07, 20.34; MS(ESI): m/z 330 $[M+H]^+$

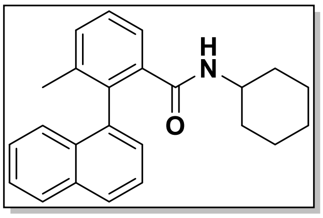

_N_-Cyclohexyl-3-methyl-2-(naphthalen-1-yl)benzamide (3d)

White solid, 98% yield, 75% ee, $[\alpha]_D^{20}$ = -67.6, M.P.: 128.8 – 129.8 °C. The enantiomeric excess was determined by HPLC analysis using a chiral stationary phase column [Daicel Chiralcel® AD-H, 254 nm, *n*-hexane/iPrOH = 95:5 as the eluent, flow rate: 1 mL/min, retention times: 7.4 min (minor isomer) and 9.0 min (major isomer)]. ^{1}H NMR (400 MHz, $CDCl_3$, ppm) δ 7.94 (m, 2H), 7.72 (dd, *J* = 6.6, 2.4 Hz, 1H), 7.58 – 7.55 (m, 1H), 7.53 (m, 1H), 7.48 – 7.41 (m, 4H), 7.36 (m, 1H), 5.01 (s, 1H), 3.68 – 3.18 (m, 1H), 1.99 (s, 3H), 1.38 – 1.29 (m, 1H), 1.23 (m, 2H), 1.17 – 0.94 (m, 4H), 0.87 – 0.73 (m, 1H), 0.26 (m, 1H), 0.10 – -0.04 (m, 1H); ^{13}C NMR (101 MHz, $CDCl_3$, ppm) δ 167.80, 137.59, 137.57, 137.28, 136.49, 133.74, 132.04, 131.58, 128.64, 128.07, 128.00, 127.03, 126.92, 126.52, 126.24, 125.81, 124.87, 47.54, 32.00, 31.75, 25.24, 24.19, 24.12, 20.35; MS(ESI): m/z 344 $[M+H]^+$

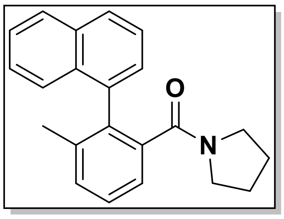

3-Methyl-2-(naphthalen-1-yl)phenyl)(pyrrolidin-1-yl)methanone (3e)

White solid, 83% yield, 77% ee, $[\alpha]_D^{20}$ = -108.3, M.P.: 105.5 –106.7 °C. The enantiomeric excess was determined by HPLC analysis using a chiral stationary phase column [Daicel Chiralcel® AD-H, 254 nm, *n*-hexane/iPrOH = 95:5 as the eluent, flow rate: 1 mL/min, retention times: 12.0 min (minor isomer) and 17.0 min (major isomer)]. ^{1}H NMR (400 MHz, $CDCl_3$, ppm) δ 7.86 (m, 1H), 7.82 (d, *J* = 8.2 Hz, 1H), 7.50 (d, *J* = 7.1 Hz, 1H), 7.47 (d, *J* = 5.8 Hz, 1H), 7.44 (d, *J* = 1.1 Hz, 1H), 7.42 (d, *J* = 6.9 Hz, 1H), 7.40 – 7.37 (m, 1H), 7.37 – 7.32 (m, 2H), 7.25 (dd, *J* = 6.5, 1.8 Hz, 1H), 7.25 (m, 1H), 3.24 – 3.12 (m, 1H), 3.07 (dt, *J* = 10.9, 6.6 Hz, 1H), 3.03 – 2.94 (m, 1H), 2.94 – 2.85 (m, 1H), 1.98 (s, 3H), 1.66 – 1.56 (m, 1H), 1.56 – 1.48 (m, 1H), 1.49 – 1.38 (m, 1H), 1.14 (m, 1H); ^{13}C NMR (101 MHz, $CDCl_3$, ppm) δ 169.10, 139.18, 138.06, 136.17, 133.39, 131.56, 130.17, 128.41, 127.90, 127.85, 127.24, 125.92, 125.69, 125.60, 125.30, 123.52, 48.40, 44.81, 25.43, 24.08, 20.31; MS(ESI): m/z 316 $[M+H]^+$

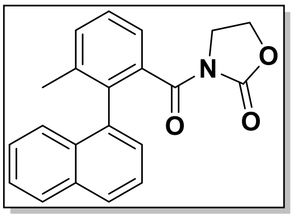

3-(3-Methyl-2-(naphthalen-1-yl)benzoyl)oxazolidin-2-one (3f)

White solid, 76% yield, 70% ee, $[\alpha]_D^{20}$ = -47.9, M.P.: 74.3 – 75.5 °C. The enantiomeric excess was determined by HPLC analysis using a chiral stationary phase column [Daicel Chiralcel® AD-H, 254 nm, *n*-hexane/iPrOH = 97:3 as the eluent, flow rate: 1 mL/min, retention times: 34.2 min (major isomer) and 38.9 min (minor isomer)]. ^{1}H NMR (400 MHz, $CDCl_3$, ppm) δ 7.78 (m, 2H), 7.44 – 7.36 (m, 5H), 7.34 (d, *J* = 8.3 Hz, 1H), 7.29 (m, 2H), 3.73 (m, 1H), 3.66 (m, 1H), 3.37 (m, 1H), 3.19 (m, 1H), 1.95 (s, 3H); ^{13}C NMR (101 MHz, $CDCl_3$ ppm) δ 169.79, 152.47, 137.62, 136.82, 136.26, 135.86, 133.33, 132.12, 131.71, 127.93, 127.91, 127.67, 126.36, 126.32, 126.15, 125.09, 124.43, 61.96, 42.51, 20.19; MS(ESI): m/z 354 $[M+Na]^+$

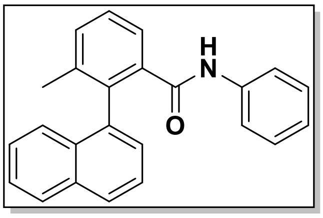

3-Methyl-2-(naphthalen-1-yl)-*N*-phenylbenzamide (3g)

White solid, 85% yield, 78% ee, $[\alpha]_D^{20}$ = -102.4, M.P.: 125.9 – 126.7 °C. The enantiomeric excess was determined by HPLC analysis using a chiral stationary phase column [Daicel Chiralcel® AD-H, 254 nm, *n*-hexane/iPrOH = 97:3 as the eluent, flow rate: 1 mL/min, retention times: 27.4 min (major isomer) and 32.4 min (minor isomer)]. ^{1}H NMR (400 MHz, $CDCl_3$, ppm) δ 7.95 (d, *J* = 8.1 Hz, 1H), 7.90 (d, *J* = 8.3 Hz, 1H), 7.86 (d, *J* = 4.9 Hz, 1H), 7.57 – 7.52 (m, 2H), 7.51 (d, *J* = 2.8 Hz, 1H), 7.48 (m, 3H), 7.39 (d, *J* = 6.9 Hz, 1H), 7.02 (m, 2H), 6.94 (s, 1H), 6.88 (m, 1H), 6.62 (m, 2H), 1.99 (s, 3H); ^{13}C NMR (101 MHz, $CDCl_3$, ppm) δ 166.63, 137.92, 137.39, 136.89, 136.84, 136.69, 133.81, 132.35, 131.70, 128.92, 128.58, 128.53, 128.26, 127.46, 127.29, 127.09, 126.53, 126.09, 124.72, 123.99, 119.44, 77.37, 77.05, 76.73, 20.35; MS(ESI): m/z 338 $[M+H]^+$

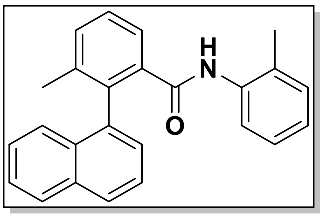

3-Methyl-2-(naphthalen-1-yl)-*N*-(*o*-tolyl)benzamide (3h)

White solid, 85% yield, 80% ee, $[\alpha]_D^{20}$ = -61.1, M.P.: 108.1 – 108.5 °C. The enantiomeric excess was determined by HPLC analysis using a chiral stationary phase column [Daicel Chiralcel® AD-H, 254 nm, *n*-hexane/iPrOH = 95:5 as the eluent, flow rate: 1 mL/min, retention times: 27.0 min (major isomer) and 30.8 min (minor isomer)]. ^{1}H NMR (400 MHz, $CDCl_3$, ppm) δ 7.91 (d, *J* = 7.9 Hz, 1H), 7.87 (d, *J* = 8.2 Hz, 1H), 7.83 (d, *J* = 5.5 Hz, 1H), 7.53 (m, 3H), 7.49 – 7.41 (m, 4H), 7.31 (d, *J* = 7.9 Hz, 1H), 6.98 (m,1H), 6.89 (m, 2H), 6.68 (s, 1H), 6.68 (s, 1H), 1.96 (s, 3H), 1.28 (s, 3H); ^{13}C NMR (101 MHz, $CDCl_3$, ppm) δ 167.31, 138.00, 137.36, 136.76, 136.45, 135.43, 133.85, 132.15, 131.88, 130.07, 129.37, 128.84, 128.47, 128.20, 127.21, 127.18, 127.07, 126.39, 126.31, 126.01, 124.98, 122.84, 20.38, 16.69; MS(ESI): m/z 352 $[M+H]^+$

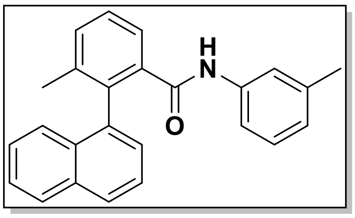

3-Methyl-2-(naphthalen-1-yl)-*N*-(*m*-tolyl)benzamide (3i)

White solid, 73% yield, 77% ee, $[\alpha]_D^{20}$ = -45.3, M.P.: 116.6 – 117.7 °C. The enantiomeric excess was determined by HPLC analysis using a chiral stationary phase column [Daicel Chiralcel® OD-H, 254 nm, *n*-hexane/iPrOH = 95:5 as the eluent, flow rate: 1 mL/min, retention times: 12.1 min (minor isomer) and 14.0 min (major isomer)]. ^{1}H NMR (400 MHz, $CDCl_3$, ppm) δ 7.96 (d, *J* = 8.1 Hz, 1H), 7.89 (d, *J* = 8.3 Hz, 1H), 7.85 (dd, *J* = 5.4, 3.6 Hz, 1H), 7.57 (d, *J* = 2.0 Hz, 1H), 7.56 – 7.54 (m, 1H), 7.53 (d, *J* = 1.0 Hz, 1H), 7.51 (d, *J* = 3.8 Hz, 2H), 7.49 – 7.46 (m, 2H), 7.38 (d, *J* = 6.9 Hz, 1H), 6.95 – 6.82 (m, 2H), 6.70 (d, *J* = 7.6 Hz, 1H), 6.51 (s, 1H), 6.31 (d, *J* = 7.9 Hz, 1H), 2.13 (s, 3H), 2.00 (s, 3H); ^{13}C NMR (101 MHz, $CDCl_3$, ppm) δ 166.62, 138.43, 137.90, 137.27, 137.03, 136.86, 136.68, 133.85, 132.26, 131.73, 128.88, 128.47, 128.32, 128.24, 127.45, 127.22, 127.09, 126.48, 126.11, 124.79, 124.72, 120.19, 116.47, 77.34, 77.03, 76.71, 21.29, 20.32; MS(ESI): m/z 352 $[M+H]^+$

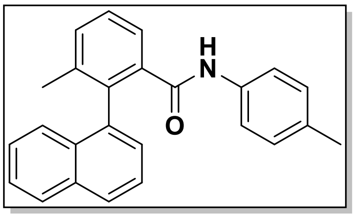

3-Methyl-2-(naphthalen-1-yl)-*N*-(*p*-tolyl)benzamide (3j)

White solid, 72% yield, 75% ee, $[\alpha]_D^{20}$ = -125.5, M.P.: 109.5 – 110.0 °C. The enantiomeric excess was determined by HPLC analysis using a chiral stationary phase column [Daicel Chiralcel® OD-H, 254 nm, *n*-hexane/iPrOH = 95:5 as the eluent, flow rate: 1 mL/min, retention times: 12.1 min (minor isomer) and 13.0 min (major isomer)]. ^{1}H NMR (400 MHz, $CDCl_3$, ppm) δ 7.95 (d, *J* = 8.1 Hz, 1H), 7.90 (d, *J* = 8.3 Hz, 1H), 7.86 (dd, *J* = 6.1, 3.0 Hz, 1H), 7.57 – 7.51 (m, 2H), 7.50 (m, 2H), 7.49 – 7.45 (m, 2H), 7.39 (d, *J* = 6.9 Hz, 1H), 6.83 (m, 2H), 6.50 (m, 2H), 2.16 (s, 3H), 1.99 (s, 3H); ^{13}C NMR (101 MHz, $CDCl_3$, ppm) δ 166.51, 137.86, 136.98, 136.89, 136.63, 134.81, 133.79, 133.61, 132.24, 131.70, 129.07, 128.88, 128.47, 128.23, 127.42, 127.27, 127.07, 126.50, 126.08, 124.73, 119.52, 20.74, 20.35; MS(ESI): m/z 352 $[M+H]^+$

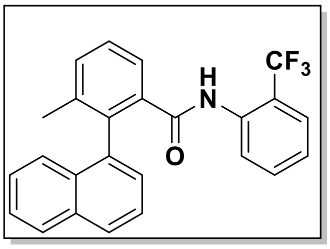

3-Methyl-2-(naphthalen-1-yl)-*N*-(2-(trifluoromethyl)phenyl) benzamide (3k)

White solid, 90% yield, 72% ee, $[\alpha]_D^{20}$ = -49.2, M.P.: 80.4 – 81.5 °C. The enantiomeric excess was determined by HPLC analysis using a chiral stationary phase column [Daicel Chiralcel® OD-H, 254 nm, *n*-hexane/iPrOH = 95:5 as the eluent, flow rate: 1 mL/min, retention times: 6.7 min (minor isomer) and 7.5 min (major isomer)]. ^{1}H NMR (400 MHz, $CDCl_3$, ppm) δ 7.87 (d, *J* = 8.1 Hz, 1H), 7.84 (d, *J* = 8.3 Hz, 1H), 7.72 (dd, *J* = 6.4, 1.8 Hz, 1H), 7.56 – 7.47 (m, 5H), 7.44 (m, 3H), 7.33 (m, 1H), 7.23 (m, 1H), 7.11 (m, 1H), 2.04 (s, 3H); ^{13}C NMR (101 MHz, $CDCl_3$, ppm) δ 167.68, 138.30, 137.44, 137.08, 136.43, 134.72, 133.61, 132.47, 132.43, 131.87, 128.49, 128.28, 128.13, 126.80, 126.75, 126.12, 125.79, 125.79 (q, 20 Hz), 125.13, 124.96, 124.56, 20.33; ^{19}F NMR (377 MHz, $CDCl_3$, ppm) δ -61.06; MS(ESI): m/z 406 $[M+H]^+$

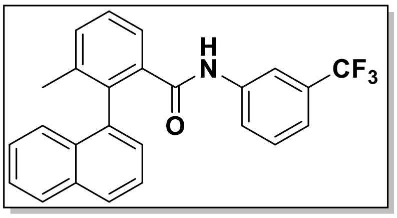

3-Methyl-2-(naphthalen-1-yl)-*N*-(3-(trifluoromethyl)phenyl)benzamide (3l)

White solid, 91% yield, 76% ee, $[\alpha]_D^{20}$ = -52.0, M.P.: 124.5 – 125.6 °C. The enantiomeric excess was determined by HPLC analysis using a chiral stationary phase column [Daicel Chiralcel® OD-H, 254 nm, *n*-hexane/iPrOH = 97:3 as the eluent, flow rate: 1 mL/min, retention times: 9.9 min (minor isomer) and 10.7 min (major isomer)]. ^{1}H NMR (400 MHz, $CDCl_3$, ppm) δ 7.98 (d, *J* = 8.1 Hz, 1H), 7.91 (d, *J* = 8.3 Hz, 1H), 7.90 – 7.86 (m, 1H), 7.58 (dd, *J* = 7.9, 3.8 Hz, 1H), 7.55 (dd, *J* = 7.0, 1.8 Hz, 1H), 7.52 (d, *J* = 2.3 Hz, 1H), 7.52 (m, 2H), 7.50 (d, *J* = 2.1 Hz, 1H), 7.39 (dd, *J* = 7.0, 0.9 Hz, 1H), 7.16 – 7.10 (m, 2H), 7.00 (s, 1H), 6.91 – 6.85 (m, 1H), 6.65 (s, 1H), 2.03 (s, 3H); ^{13}C NMR (101 MHz, $CDCl_3$, ppm) δ 166.76, 138.01, 137.78, 136.75, 136.70, 136.34, 133.86, 132.71, 131.59, δ 130.88 (q, *J* = 32.4 Hz), 129.10, 129.06, 128.66, 128.39, 127.73, 127.41, 127.15, 126.70, 126.18, 124.49, 122.31, 120.47, 120.44, 115.99, 115.95, 20.33; ^{19}F NMR (376 MHz, $CDCl_3$) δ -62.95; MS(ESI): m/z 406 $[M+H]^+$

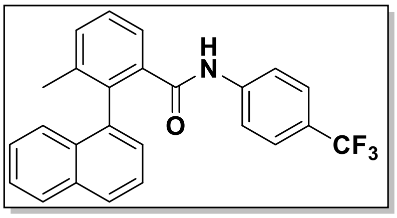

3-Methyl-2-(naphthalen-1-yl)-*N*-(4-(trifluoromethyl)phenyl)benzamide (3m)

White solid, 85% yield, 77% ee, $[\alpha]_D^{20}$ = -54.0, M.P.: 135.0 – 136.3 °C. The enantiomeric excess was determined by HPLC analysis using a chiral stationary phase column [Daicel Chiralcel® AD-H, 254 nm, *n*-hexane/iPrOH = 95:5 as the eluent, flow rate: 1 mL/min, retention times: 13.2 min (minor isomer) and 15.1 min (major isomer)]. ^{1}H NMR (400 MHz, $CDCl_3$, ppm) δ 7.98 (d, *J* = 8.1 Hz, 1H), 7.91 (d, *J* = 8.3 Hz, 1H), 7.87 (dd, *J* = 8.5, 4.5 Hz, 1H), 7.60 – 7.56 (m, 1H), 7.56 – 7.52 (m, 1H), 7.53 – 7.50 (m, 4H), 7.39 (dd, *J* = 6.9, 0.9 Hz, 1H), 7.28 (m, 2H), 7.06 (s, 1H), 6.70 (m, 2H), 2.01 (s, 3H); ^{13}C NMR (101 MHz, $CDCl_3$, ppm) δ 166.75, 140.38, 138.06, 136.72, 136.66, 136.27, 133.81, 132.78, 131.57, 129.02, 128.70, 128.40, 127.67, 127.46, 127.15, 126.70, 126.16, 125.86 (q, *J* = 12.0 Hz), 124.55, 118.72, 20.32; ^{19}F NMR (376 MHz, $CDCl_3$) δ -62.20; MS(ESI): m/z 406 [M+H]$^+$

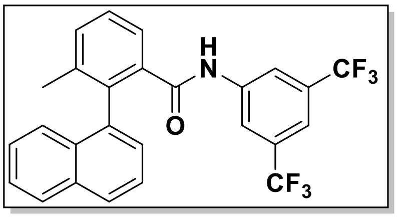

***N*-(3,5-bis(trifluoromethyl)phenyl)-3-methyl-2-(naphthalen-1-yl)benzamide (3n)**

White solid, 95% yield, 75% ee, $[\alpha]_D^{20}$ = -83.8, M.P.: 205.0 – 206.6 °C. The enantiomeric excess was determined by HPLC analysis using a chiral stationary phase column [Daicel Chiralcel® OD-H, 254 nm, *n*-hexane/iPrOH = 95:5 as the eluent, flow rate: 1 mL/min, retention times: 8.6 min (minor isomer) and 9.3 min (major isomer)]. ^{1}H NMR (400 MHz, $CDCl_3$, ppm) δ 8.00 (d, *J* = 8.0 Hz, 1H), 7.92 (d, *J* = 8.3 Hz, 1H), 7.88 (dd, *J* = 6.9, 1.4 Hz, 1H), 7.63 – 7.59 (m, 1H), 7.58 (m, 1H), 7.54 (m, 4H), 7.38 (d, *J* = 7.0 Hz, 1H), 7.36 (s, 1H), 7.11 (s, 1H), 6.93 (s, 2H), 2.06 (s, 3H); ^{13}C NMR (101 MHz, $CDCl_3$, ppm) δ 166.82, 138.58, 138.11, 136.81, 136.73, 136.59, 135.76, 133.89, 133.10, 131.98, 131.65, 131.50, 129.18, 128.78, 128.54, 128.02, 127.57, 127.25, 126.88, 126.31, 124.30, 121.50, 118.83 (q, *J* = 16 Hz), (q, *J* = 16 Hz), 20.32; ^{19}F NMR (377 MHz, $CDCl_3$, ppm) δ -63.23; MS(ESI): m/z 474 [M+H]$^+$

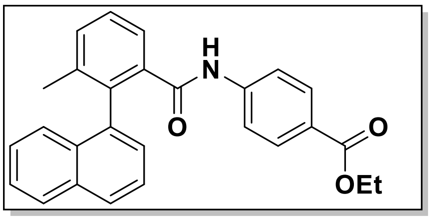

Ethyl-4-(3-methyl-2-(naphthalen-1-yl)benzamido)benzoate (3o)

White solid, 60% yield, 88% ee, $[\alpha]_D^{20}$ = -141.0, M.P.: 86.9 – 87.5 °C. The enantiomeric excess was determined by HPLC analysis using a chiral stationary phase column [Daicel Chiralcel® OD-H, 254 nm, *n*-hexane/iPrOH = 95:5 as the eluent, flow rate: 1 mL/min, retention times: 14.5 min (minor isomer) and 18.2 min (major isomer)]. ^{1}H NMR (400 MHz, $CDCl_3$ ppm) δ 7.97 (d, *J* = 8.1 Hz, 1H), 7.92 – 7.85 (m, 2H), 7.71 (m, 2H), 7.60 – 7.53 (m, 2H), 7.51 (m, 4H), 7.39 (dd, *J* = 6.9, 0.8 Hz, 1H), 7.10

(s, 1H), 6.67 (m, 2H), 4.28 (q, J = 7.1 Hz, 2H), 2.02 (s, 3H), 1.32 (t, J = 7.1 Hz, 3H); ^{13}C NMR (101 MHz, $CDCl_3$, ppm) δ 166.71, 166.02, 141.45, 138.03, 136.76, 136.64, 136.44, 133.81, 132.71, 131.57, 130.37, 129.02, 128.67, 128.36, 127.63, 127.41, 127.13, 126.65, 126.13, 125.56, 124.55, 118.14, 60.72, 20.33, 14.31; MS(ESI): m/z 410 $[M+H]^{+.}$

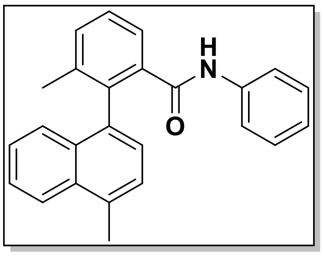

3-Methyl-2-(4-methylnaphthalen-1-yl)-*N*-phenylbenzamide (3p)

White solid, 73% yield, 75% ee, $[\alpha]_D^{20}$ = -57.3, M.P.: 143.3 – 143.9 °C. The enantiomeric excess was determined by HPLC analysis using a chiral stationary phase column [Daicel Chiralcel® OD-H, 254 nm, *n*-hexane/iPrOH = 95:5 as the eluent, flow rate: 1 mL/min, retention times: 11.0 min (minor isomer) and 12.8 min (major isomer)]. ^{1}H NMR (400 MHz, $CDCl_3$, ppm) δ 8.11 (d, J = 8.4 Hz, 1H), 7.90 – 7.85 (m, 1H), 7.62 – 7.54 (m, 1H), 7.52 (d, J = 7.7 Hz, 1H), 7.50 – 7.45 (m, 3H), 7.38 (d, J = 7.1 Hz, 1H), 7.28 (d, J = 7.1 Hz, 1H), 7.02 (m, 2H), 6.96 (s, 1H), 6.88 (m, 1H), 6.61 (m, 2H), 2.73 (s, 3H), 2.00 (s, 3H); ^{13}C NMR (101 MHz, $CDCl_3$, ppm) δ 166.64, 138.05, 137.53, 136.99, 136.90, 135.00, 134.95, 133.01, 132.31, 131.74, 128.54, 128.13, 127.34, 127.04, 126.82, 126.35, 125.32, 125.07, 123.87, 119.36, 20.38, 19.49; MS(ESI): m/z 352 $[M+H]^+$

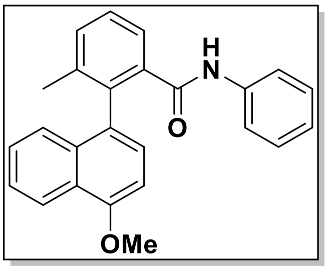

2-(4-Methoxynaphthalen-1-yl)-3-methyl-N-phenylbenzamide (3q)

White solid, 56% yield, 72% ee, $[\alpha]_D^{20}$ = -52.3, M.P.: 218.6 – 219.5 °C. The enantiomeric excess was determined by HPLC analysis using a chiral stationary phase column [Daicel Chiralcel® IA-3, 254 nm, *n*-hexane/iPrOH = 90:10 as the eluent, flow rate: 1 mL/min, retention times: 11.1 min (major isomer) and 12.7 min (minor isomer)]. ^{1}H NMR (400 MHz, $CDCl_3$, ppm) δ 8.39 (d, J = 8.2 Hz, 1H), 7.91 – 7.79 (m, 1H), 7.53 (m, 2H), 7.49 – 7.42 (m, 3H), 7.29 (d, J = 7.8 Hz, 1H), 7.04 (m, 3H), 6.90 (d, J = 7.4 Hz, 1H), 6.87 (d, J = 7.7 Hz, 1H), 6.65 (m, 2H), 4.01 (s, 3H), 1.99 (s, 3H); ^{13}C NMR (101 MHz, $CDCl_3$) δ 166.84, 155.53, 138.51, 137.54, 137.19, 136.75, 132.52, 132.26, 128.65, 128.59, 128.09, 127.92, 127.28, 125.91, 125.79, 124.44, 123.89, 123.00, 119.41, 104.02, 55.64, 20.36; MS(ESI): m/z 368 $[M+H]^+$

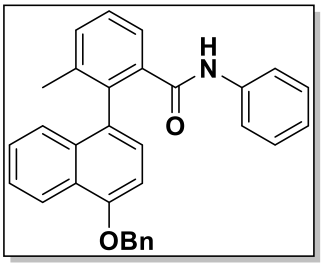

2-(4-(Benzyloxy)naphthalen-1-yl)-3-methyl-*N*-phenylbenzamide (3r)

White solid, 60% yield, 70% ee, $[\alpha]_D^{20}$ = 47.4, M.P.: 71.9 – 73.0 °C. The enantiomeric excess was determined by HPLC analysis using a chiral stationary phase column [Daicel Chiralcel® OD-H, 254 nm,

n-hexane/iPrOH = 90:10 as the eluent, flow rate: 1 mL/min, retention times: 11.8 min (minor isomer) and 13.5 min (major isomer)]. ^{1}H NMR (400 MHz, $CDCl_3$, ppm) δ 8.48 (d, *J* = 8.0 Hz, 1H), 7.91 – 7.80 (m, 1H), 7.57 – 7.48 (m, 4H), 7.47 (m, 3H), 7.44 – 7.34 (m, 3H), 7.26 (d, *J* = 4.7 Hz, 1H), 7.03 (m, 3H), 6.92 (m, 2H), 6.65 (m, 2H), 2.01 (s, 3H); ^{13}C NMR (101 MHz, $CDCl_3$, ppm) δ 166.80, 154.60, 138.48, 137.56, 137.21, 136.81, 136.70, 132.62, 132.27, 128.97, 128.67, 128.60, 128.12, 128.07, 127.99, 127.35, 127.31, 127.22, 126.08, 125.88, 124.48, 123.89, 123.22, 119.40, 105.39, 99.99, 70.25, 20.38; MS(ESI): m/z 444 $[M+H]^+$

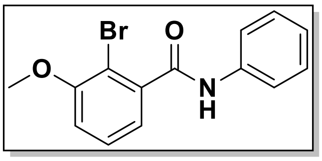

2-Bromo-3-methoxy-*N*-phenylbenzamide (4i)

^{1}H NMR (400 MHz, $CDCl_3$, ppm) δ 7.65 (m, 3H), 7.39 (m, 3H), 7.25 – 7.14 (m, 2H), 7.01 (d, *J* = 8.2 Hz, 1H), 3.95 (s, 3H); ^{13}C NMR (101 MHz, $CDCl_3$, ppm) δ 165.69, 156.23, 139.82, 137.56, 129.13, 128.79, 124.83, 121.10, 120.05, 113.25, 109.18, 56.62.

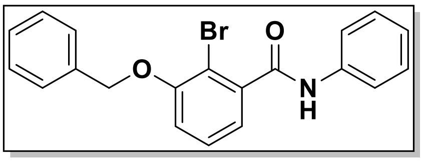

3-(Benzyloxy)-2-bromo-*N*-phenylbenzamide (4j)

^{1}H NMR (400 MHz, $CDCl_3$, ppm) δ 7.65 (m, 2H), 7.60 (s, 1H), 7.49 (m, 2H), 7.42 (m, 2H), 7.38 8.7 Hz(m, 2H), 7.35 (m, 1H), 7.32 (d, *J* = 8.0 Hz, 1H), 7.21 (dd, *J* = 7.7, 1.4 Hz, 1H), 7.18 (m, 1H), 7.03 (dd, *J* = 8.2, 1.2 Hz, 1H), 5.21 (s, 2H); ^{13}C NMR (101 MHz, $CDCl_3$, ppm) δ 165.64, 155.35, 139.91, 137.56, 136.07, 129.15, 128.69, 128.14, 127.02, 124.85, 121.46, 120.04, 115.09, 109.96, 71.19.

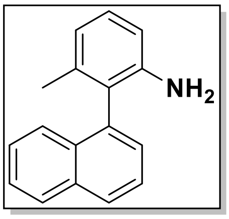

3-Methyl-2-(naphthalen-1-yl)aniline (5a)

Colourless oil : Known compound: ^{1}H NMR (400 MHz, $CDCl_3$) δ 7.90 (m, 2H), 7.57 (m, 1H), 7.54 – 7.46 (m, 2H), 7.44 – 7.36 (m, 2H), 7.17 (m, 1H), 6.79 (d, *J* = 7.5 Hz, 1H), 6.71 (d, *J* = 8.0 Hz, 1H), 1.89 (s, 3H); ^{13}C NMR (101 MHz, $CDCl_3$) δ 144.43, 137.98, 135.77, 134.09, 131.86, 128.38, 128.30, 127.90, 127.65, 126.40, 126.11, 126.06, 125.56, 125.33, 120.10, 112.91, 20.26.

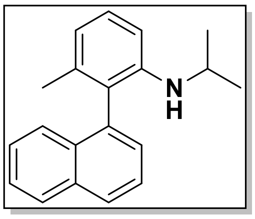

***N*-Isopropyl-3-methyl-2-(naphthalen-1-yl)aniline (5b)**

Yellow oil, 99% yield, 10% ee, $[\alpha]_D^{20}$ = 29.8, The enantiomeric excess was determined by HPLC analysis using a chiral stationary phase column [Daicel Chiralcel® OD-H, 254 nm, *n*-hexane/iPrOH = 99:1 as the eluent, flow rate: 1 mL/min, retention times: 5.9 min (major isomer) and 6.2 min (minor isomer)]. ^{1}H NMR (400 MHz, $CDCl_3$, ppm) δ 7.89 (m, 2H), 7.56 (m, 1H), 7.47 (m, 2H), 7.36 (m, 2H), 7.23 (m, 1H), 6.72 – 6.64 (m, 2H), 3.63 – 3.50 (m, 1H), 2.92 (s, 1H), 1.82 (s, 3H), 0.97 (d, *J* = 6.3 Hz, 3H), 0.89 (d, *J* = 6.3 Hz, 3H); ^{13}C NMR (101 MHz, $CDCl_3$, ppm) δ 145.67, 137.66, 134.11, 132.11, 128.42, 128.31, 128.11, 127.87, 126.25, 126.12, 125.51, 118.40, 109.17, 44.57, 22.97, 22.73, 20.49; MS(ESI): m/z 276 $[M+H]^+$

N-Benzyl-3-methyl-2-(naphthalen-1-yl)aniline (5c)

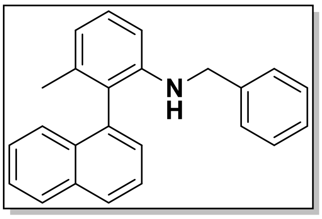

Yellow oil, 99% yield, 10% ee, $[\alpha]_D^{20}$ = 1.7, The enantiomeric excess was determined by HPLC analysis using a chiral stationary phase column [Daicel Chiralcel® IA-3, 254 nm, *n*-hexane/iPrOH = 99:1 as the eluent, flow rate: 0.8 mL/min, retention times: 6.08 min (major isomer) and 9.1 min (minor isomer)]. ^{1}H NMR (400 MHz, $CDCl_3$, ppm) δ 7.94 (m, 2H), 7.62 (d, *J* = 7.0 Hz, 1H), 7.59 (d, *J* = 8.1 Hz, 1H), 7.56 – 7.52 (m, 1H), 7.47 – 7.42 (m, 2H), 7.28 – 7.25 (m, 1H), 7.24 (d, *J* = 5.8 Hz, 1H), 7.20 (m, 2H), 7.17 – 7.13 (m, 2H), 6.76 (d, *J* = 7.5 Hz, 1H), 6.59 (d, *J* = 8.2 Hz, 1H), 4.26 (s, 2H), 1.90 (s, 3H); ^{13}C NMR (101 MHz, $CDCl_3$, ppm) δ 145.69, 139.53, 137.60, 135.61, 134.18, 132.14, 128.46, 128.41, 128.05, 128.03, 126.88, 126.39, 126.21, 126.17, 125.49, 119.11, 108.68, 48.06, 20.35; MS(ESI): m/z 324 $[M+H]^+$

N-(3-Methyl-2-(naphthalen-1-yl)phenyl)benzamide (5d)

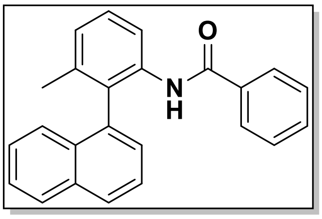

White solid, 94% yield, 22% ee, $[\alpha]_D^{20}$ = 31.0, M.P.: 111.9 - 112.7 °C. The enantiomeric excess was determined by HPLC analysis using a chiral stationary phase column [Daicel Chiralcel® AD-H, 254 nm, *n*-hexane/iPrOH = 97:3 as the eluent, flow rate: 1 mL/min, retention times: 18.2 min (minor isomer) and 22.4 min (major isomer)]. ^{1}H NMR (400 MHz, $CDCl_3$, ppm) δ 8.33 (d, *J* = 8.2 Hz, 1H), 7.95 (m,,2H), 7.61 (m, 1H), 7.55 – 7.44 (m, 5H), 7.40 (m, 1H), 7.30 (m, 1H), 7.15 (m, 2H), 7.07 (m, 2H), 6.88 (d, *J* = 8.3 Hz, 1H), 3.67 (s, 3H); ^{13}C NMR (101 MHz, $CDCl_3$, ppm) δ 164.83, 157.57, 137.35, 134.77, 134.01, 132.04, 131.48, 131.31, 129.64, 128.90, 128.66, 128.53, 126.88, 126.52, 126.42, 125.85, 125.46, 118.51, 113.05, 107.01, 55.90; MS(ESI): m/z 338 $[M+H]^+$

Methyl 3-methyl-2-(naphthalen-1-yl)benzoate (5e)

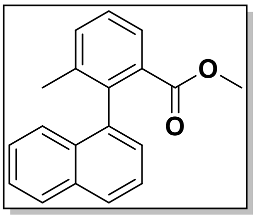

White solid, 77% yield, 35% ee, $[\alpha]_D^{20}$ = -10.7, M.P.: 79.0 - 80.1 °C. The enantiomeric excess was determined by HPLC analysis using a chiral stationary phase column [Daicel Chiralcel® AD-H, 254 nm, *n*-hexane/iPrOH = 95:5 as the eluent, flow rate: 1 mL/min, retention times: 5.5 min (minor isomer) and 6.0 min (major isomer)]. ^{1}H NMR (400 MHz, $CDCl_3$, ppm) δ 7.97 (d, *J* = 8.2 Hz, 1H), 7.94 (m, 2H), 7.60 (d, *J* = 7.3 Hz, 1H), 7.55 (m, 2H), 7.50 (d, *J* = 7.9 Hz, 1H), 7.43 (m, 2H), 7.31 (d, *J* = 7.0 Hz, 1H), 3.42 (s, 3H), 2.03 (s, 3H); ^{13}C NMR (101 MHz, $CDCl_3$, ppm) δ 168.17, 140.43, 138.43, 138.34, 133.40, 132.11, 128.35, 127.55, 127.54, 127.47, 126.09, 125.86, 125.70, 125.51, 125.37, 51.70, 20.40; MS(ESI): m/z 299 $[M+Na]^+$

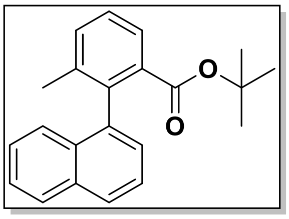

***tert*-Butyl 3-methyl-2-(naphthalen-1-yl)benzoate (5f)**

Off white solid, 90% yield, 70% ee, $[\alpha]_D^{20}$ = 4.2, M.P.: 86.2 - 87.4 °C. The enantiomeric excess was determined by HPLC analysis using a chiral stationary phase column [Daicel Chiralcel® AD-H, 254 nm, *n*-hexane/iPrOH = 97:3 as the eluent, flow rate: 1 mL/min, retention times: 4.32 min (major isomer) and 4.65 min (minor isomer)]. ^{1}H NMR (400 MHz, $CDCl_3$ ppm) δ 7.77 (dd, *J* = 7.3, 1.5 Hz, 1H), 7.53 (m, 1H), 7.52 – 7.48 (m, 1H), 7.48 – 7.42 (m, 3H), 7.42 – 7.36 (m, 1H), 7.26 (dd, *J* = 7.0, 1.0 Hz, 1H), 1.99 (s, 3H), 0.79 (s, 9H); ^{13}C NMR (101 MHz, $CDCl_3$ ppm) δ 168.18, 138.95, 138.71, 137.89, 134.91, 133.49, 132.45, 132.43, 128.05, 127.49, 127.27, 126.92, 126.15, 126.10, 126.00, 125.71, 125.37, 80.66, 27.09, 20.36; MS(ESI): m/z 319 $[M+H]^+$

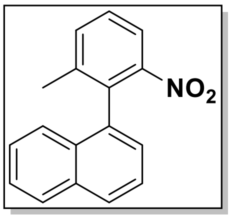

1-(2-Methyl-6-nitrophenyl)naphthalene (5g)

Yellow solid, 98% yield, 75% ee, $[\alpha]_D^{20}$ = 121.1, M.P.: 98.0 - 99.1 °C. The enantiomeric excess was determined by HPLC analysis using a chiral stationary phase column [Daicel Chiralcel® OD-H, 254 nm, *n*-hexane/iPrOH = 95:5 as the eluent, flow rate: 1 mL/min, retention times: 6.44 min (minor isomer) and 7.4 min (major isomer)]. ^{1}H NMR (400 MHz, $CDCl_3$, ppm) δ 7.89 (m, 2H), 7.82 (d, *J* = 8.0 Hz, 1H), 7.55 (d, *J* = 7.6 Hz, 1H), 7.48 (m, 3H), 7.39 (m, 1H), 7.33 (d, *J* = 8.4 Hz, 1H), 7.24 (d, *J* = 6.9 Hz, 1H), 1.97 (s, 3H); ^{13}C NMR (101 MHz, $CDCl_3$, ppm) δ 150.79, 140.34, 133.99, 133.96, 133.93, 133.54, 131.61, 128.55, 128.50, 128.24, 126.62, 126.08, 125.96, 125.43, 124.91, 121.43, 20.27; MS(ESI): m/z 286 $[M+Na]^+$

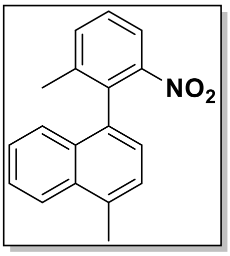

1-Methyl-4-(2-methyl-6-nitrophenyl)naphthalene (5h)

Yellow solid, 97% yield, 83% ee, $[\alpha]_D^{20}$ = 65.5, M.P.: 84.4 - 85.2 °C. The enantiomeric excess was determined by HPLC analysis using a chiral stationary phase column [Daicel Chiralcel® OD-H, 254 nm, *n*-hexane/iPrOH = 98:2 as the eluent, flow rate: 1 mL/min, retention times: 6.74 min (minor isomer) and 7.22 min (major isomer)]. ^{1}H NMR (400 MHz, $CDCl_3$ ppm) δ 8.10 (d, *J* = 8.4 Hz, 1H), 7.83 (d, *J* = 8.0 Hz, 1H), 7.58 (m, 1H), 7.57 – 7.53 (m, 1H), 7.50 (m, 1H), 7.47 – 7.41 (m, 1H), 7.41 – 7.35 (m, 2H), 7.17 (d, *J* = 7.1 Hz, 1H), 2.78 (s, 3H), 2.02 (s, 3H); ^{13}C NMR (101 MHz, $CDCl_3$ ppm) δ 150.99, 140.48, 134.88, 134.21, 133.78, 132.72, 132.09, 131.65, 128.10, 126.21, 125.87, 125.66, 125.52, 124.69, 121.30, 20.30, 19.56; MS(ESI): m/z 278 $[M+H]^+$

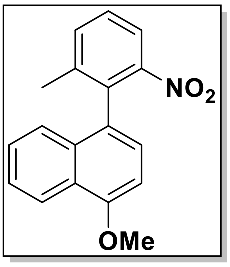

1-Methoxy-4-(2-methyl-6-nitrophenyl)naphthalene (5i)

Yellow solid, 98% yield, 85% ee, $[\alpha]_D^{20}$ = 72.7, M.P.: 87.8 - 89.1 °C. The enantiomeric excess was determined by HPLC analysis using a chiral stationary phase column [Daicel Chiralcel® OD-H, 254 nm, *n*-hexane/iPrOH = 98:2 as the eluent, flow rate: 1 mL/min, retention times: 6.57 min (minor isomer) and 8.09 min (major isomer)]. ^{1}H NMR (400 MHz, $CDCl_3$, ppm) δ 8.38 (d, *J* = 8.4 Hz, 1H), 7.79 (d, *J* = 8.0 Hz, 1H), 7.59 – 7.55 (m, 1H), 7.54 – 7.47 (m, 2H), 7.44 (dd, *J* = 7.9, 1.1 Hz, 1H), 7.31 (d, *J* = 8.3 Hz, 1H), 7.18 (dd, *J* = 7.8, 1.2 Hz, 1H), 6.87 (d, *J* = 7.9 Hz, 1H), 4.07 (s, 3H), 2.03 (s, 3H); ^{13}C NMR (101 MHz, $CDCl_3$ ppm) δ 155.62, 151.37, 140.84, 133.92, 133.62, 132.56, 128.04, 127.07, 126.08, 125.78, 125.69, 125.35, 124.74, 122.52, 121.16, 103.35, 55.53, 20.27; MS(ESI): m/z 294 $[M+H]^+$

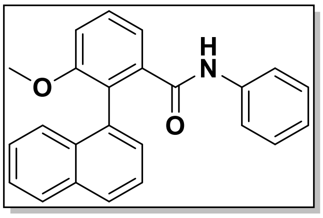

3-Methoxy-2-(naphthalen-1-yl)-*N*-phenylbenzamide (3s)

White solid, 65% yield, 18% ee, $[\alpha]_D^{20}$ = -1.4, M.P.: 74.0 – 74.9 °C. The enantiomeric excess was determined by HPLC analysis using a chiral stationary phase column [Daicel Chiralcel® OD-H, 254 nm, *n*-hexane/iPrOH = 95:5 as the eluent, flow rate: 1 mL/min, retention times: 11.8 min (minor isomer) and 13.1 min (major isomer)]. ^{1}H NMR (400 MHz, $CDCl_3$, ppm) δ 7.94 (d, *J* = 8.1 Hz, 1H), 7.90 (d, *J* = 8.2 Hz, 1H), 7.66 (d, *J* = 7.1 Hz, 1H), 7.56 (m, 2H), 7.55 – 7.50 (m, 2H), 7.48 (d, *J* = 8.1 Hz, 1H), 7.43 (d, *J* = 7.1 Hz, 1H), 7.18 (d, *J* = 8.2 Hz, 1H), 7.03 (m, 2H), 6.89 (m, 1H), 6.83 (s, 1H), 6.60 (m, 2H), 3.65 (s, 3H); ^{13}C NMR (101 MHz, $CDCl_3$, ppm) δ 166.08, 157.51, 137.97, 137.29, 133.96, 133.69, 132.16, 129.58, 128.71, 128.60, 128.58, 127.75, 127.05, 126.43, 126.33, 125.96, 125.11, 124.07, 121.80, 119.53, 113.16, 56.08; MS(ESI): m/z 354 $[M+H]^+$

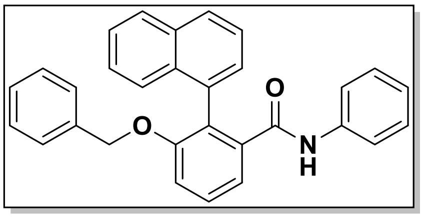

3-(Benzyloxy)-2-(naphthalen-1-yl)-*N*-phenylbenzamide (3t)

White solid, 60% yield, 23% ee, $[\alpha]_D^{20}$ = -8.6, M.P.: 93.3 – 94.5 °C. The enantiomeric excess was determined by HPLC analysis using a chiral stationary phase column [Daicel Chiralcel® IC-3, 254 nm, *n*-hexane/iPrOH = 90:10 as the eluent, flow rate: 1 mL/min, retention times: 22.9 min (minor isomer) and 24.6 min (major isomer)]. ^{1}H NMR (400 MHz, $CDCl_3$, ppm) δ 7.96 (d, *J* = 8.1 Hz, 1H), 7.91 (d, *J* = 8.2 Hz, 1H), 7.68 (d, *J* = 7.7 Hz, 1H), 7.64 (d, *J* = 8.3 Hz, 1H), 7.54 (m, 2H), 7.51 (d, *J* = 4.3 Hz, 1H), 7.47 (m, 2H), 7.19 (d, *J* = 8.2 Hz, 1H), 7.17 – 7.12 (m, 3H), 7.04 (m, 2H), 6.95 (s, 1H), 6.90 (m, 3H), 6.64 (m, 2H), 4.99 (s, 2H); ^{13}C NMR (101 MHz, $CDCl_3$, ppm) δ 165.93, 156.42, 137.92, 137.33, 136.64, 134.01, 133.72, 132.23, 129.49, 128.68, 128.59, 128.25, 127.78, 127.54, 127.28, 127.05, 126.46, 126.34, 125.92, 125.24, 124.08, 122.29, 119.55, 115.31, 70.48; MS(ESI): m/z 430 $[M+H]^+$

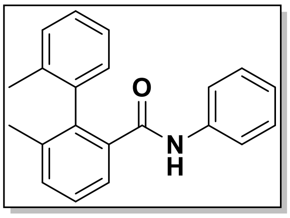

2',6-Dimethyl-*N*-phenyl-[1,1'-biphenyl]-2-carboxamide (3u)

White solid, 60% yield, 11% ee, $[\alpha]_D^{20}$ = 4.2, M.P.: 143.2 - 144.1 °C. The enantiomeric excess was determined by HPLC analysis using a chiral stationary phase column [Daicel Chiralcel® OD-H, 254 nm, *n*-hexane/iPrOH = 95:5 as the eluent, flow rate: 1 mL/min, retention times: 11.0 min (minor isomer) and 12.8 min (major isomer)]. ^{1}H NMR (400 MHz, $CDCl_3$, ppm) δ 7.95 (dd, *J* = 7.4, 1.2 Hz, 1H), 7.44 (dd, *J* = 7.4, 1.5 Hz, 1H), 7.42 (d, *J* = 7.4 Hz, 1H), 7.39 – 7.35 (m, 3H), 7.22 (d, *J* = 8.5 Hz, 1H), 7.18 (m, 2H), 7.07 – 7.02 (m, 2H), 7.02 – 6.98 (m, 1H), 2.06 (s, 3H), 2.04 (s, 3H); ^{13}C NMR (101 MHz, $CDCl_3$, ppm) δ 166.07, 138.92, 138.18, 137.73, 136.74, 136.54, 134.76, 132.79, 131.16, 129.03, 128.76, 128.61, 127.85, 127.71, 126.89, 124.13, 119.86, 20.44, 19.71; MS(ESI): m/z 302 $[M+H]^+$

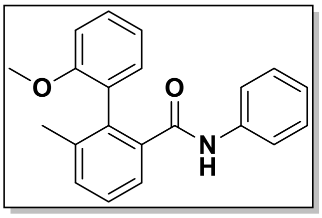

2'-Methoxy-6-methyl-*N*-phenyl-[1,1'-biphenyl]-2-carboxamide (3v)

White solid, 99% yield, 0% ee, $[\alpha]_D^{20}$ = 1.6, M.P.: 114.2 –115.8 °C. The enantiomeric excess was determined by HPLC analysis using a chiral stationary phase column [Daicel Chiralcel® OD-H, 254 nm, *n*-hexane/iPrOH = 95:5 as the eluent, flow rate: 1 mL/min, retention times: 11.8 min and 13.5 min]. ^{1}H NMR (400 MHz, $CDCl_3$, ppm) δ 7.70 (dd, *J* = 6.4, 1.8 Hz, 1H), 7.48 (s, 1H), 7.42 – 7.32 (m, 3H), 7.20 (m, 2H), 7.17 – 7.11 (m, 3H), 7.05 (d, *J* = 7.4 Hz, 1H), 7.03 – 6.98 (m, 2H), 3.82 (s, 3H), 2.06 (s, 3H); ^{13}C NMR (101 MHz, $CDCl_3$, ppm) δ 167.34, 156.04, 137.86, 137.62, 136.90, 135.18, 131.89, 130.80, 129.72, 128.79, 127.81, 126.35, 124.04, 121.67, 119.68, 111.08, 55.70, 20.36; MS(ESI): m/z 318 $[M+H]^+$

Copies of NMR spectra

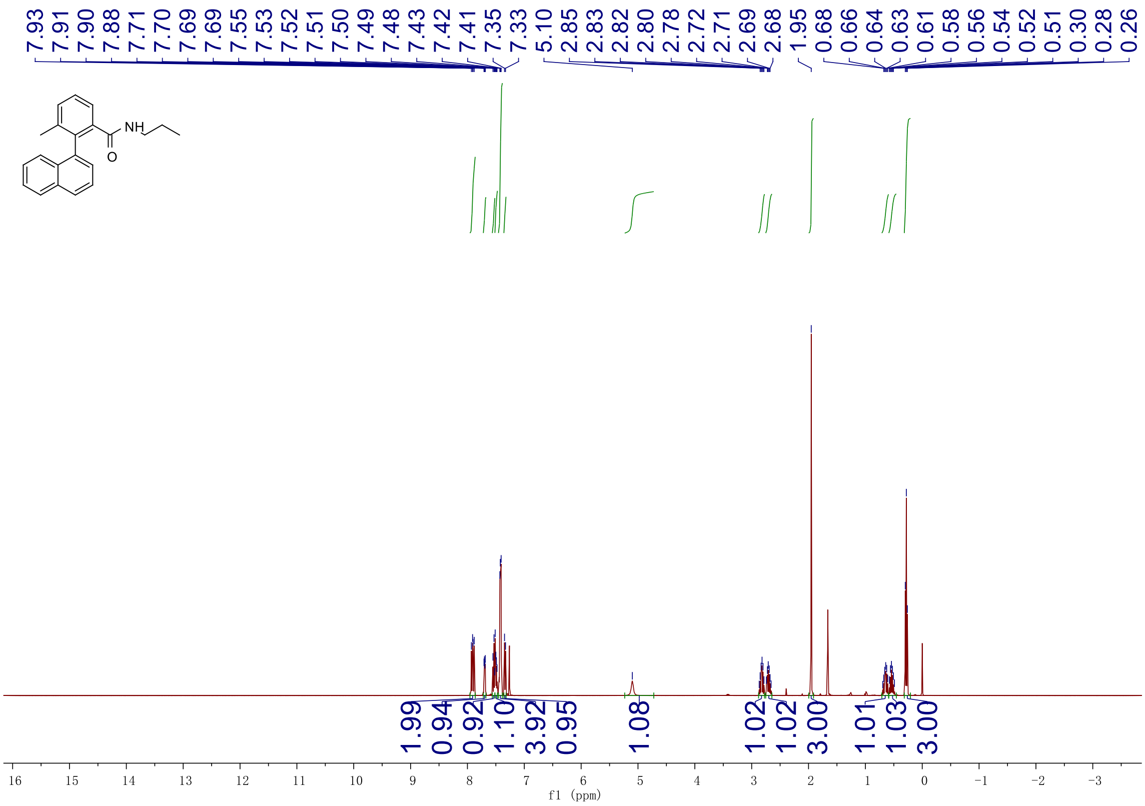

^{1}H NMR Spectrum of 3-methyl-2-(naphthalen-1-yl)-*N*-propylbenzamide (3a)

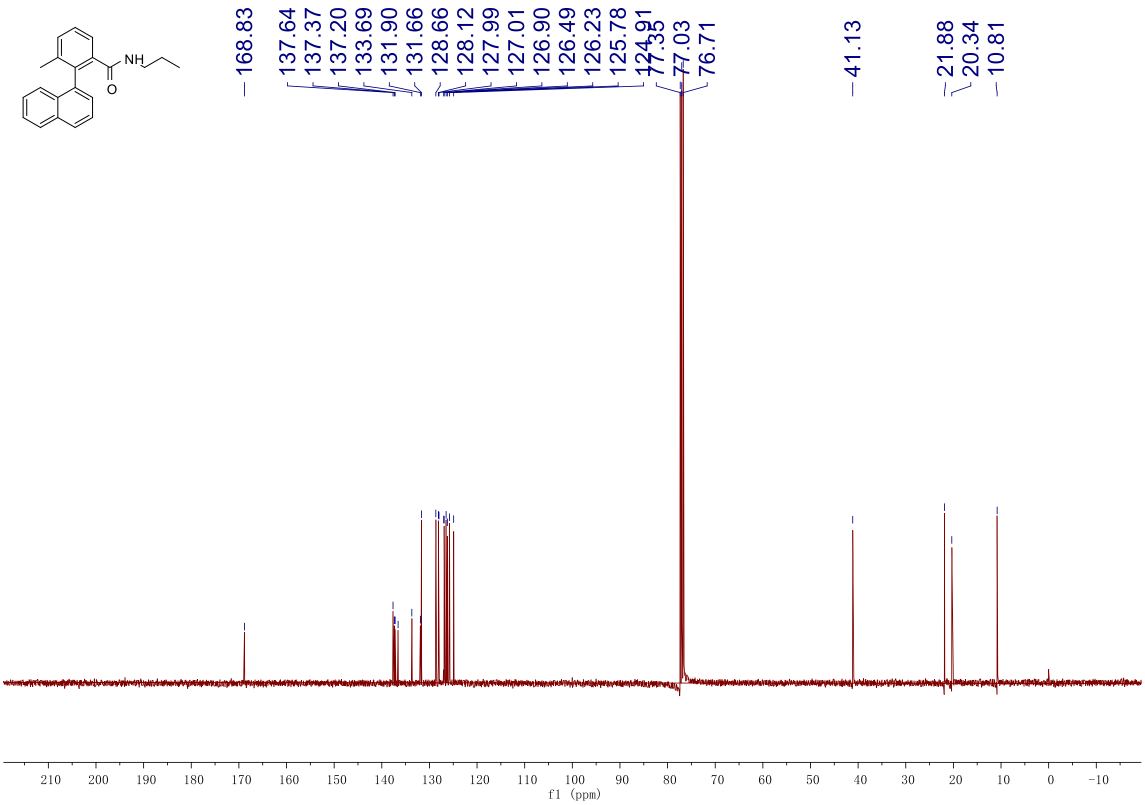

^{13}C NMR Spectrum of 3-methyl-2-(naphthalen-1-yl)-*N*-propylbenzamide (3a)

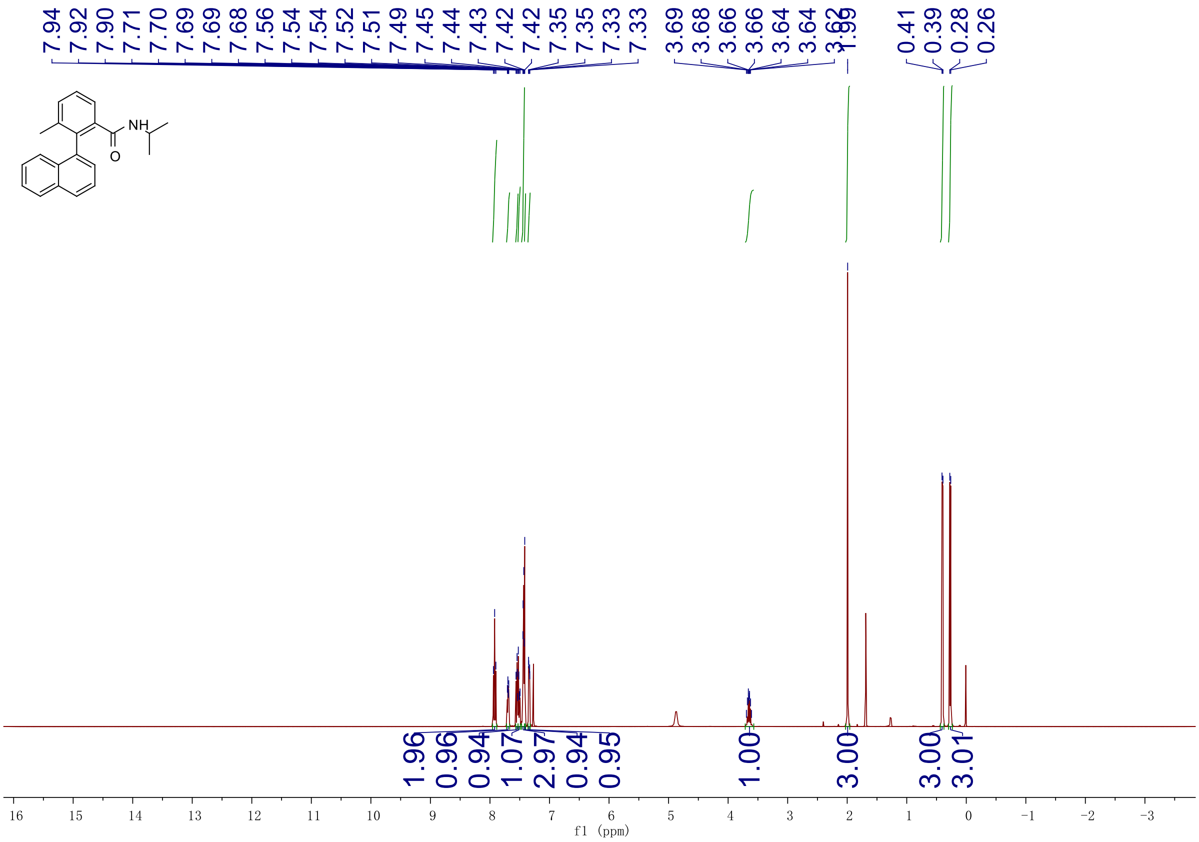

^{1}H NMR Spectrum of *N*-isopropyl-3-methyl-2-(naphthalen-1-yl)benzamide (3b)

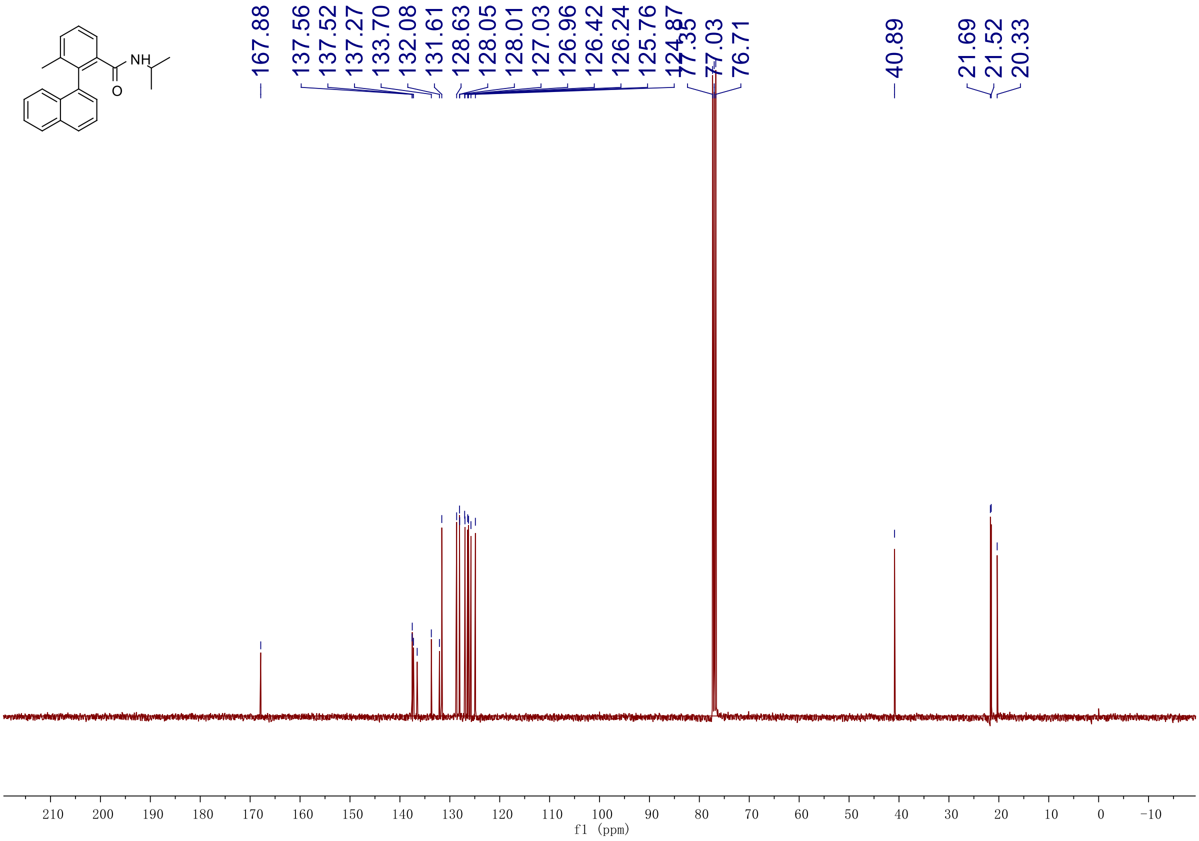

^{13}C NMR Spectrum of *N*-isopropyl-3-methyl-2-(naphthalen-1-yl)benzamide (3b)

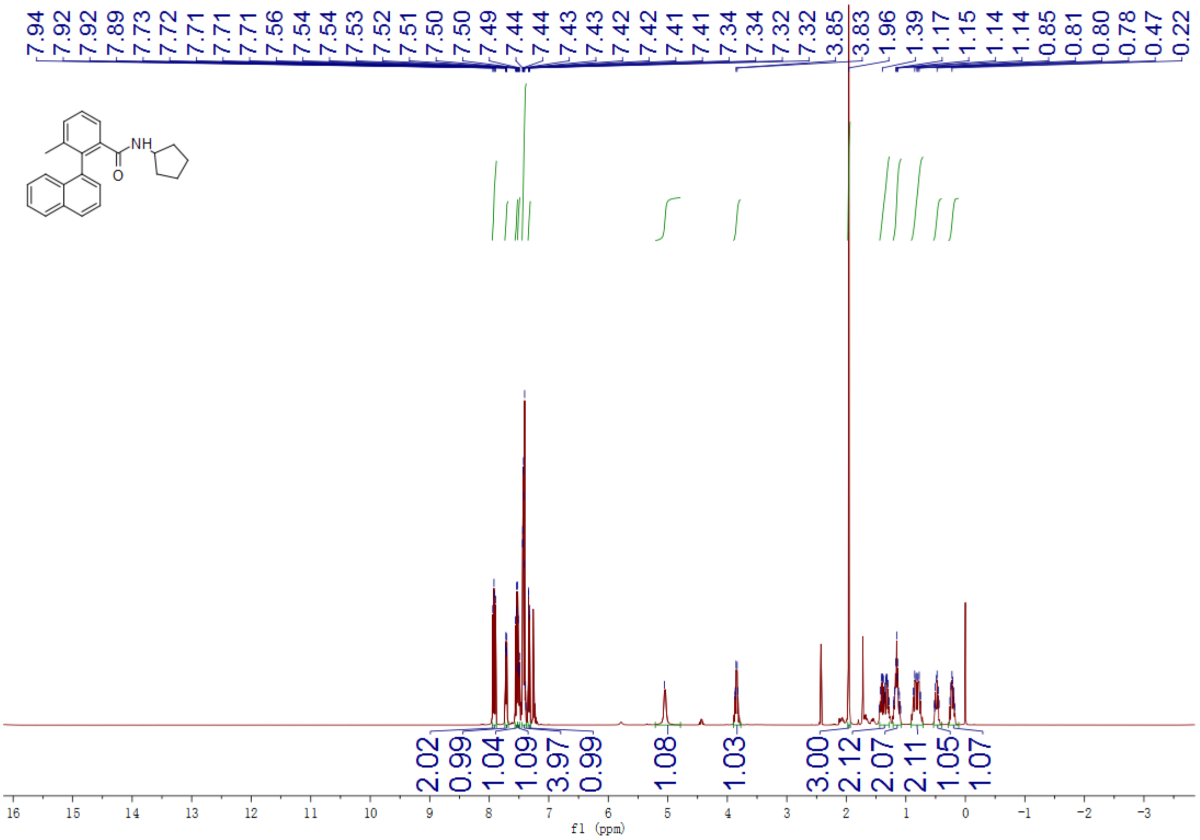

^{1}H NMR Spectrum of *N*-cyclopentyl-3-methyl-2-(naphthalen-1-yl)benzamide (3c)

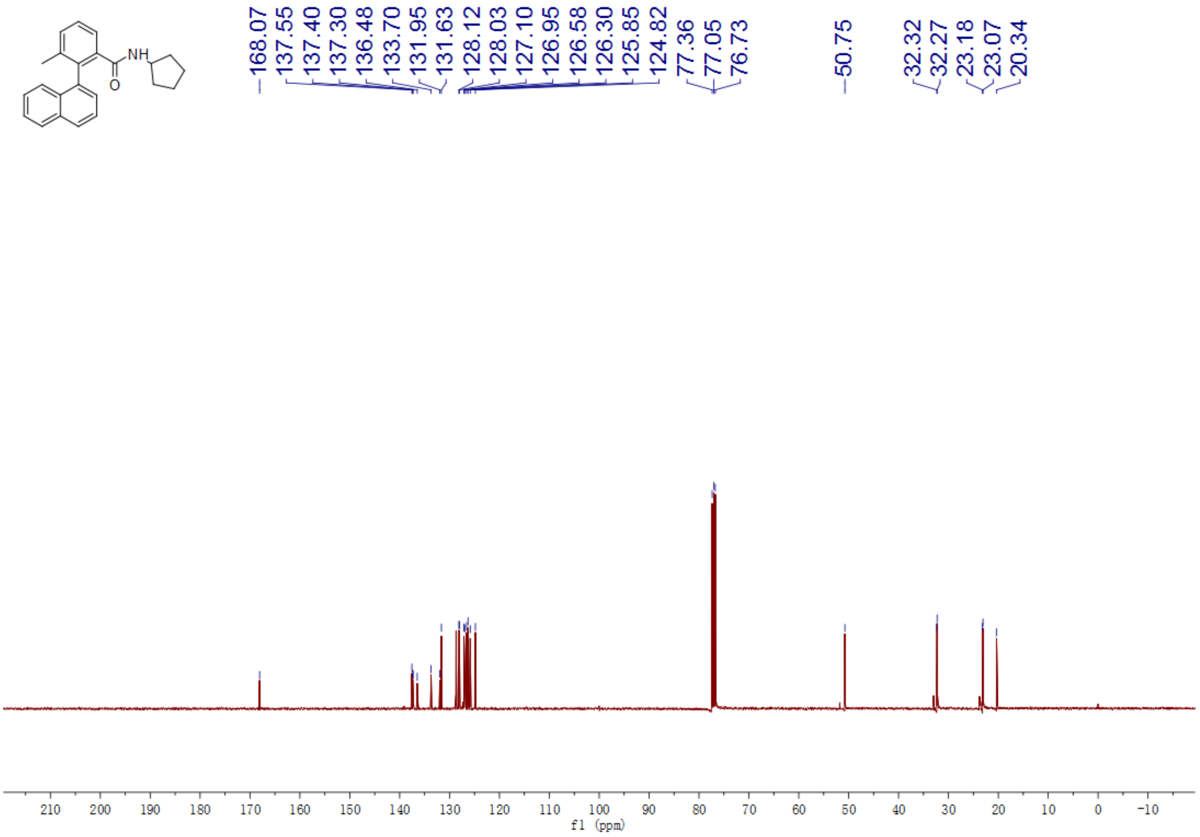

^{13}C NMR Spectrum of *N*-cyclopentyl-3-methyl-2-(naphthalen-1-yl)benzamide (3c)

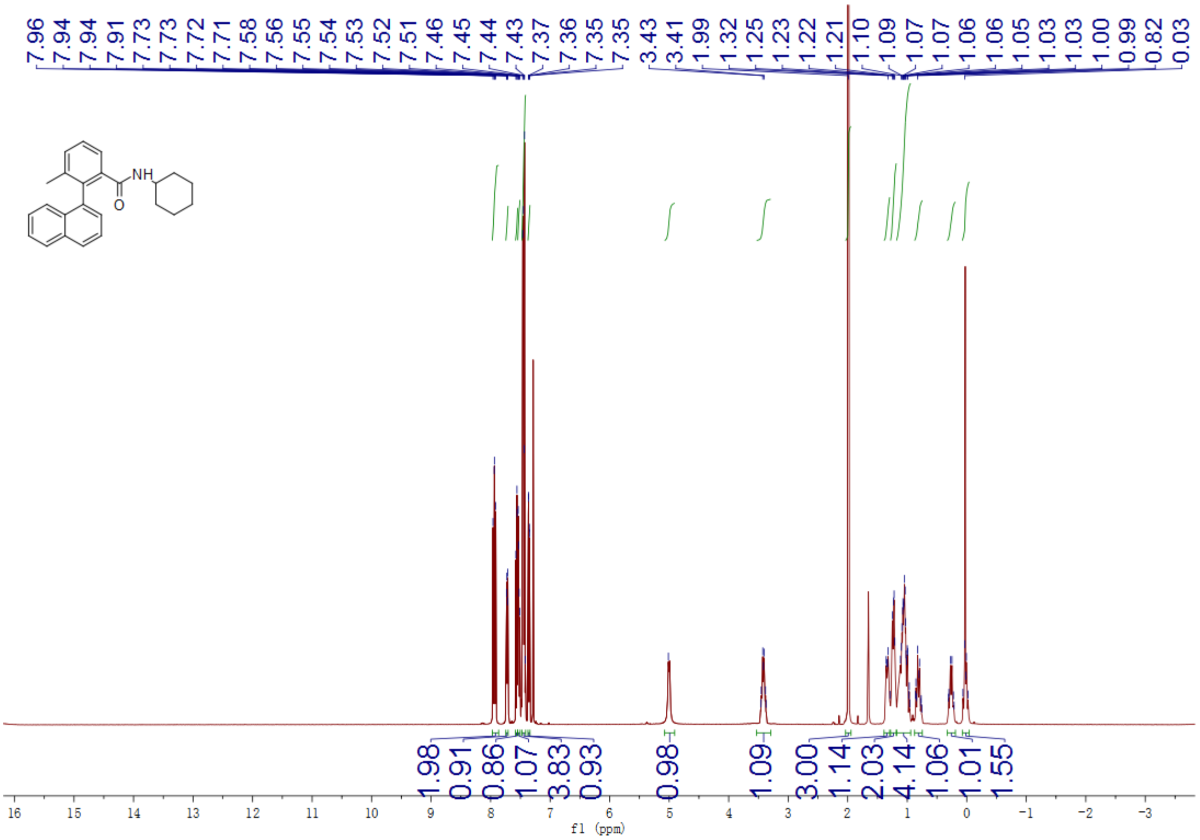

^{1}H NMR Spectrum of *N*-cyclohexyl-3-methyl-2-(naphthalen-1-yl)benzamide (3d)

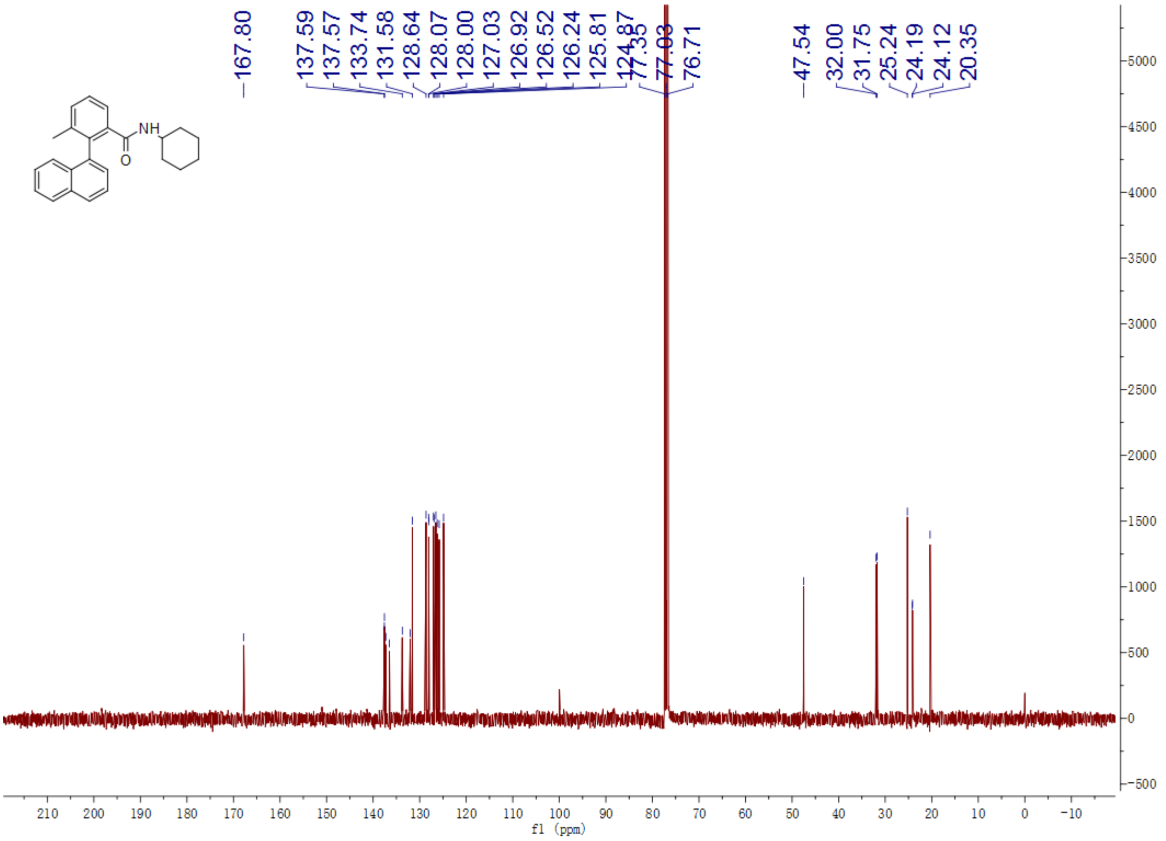

^{13}C NMR Spectrum of *N*-cyclohexyl-3-methyl-2-(naphthalen-1-yl)benzamide (3d)

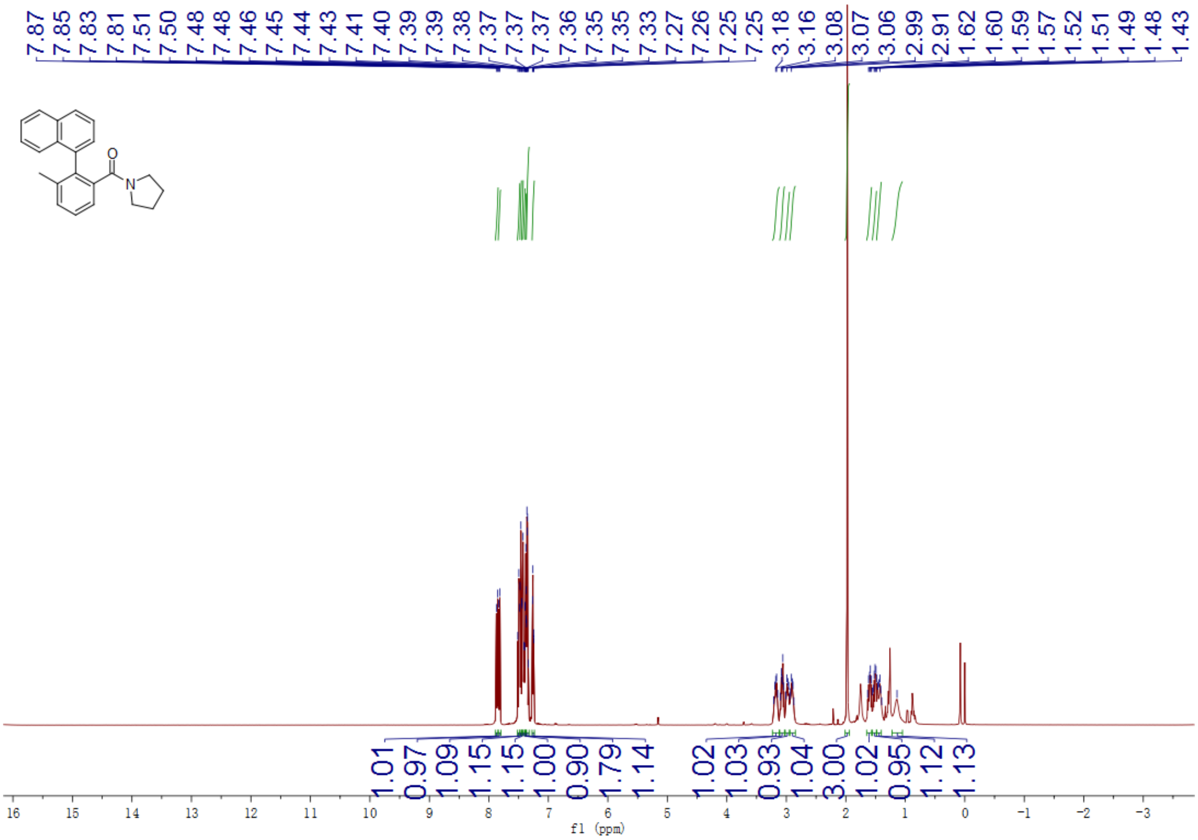

^{1}H NMR Spectrum of 3-methyl-2-(naphthalen-1-yl)phenyl)(pyrrolidin-1-yl)methanone (3e)

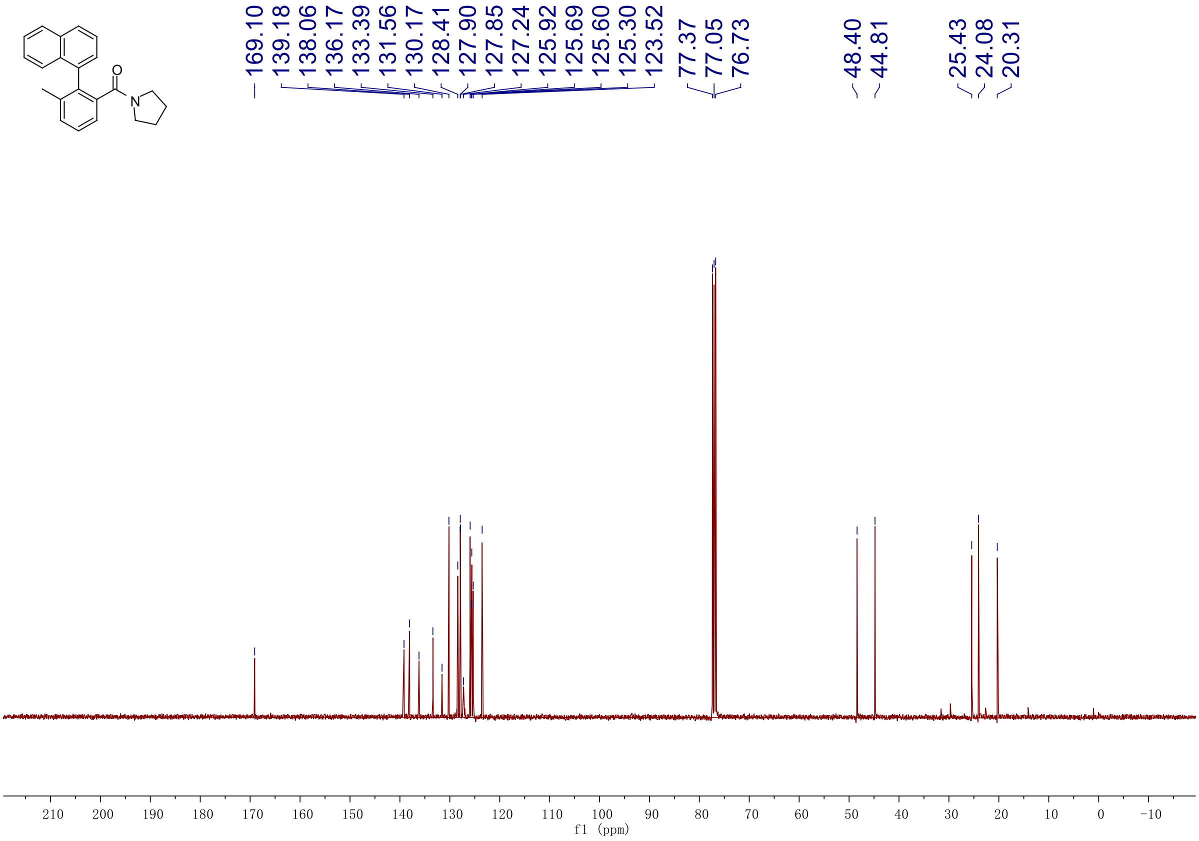

^{13}C NMR Spectrum of 3-methyl-2-(naphthalen-1-yl)phenyl)(pyrrolidin-1-yl)methanone (3e)

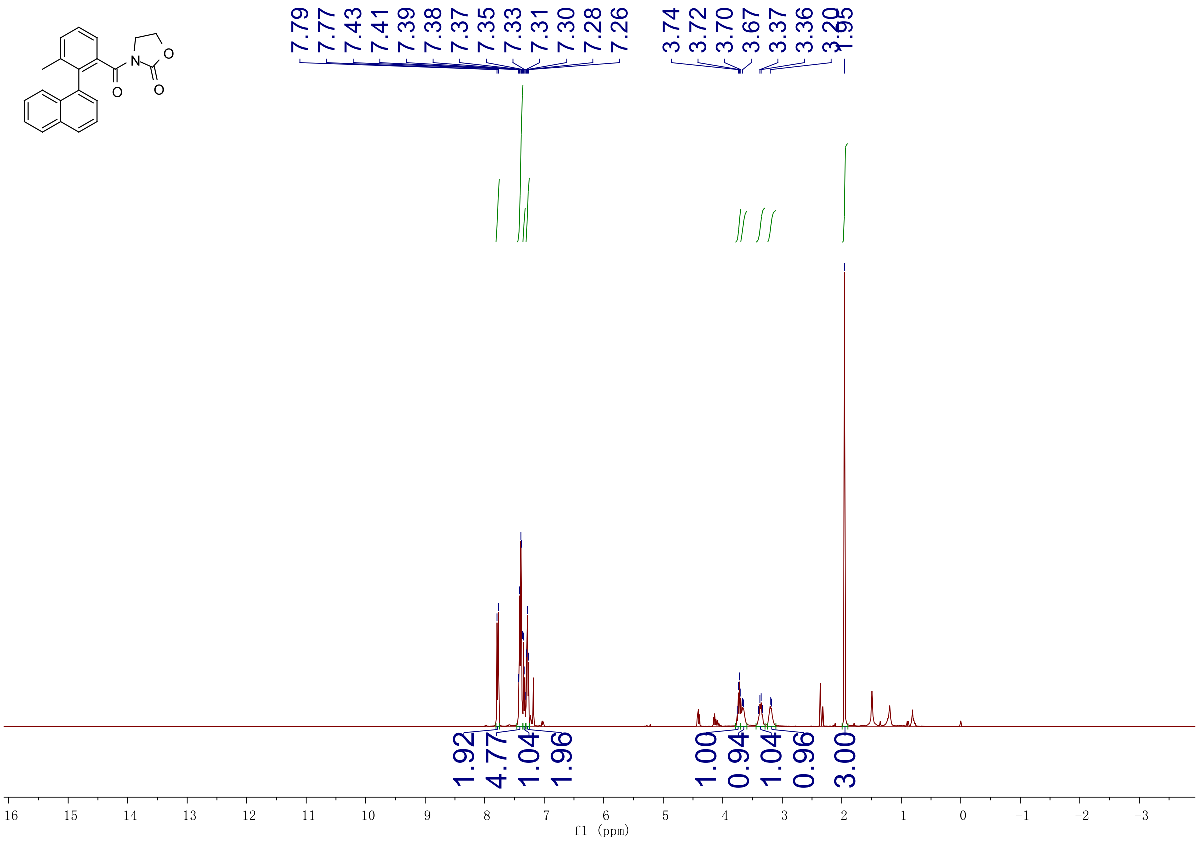

^{1}H NMR Spectrum of 3-(3-methyl-2-(naphthalen-1-yl)benzoyl)oxazolidin-2-one (3f)

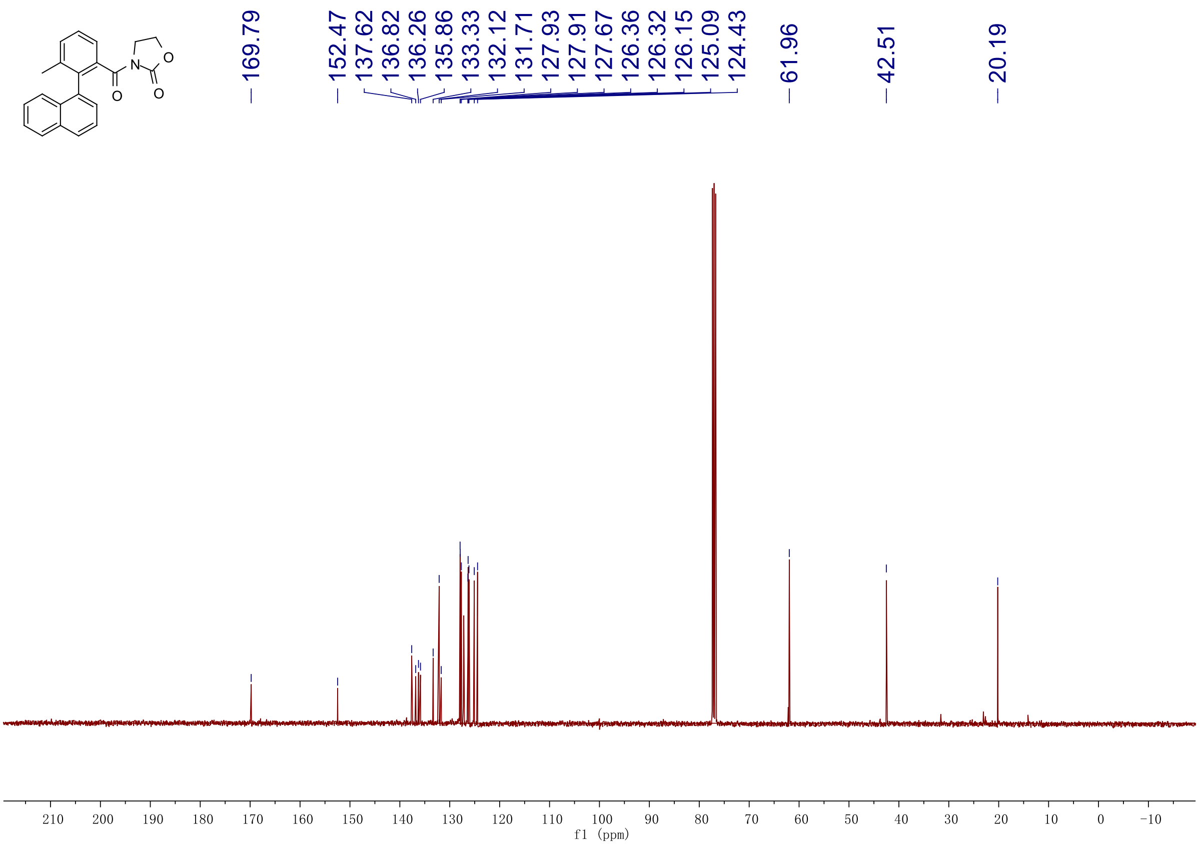

^{13}C NMR Spectrum of 3-(3-methyl-2-(naphthalen-1-yl)benzoyl)oxazolidin-2-one (3f)

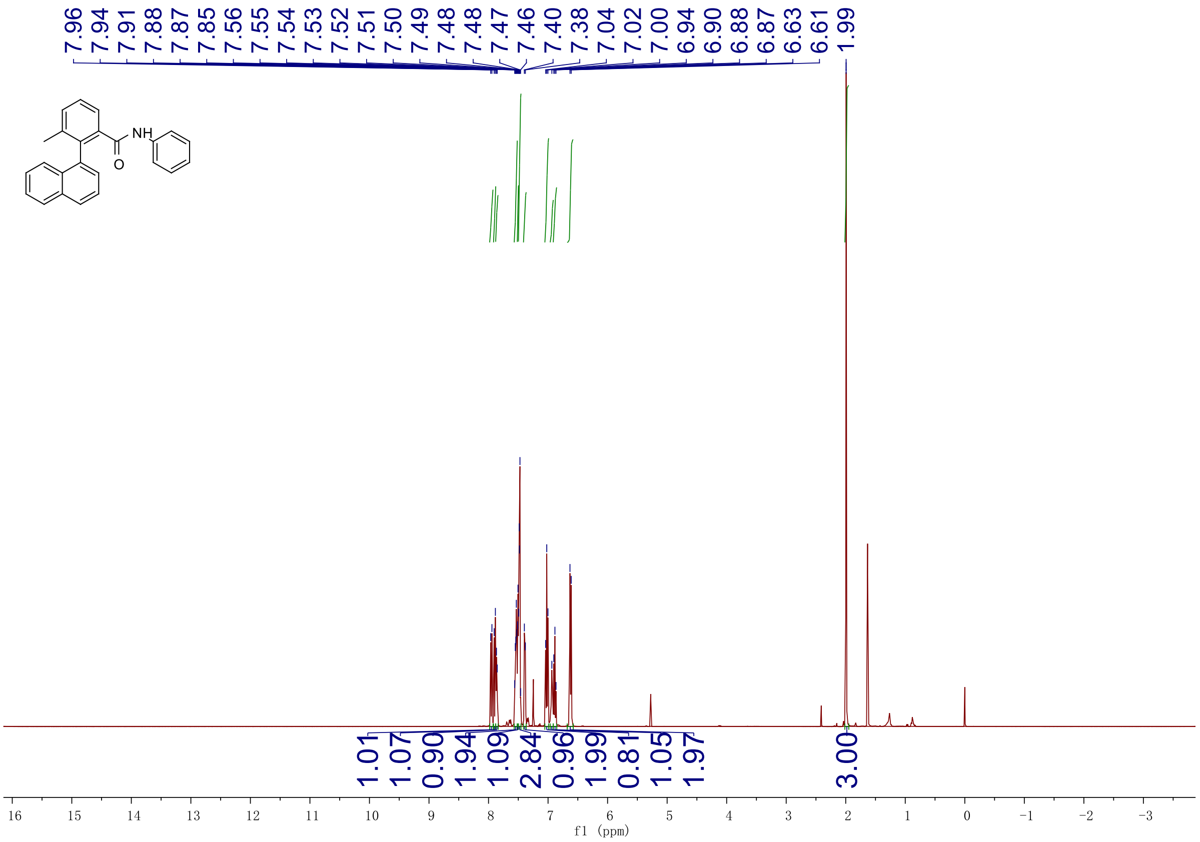

^{1}H NMR Spectrum of 3-methyl-2-(naphthalen-1-yl)-*N*-phenylbenzamide (3g)

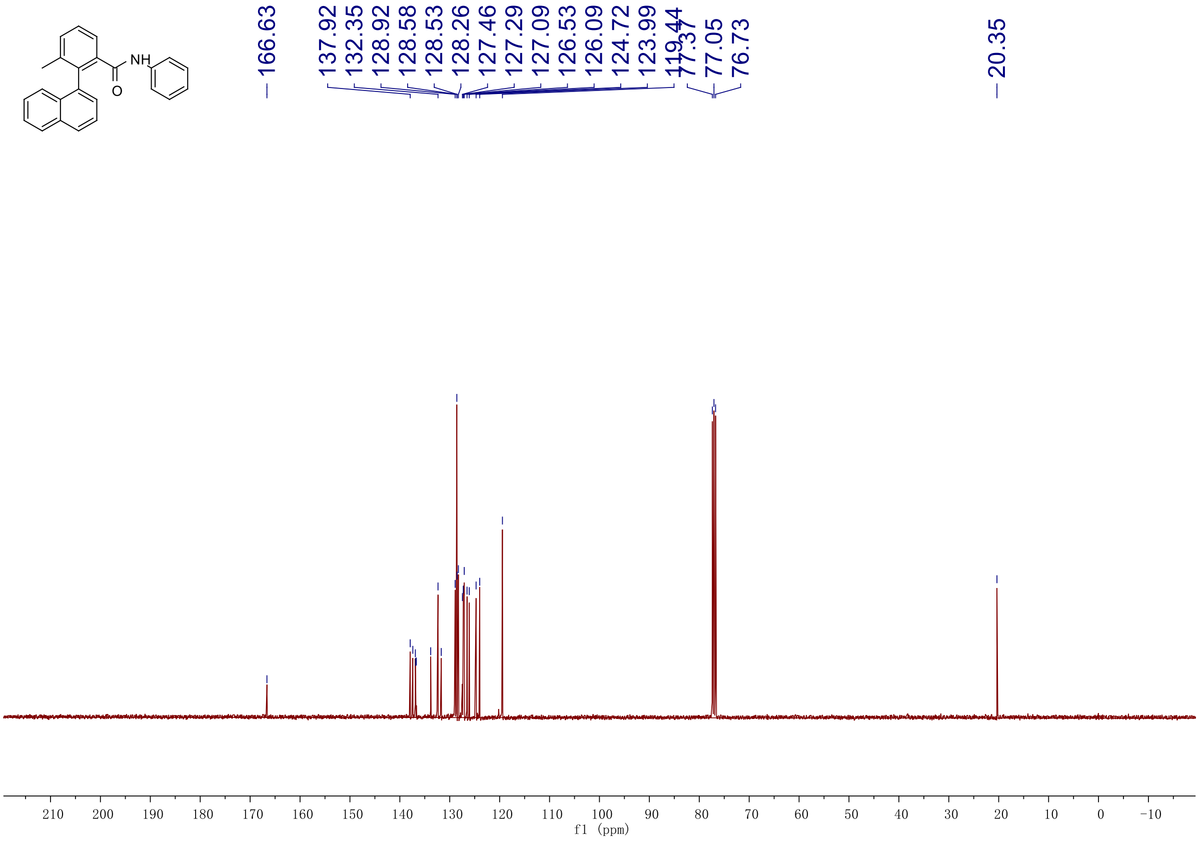

^{13}C NMR Spectrum of 3-methyl-2-(naphthalen-1-yl)-*N*-phenylbenzamide (3g)

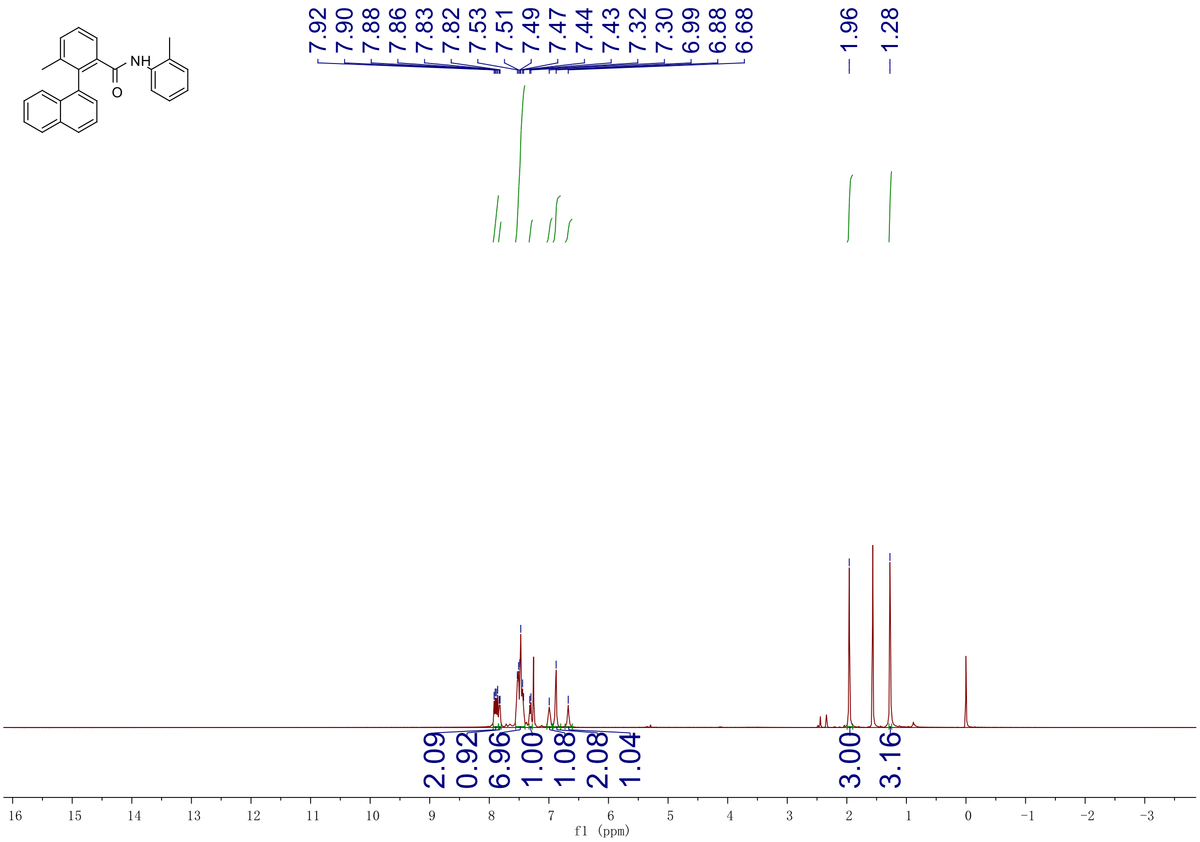

^{1}H NMR Spectrum of 3-methyl-2-(naphthalen-1-yl)-*N*-(*o*-tolyl)benzamide (3h)

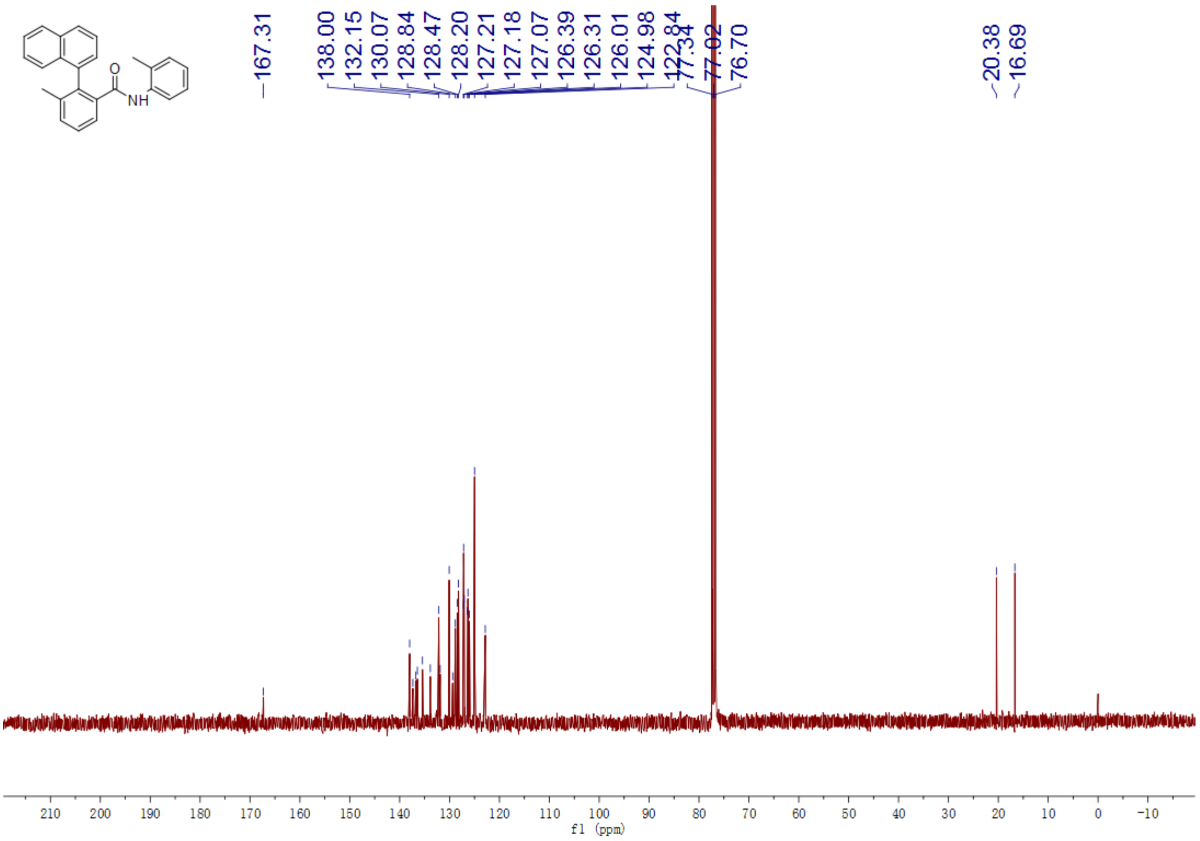

^{13}C NMR Spectrum of 3-methyl-2-(naphthalen-1-yl)-*N*-(*o*-tolyl)benzamide (3h)

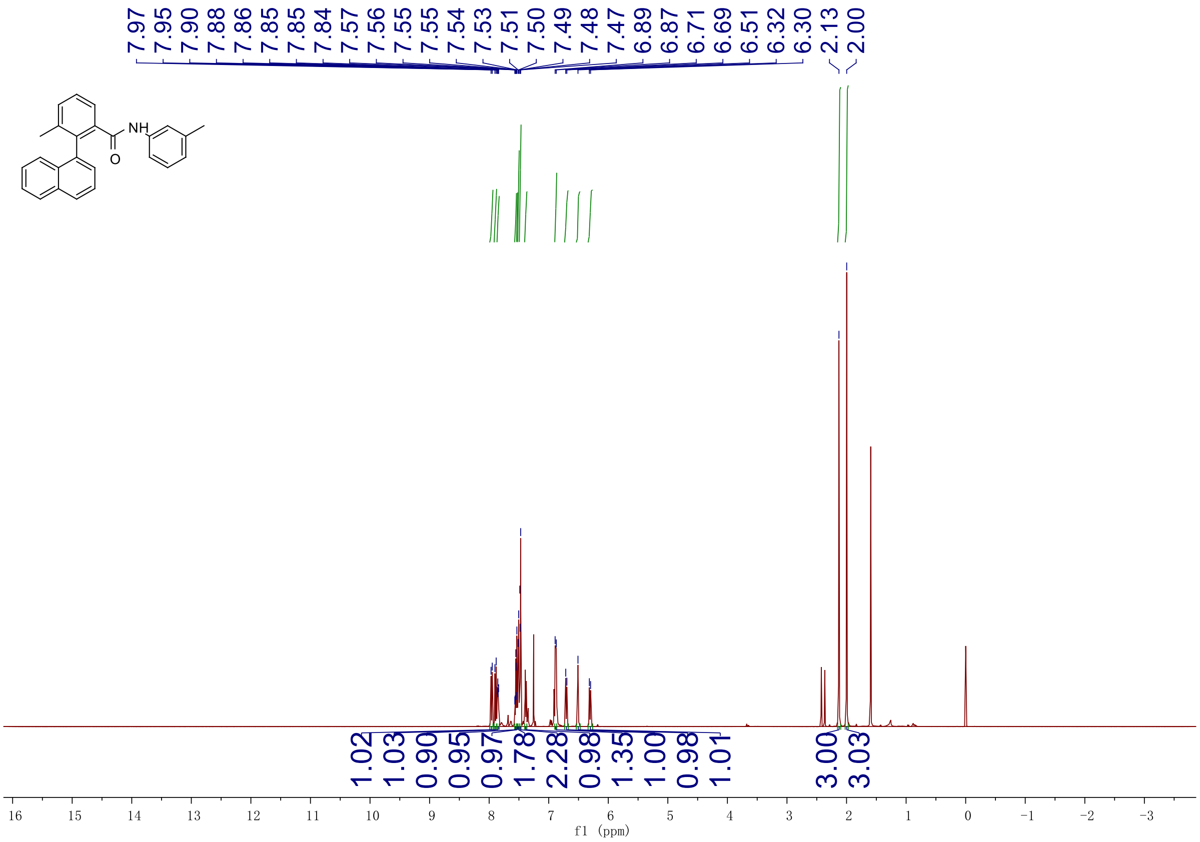

^{1}H NMR Spectrum of 3-methyl-2-(naphthalen-1-yl)-*N*-(*m*-tolyl)benzamide (3i)

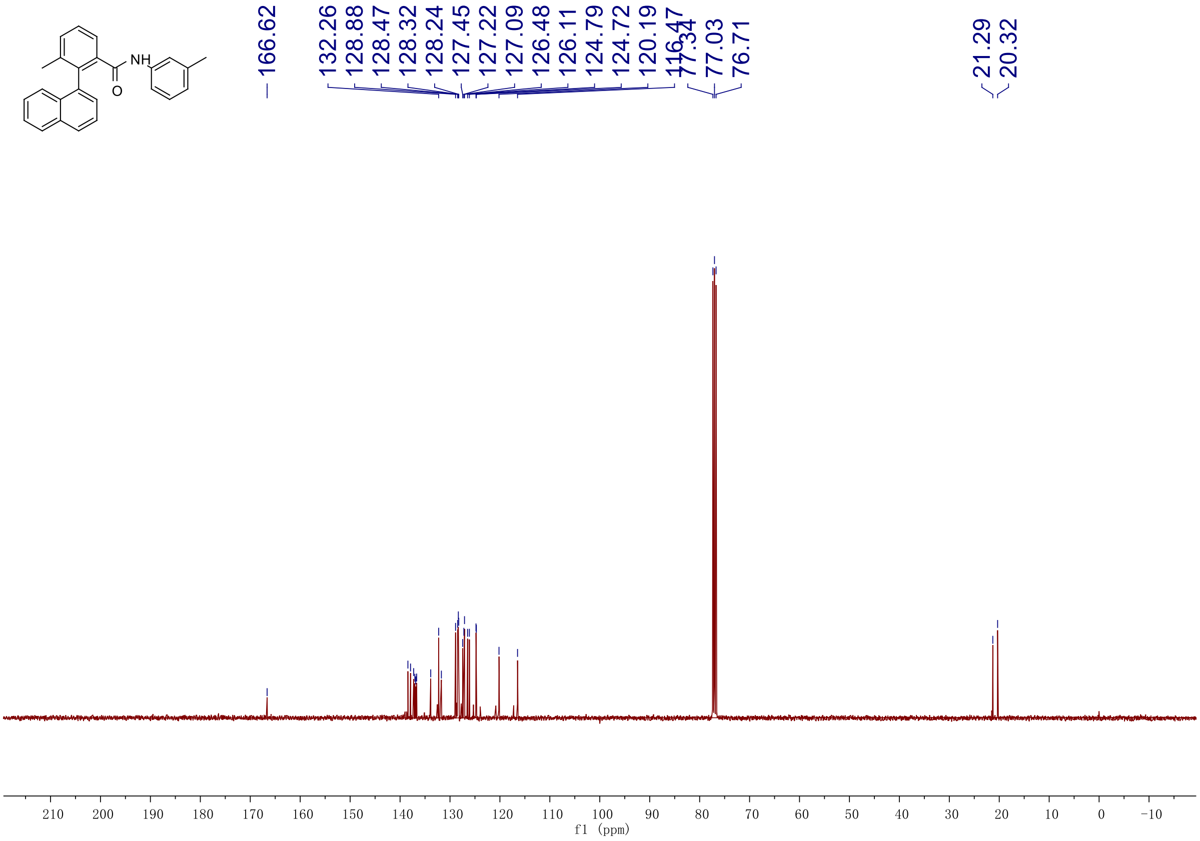

^{13}C NMR Spectrum of 3-methyl-2-(naphthalen-1-yl)-*N*-(*m*-tolyl)benzamide (3i)

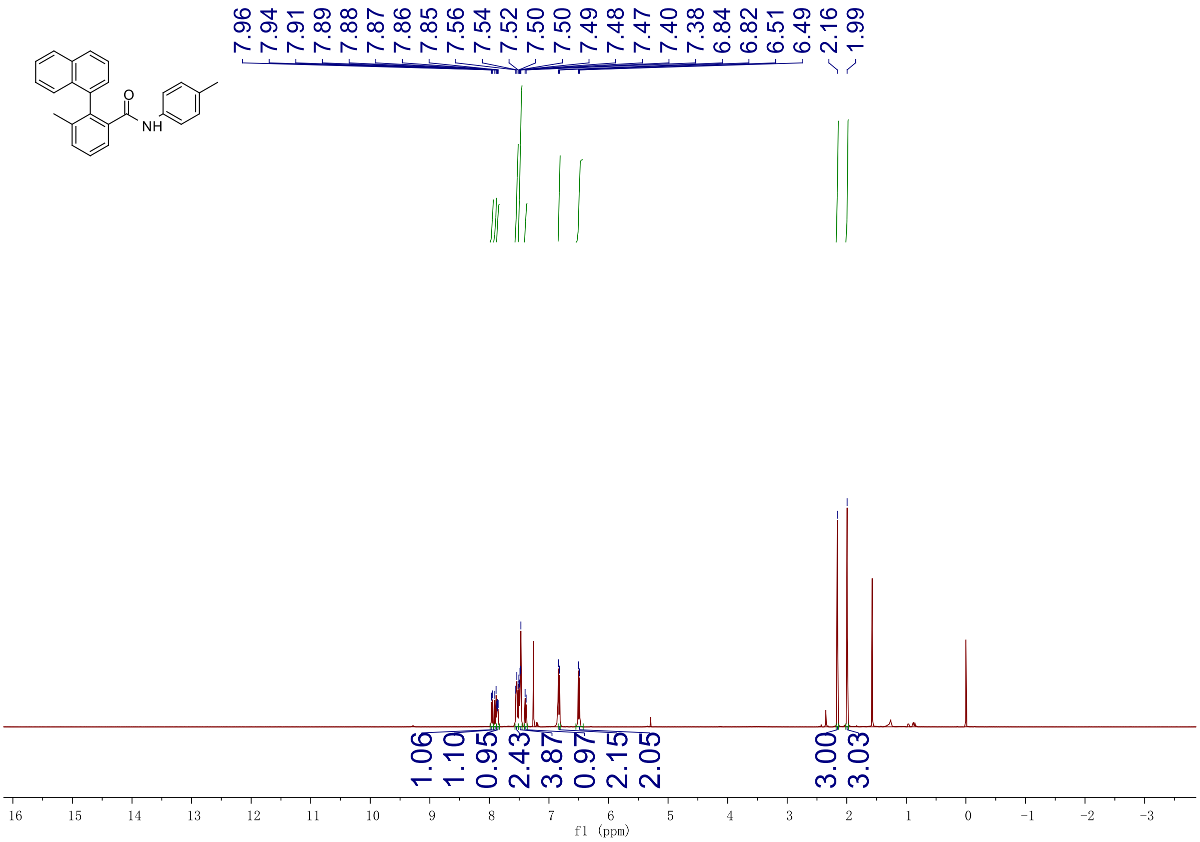

^{1}H NMR Spectrum of 3-methyl-2-(naphthalen-1-yl)-*N*-(*p*-tolyl)benzamide (3j)

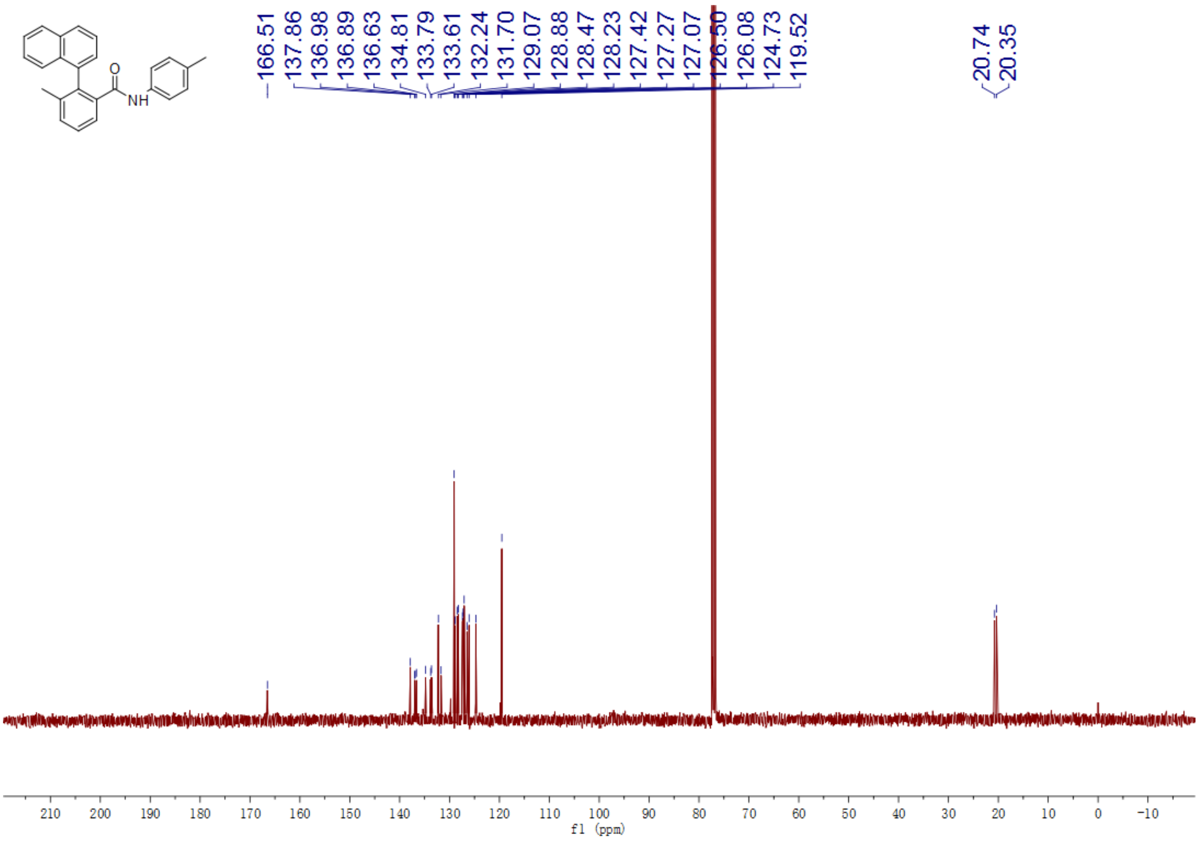

^{13}C NMR Spectrum of 3-methyl-2-(naphthalen-1-yl)-*N*-(*p*-tolyl)benzamide (3j)

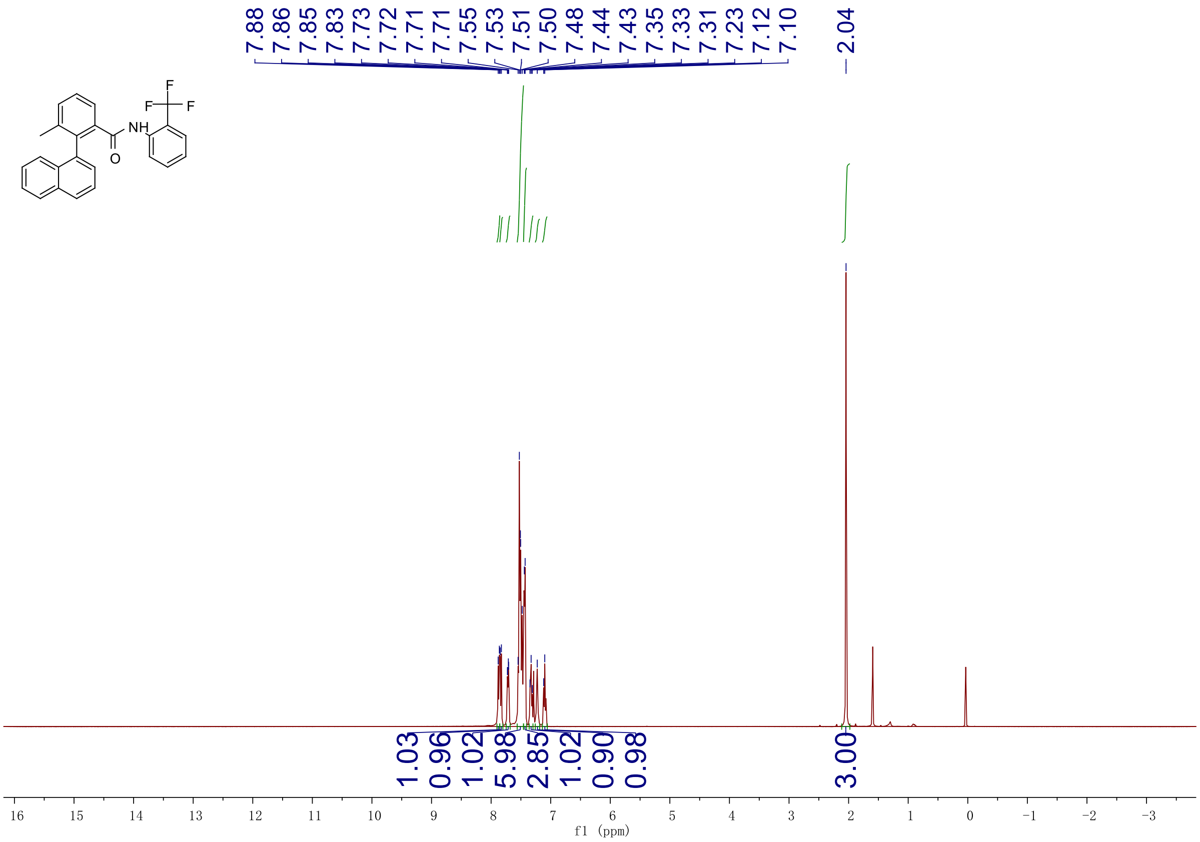

^{1}H NMR Spectrum of 3-methyl-2-(naphthalen-1-yl)-*N*-(2-(trifluoromethyl)phenyl)benzamide (3k)

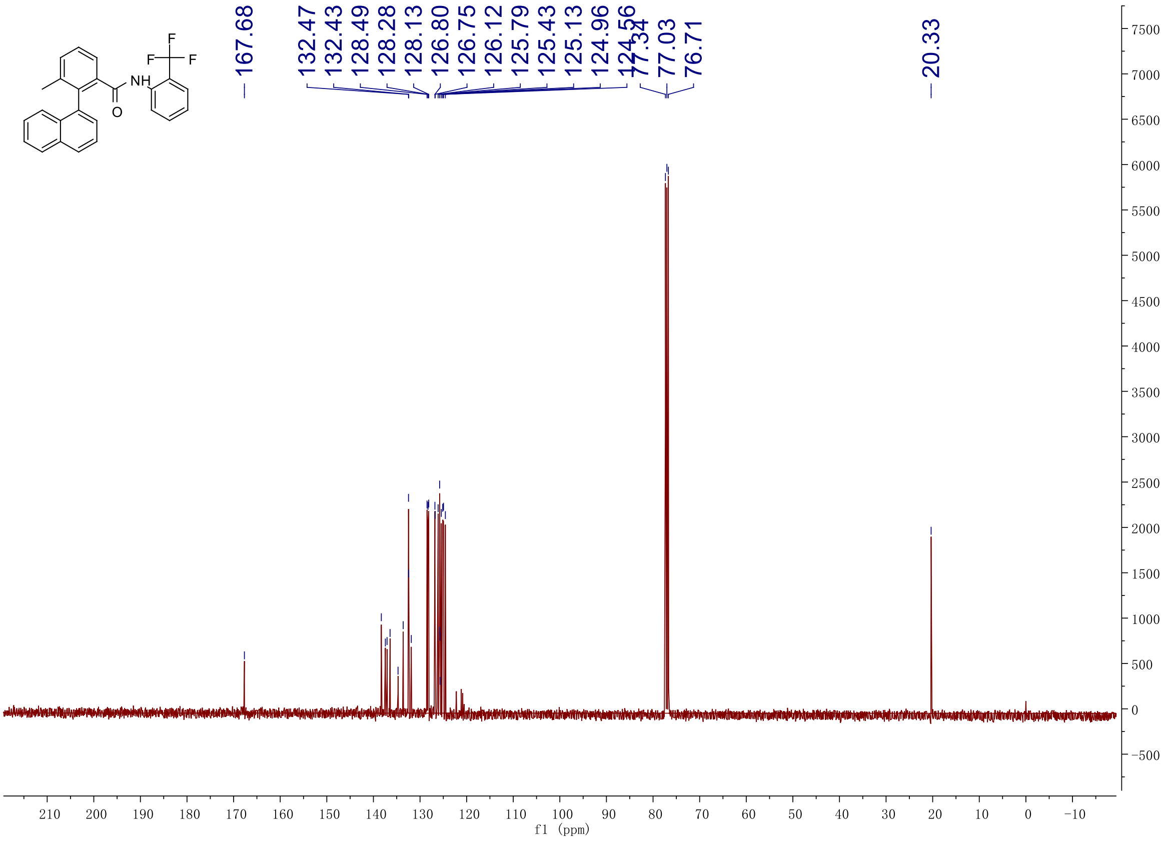

^{13}C NMR Spectrum of 3-methyl-2-(naphthalen-1-yl)-*N*-(2-(trifluoromethyl)phenyl)benzamide (3k)

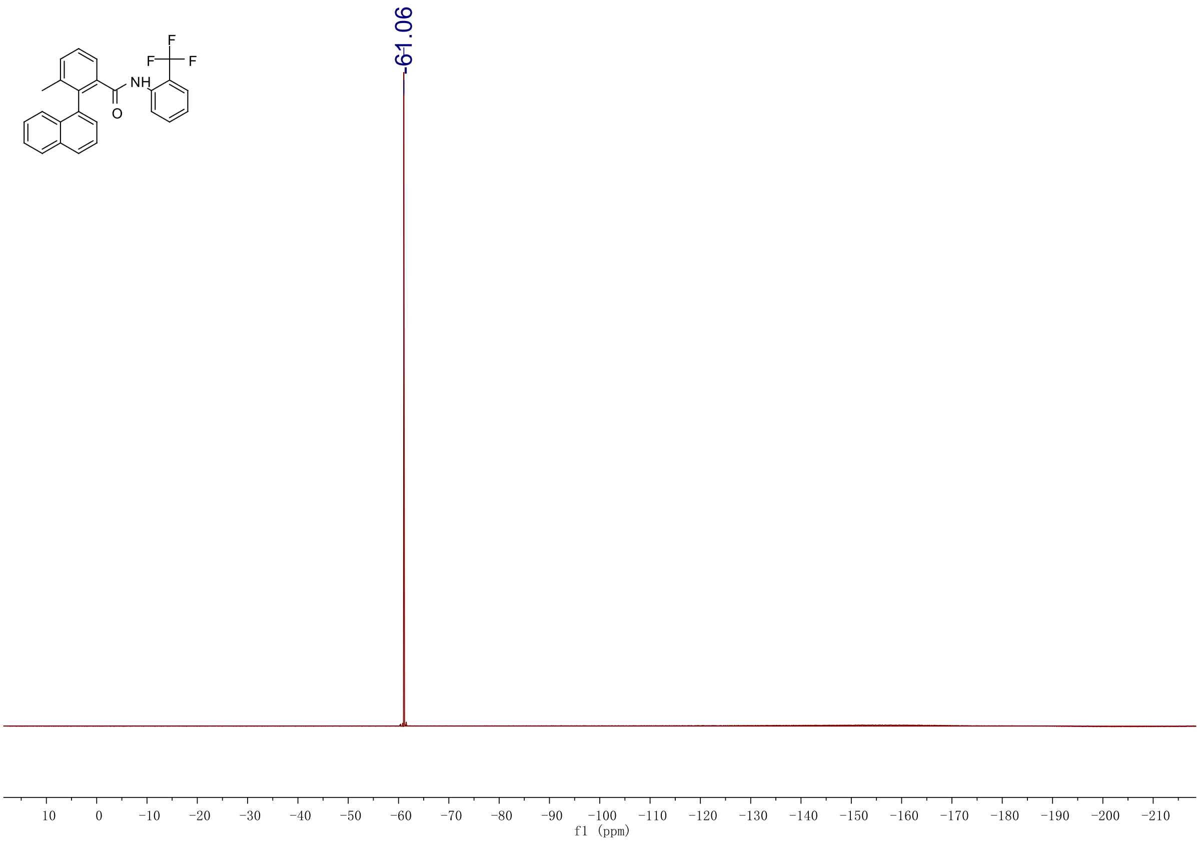

^{19}F NMR Spectrum of 3-methyl-2-(naphthalen-1-yl)-*N*-(2-(trifluoromethyl)phenyl)benzamide (3k)

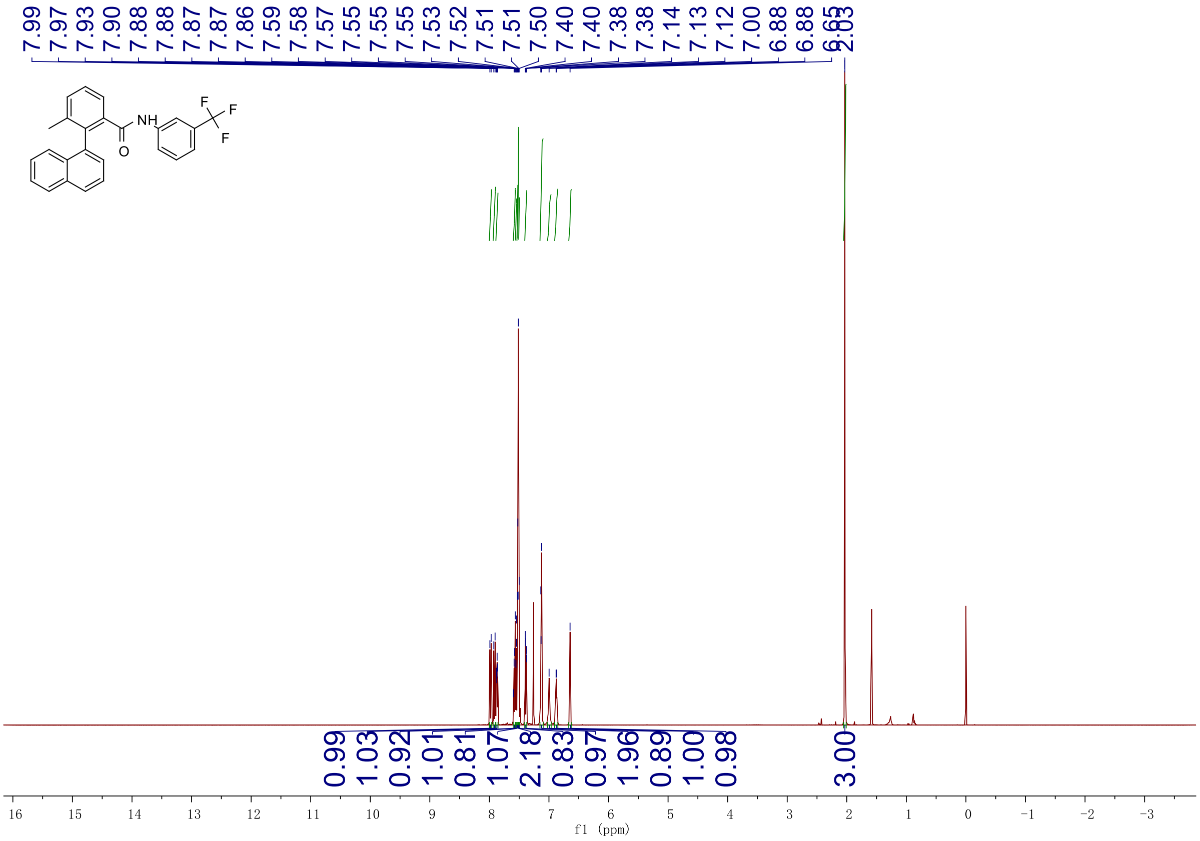

^{1}H NMR Spectrum of 3-methyl-2-(naphthalen-1-yl)-*N*-(3-(trifluoromethyl)phenyl)benzamide (3l)

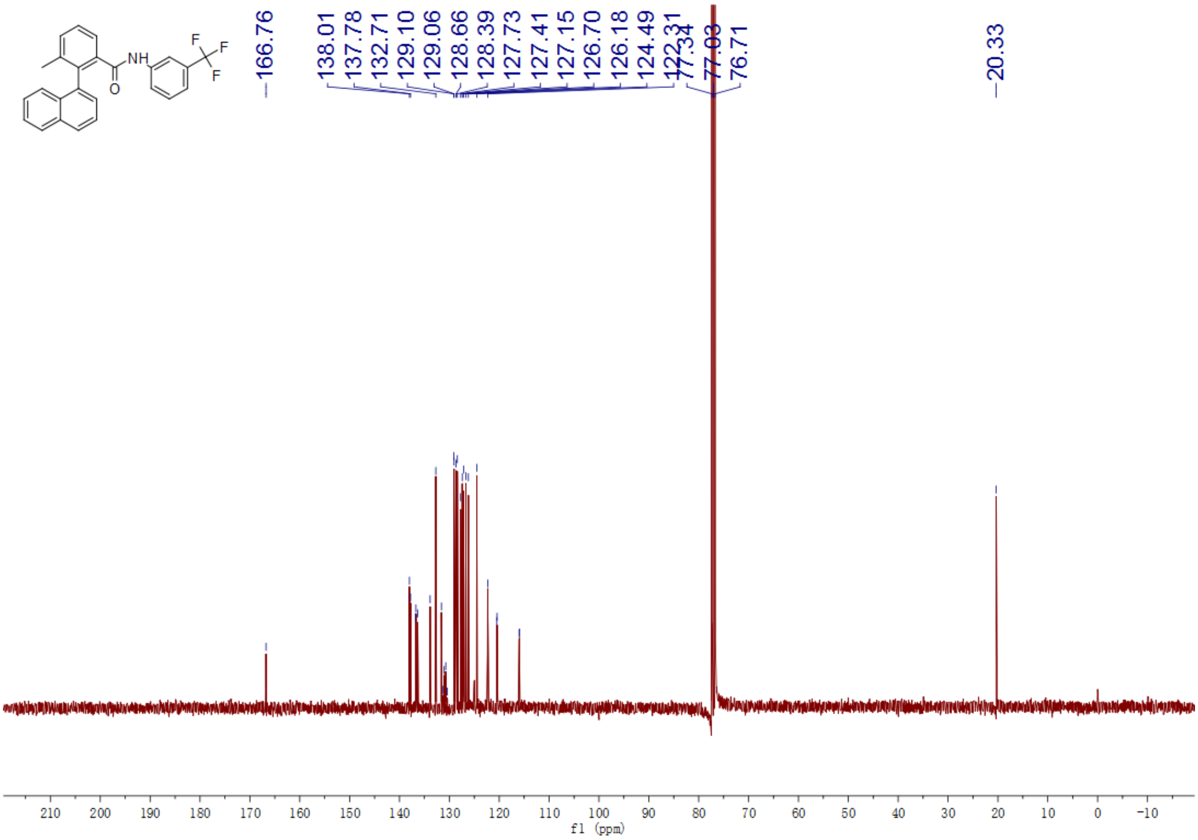

^{13}C NMR Spectrum of 3-methyl-2-(naphthalen-1-yl)-*N*-(3-(trifluoromethyl)phenyl)benzamide (3l)

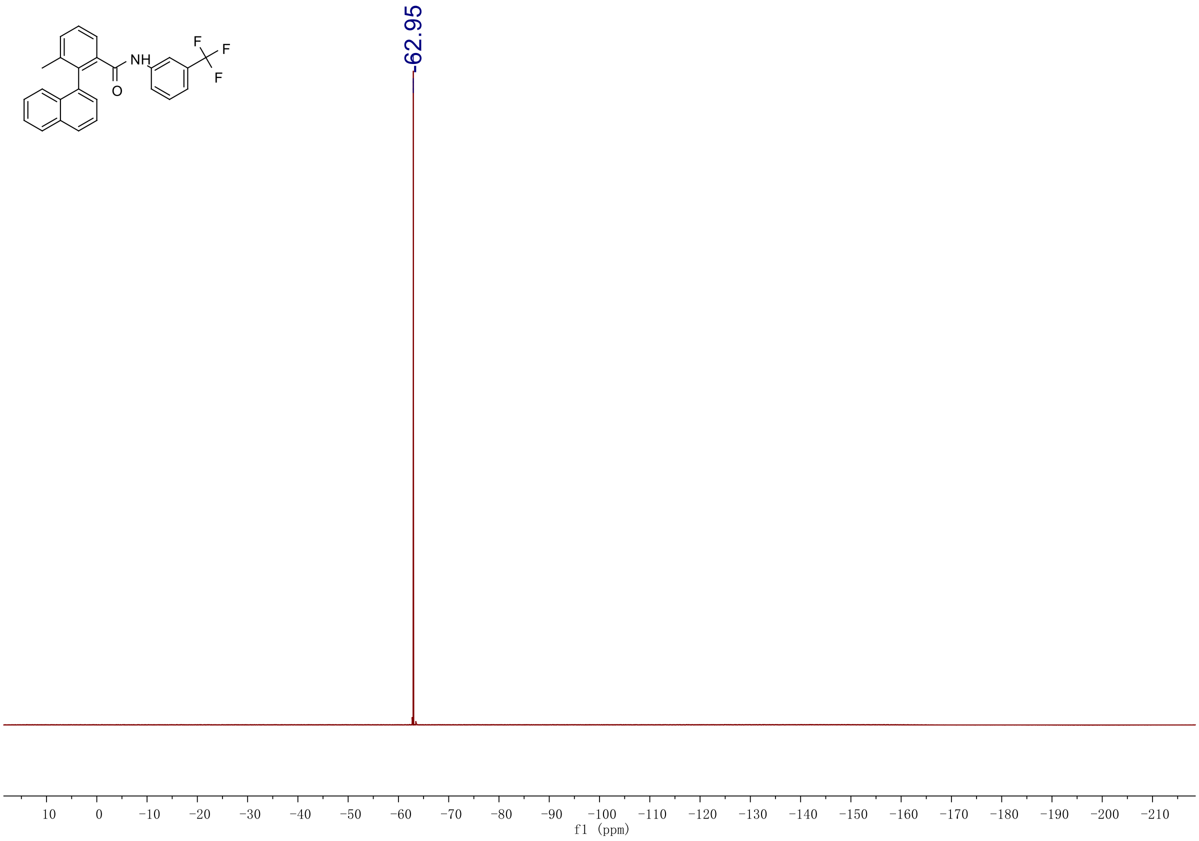

^{19}F NMR Spectrum of 3-methyl-2-(naphthalen-1-yl)-*N*-(3-(trifluoromethyl)phenyl)benzamide (3l)

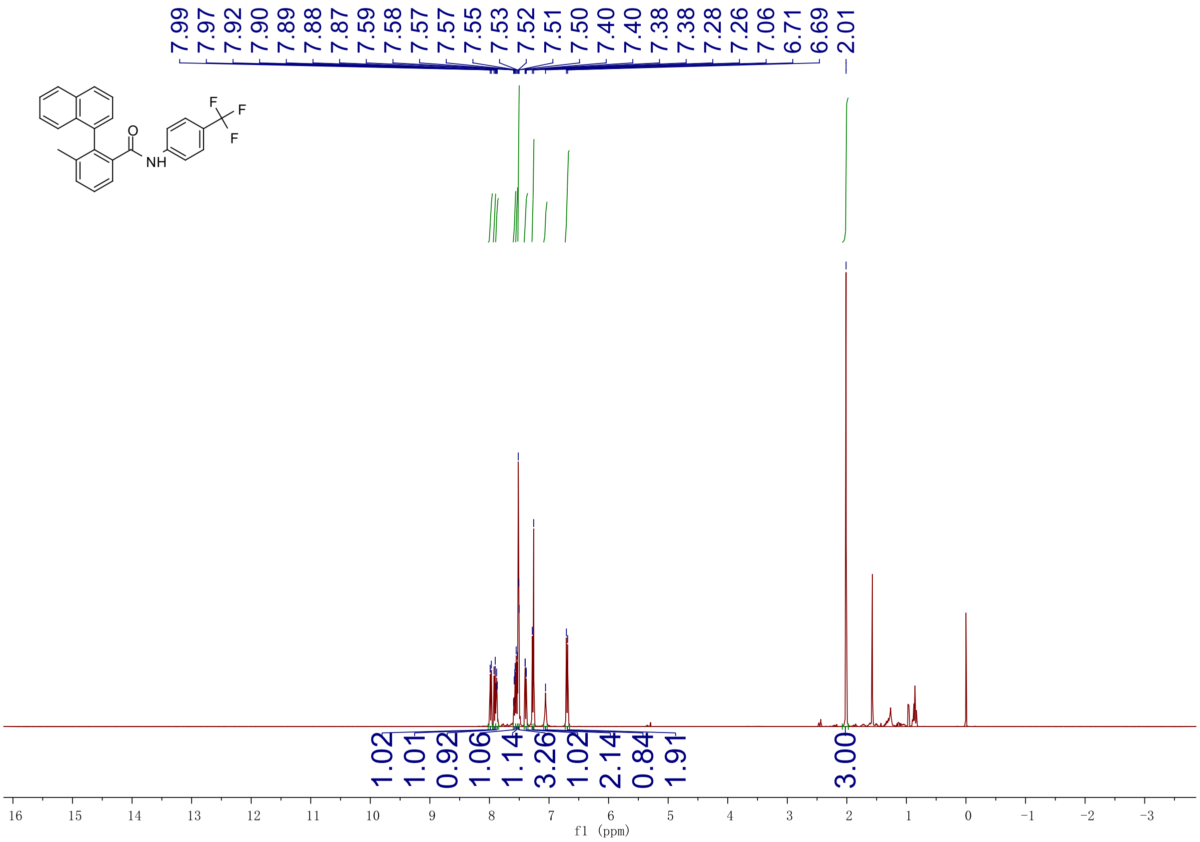

^{1}H NMR Spectrum of 3-methyl-2-(naphthalen-1-yl)-*N*-(4-(trifluoromethyl)phenyl)benzamide (3m)

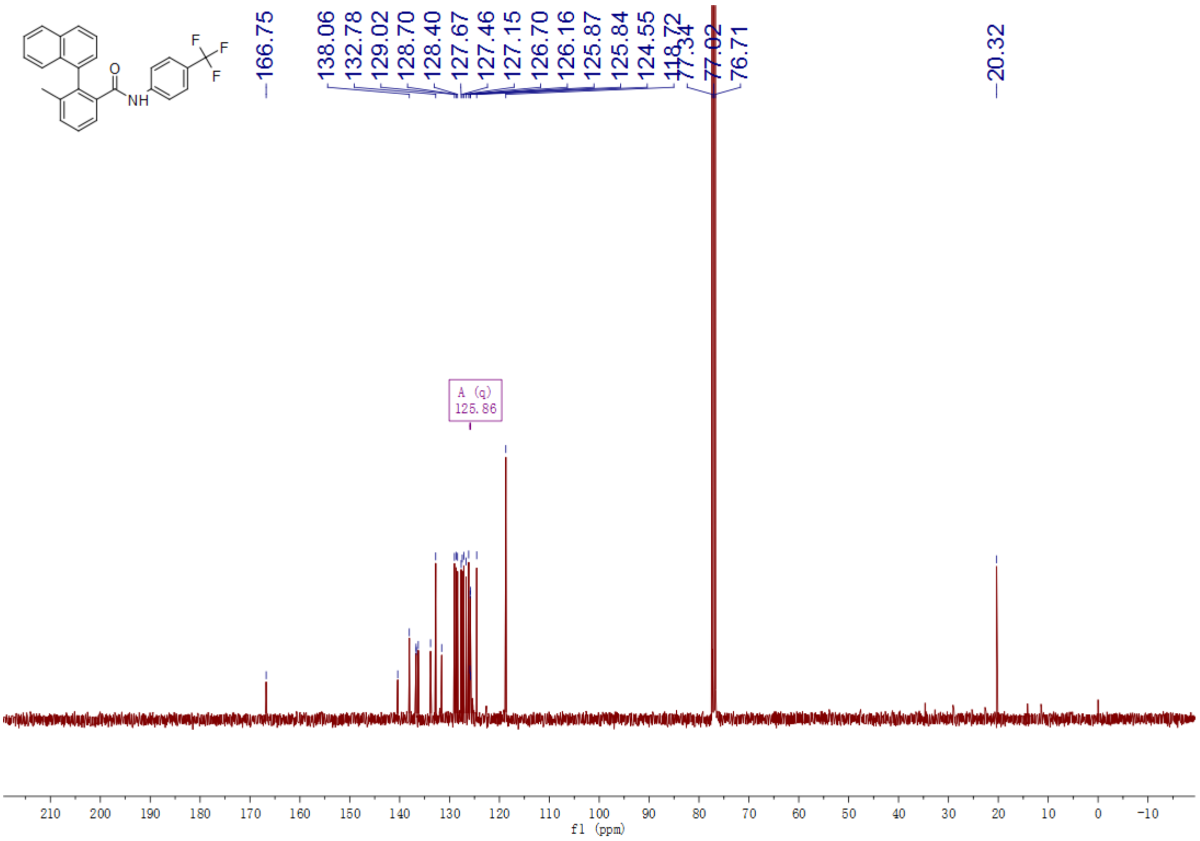

^{13}C NMR Spectrum of 3-methyl-2-(naphthalen-1-yl)-*N*-(4-(trifluoromethyl)phenyl)benzamide (3m)

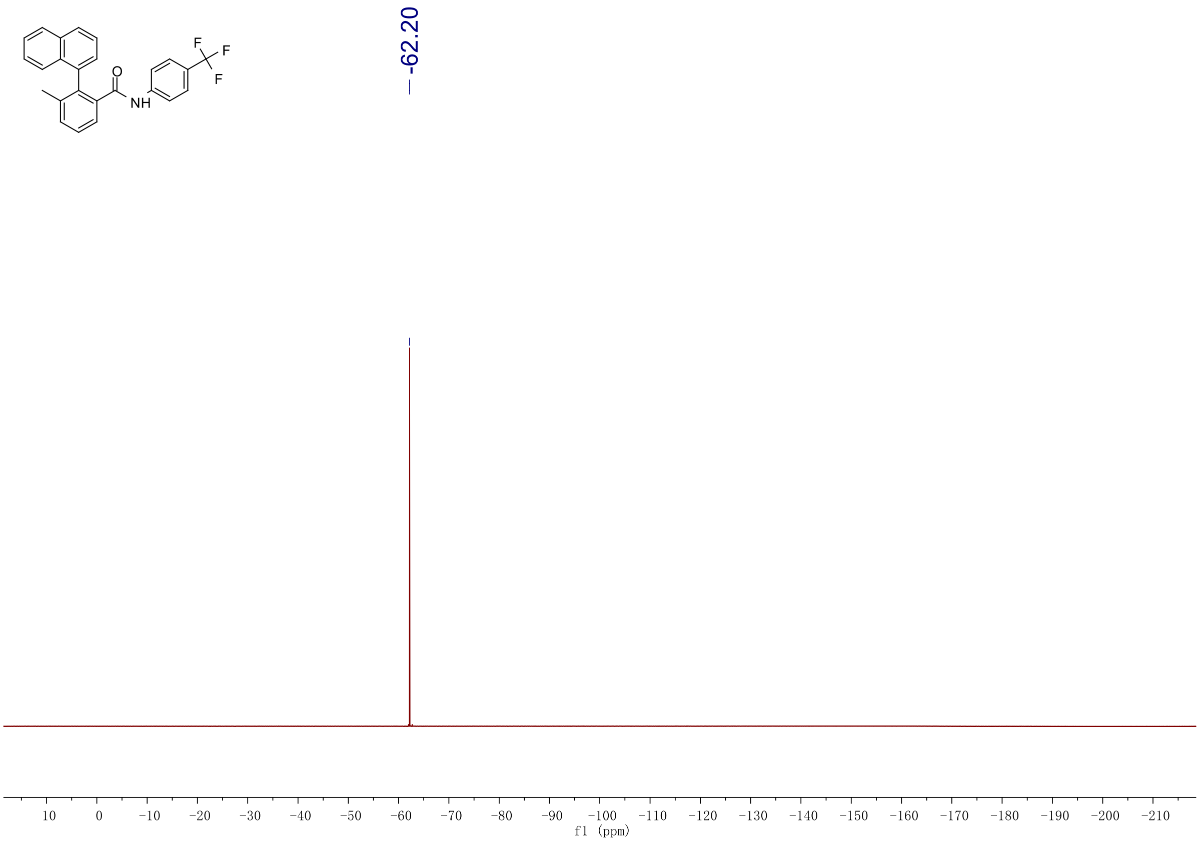

^{19}F NMR Spectrum of 3-methyl-2-(naphthalen-1-yl)-*N*-(4-(trifluoromethyl)phenyl)benzamide (3m)

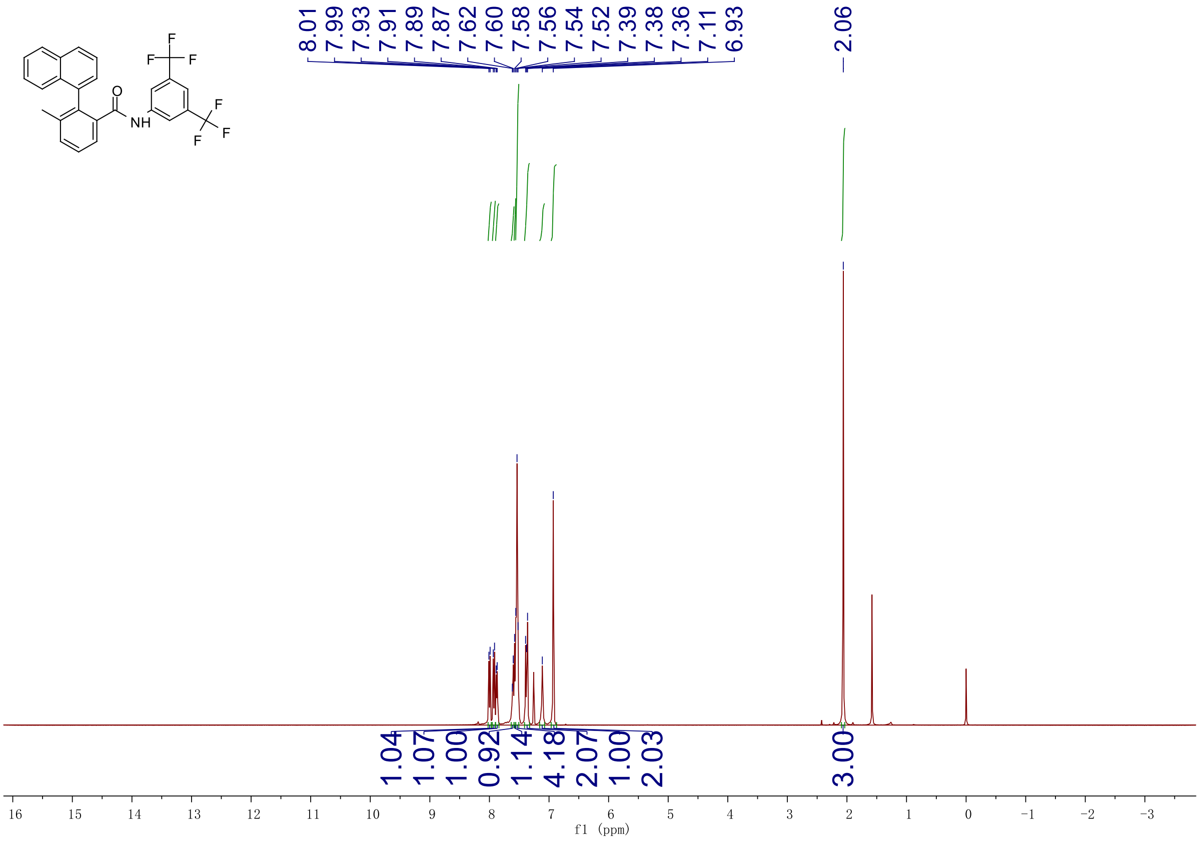

^{1}H NMR Spectrum of *N*-(3,5-bis(trifluoromethyl)phenyl)-3-methyl-2-(naphthalen-1-yl)benzamide (3n)

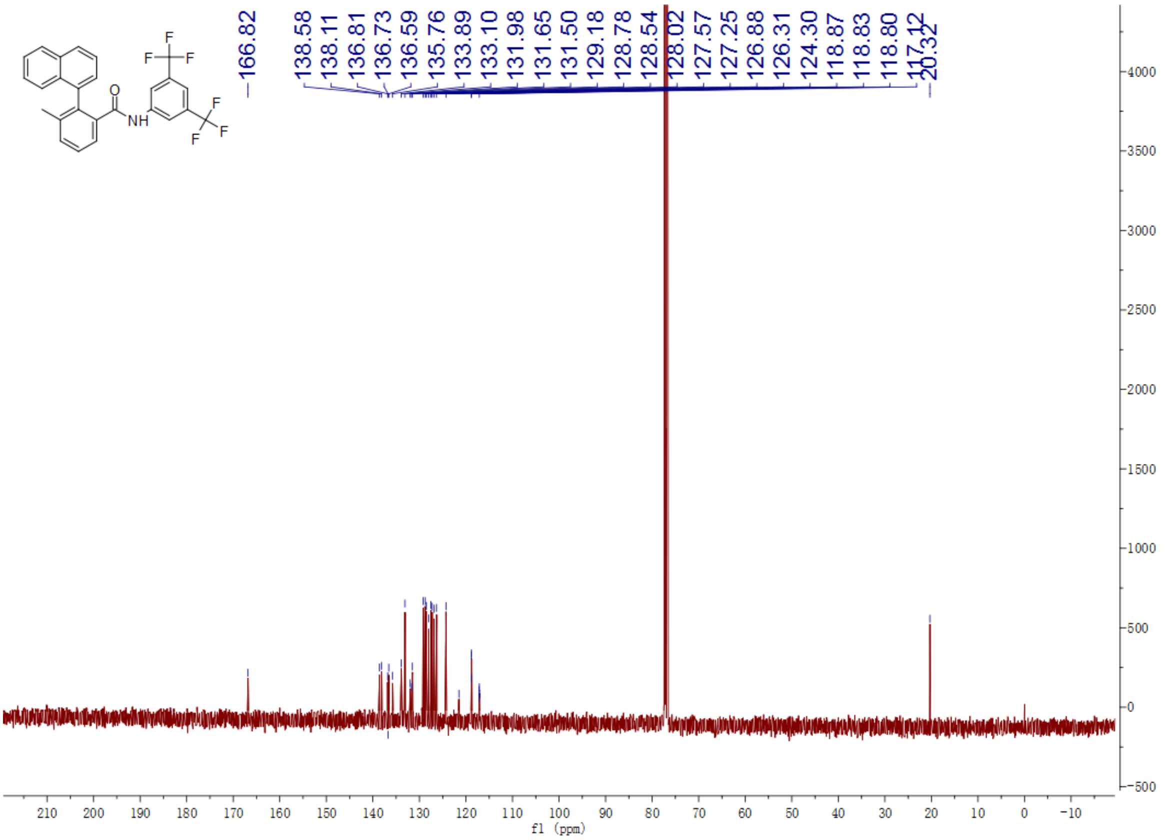

^{13}C NMR Spectrum of *N*-(3,5-bis(trifluoromethyl)phenyl)-3-methyl-2-(naphthalen-1-yl)benzamide (3n)

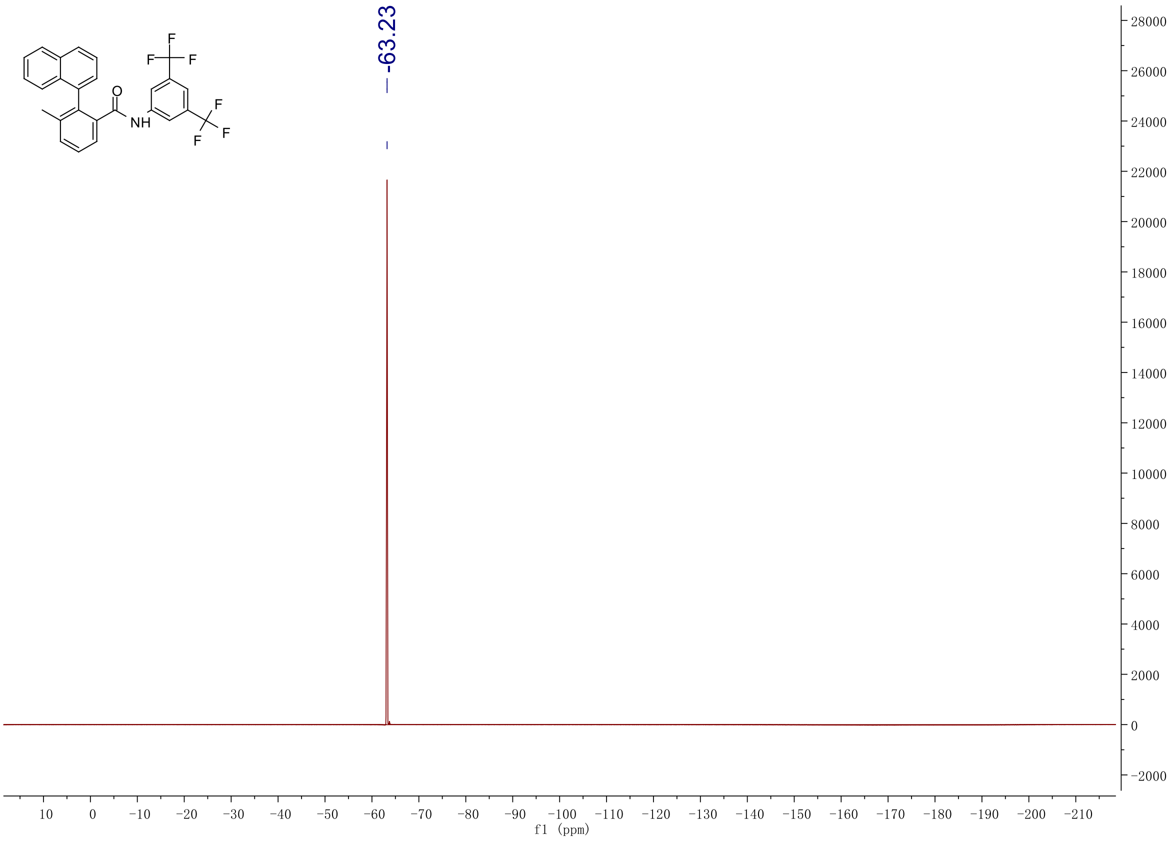

^{19}F NMR Spectrum of *N*-(3,5-bis(trifluoromethyl)phenyl)-3-methyl-2-(naphthalen-1-yl)benzamide (3n)

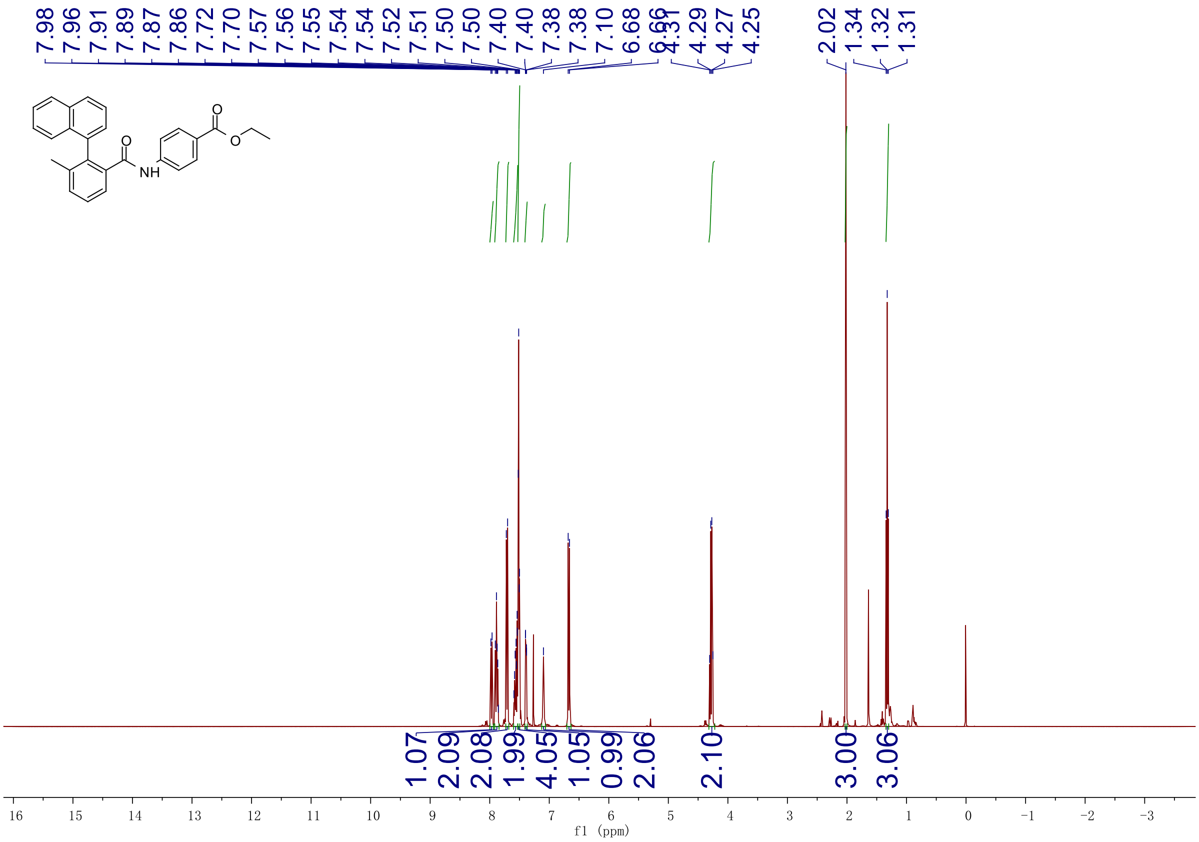

^{1}H NMR Spectrum of ethyl 4-(3-methyl-2-(naphthalen-1-yl)benzamido)benzoate (3o)

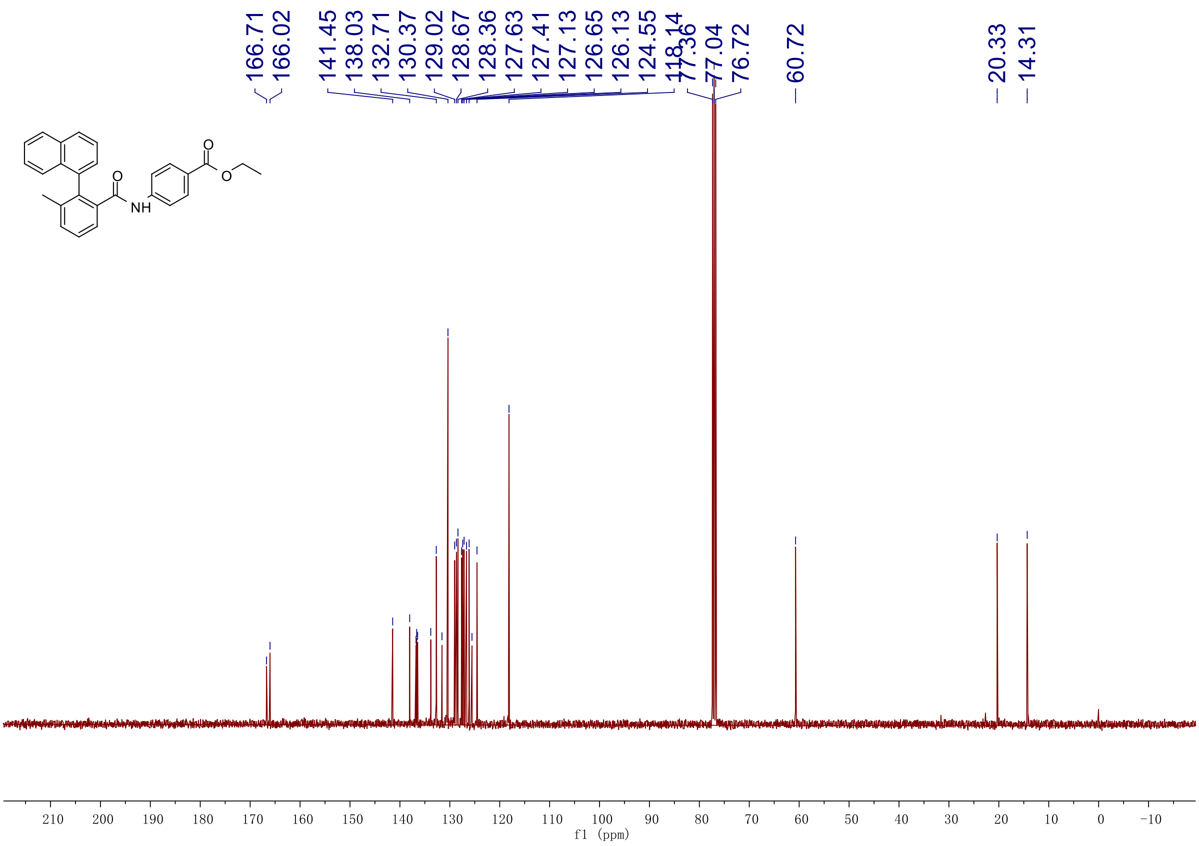

^{13}C NMR Spectrum of ethyl 4-(3-methyl-2-(naphthalen-1-yl)benzamido)benzoate (3o)

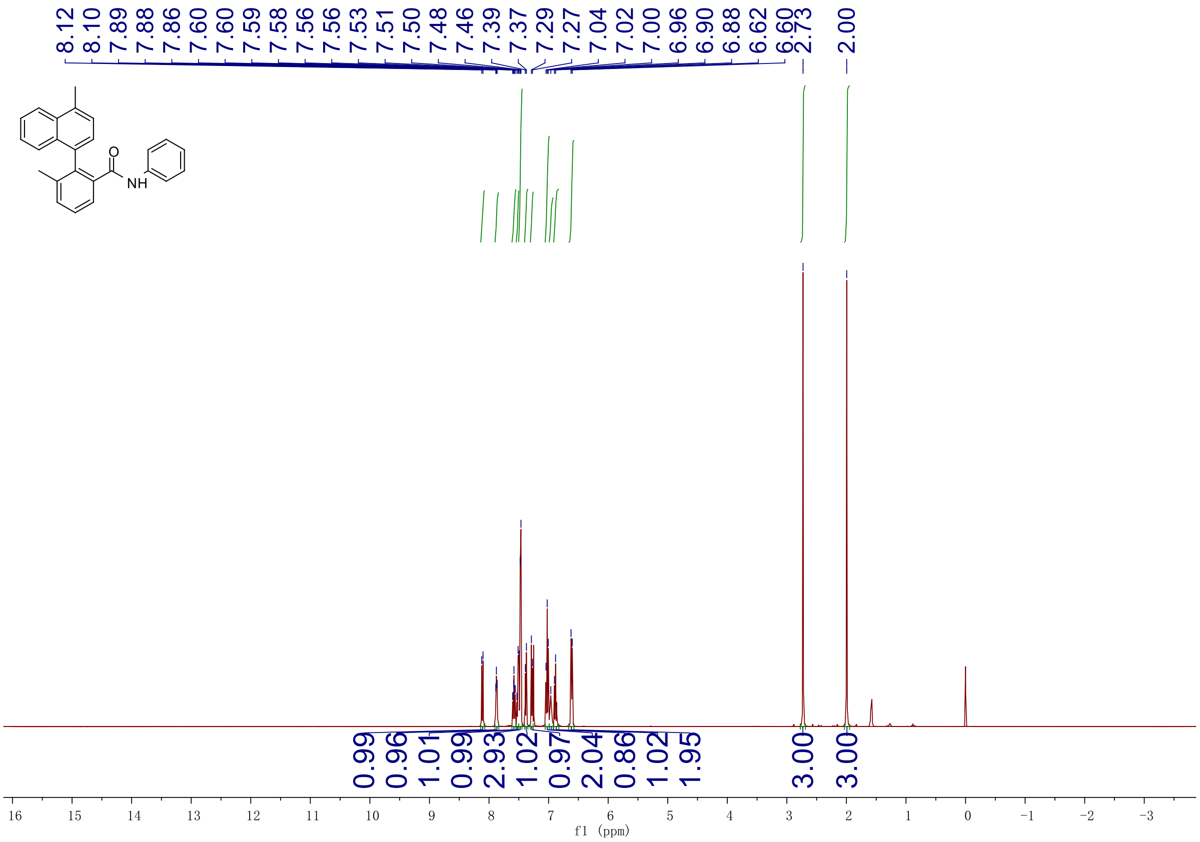

^{1}H NMR Spectrum of 3-methyl-2-(4-methylnaphthalen-1-yl)-*N*-phenylbenzamide (3p)

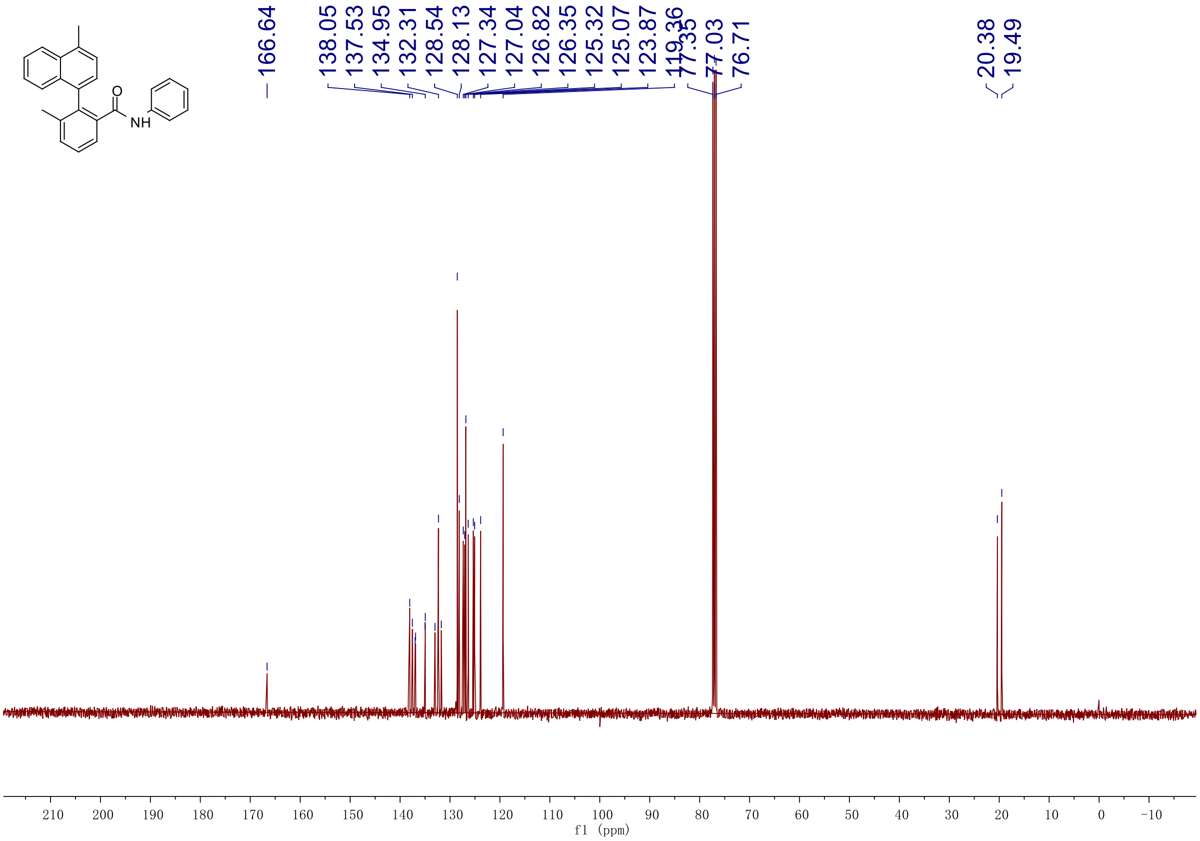

^{13}C NMR Spectrum of 3-methyl-2-(4-methylnaphthalen-1-yl)-*N*-phenylbenzamide (3p)

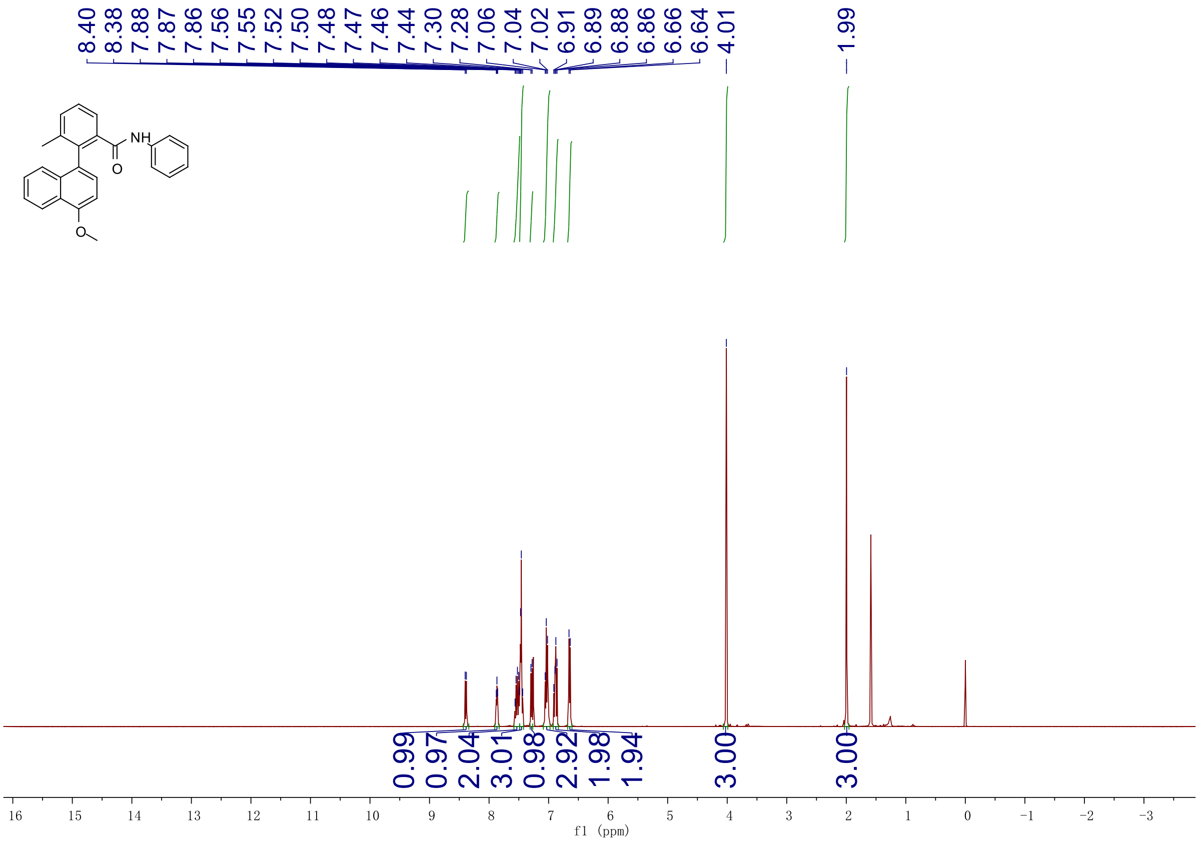

^{1}H NMR Spectrum of 4-methoxy-*N*-phenyl-1-(*o*-tolyl)-2-naphthamide (3q)

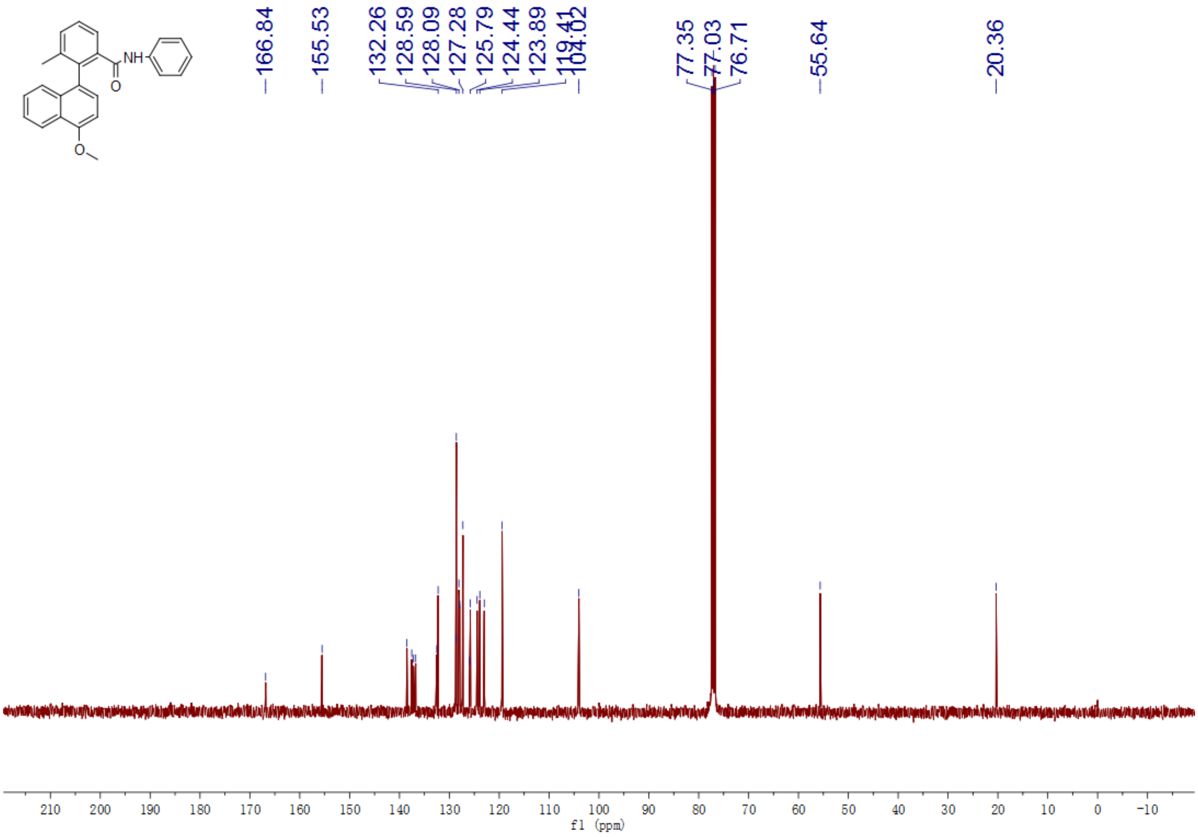

^{13}C NMR Spectrum of 4-methoxy-*N*-phenyl-1-(*o*-tolyl)-2-naphthamide (3q)

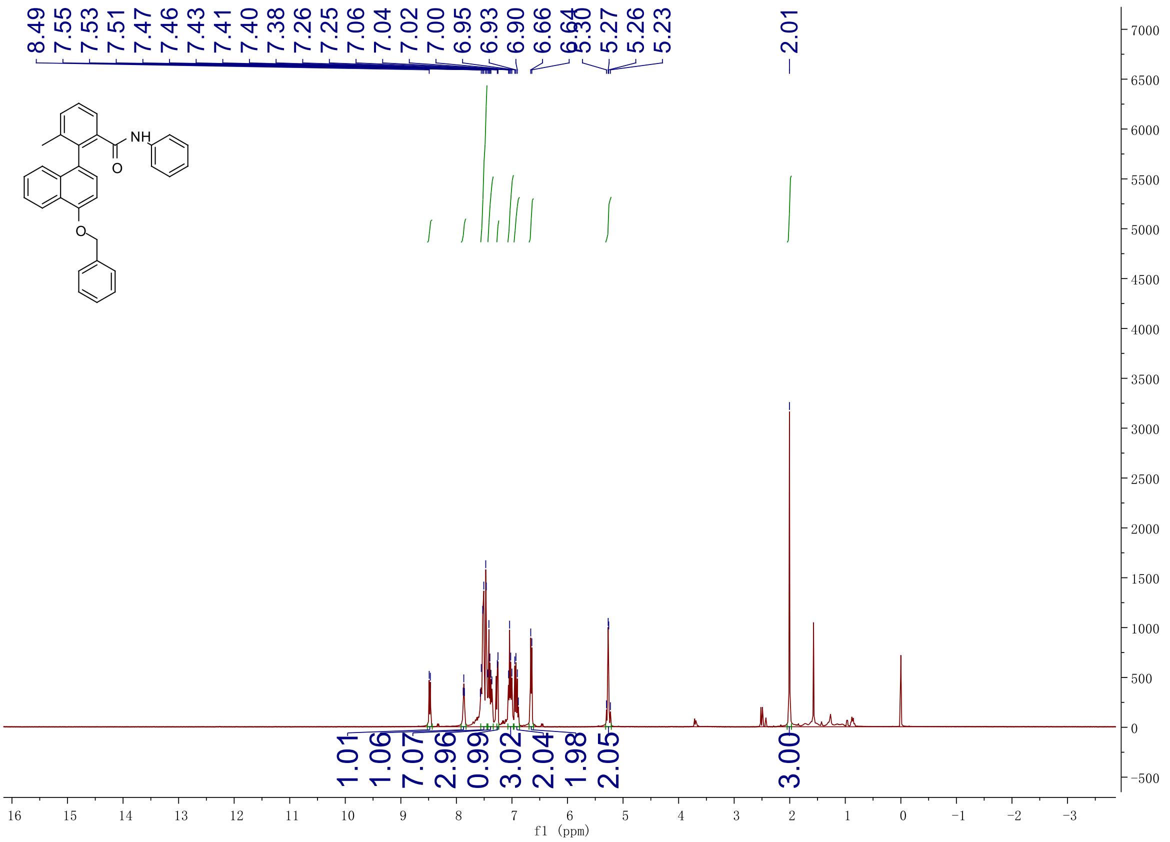

^{1}H NMR Spectrum of 2-(4-(benzyloxy)naphthalen-1-yl)-3-methyl-*N*-phenylbenzamide (3r)

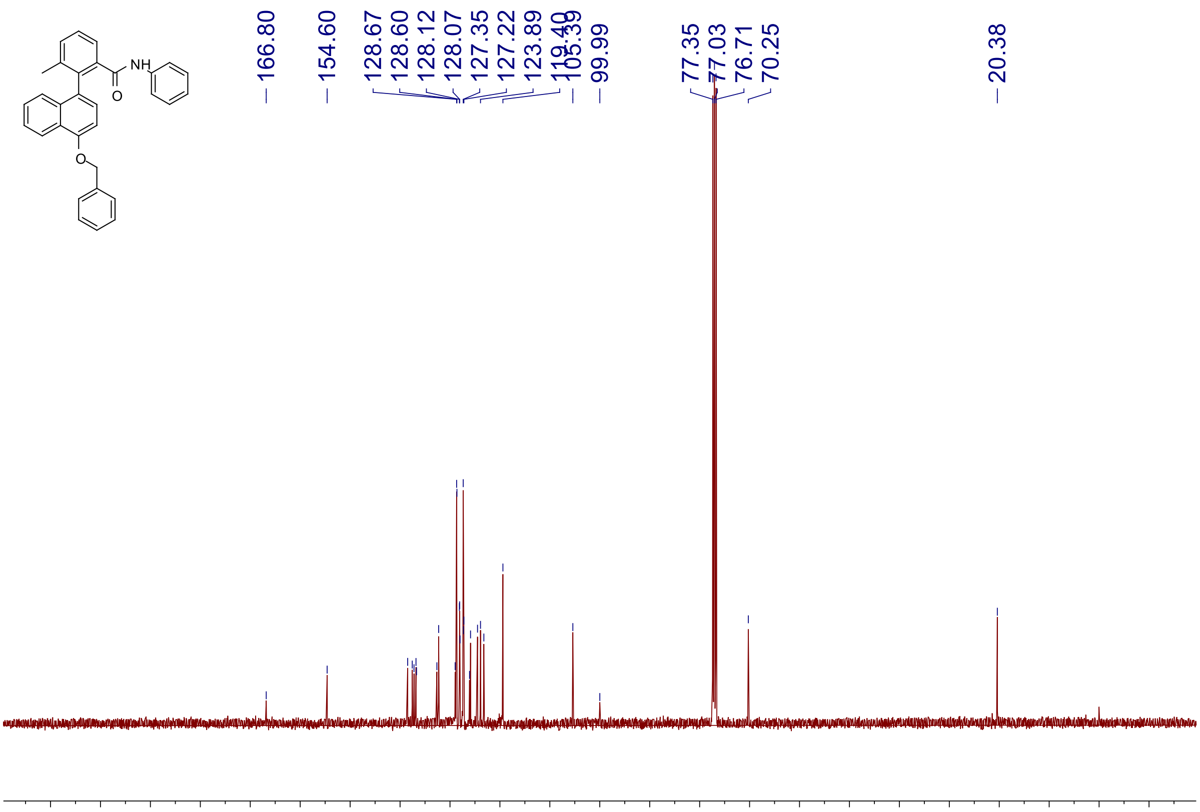

^{13}C NMR Spectrum of 2-(4-(benzyloxy)naphthalen-1-yl)-3-methyl-*N*-phenylbenzamide (3r)

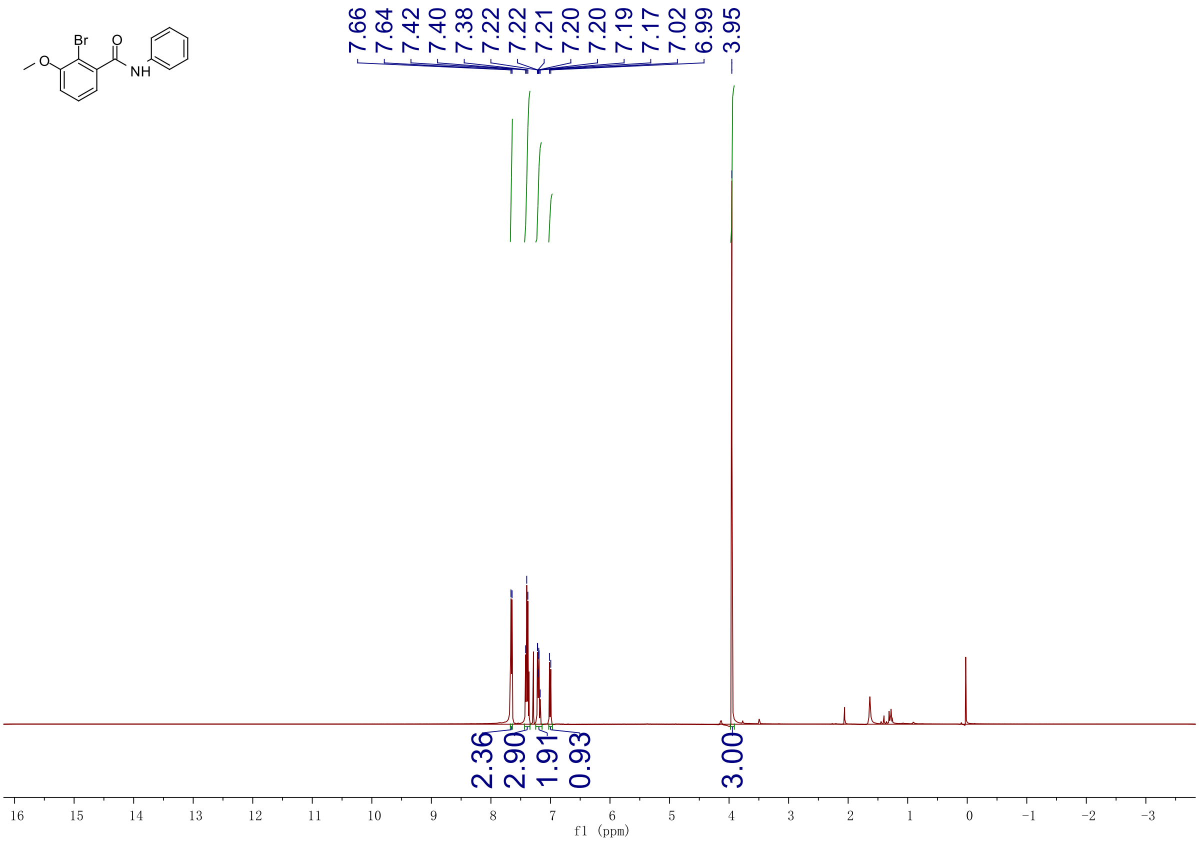

^{1}H NMR Spectrum of 2-bromo-3-methoxy-*N*-phenylbenzamide (4i)

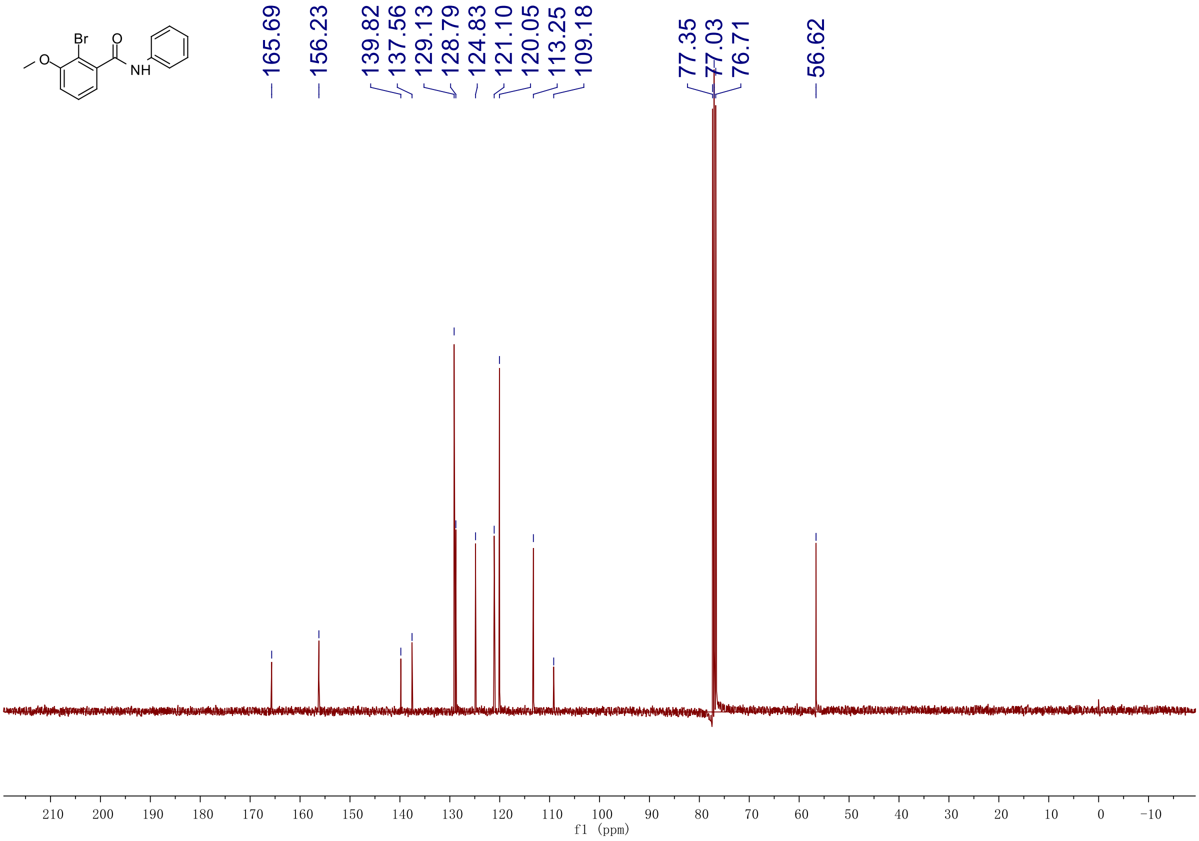

^{13}C NMR Spectrum of 2-bromo-3-methoxy-*N*-phenylbenzamide (4i)

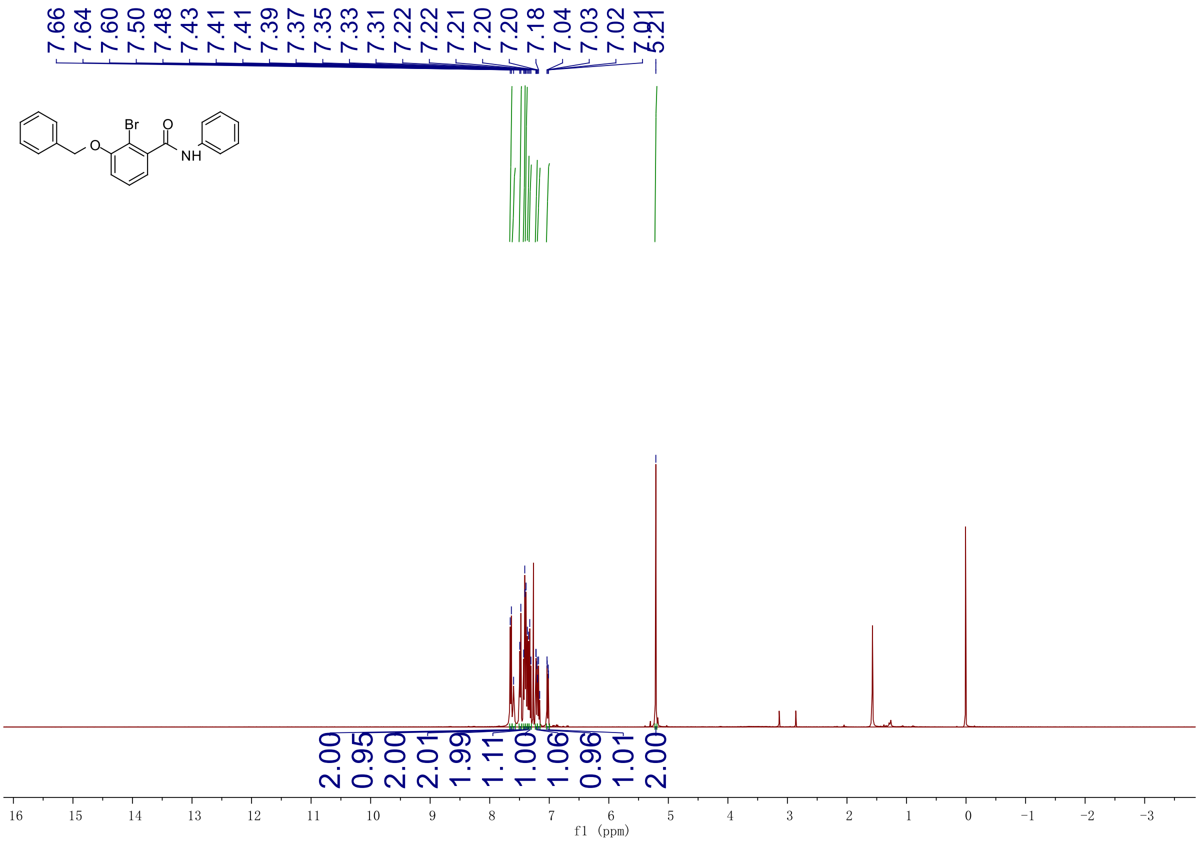

^{1}H NMR Spectrum of 3-(benzyloxy)-2-bromo-*N*-phenylbenzamide (4j)

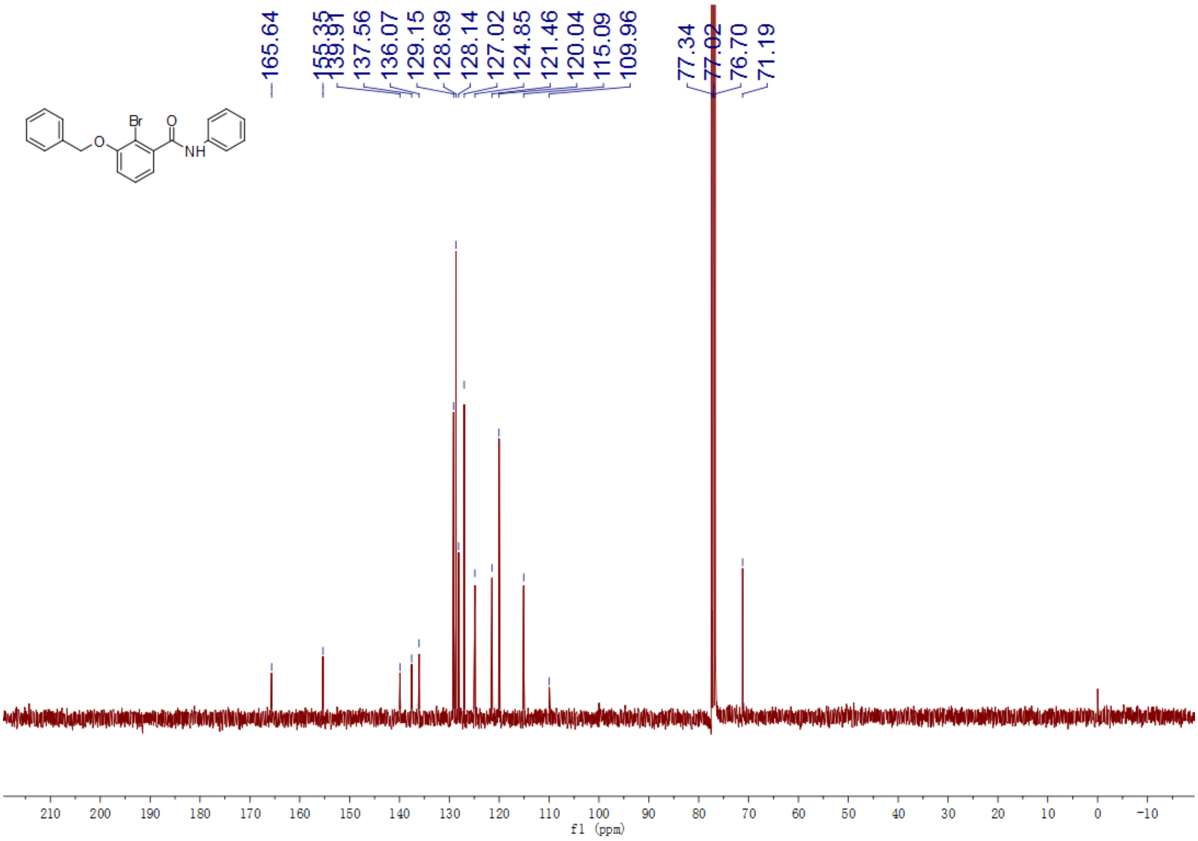

^{13}C NMR Spectrum of 3-(benzyloxy)-2-bromo-*N*-phenylbenzamide (4j)

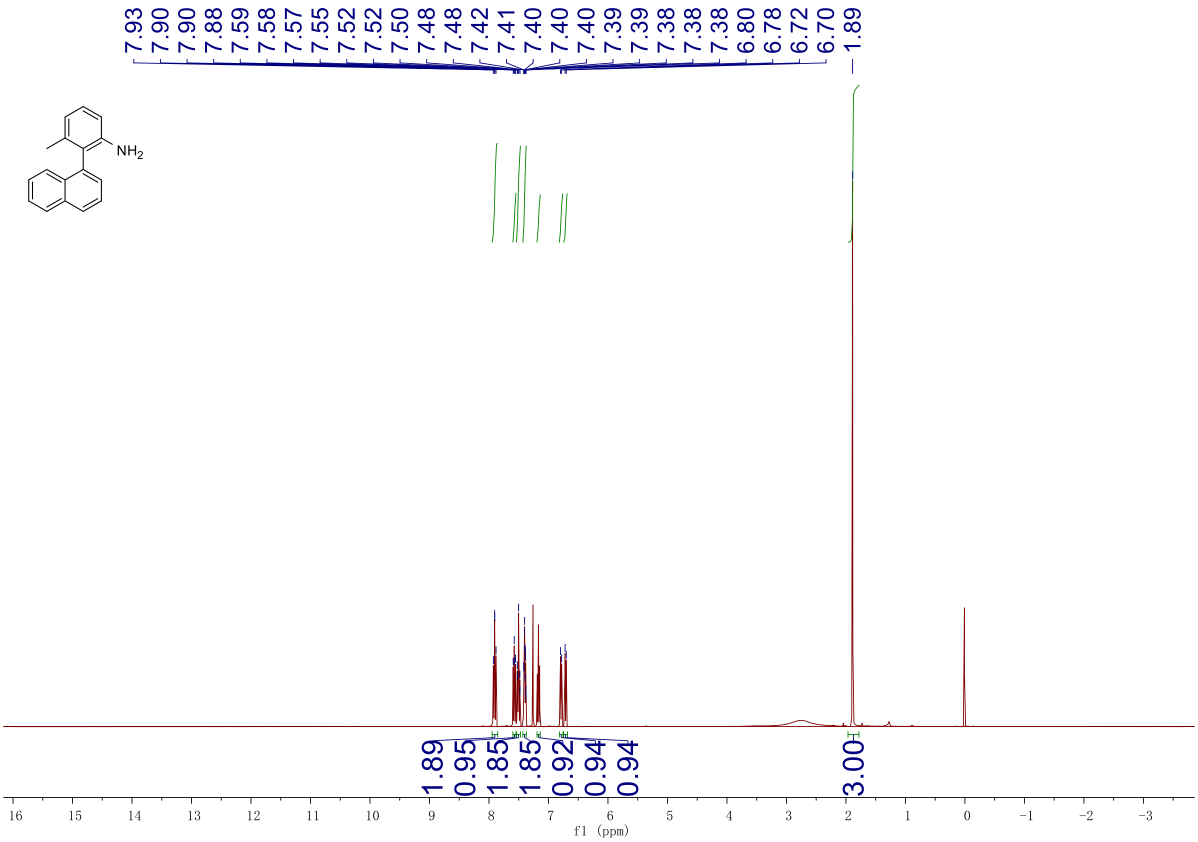

^{1}H NMR Spectrum of 3-methyl-2-(naphthalen-1-yl)aniline (5a)

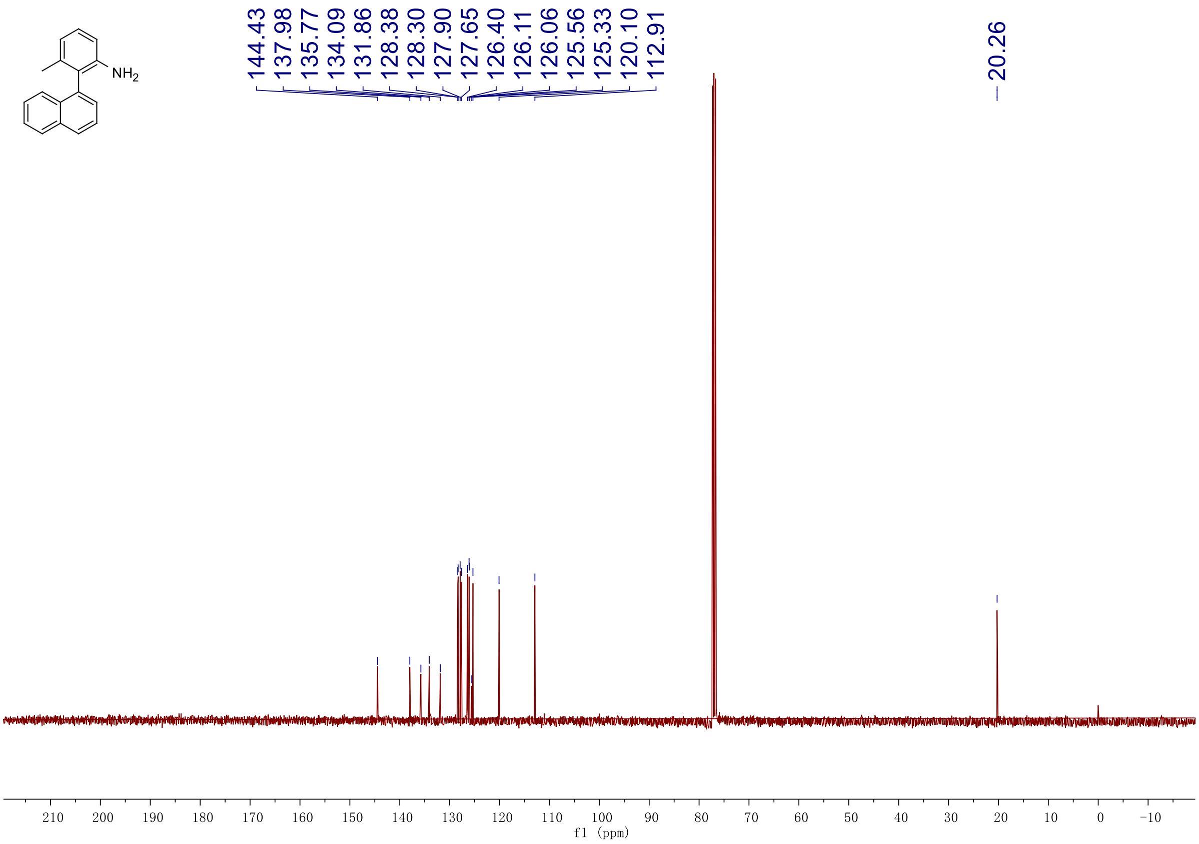

^{13}C NMR Spectrum of 3-methyl-2-(naphthalen-1-yl)aniline (5a)

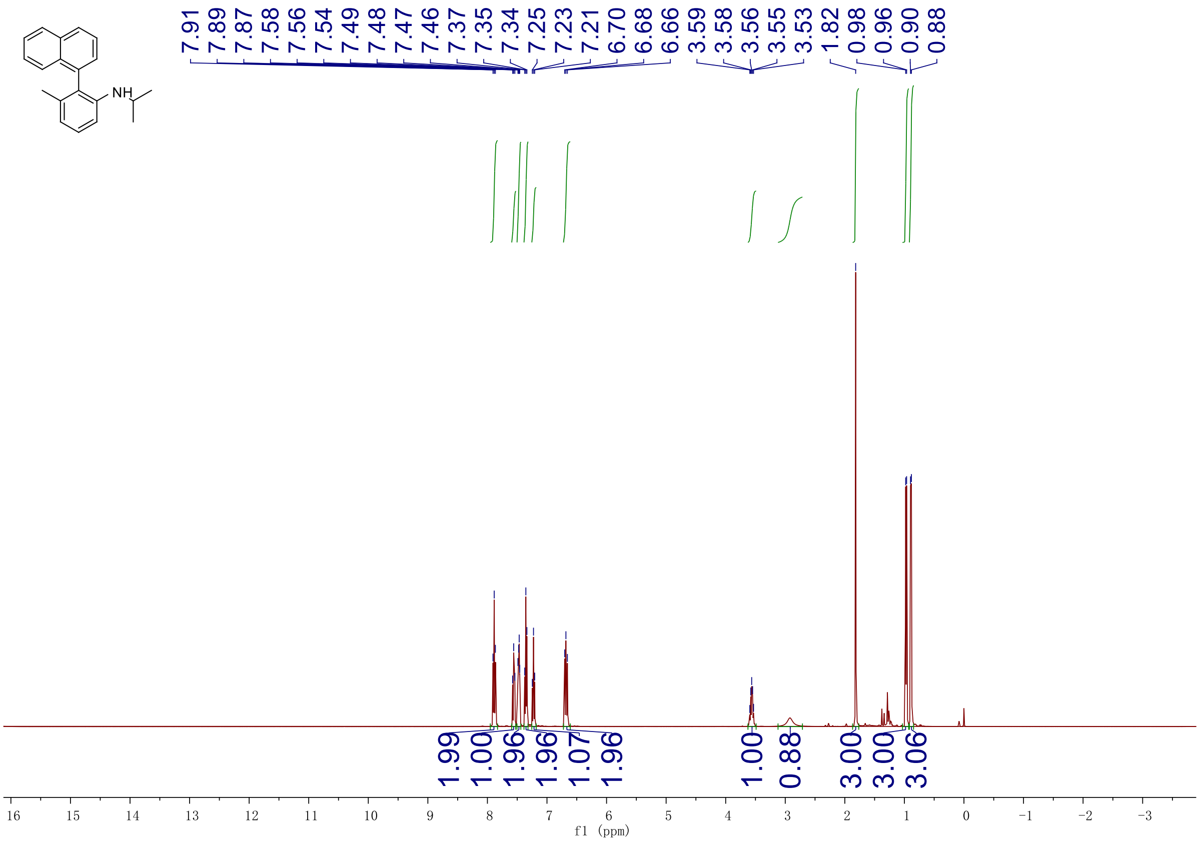

^{1}H NMR Spectrum of *N*-isopropyl-3-methyl-2-(naphthalen-1-yl)aniline (5b)

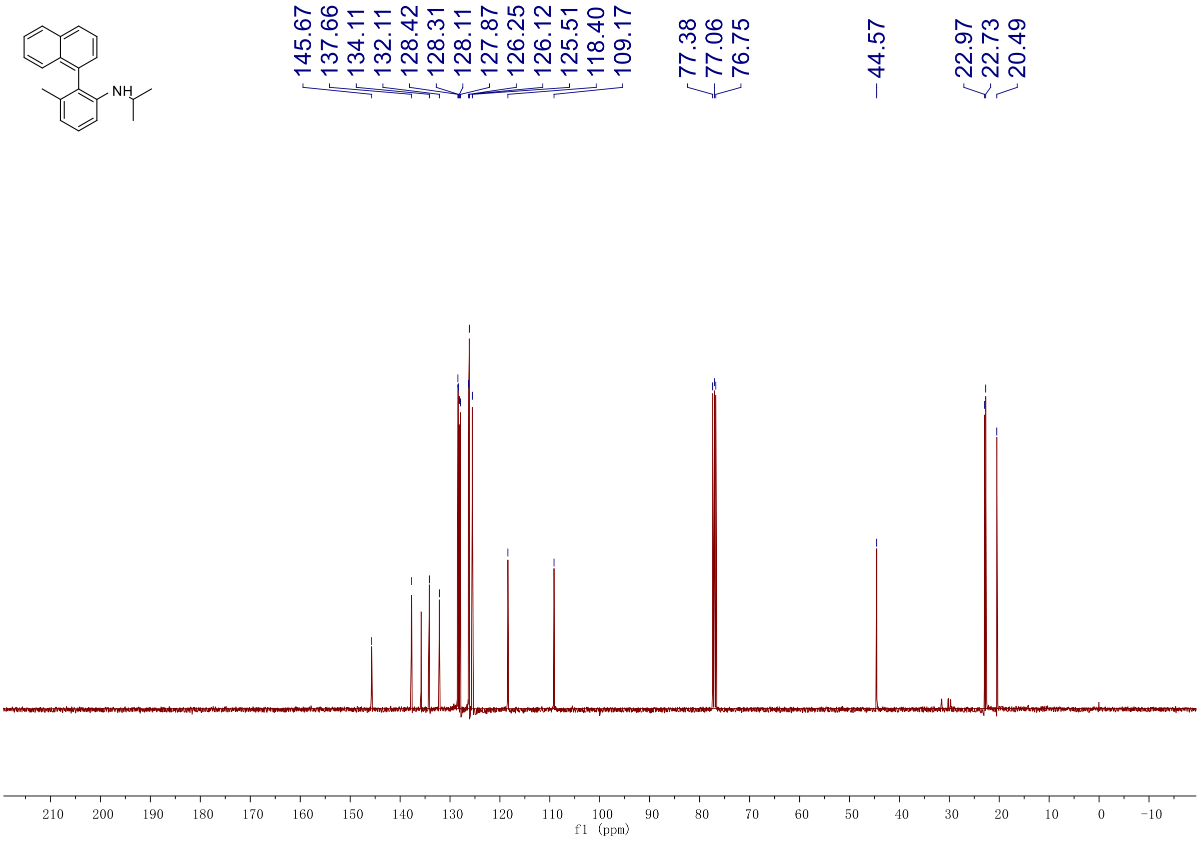

^{13}C NMR Spectrum of *N*-isopropyl-3-methyl-2-(naphthalen-1-yl)aniline (5b)

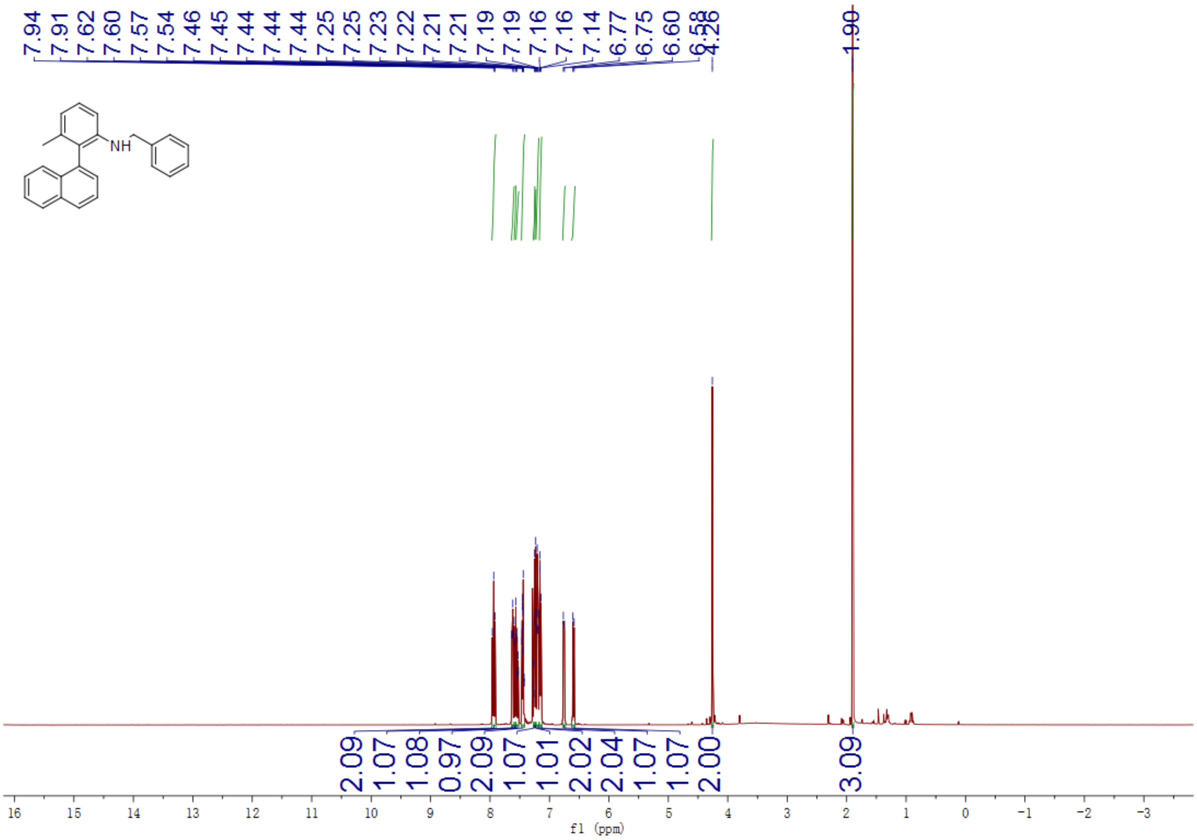

^{1}H NMR Spectrum of *N*-benzyl-3-methyl-2-(naphthalen-1-yl)aniline (5c)

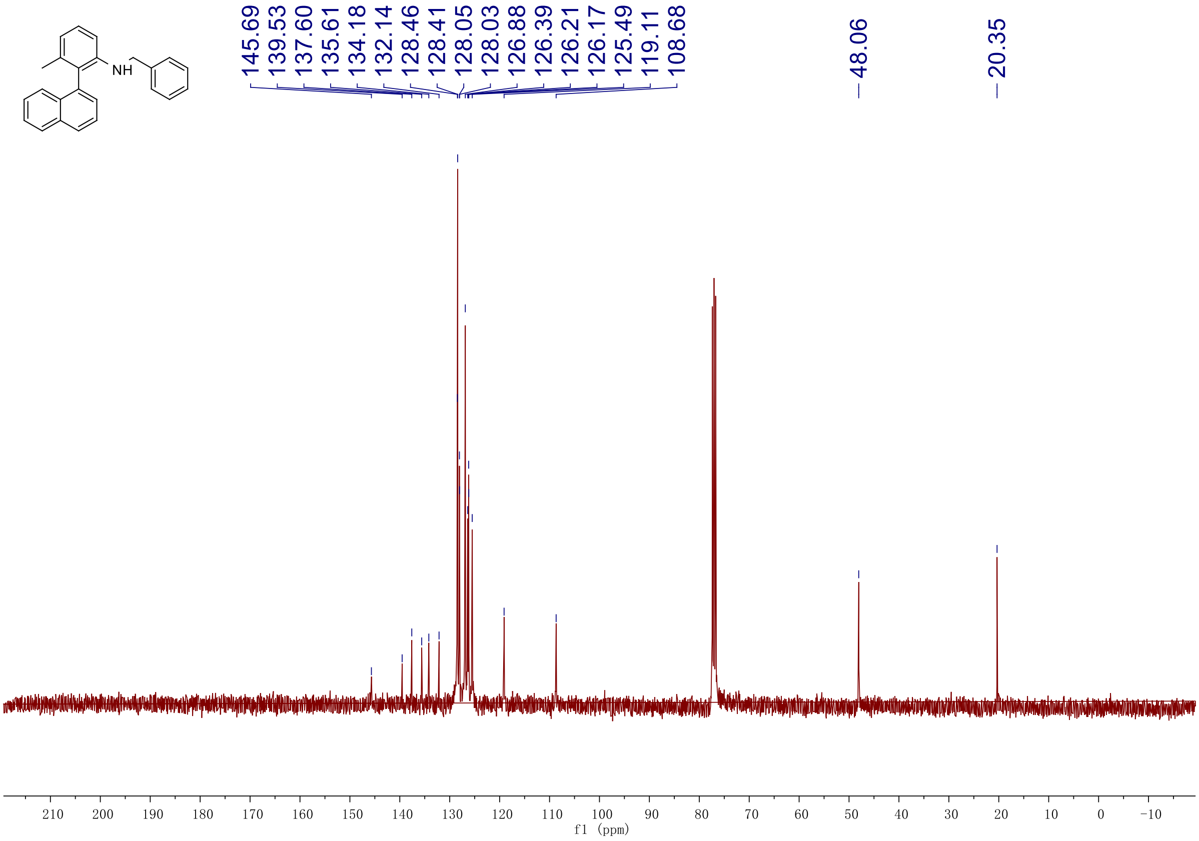

^{13}C NMR Spectrum of *N*-benzyl-3-methyl-2-(naphthalen-1-yl)aniline (5c)

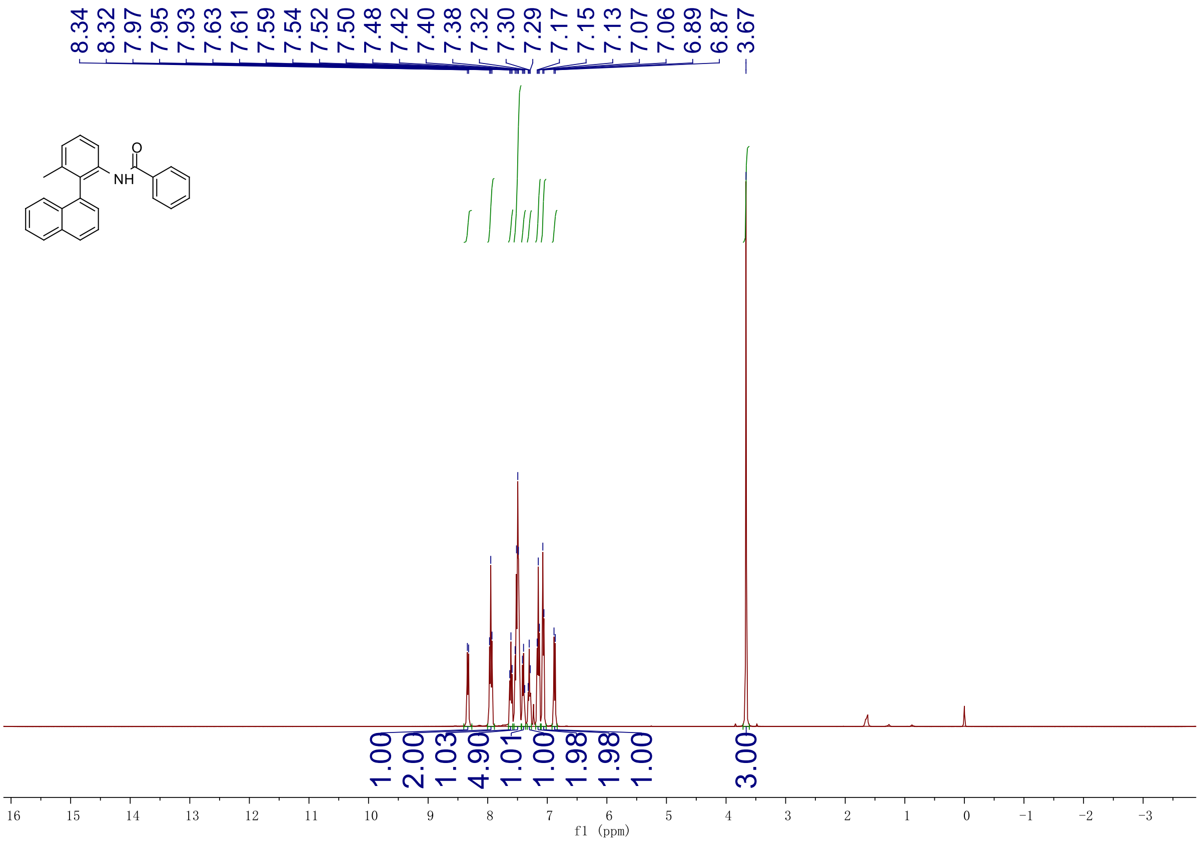

^{1}H NMR Spectrum of *N*-(3-methyl-2-(naphthalen-1-yl)phenyl)benzamide (5d)

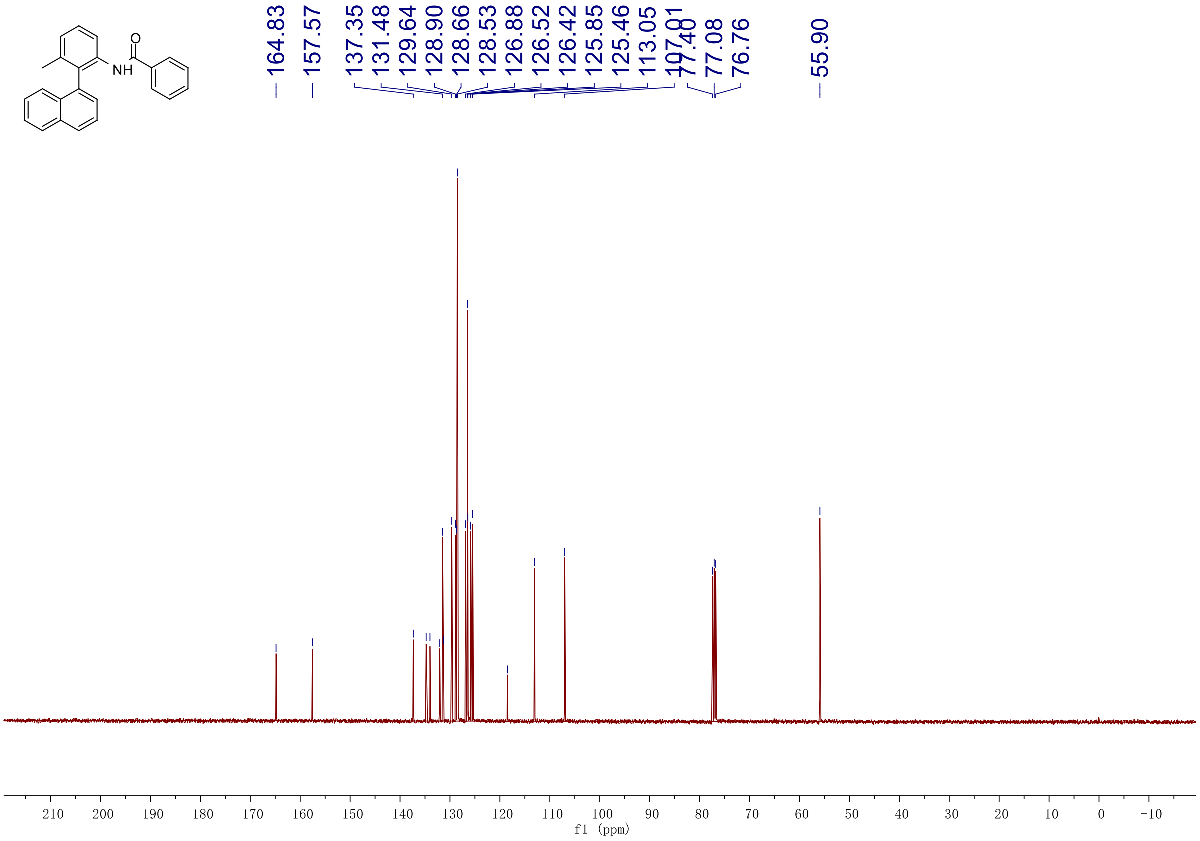

^{13}C NMR Spectrum of *N*-(3-methyl-2-(naphthalen-1-yl)phenyl)benzamide (5d)

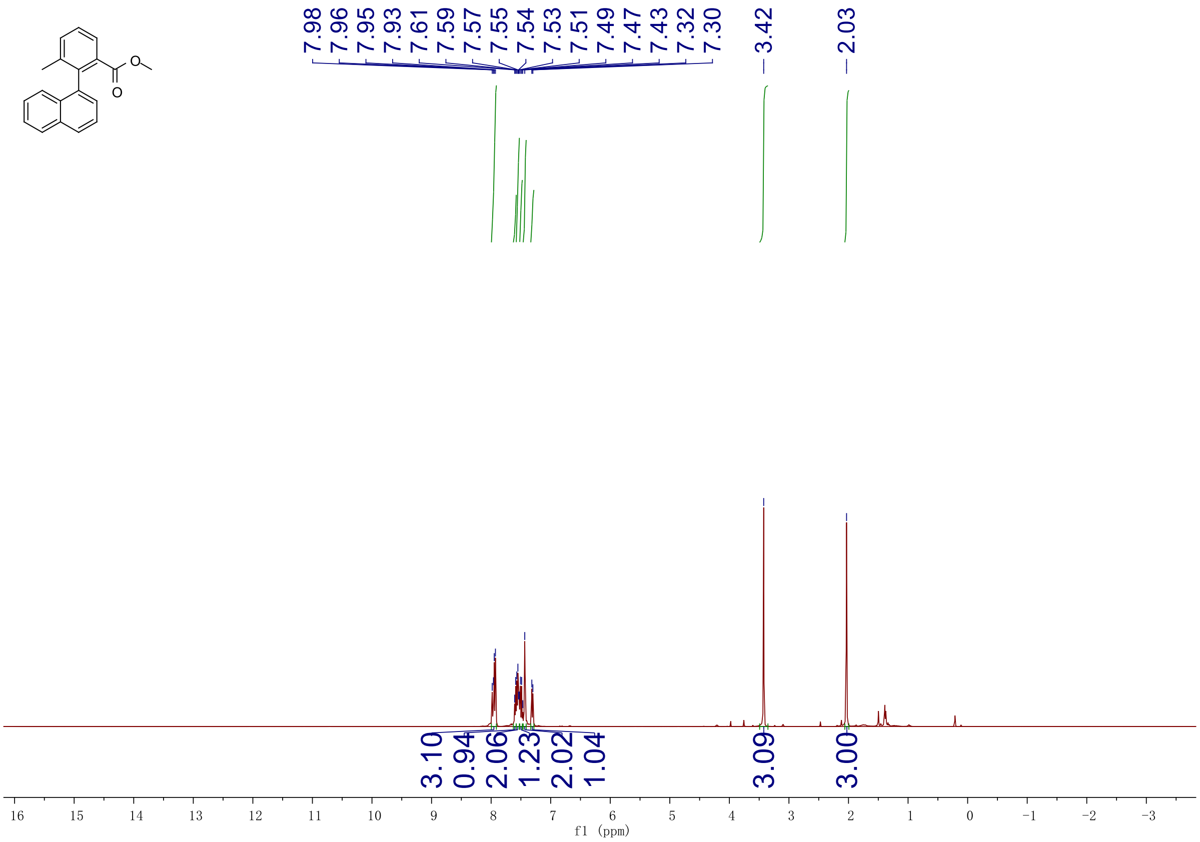

^{1}H NMR Spectrum of methyl 3-methyl-2-(naphthalen-1-yl)benzoate (5e)

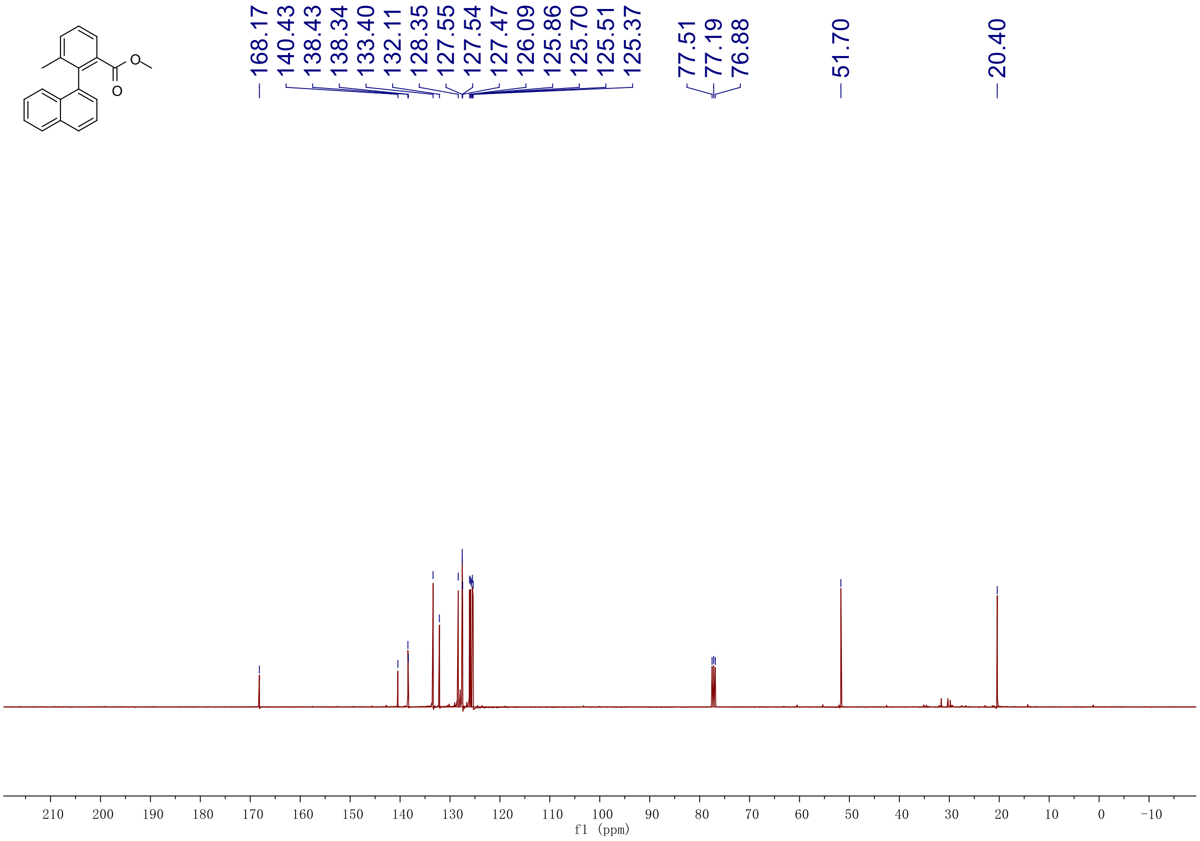

^{13}C NMR Spectrum of methyl 3-methyl-2-(naphthalen-1-yl)benzoate (5e)

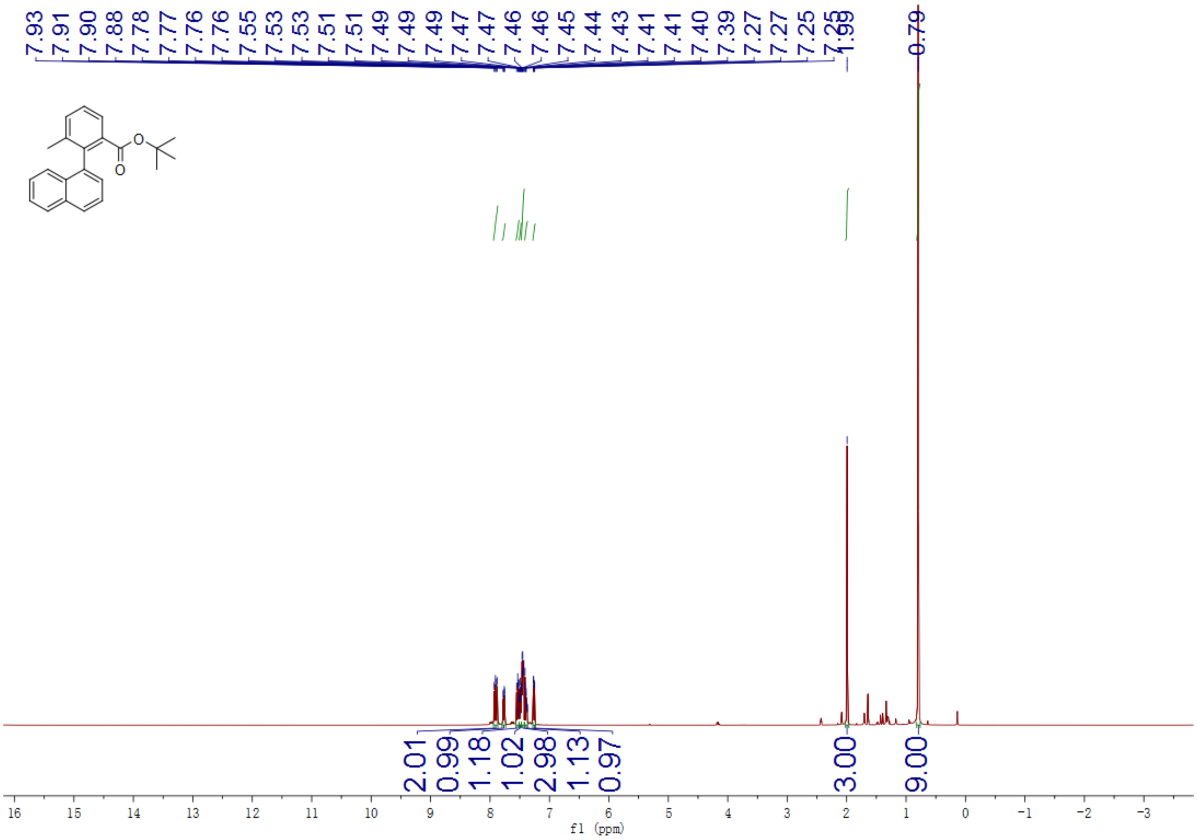

^{1}H NMR Spectrum of *tert*-butyl 3-methyl-2-(naphthalen-1-yl)benzoate (5f)

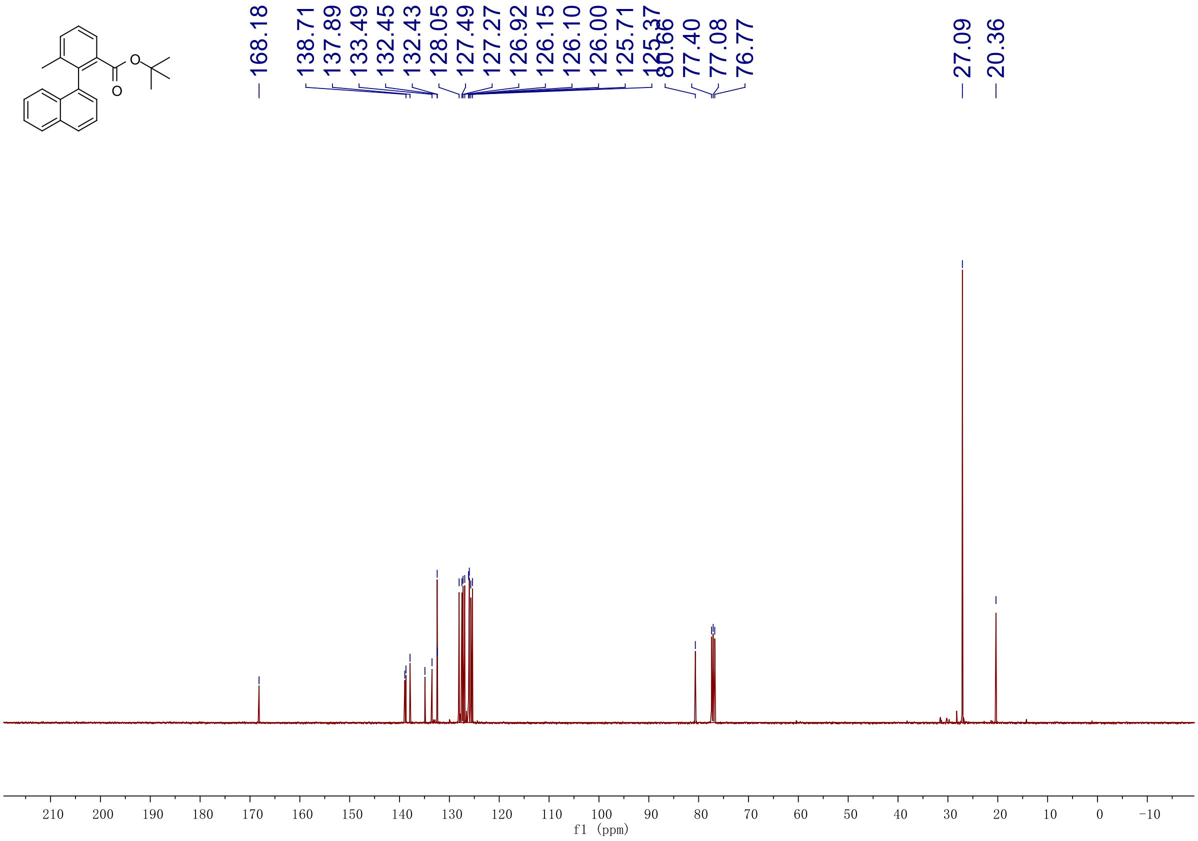

^{13}C NMR Spectrum of *tert*-butyl 3-methyl-2-(naphthalen-1-yl)benzoate (5f)

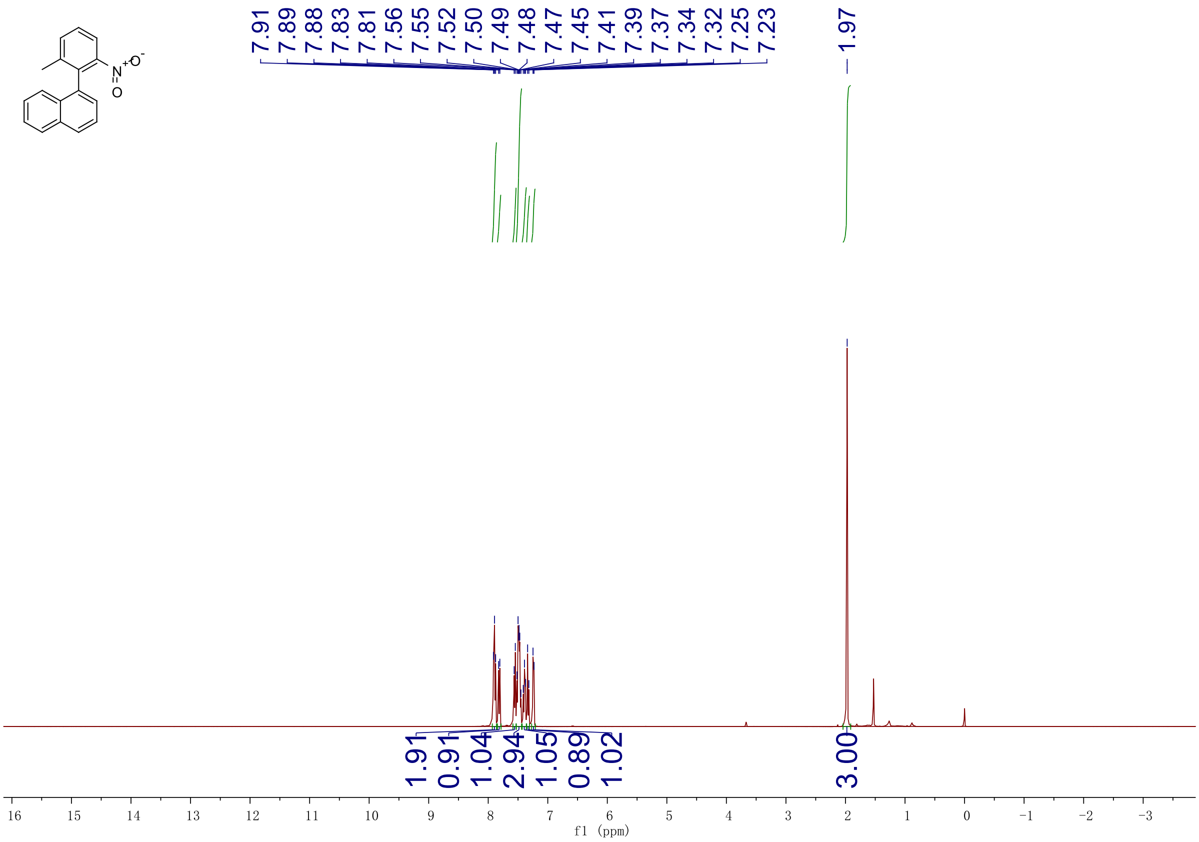

^{1}H NMR Spectrum of 1-(2-methyl-6-nitrophenyl)naphthalene (5g)

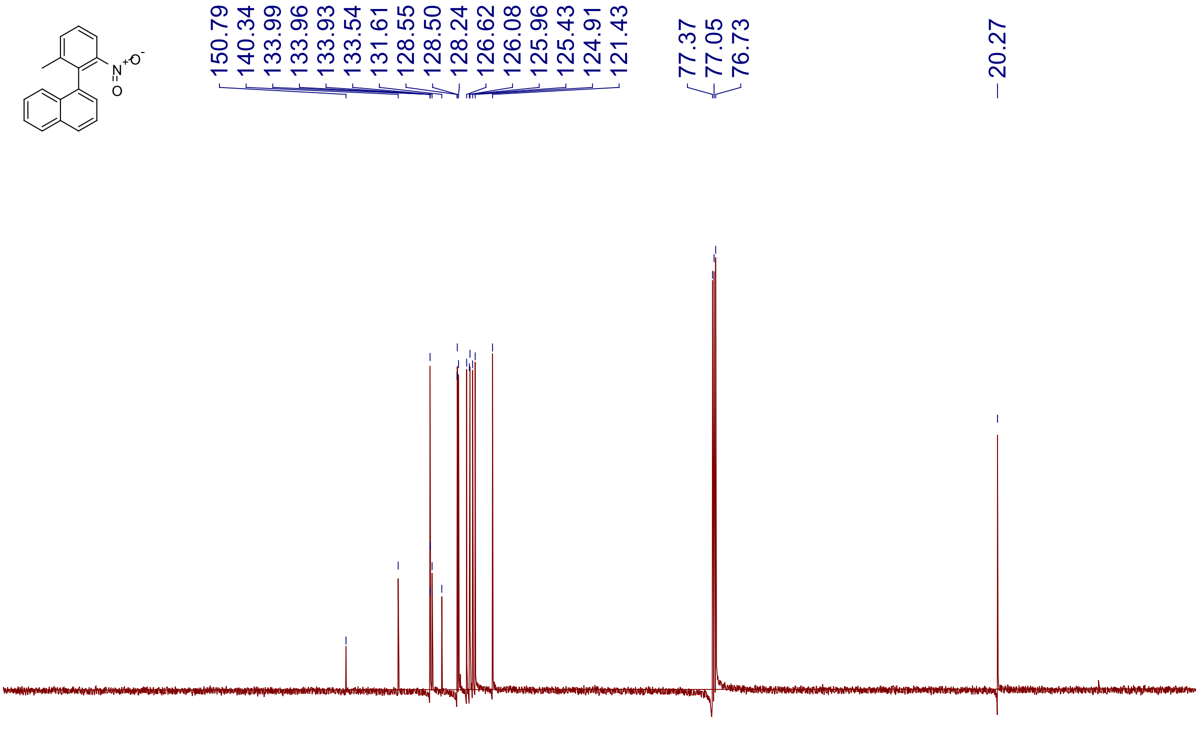

^{13}C NMR Spectrum of 1-(2-methyl-6-nitrophenyl)naphthalene (5g)

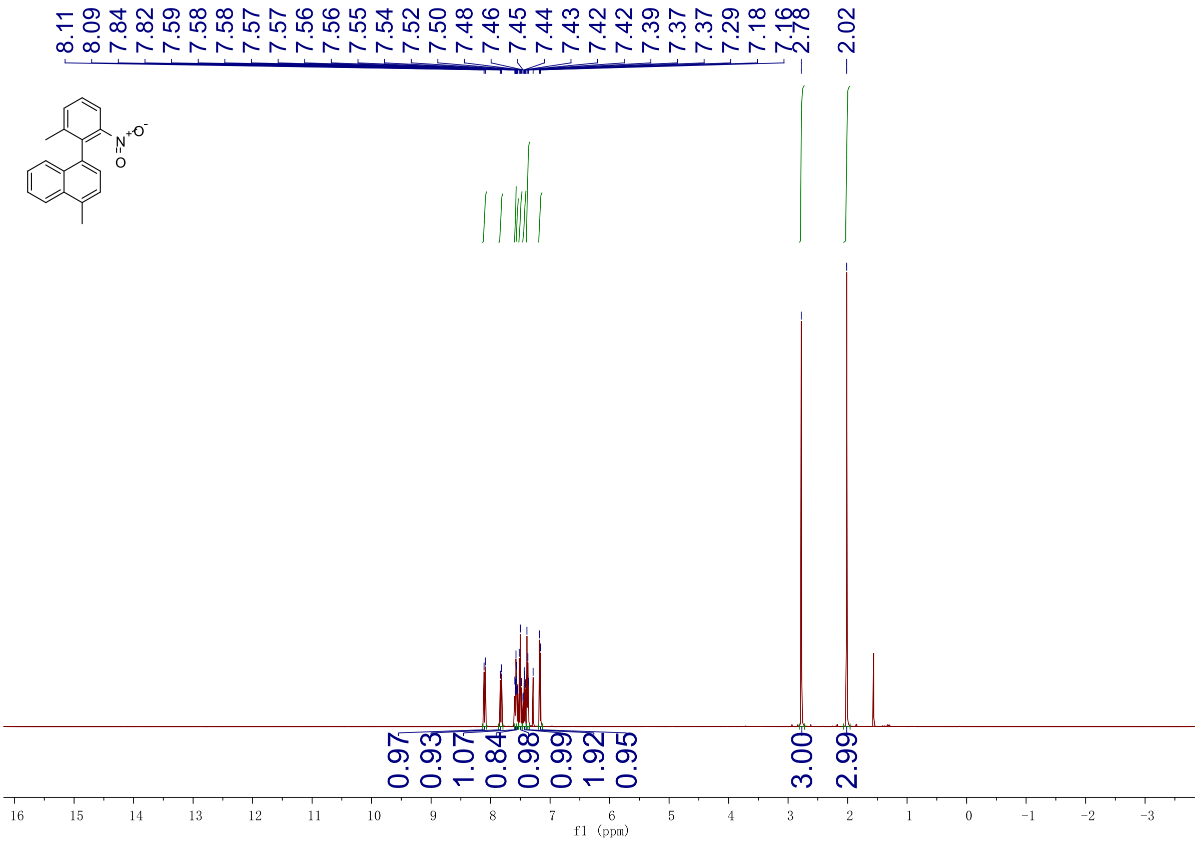

^{1}H NMR Spectrum of 1-methyl-4-(2-methyl-6-nitrophenyl)naphthalene (5h)

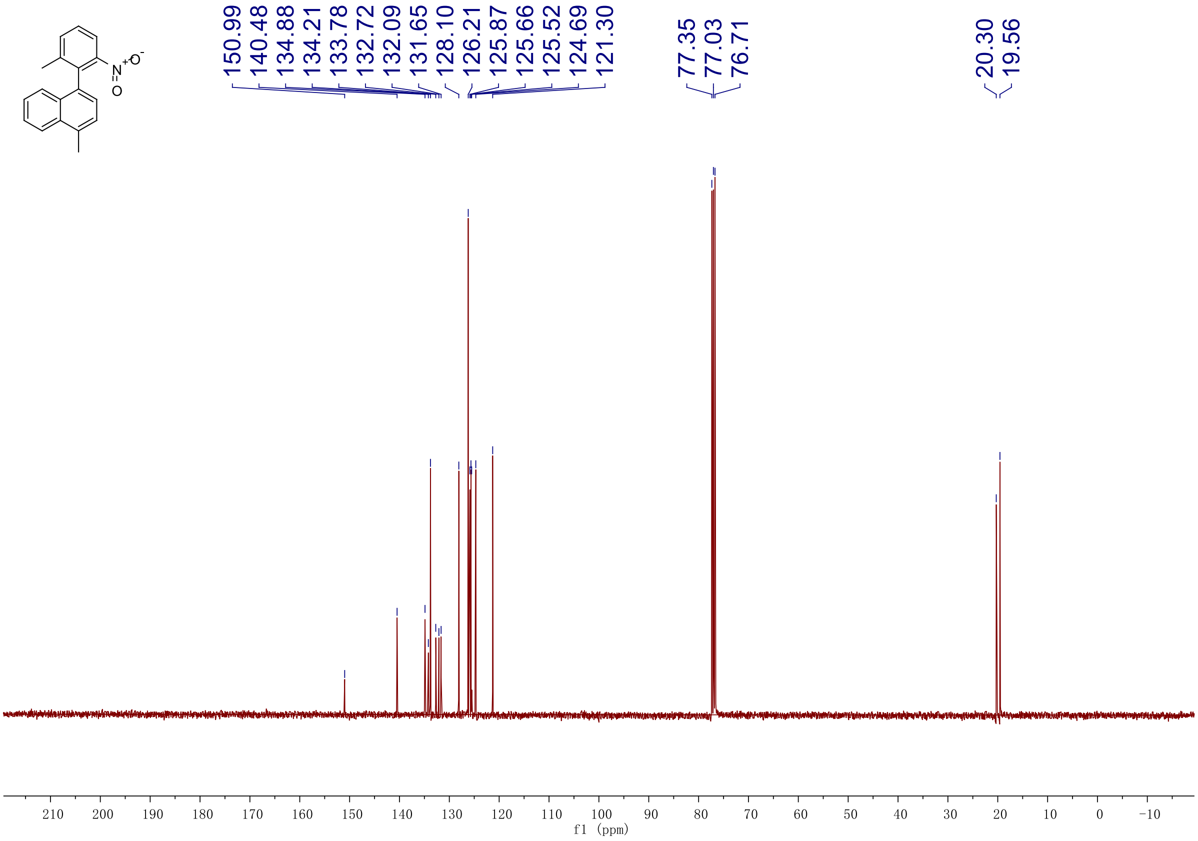

^{13}C NMR Spectrum of 1-methyl-4-(2-methyl-6-nitrophenyl)naphthalene (5h)

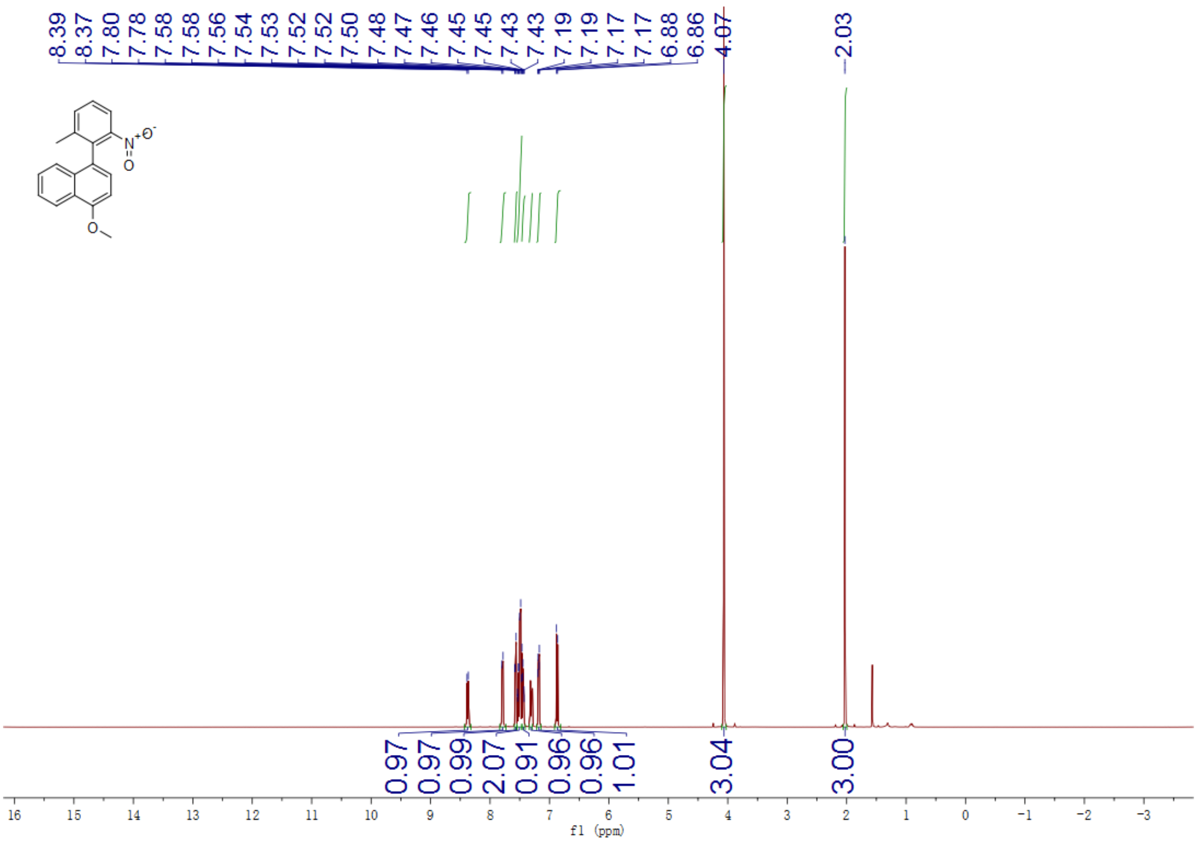

^{1}H NMR Spectrum of 1-methoxy-4-(2-methyl-6-nitrophenyl)naphthalene (5i)

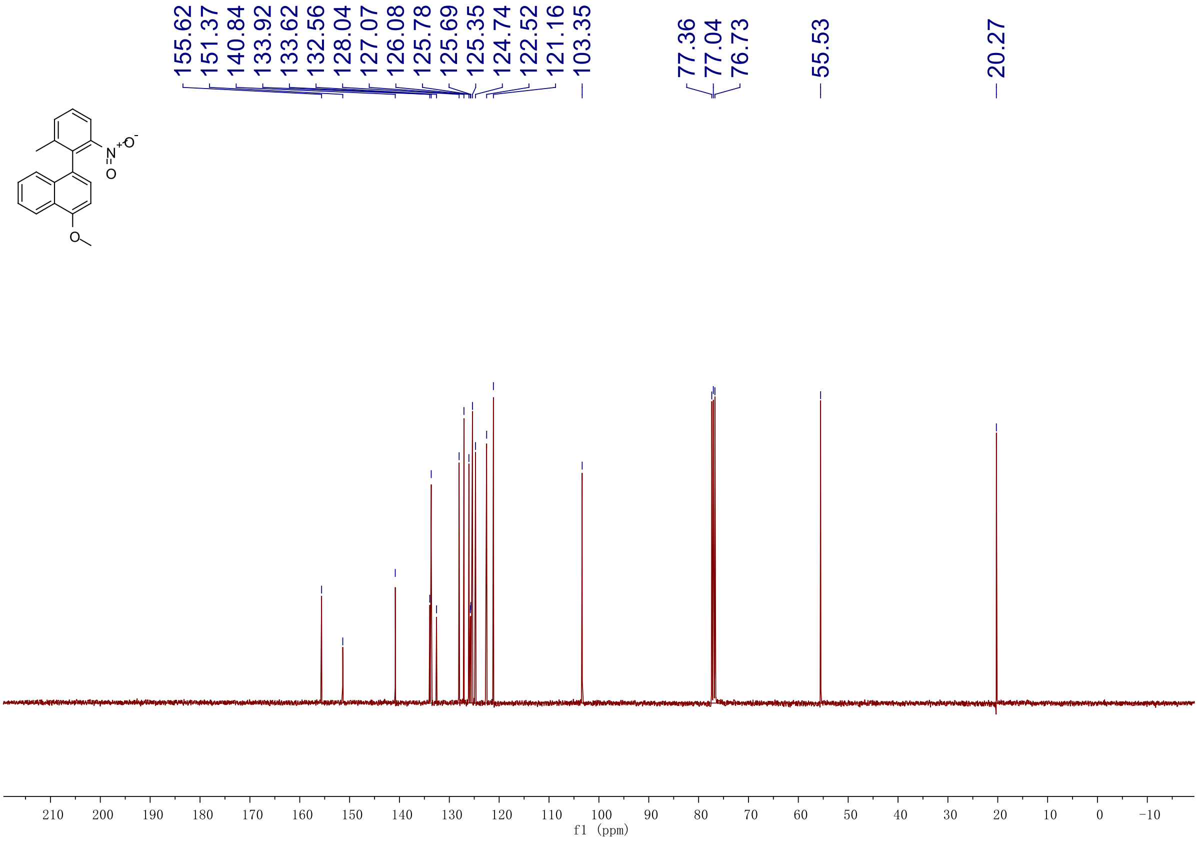

^{13}C NMR Spectrum of 1-methoxy-4-(2-methyl-6-nitrophenyl)naphthalene (5i)

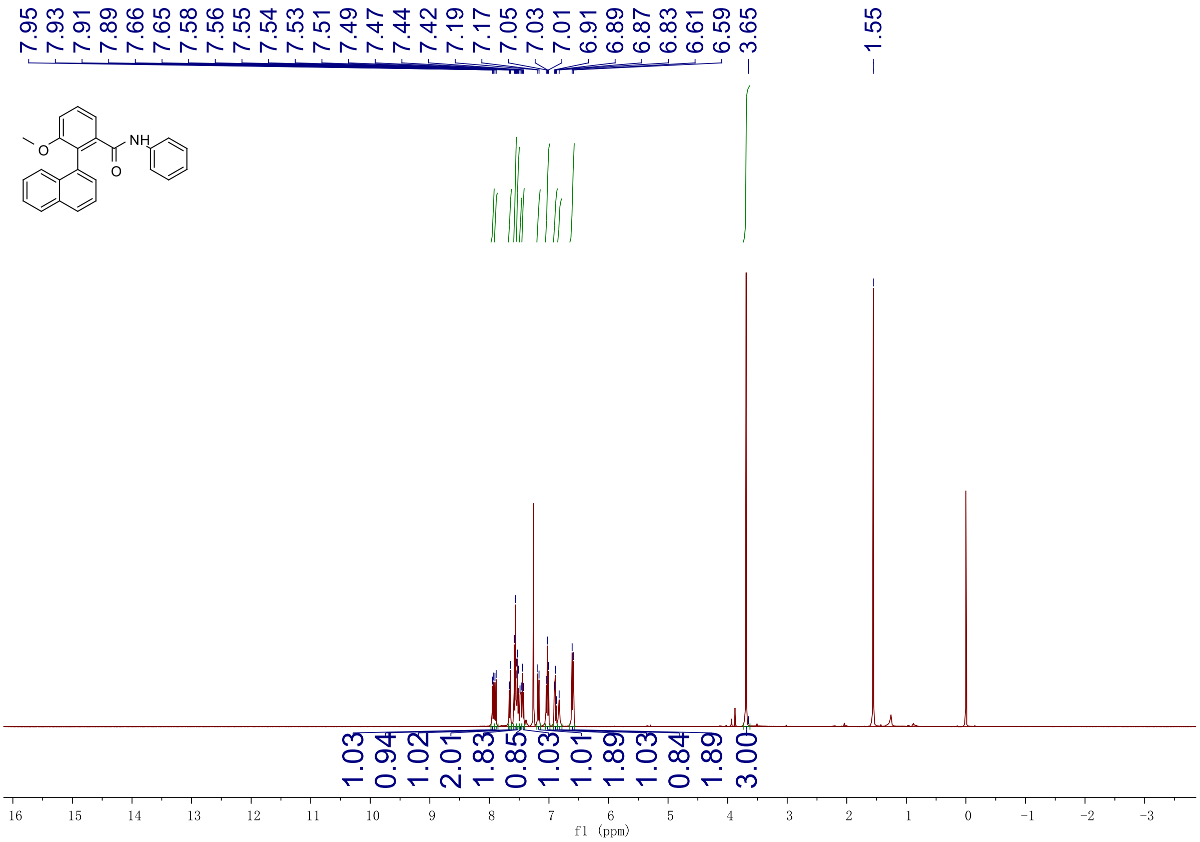

^{1}H NMR Spectrum of 3-methoxy-2-(naphthalen-1-yl)-*N*-phenylbenzamide (3s)

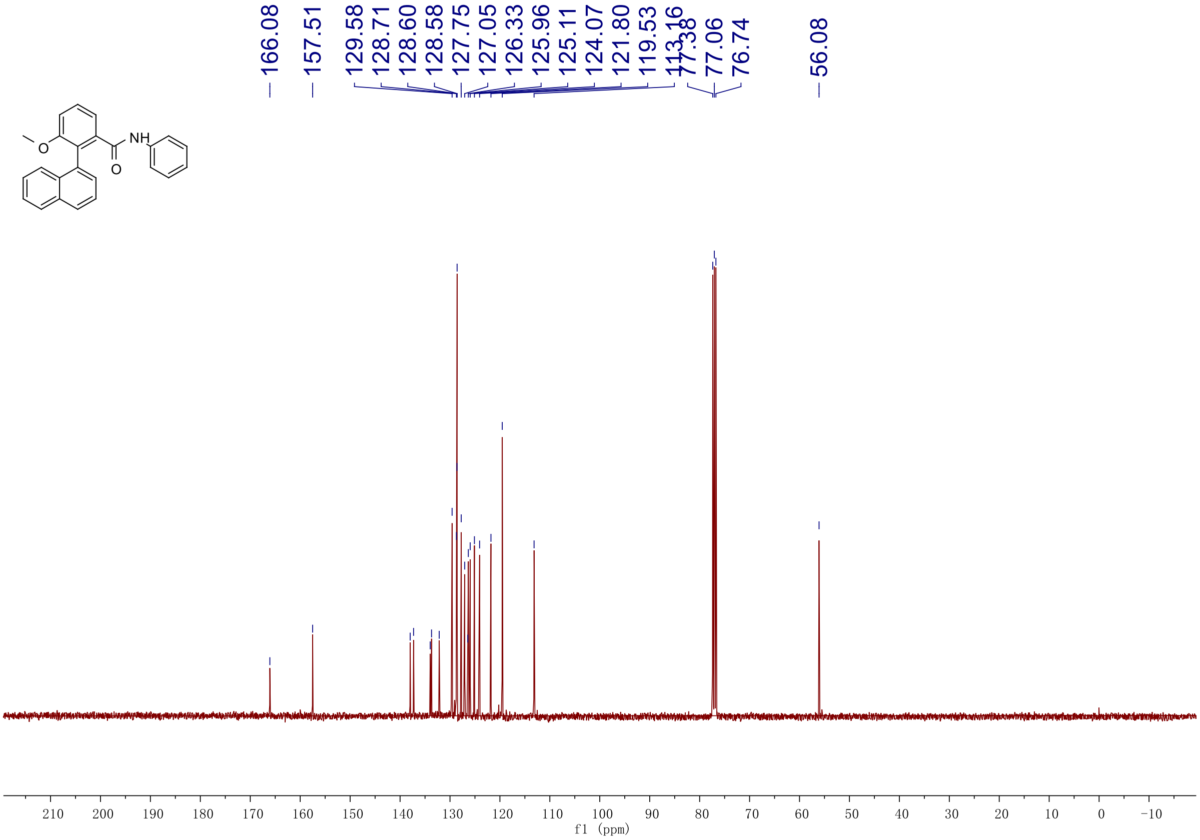

^{13}C NMR Spectrum of 3-methoxy-2-(naphthalen-1-yl)-*N*-phenylbenzamide (3s)

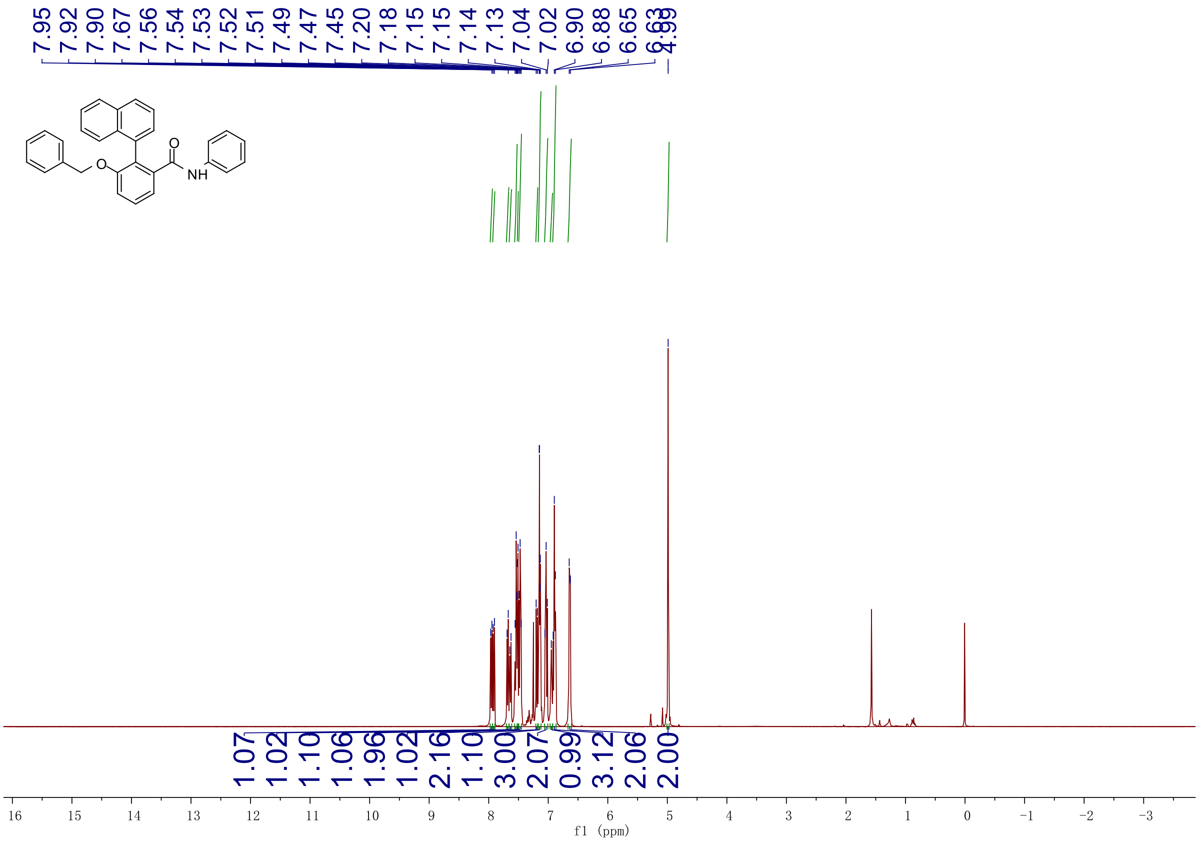

^{1}H NMR Spectrum of 3-(benzyloxy)-2-(naphthalen-1-yl)-*N*-phenylbenzamide (3t)

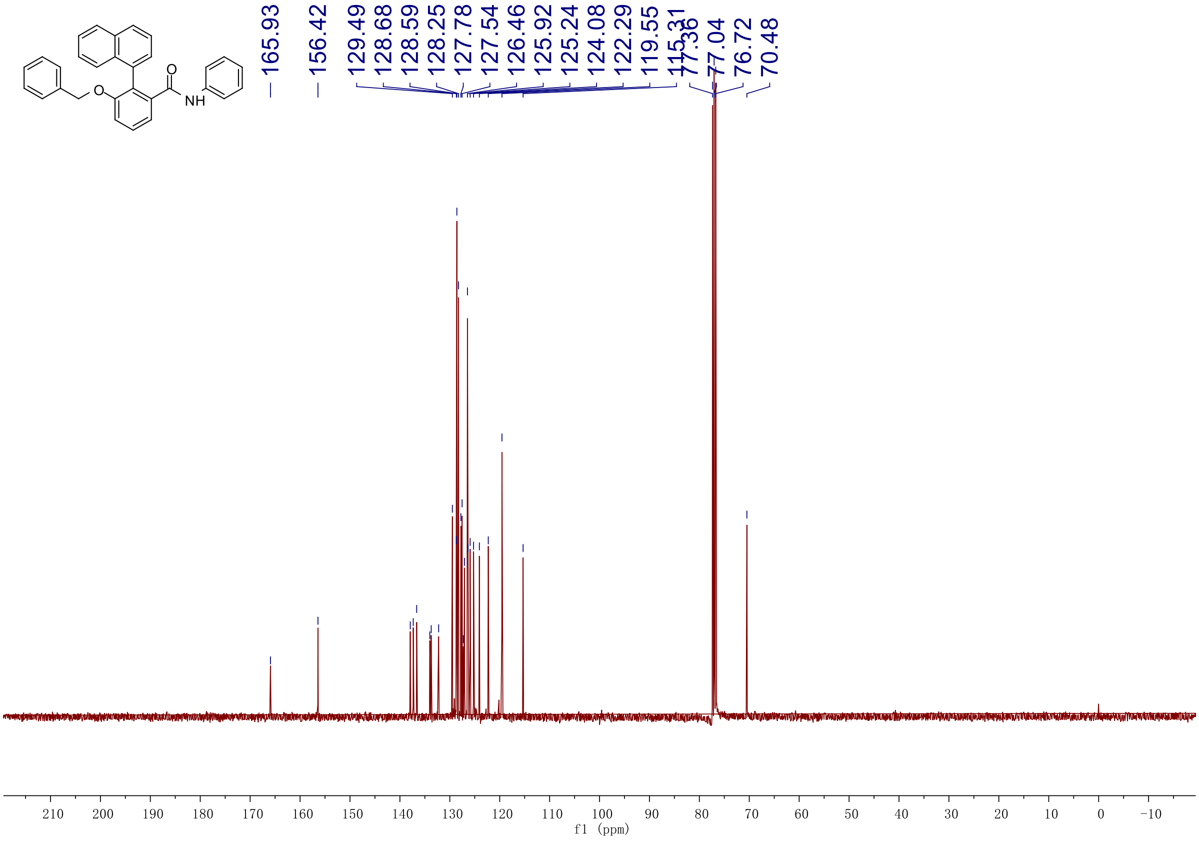

^{13}C NMR Spectrum of 3-(benzyloxy)-2-(naphthalen-1-yl)-*N*-phenylbenzamide (3t)

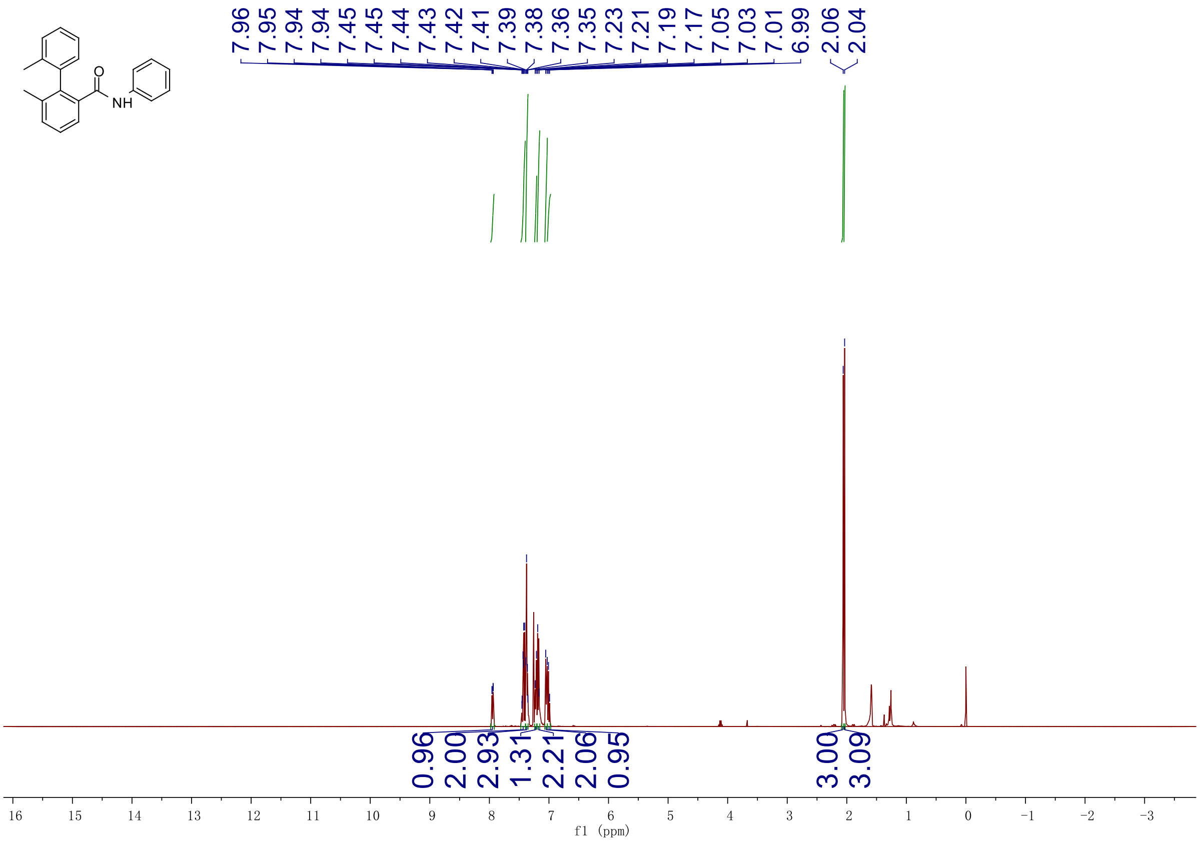

^{1}H NMR Spectrum of 2',6-dimethyl-*N*-phenyl-[1,1'-biphenyl]-2-carboxamide (3u)

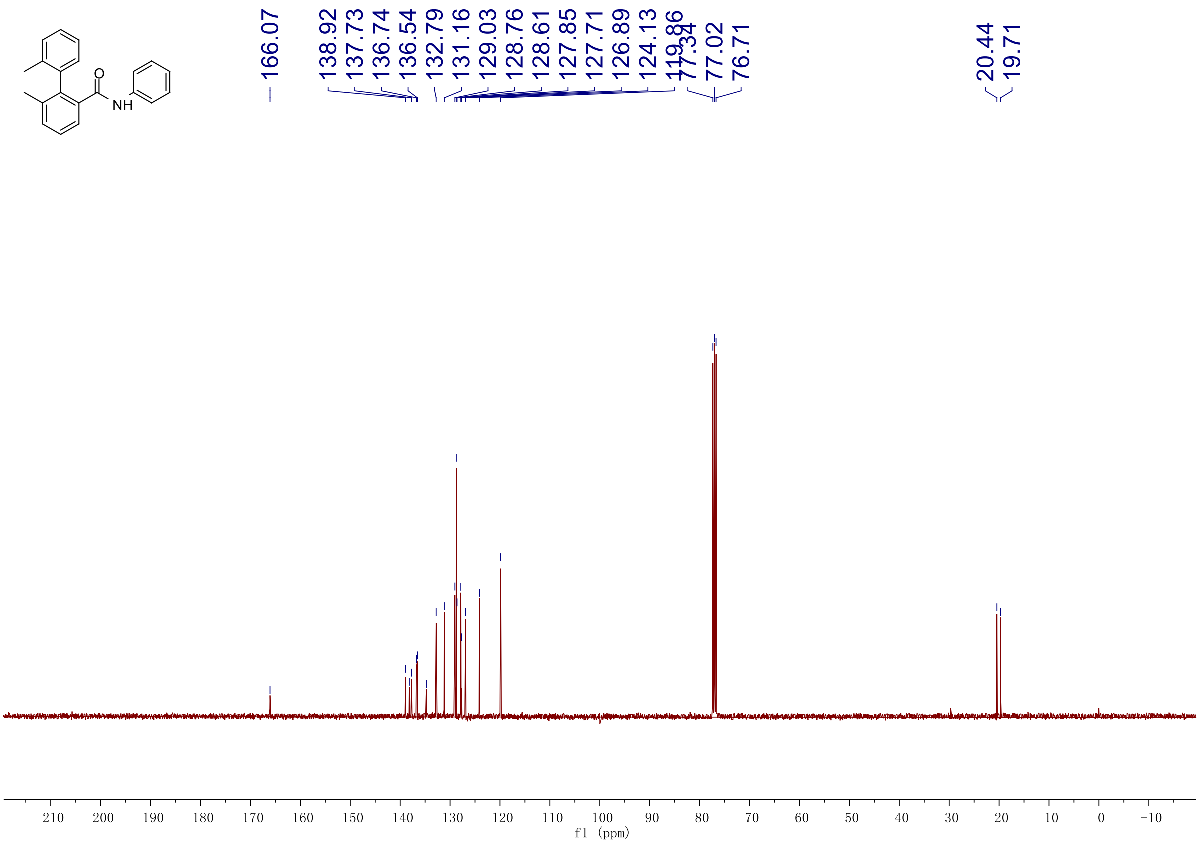

^{13}C NMR Spectrum of 2',6-dimethyl-*N*-phenyl-[1,1'-biphenyl]-2-carboxamide (3u)

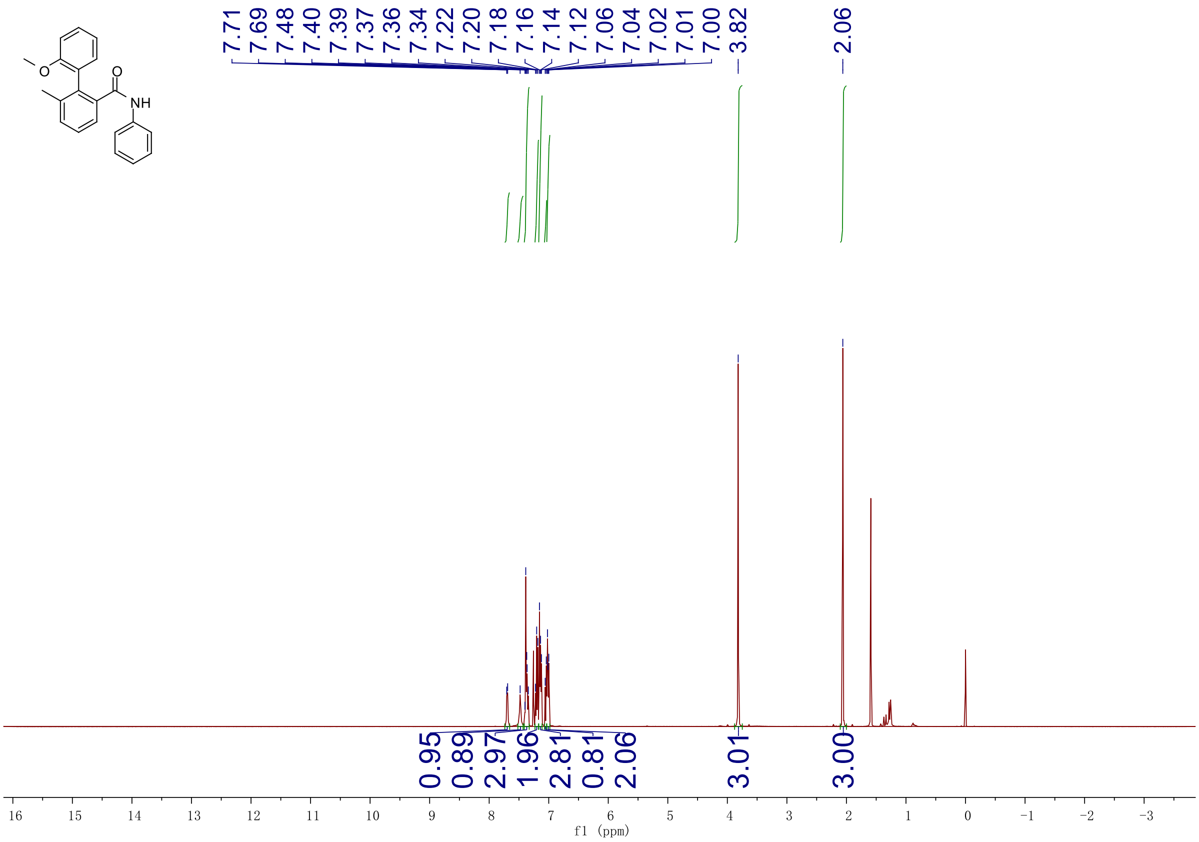

^{1}H NMR Spectrum of 2'-methoxy-6-methyl-*N*-phenyl-[1,1'-biphenyl]-2-carboxamide (3v)

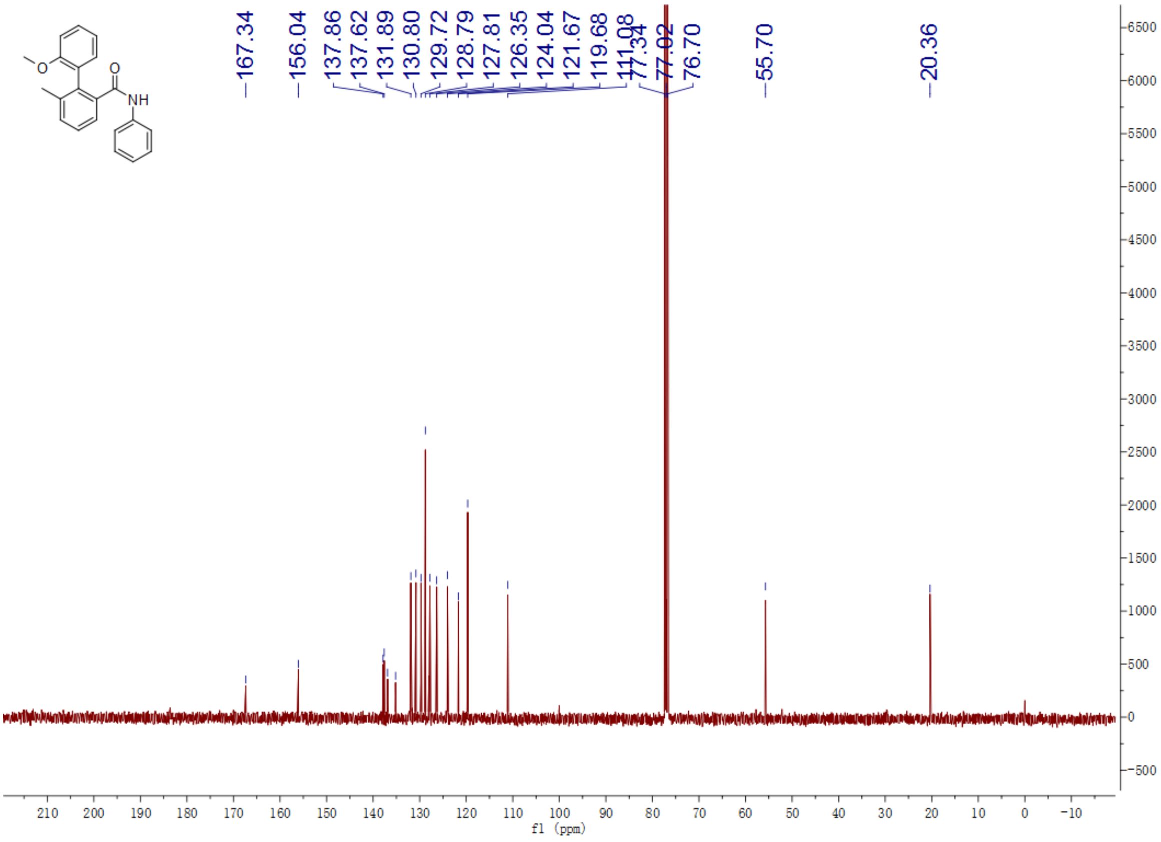

^{13}C NMR Spectrum of 2'-methoxy-6-methyl-*N*-phenyl-[1,1'-biphenyl]-2-carboxamide (3v)

Copies of HPLC Chromatograms

HPLC Spectra of 3-methyl-2-(naphthalen-1-yl)-*N*-propylbenzamide (3a)

(The top one is racemic, and the bottom one is chiral)

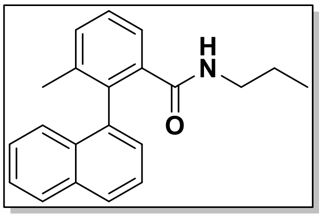

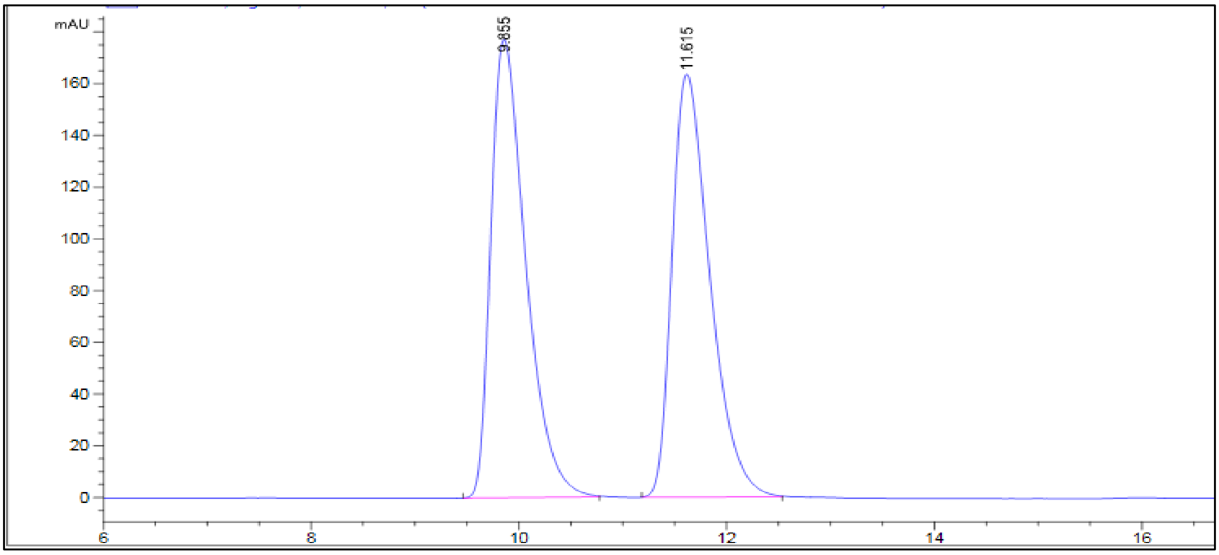

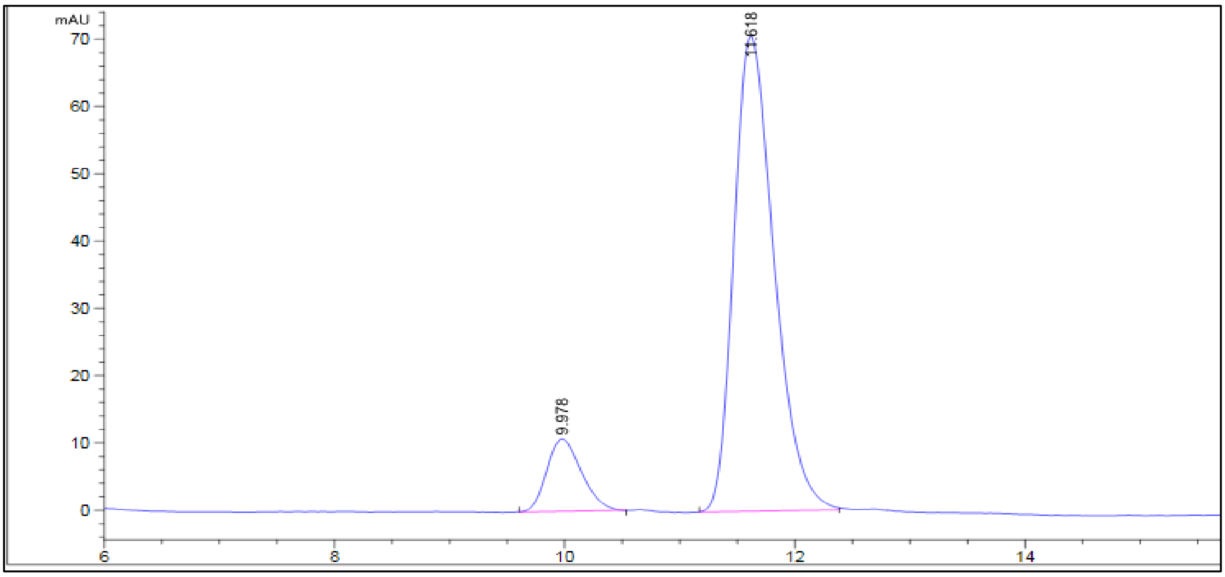

Entry #	RetTime [min]	Width [min]	Area [mAU*s]	Height [mAU]	Area %
1	9.978	0.3219	224.22993	10.72723	11.5749
2	11.618	0.3728	1712.97717	70.67101	88.4251

HPLC Spectra of *N*-isopropyl-3-methyl-2-(naphthalen-1-yl)benzamide (3b)

(The top one is racemic, and the bottom one is chiral)

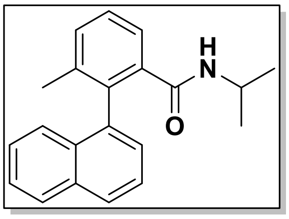

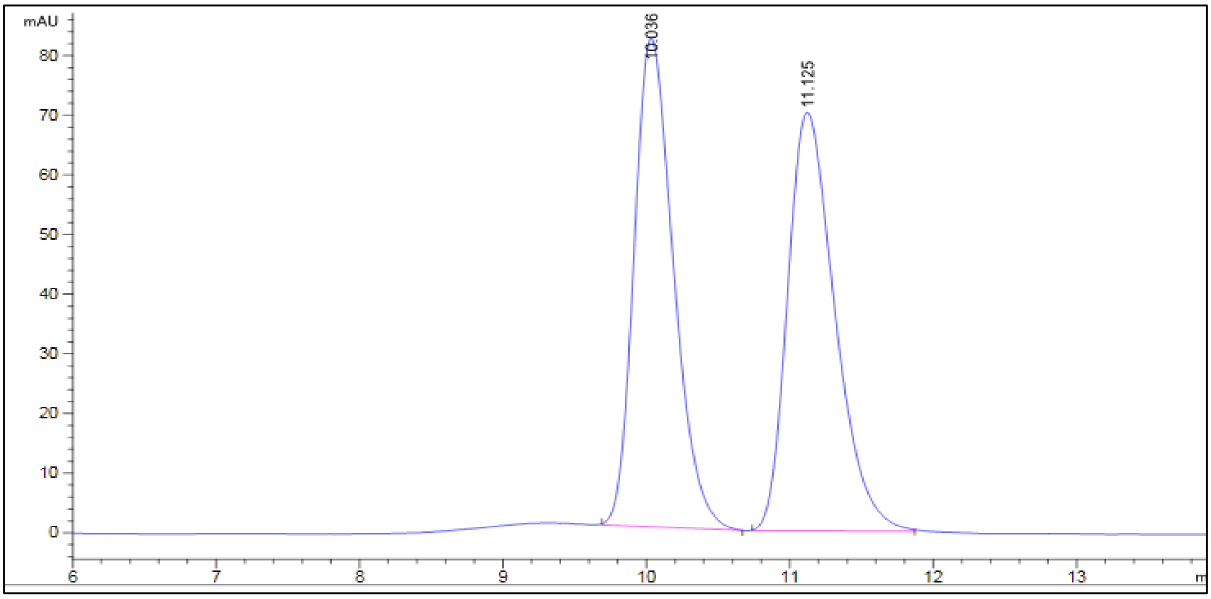

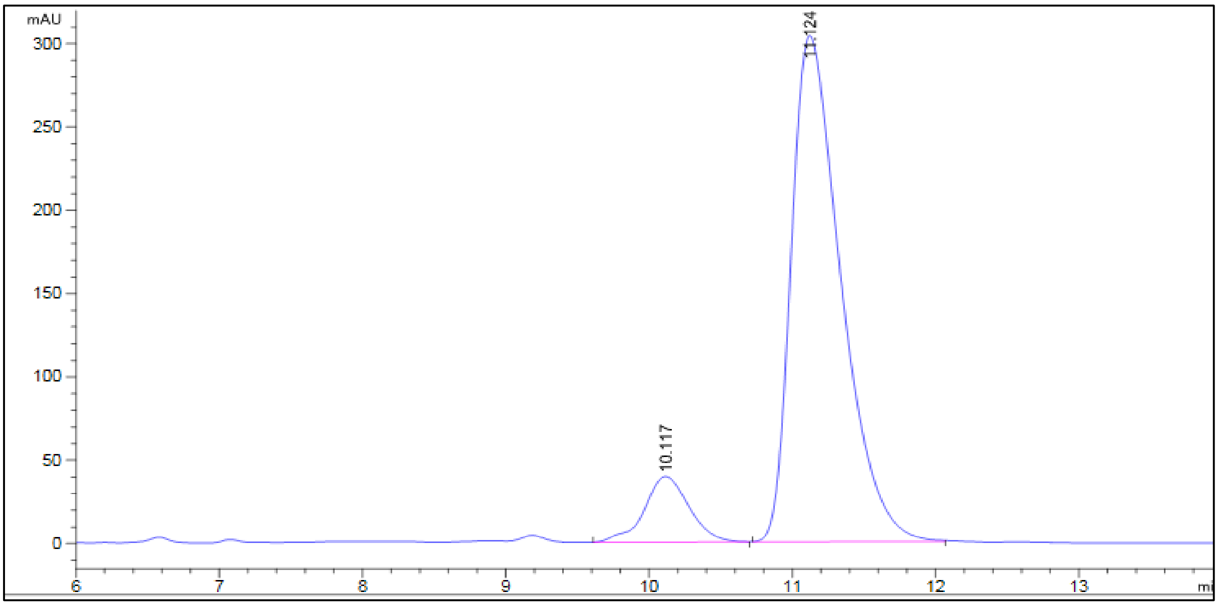

Entry #	RetTime [min]	Width [min]	Area [mAU*s]	Height [mAU]	Area %
1	10.117	0.3332	863.67535	39.47809	10.5638
2	11.124	0.3690	7312.15186	303.58383	89.4362

HPLC Spectra of *N*-cyclopentyl-3-methyl-2-(naphthalen-1-yl)benzamide (3c)

(The top one is racemic, and the bottom one is chiral)

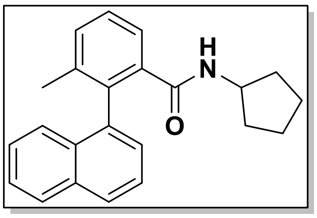

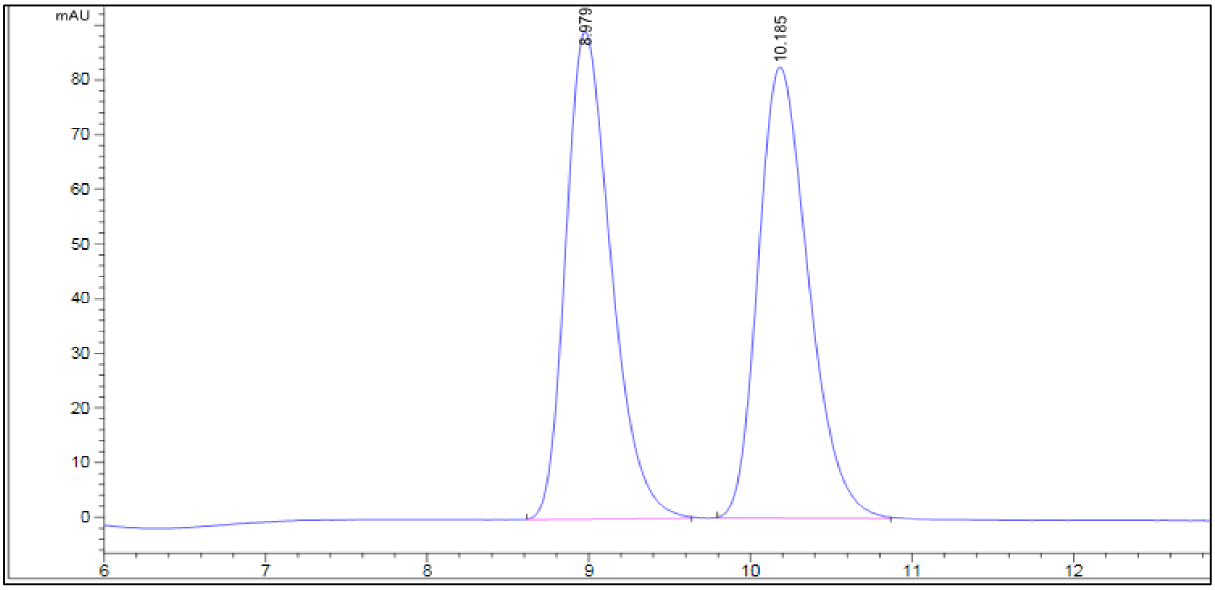

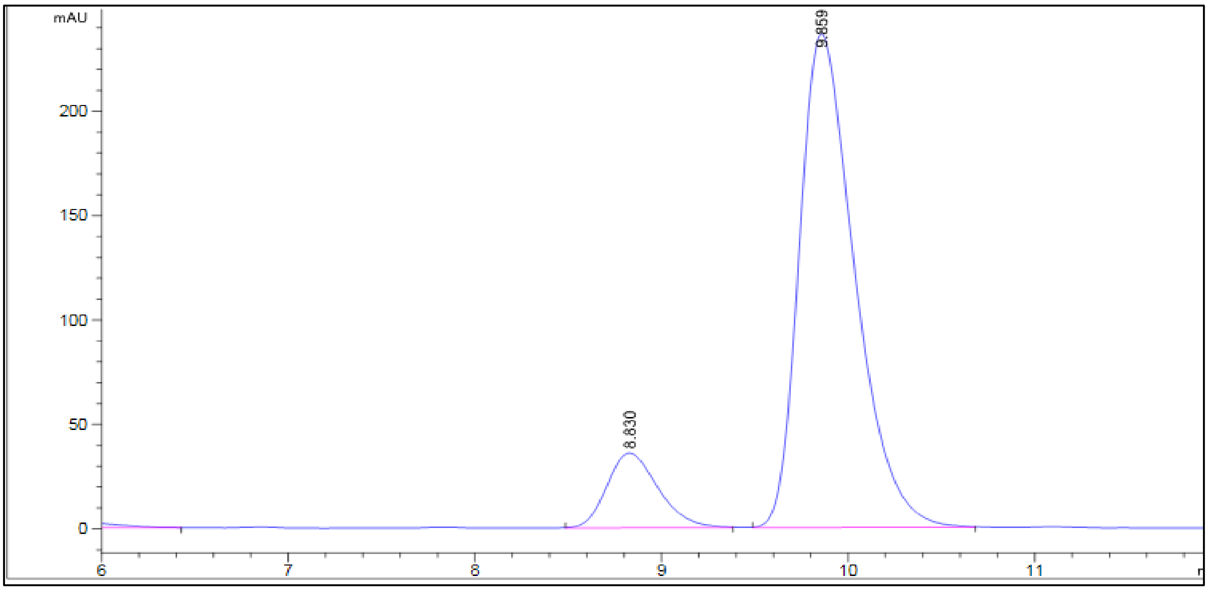

Entry #	RetTime [min]	Width [min]	Area [mAU*s]	Height [mAU]	Area %
1	8.830	0.2951	685.13452	35.82714	12.3122
2	9.859	0.3148	4879.55713	236.44583	87.6878

HPLC Spectra of *N*-cyclohexyl-3-methyl-2-(naphthalen-1-yl)benzamide (3d)

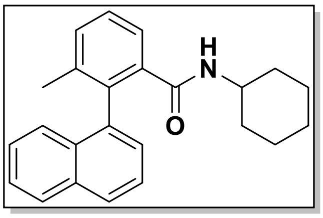

(The top one is racemic, and the bottom one is chiral)

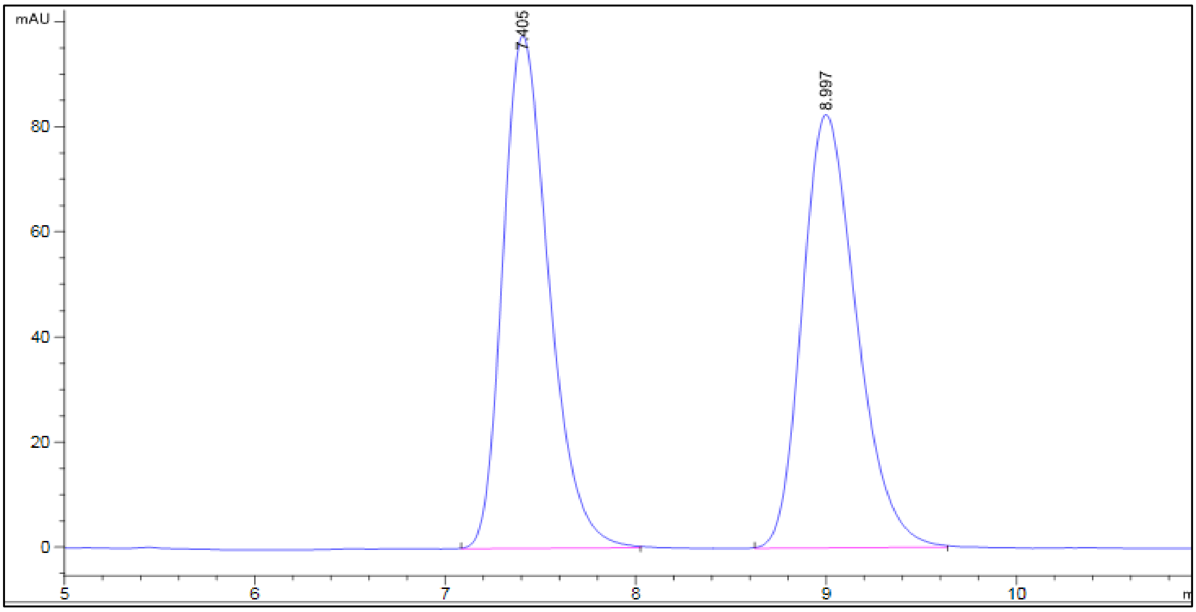

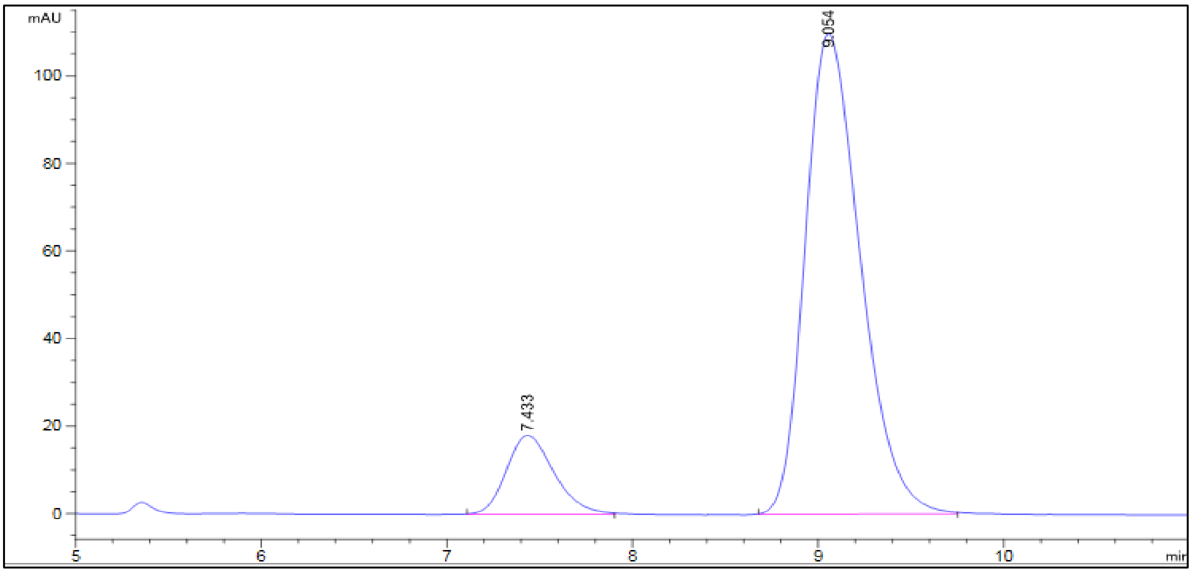

Entry #	RetTime [min]	Width [min]	Area [mAU*s]	Height [mAU]	Area %
1	7.433	0.2726	321.84262	18.00181	12.4258
2	9.054	0.3192	2268.27881	109.72440	87.5742

HPLC Spectra of 3-methyl-2-(naphthalen-1-yl)phenyl)(pyrrolidin-1-yl)methanone (3e)

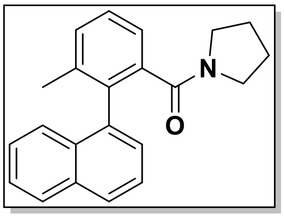

(The top one is racemic, and the bottom one is chiral)

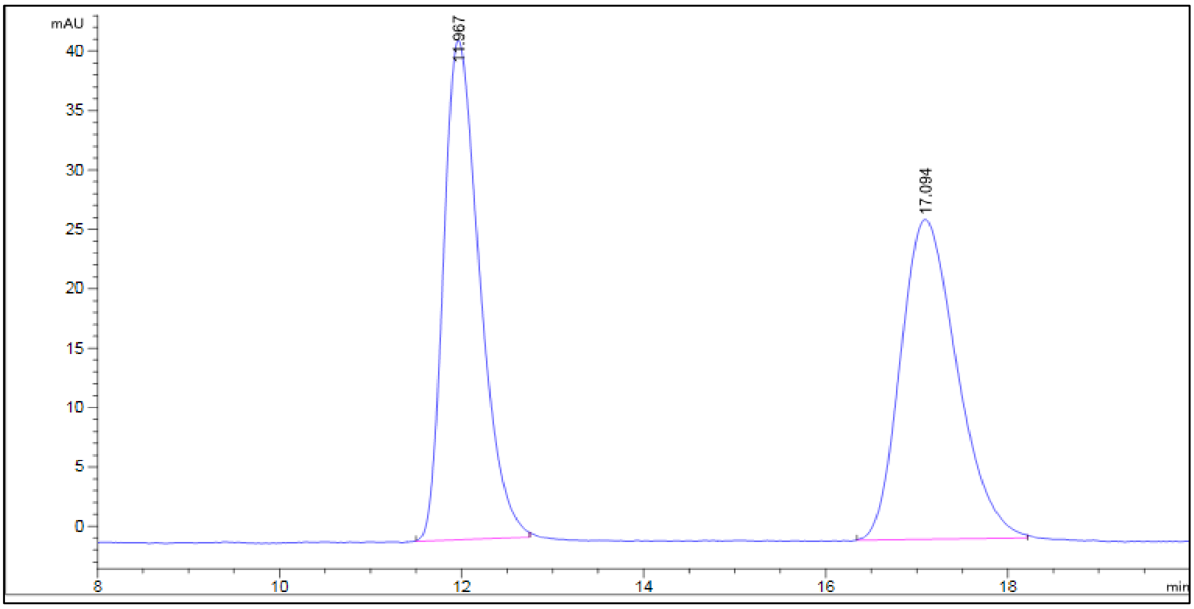

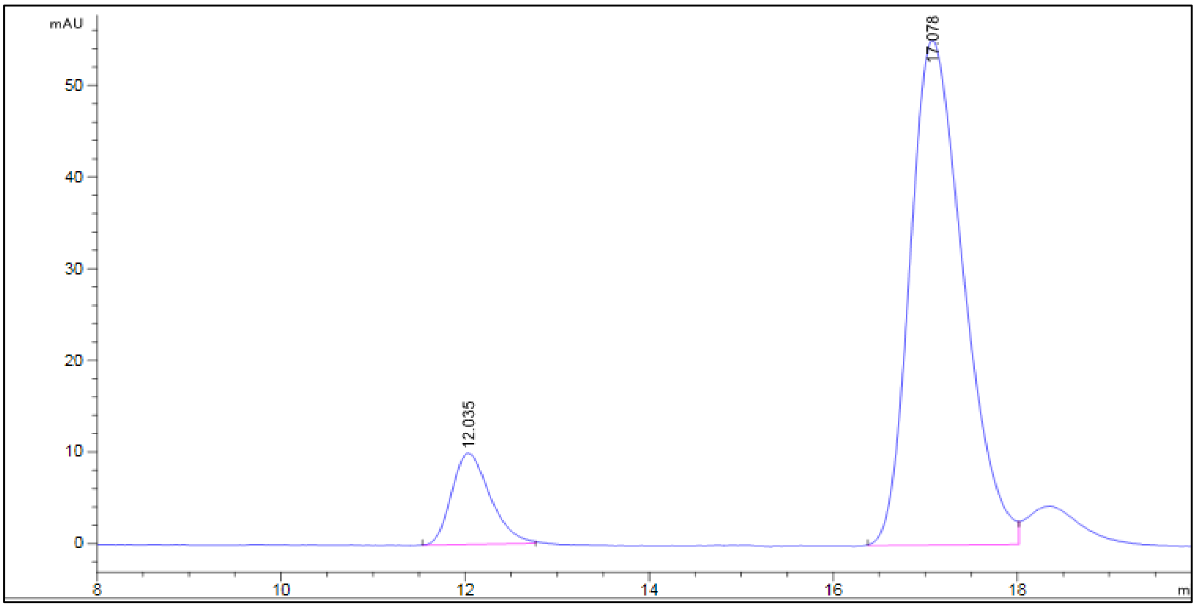

Entry #	RetTime [min]	Width [min]	Area [mAU*s]	Height [mAU]	Area %
1	12.035	0.4281	288.15387	9.94456	11.4914
2	17.078	0.6159	2219.41528	55.10642	88.5086

HPLC Spectra of 3-(3-methyl-2-(naphthalen-1-yl)benzoyl)oxazolidin-2-one (3f)

(The top one is racemic, and the bottom one is chiral)

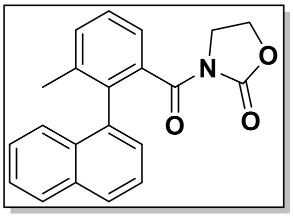

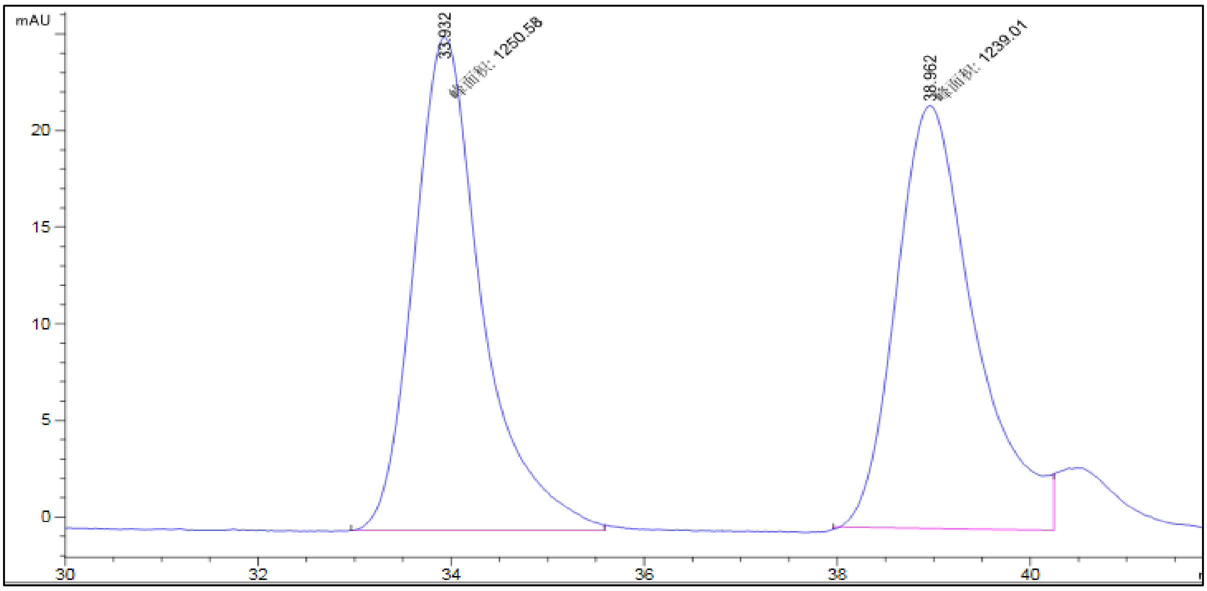

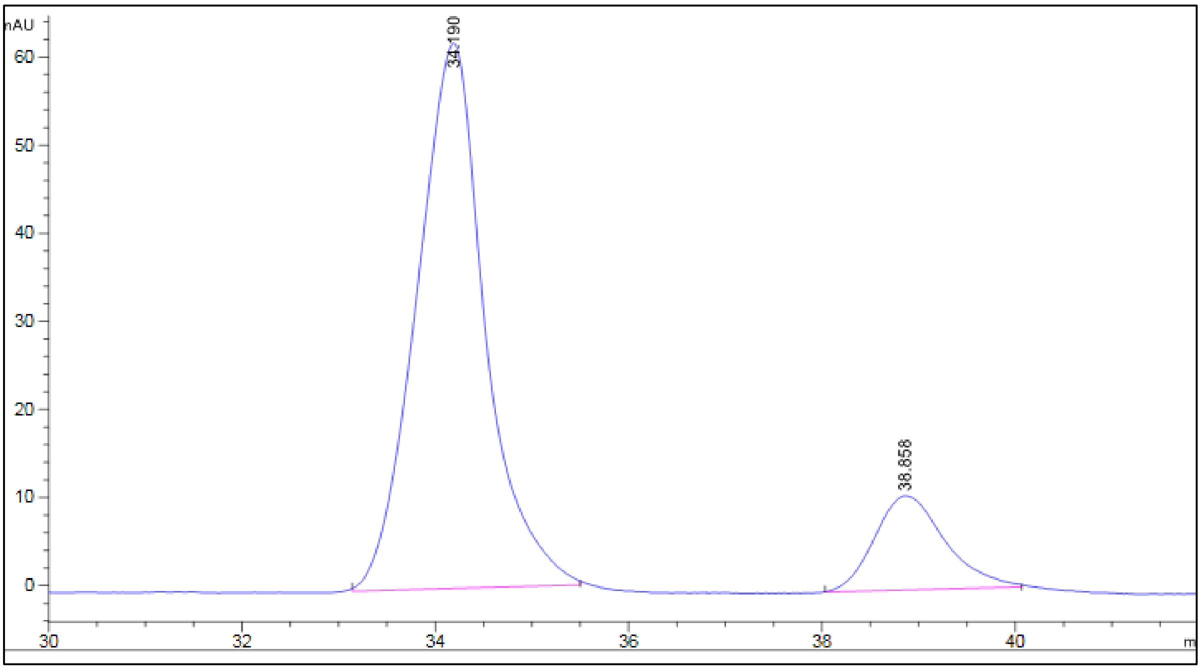

Entry #	RetTime [min]	Width [min]	Area [mAU*s]	Height [mAU]	Area %
1	34.190	0.7179	3044.07959	61.88698	84.9339
2	38.858	0.6854	539.97894	10.65198	15.0661

HPLC Spectra of 3-methyl-2-(naphthalen-1-yl)-*N*-phenylbenzamide (3g)

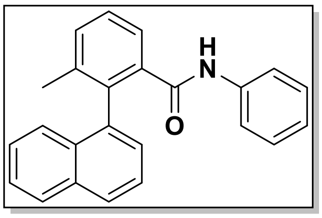

(The top one is racemic, and the bottom one is chiral)

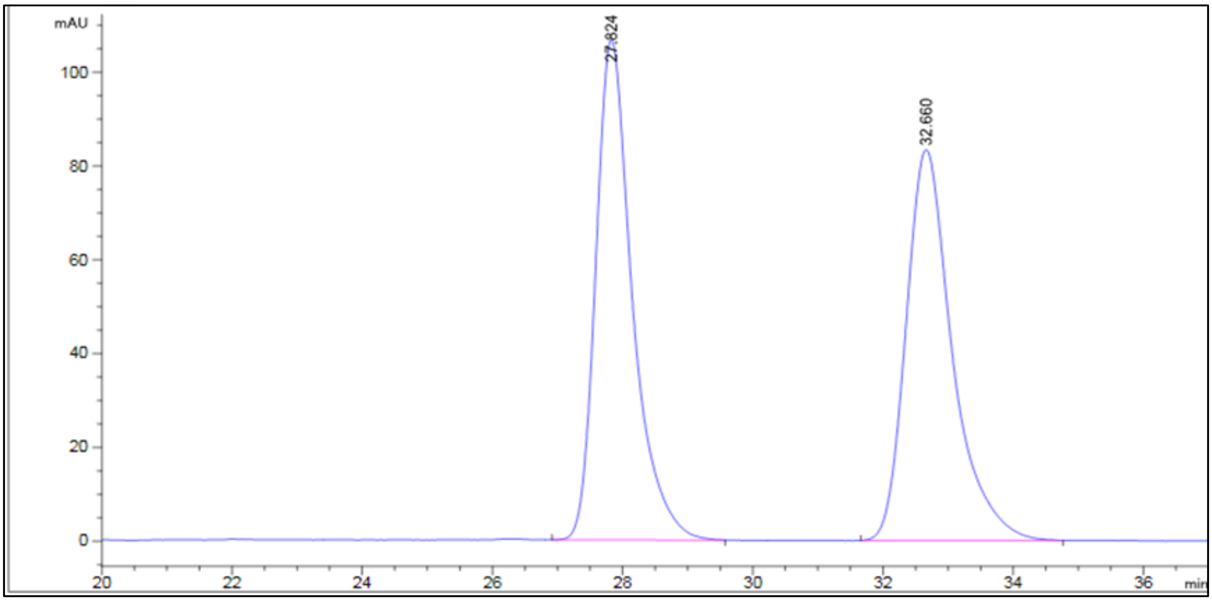

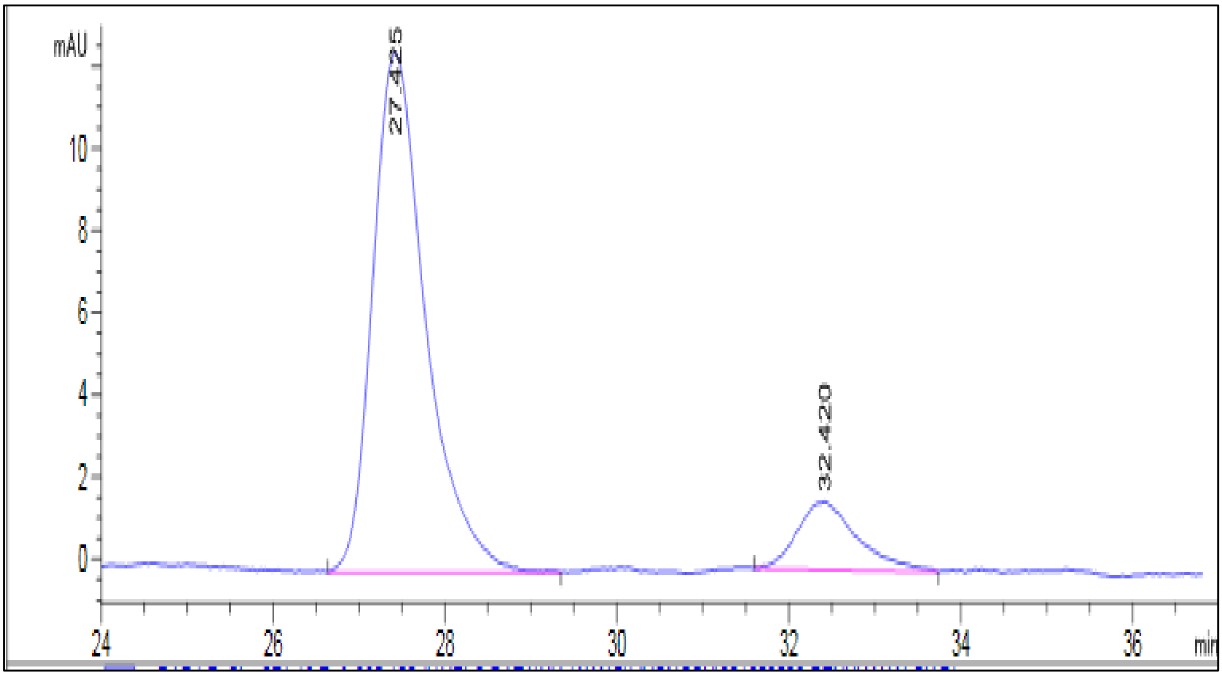

Entry #	RetTime [min]	Width [min]	Area [mAU*s]	Height [mAU]	Area %
1	27.424	0.7032	534.64655	12.67197	88.8682
2	32.416	0.7310	66.97066	1.52693	11.1318

HPLC Spectra of 3-methyl-2-(naphthalen-1-yl)-*N*-(*o*-tolyl)benzamide (3h)

(The top one is racemic, and the bottom one is chiral)

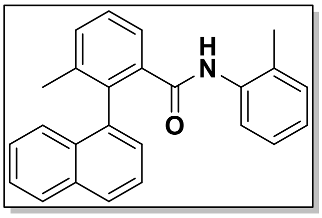

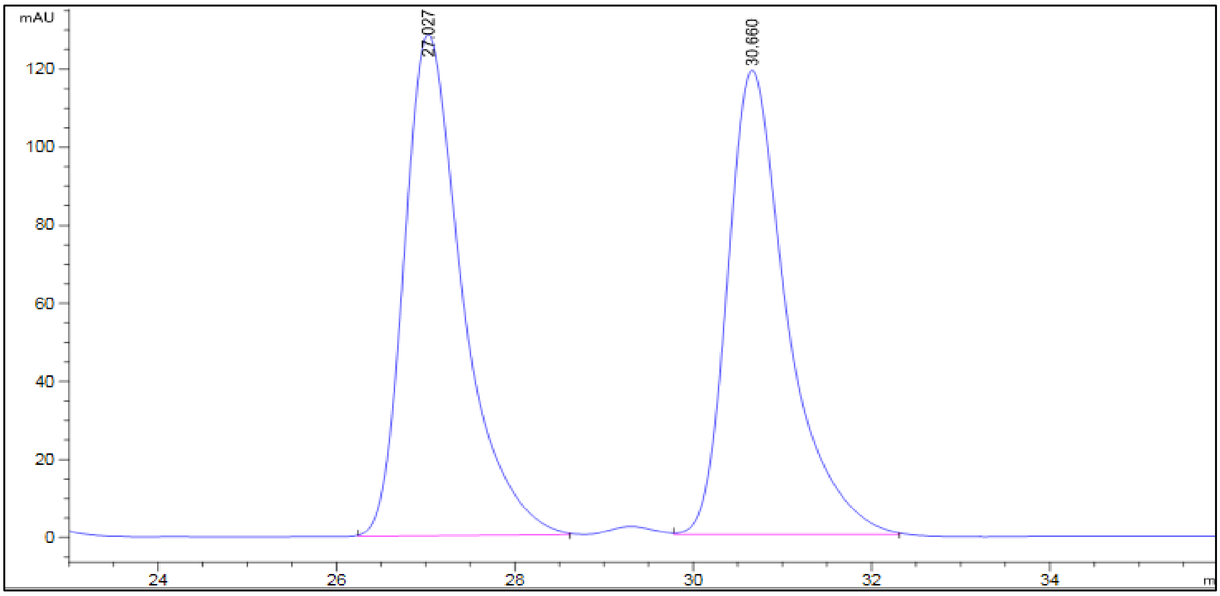

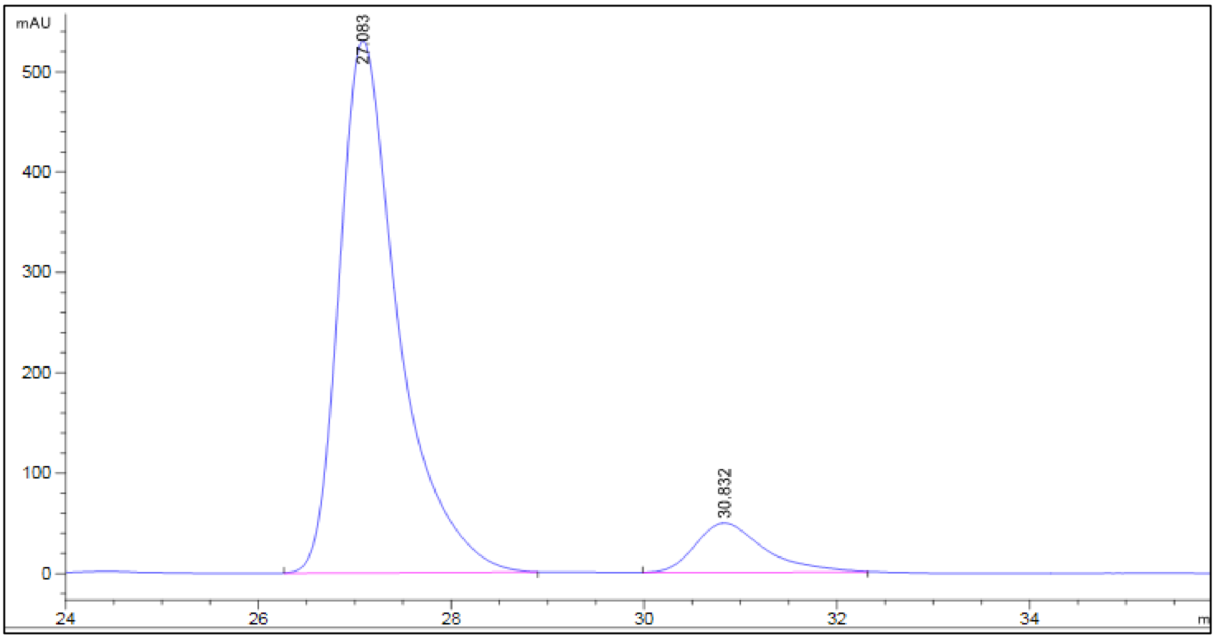

Entry #	RetTime [min]	Width [min]	Area [mAU*s]	Height [mAU]	Area %
1	27.083	0.6354	2.261604	530.45544	90.2187
2	30.832	0.7415	2451.97192	49.19078	9.7813

HPLC Spectra of 3-methyl-2-(naphthalen-1-yl)-*N*-(*m*-tolyl)benzamide (3i)

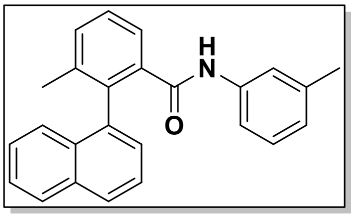

(The top one is racemic, and the bottom one is chiral)

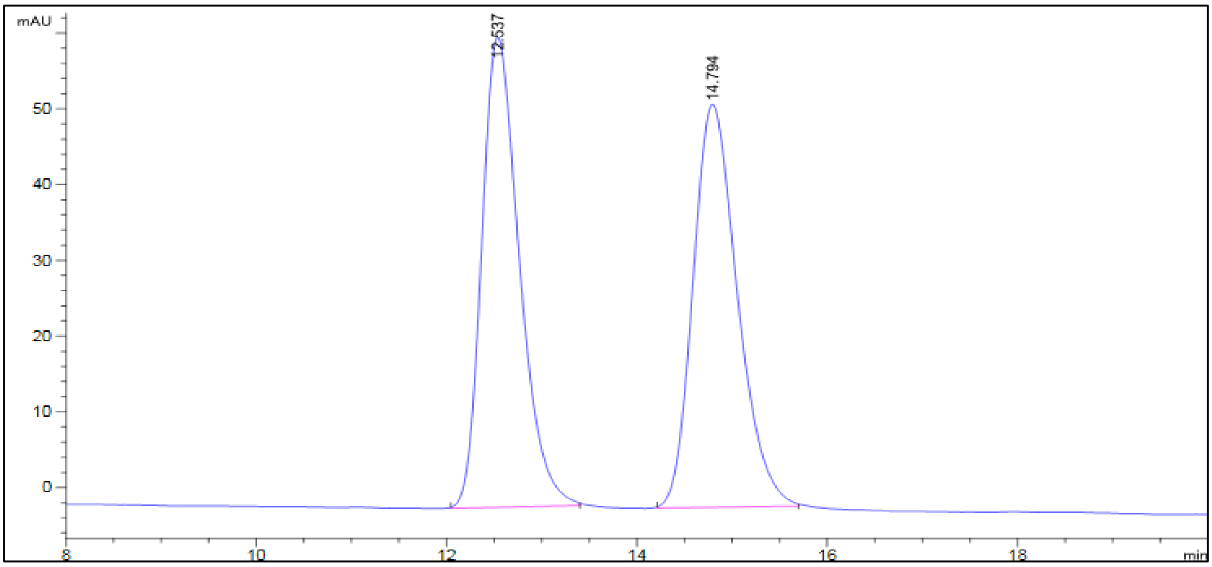

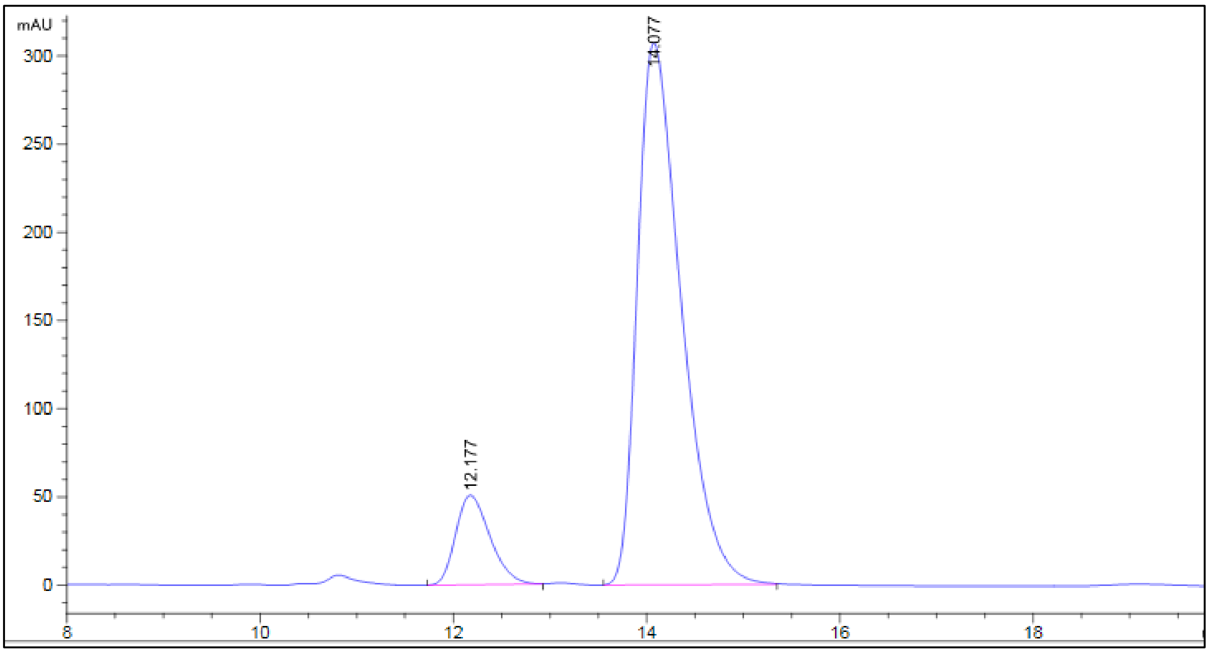

Entry #	RetTime [min]	Width [min]	Area [mAU*s]	Height [mAU]	Area %
1	12.177	0.3825	1254.59412	50.73551	11.4444
2	14.077	0.4806	9707.88672	307.29858	88.5556

HPLC Spectra of 3-methyl-2-(naphthalen-1-yl)-*N*-(*p*-tolyl)benzamide (3j)

(The top one is racemic, and the bottom one is chiral)

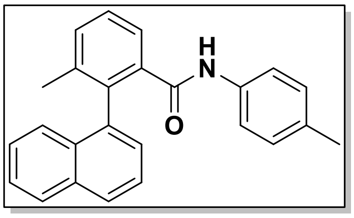

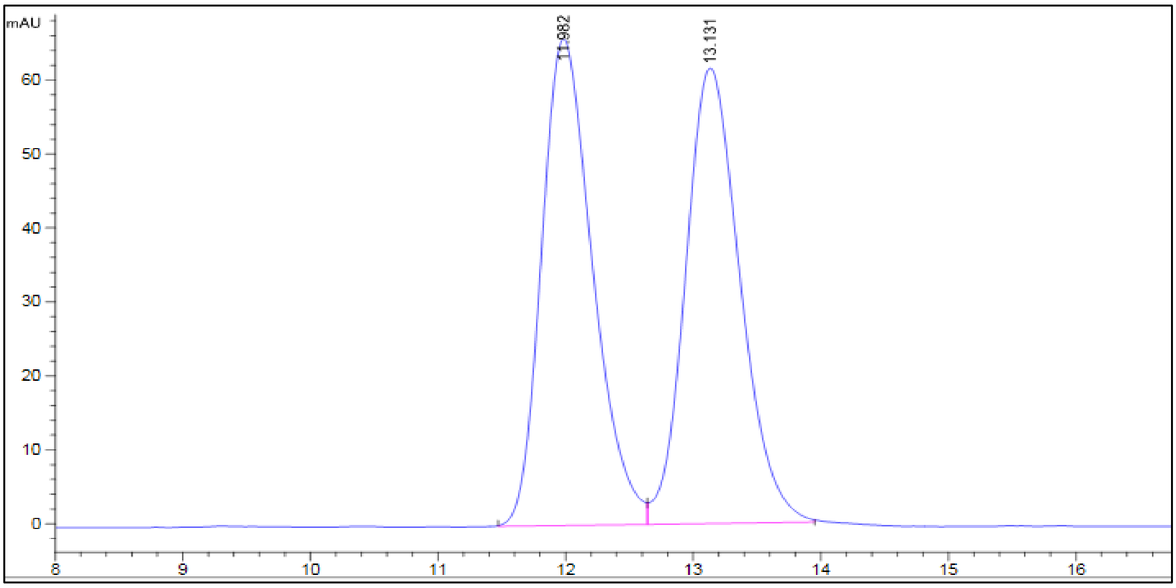

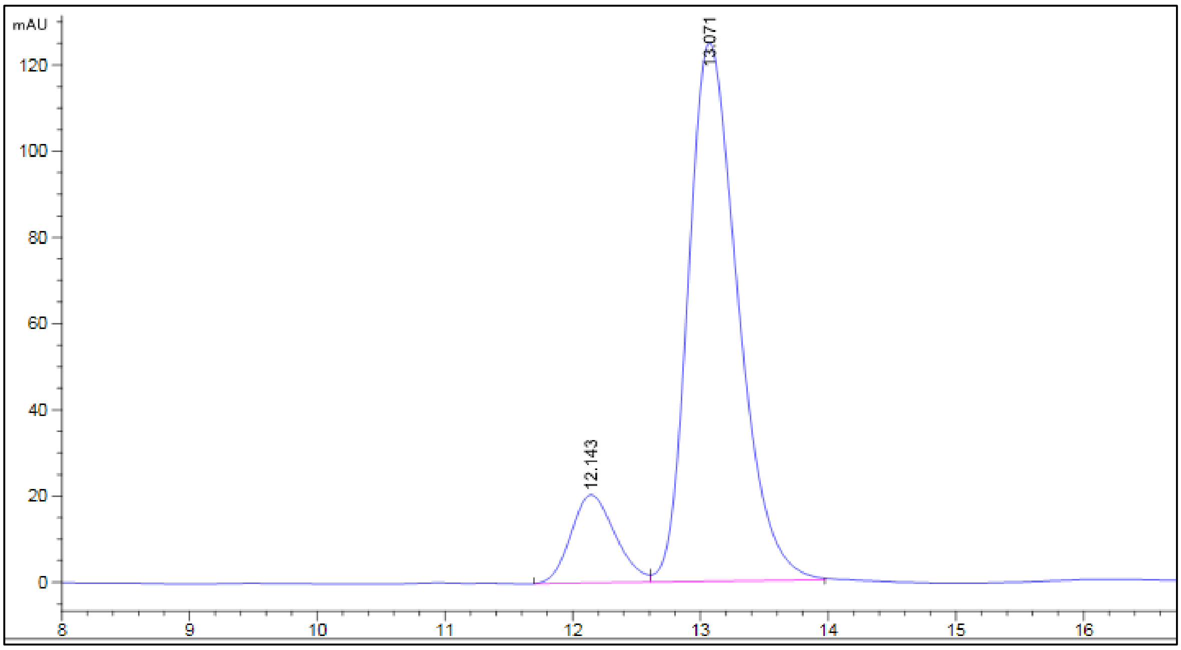

Entry #	RetTime [min]	Width [min]	Area [mAU*s]	Height [mAU]	Area %
1	12.143	0.3743	489.74542	20.38763	12.6879
2	13.071	0.4168	3370.20068	124.92123	87.3121

HPLC Spectra of 3-methyl-2-(naphthalen-1-yl)-*N*-(2-(trifluoromethyl)phenyl)benzamide (3k)

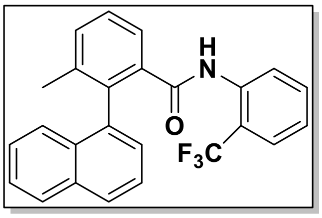

(The top one is racemic, and the bottom one is chiral)

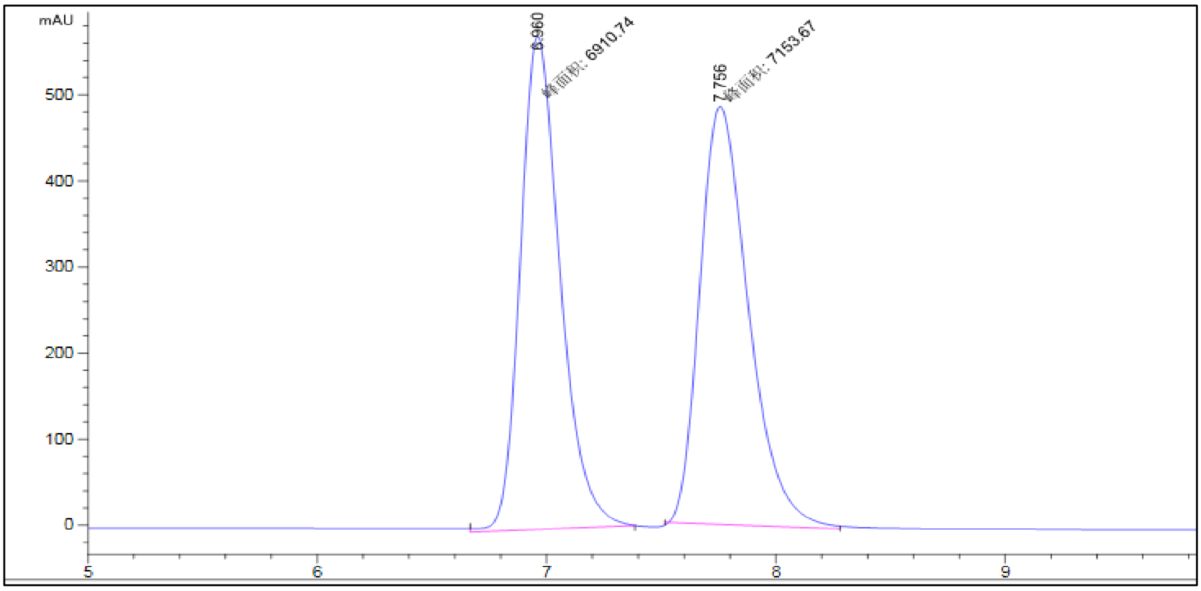

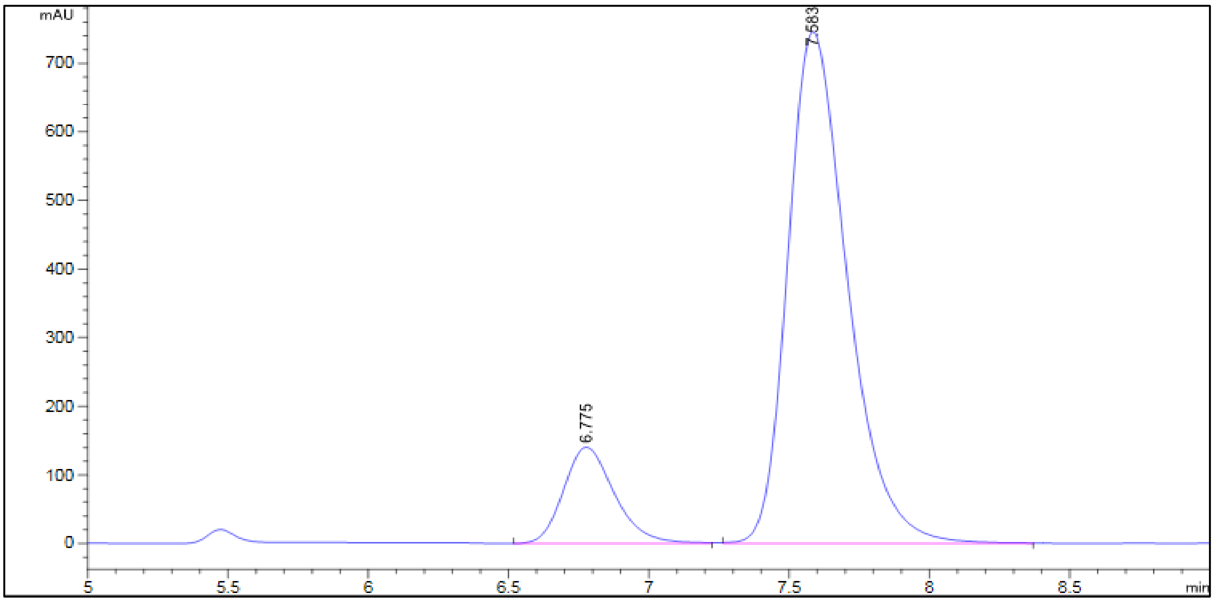

Entry #	RetTime [min]	Width [min]	Area [mAU*s]	Height [mAU]	Area %
1	6.775	0.1954	1775.48633	140.49475	13.8597
2	7.583	0.2266	1.103494	745.82605	86.1403

HPLC Spectra of 3-methyl-2-(naphthalen-1-yl)-*N*-(3-(trifluoromethyl)phenyl)benzamide (3l)

(The top one is racemic, and the bottom one is chiral)

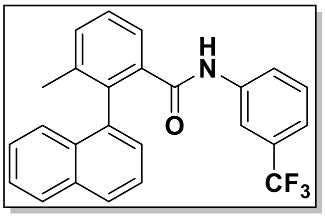

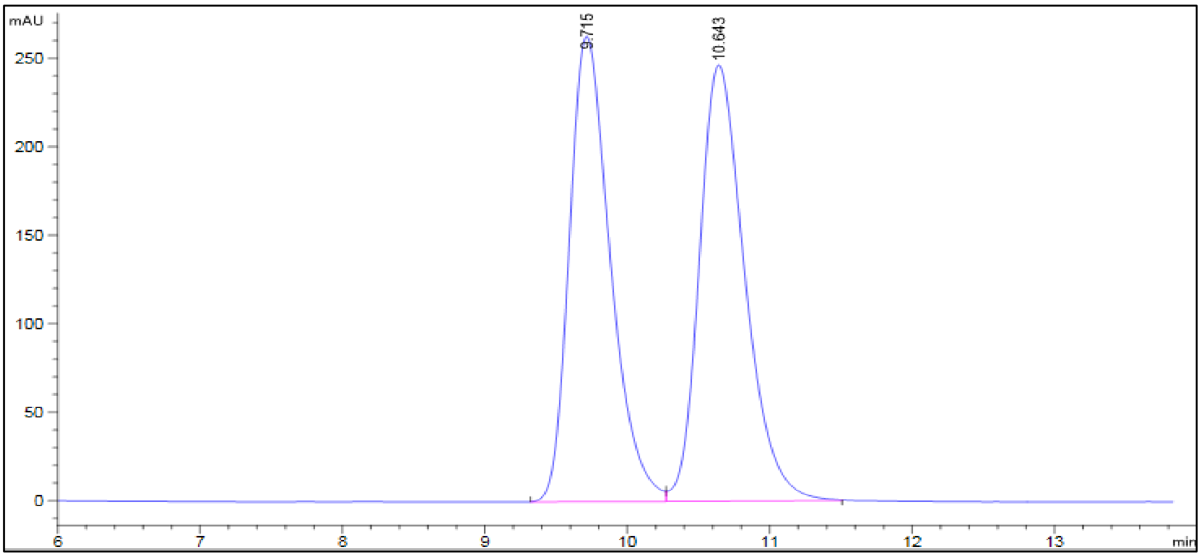

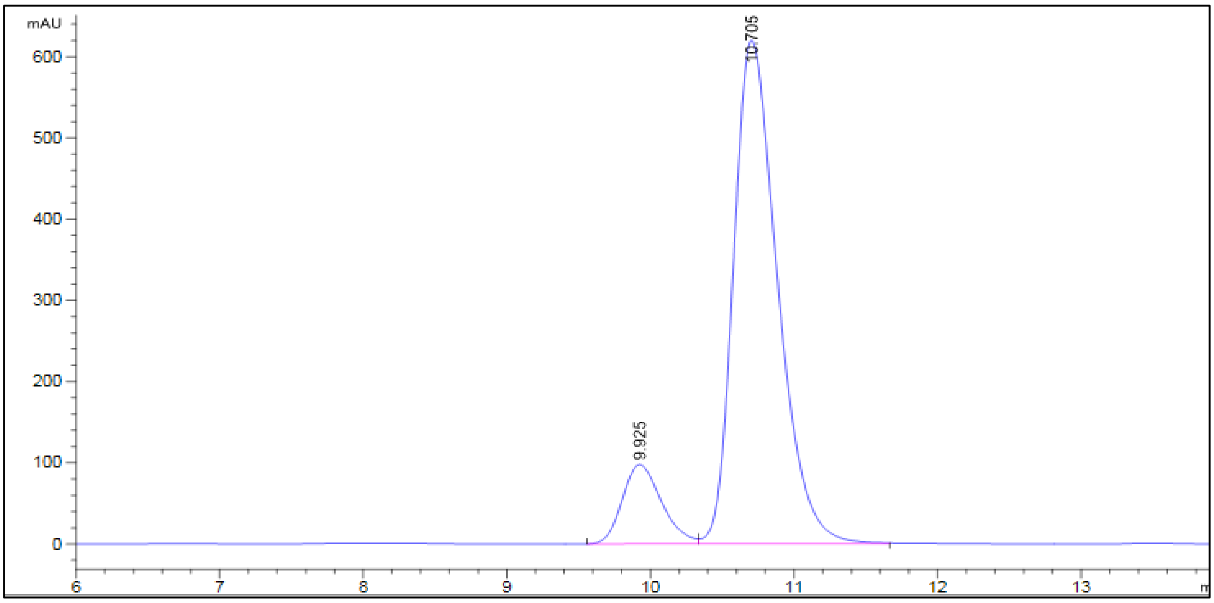

Entry #	RetTime [min]	Width [min]	Area [mAU*s]	Height [mAU]	Area %
1	9.925	0.2932	1850.31958	97.59919	12.3212
2	10.705	0.3238	1.31670e4	619.93048	87.6788

HPLC Spectra of 3-methyl-2-(naphthalen-1-yl)-*N*-(4-(trifluoromethyl)phenyl)benzamide (3m)

(The top one is racemic, and the bottom one is chiral)

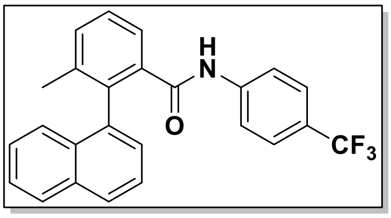

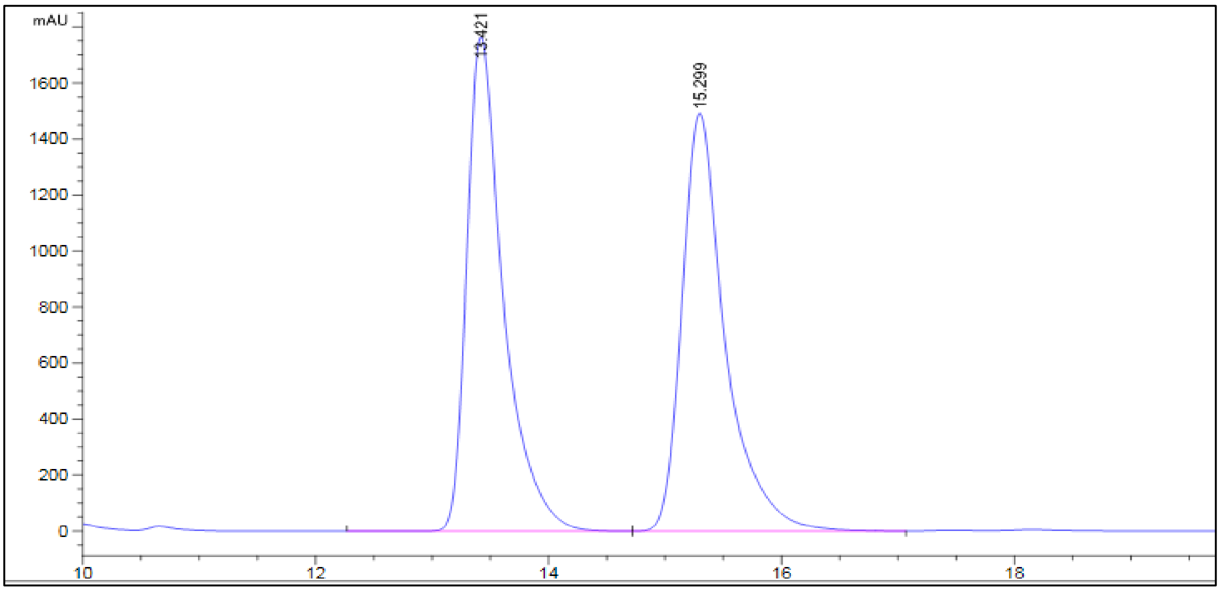

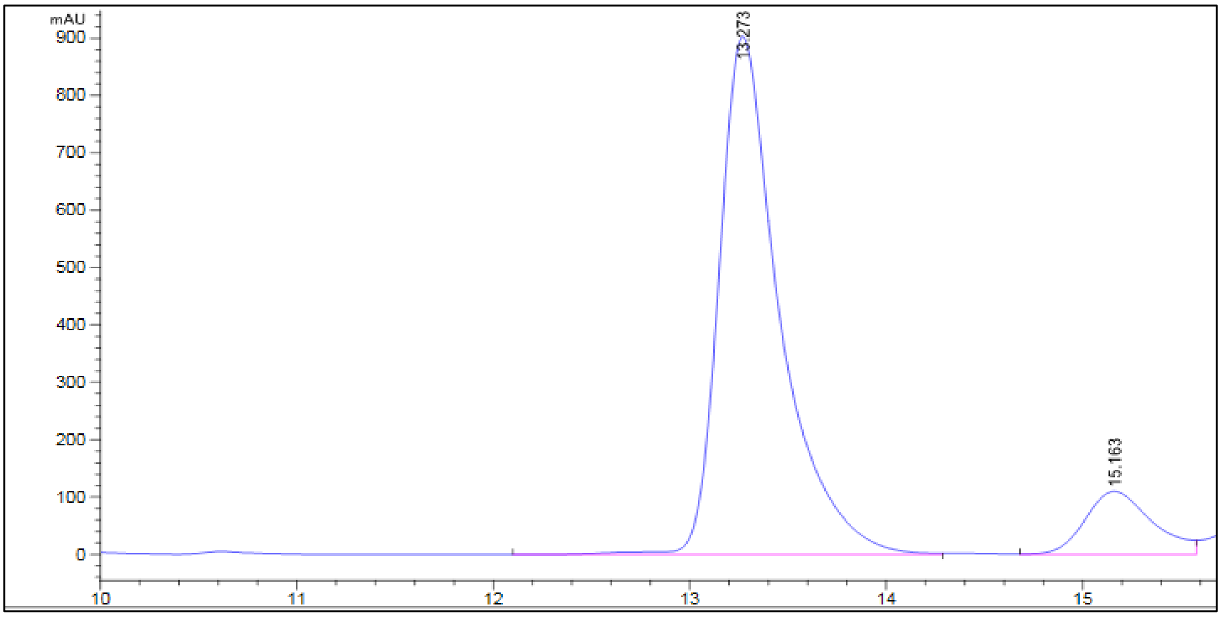

Entry #	RetTime [min]	Width [min]	Area [mAU*s]	Height [mAU]	Area %
1	13.273	0.3141	1.935414	902.77246	88.1106
2	15.163	0.3571	2611.59253	109.95181	11.8894

HPLC Spectra of *N*-(3,5-bis(trifluoromethyl)phenyl)-3-methyl-2-(naphthalen-1-yl)benzamide (3n)

(The top one is racemic, and the bottom one is chiral)

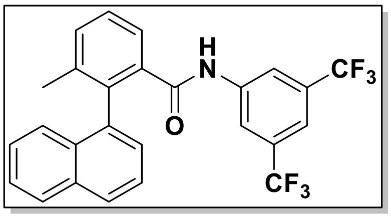

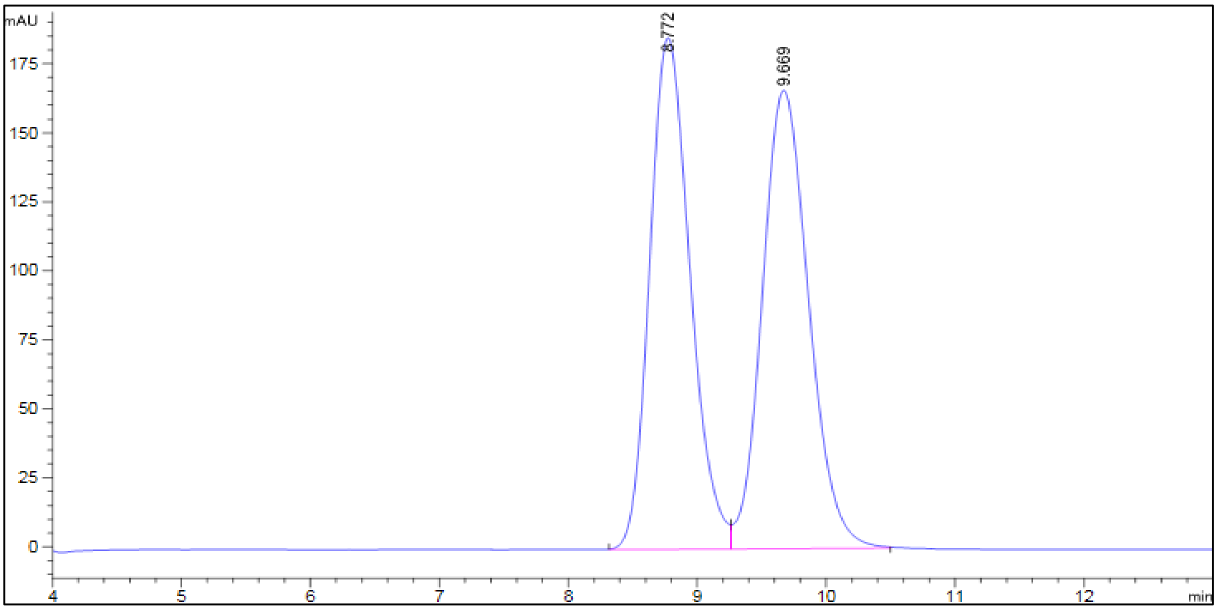

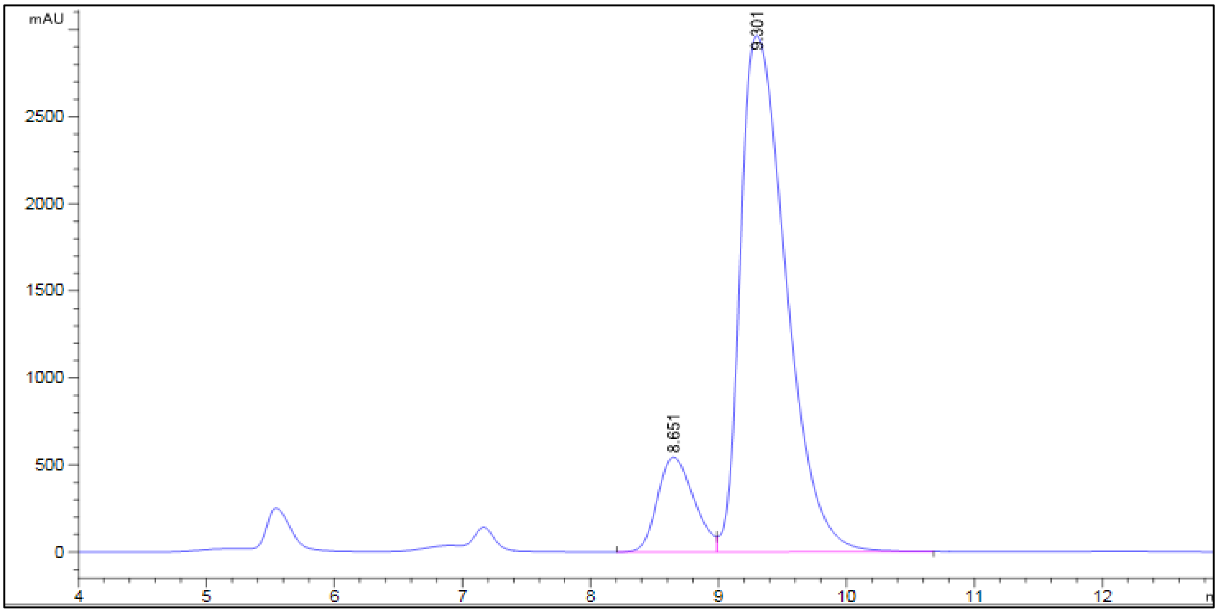

Entry #	RetTime [min]	Width [min]	Area [mAU*s]	Height [mAU]	Area %
1	8.651	0.3095	1.077214	542.77826	12.7904
2	9.301	0.3852	7.34478e	2962.53540	87.2096

HPLC Spectra of ethyl 4-(3-methyl-2-(naphthalen-1-yl)benzamido)benzoate (3o)

(The top one is racemic, and the bottom one is chiral)

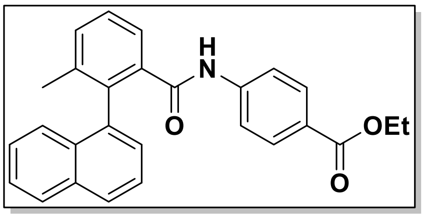

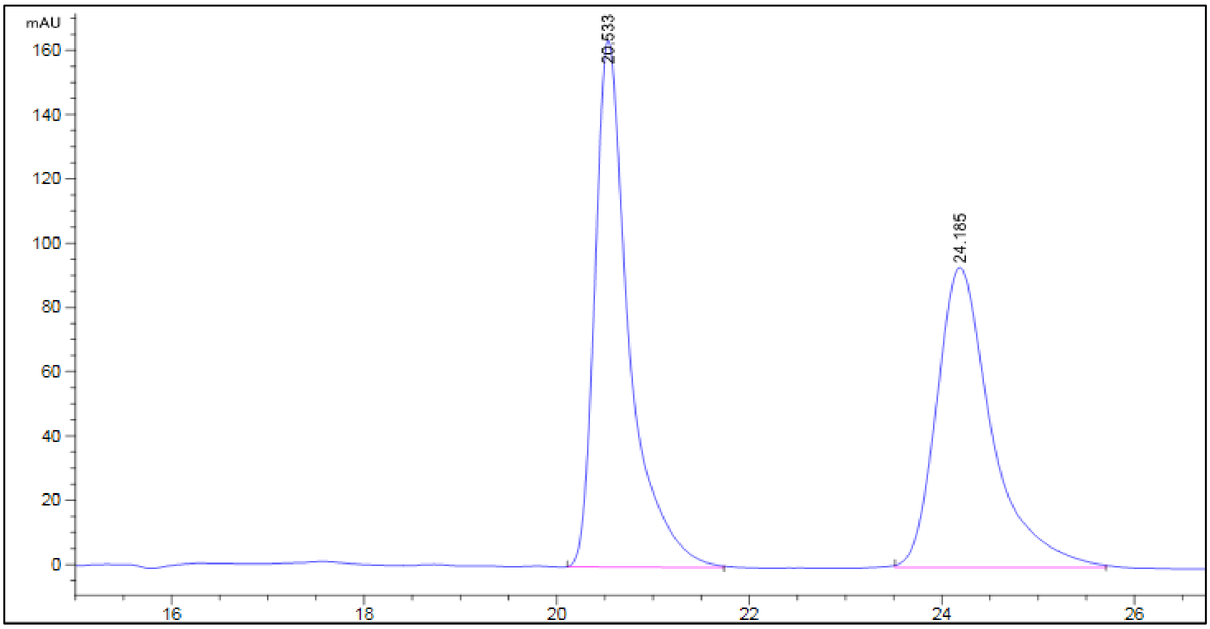

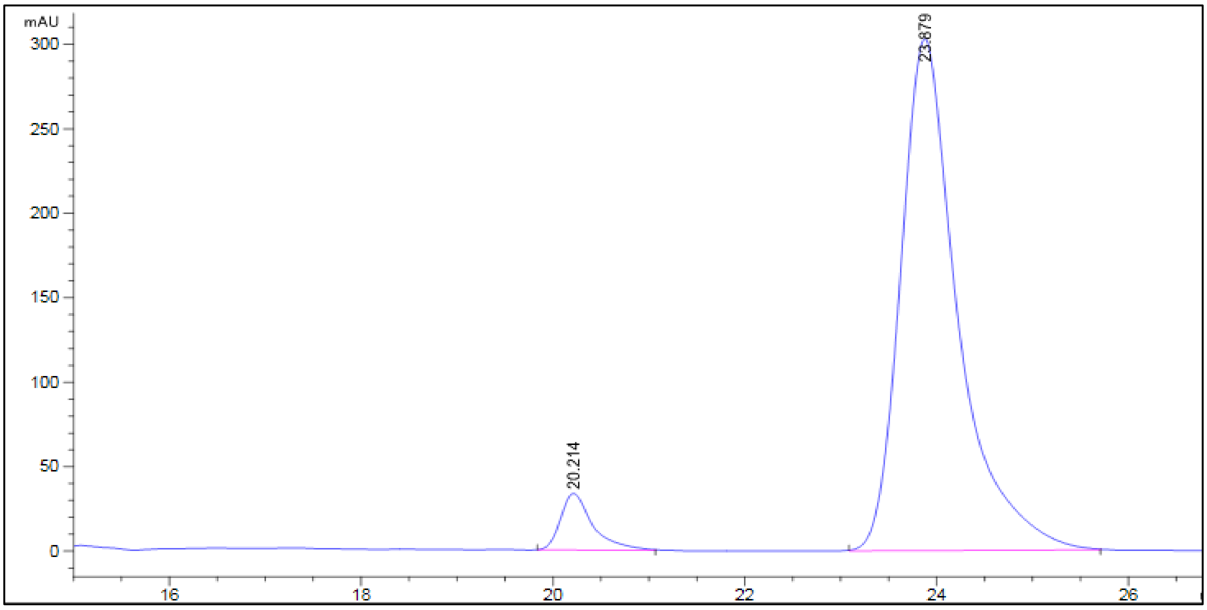

Entry #	RetTime [min]	Width [min]	Area [mAU*s]	Height [mAU]	Area %
1	20.214	0.3471	779.74506	33.31474	5.8500
2	23.879	0.6114	1.25493e4	302.87259	94.1500

HPLC Spectra of 3-methyl-2-(4-methylnaphthalen-1-yl)-*N*-phenylbenzamide (3p)

(The top one is racemic, and the bottom one is chiral)

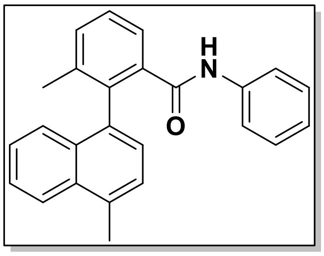

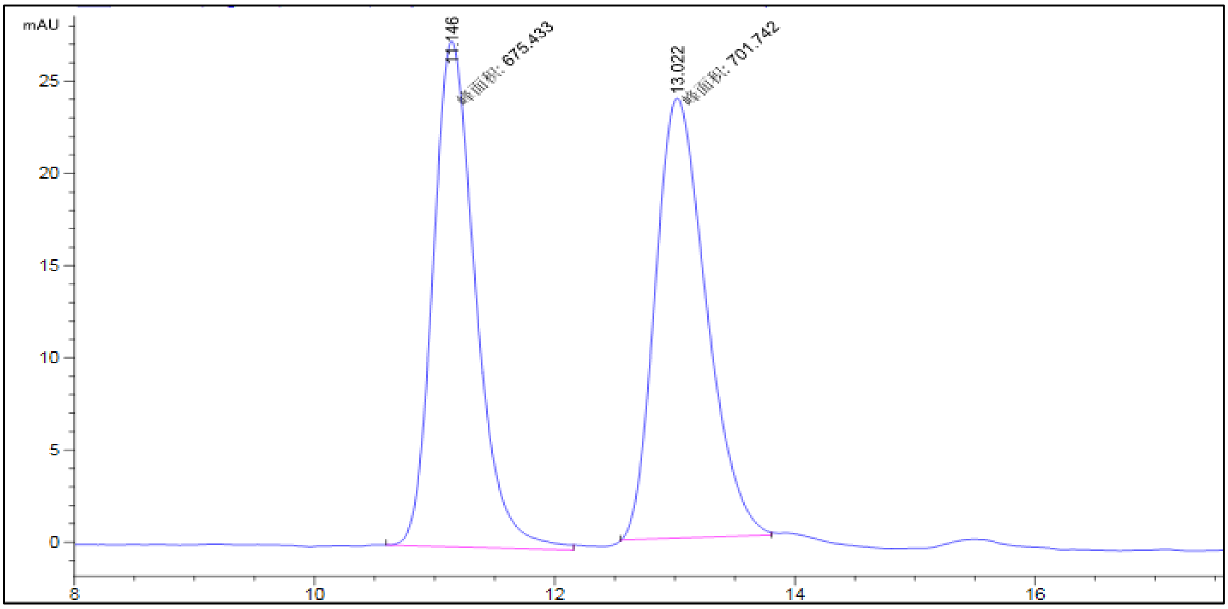

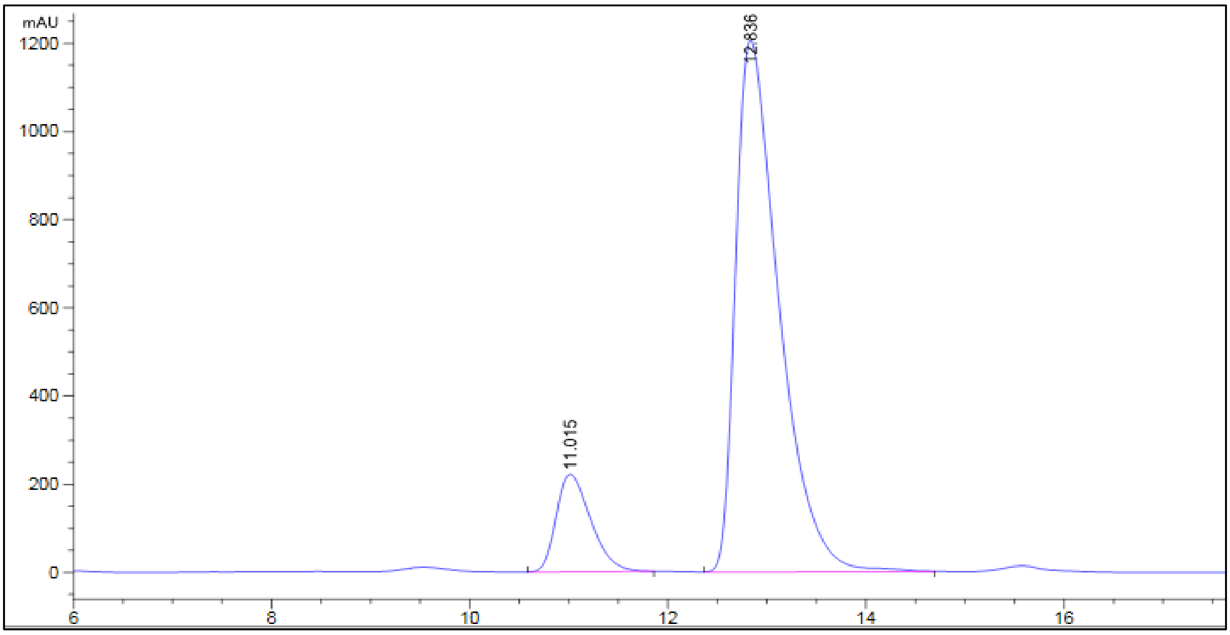

Entry #	RetTime [min]	Width [min]	Area [mAU*s]	Height [mAU]	Area %
1	11.015	0.3730	5353.32275	220.65773	12.7540
2	12.836	0.4582	3.662024	1206.12109	87.2460

HPLC Spectra of 4-methoxy-*N*-phenyl-1-(*o*-tolyl)-2-naphthamide (3q)

(The top one is racemic, and the bottom one is chiral)

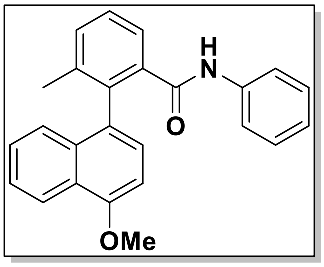

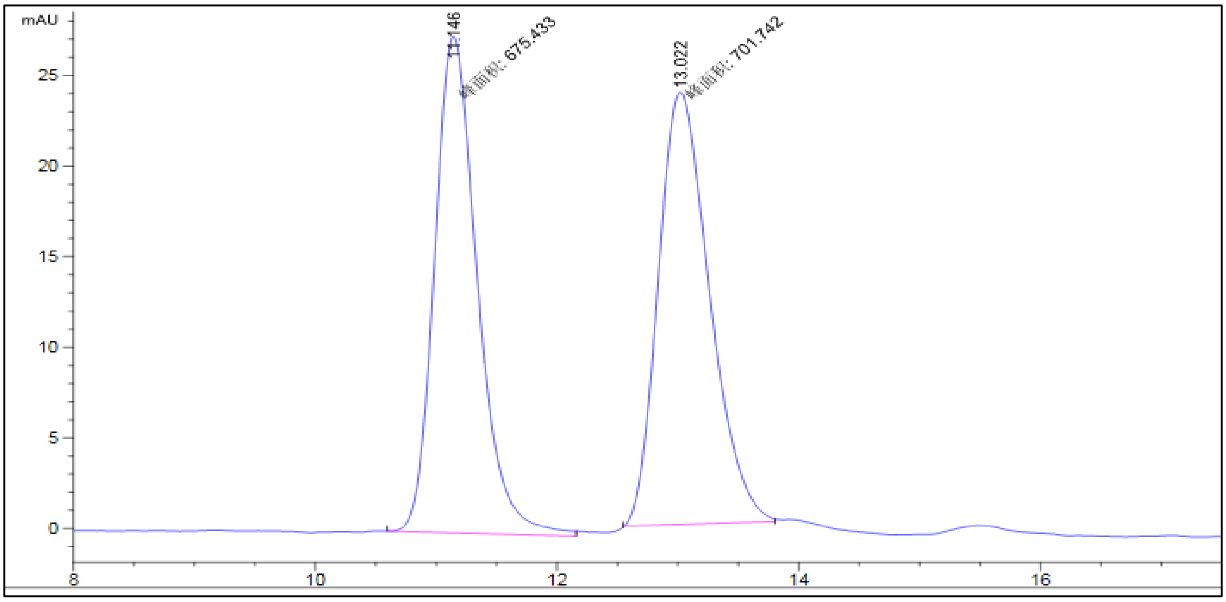

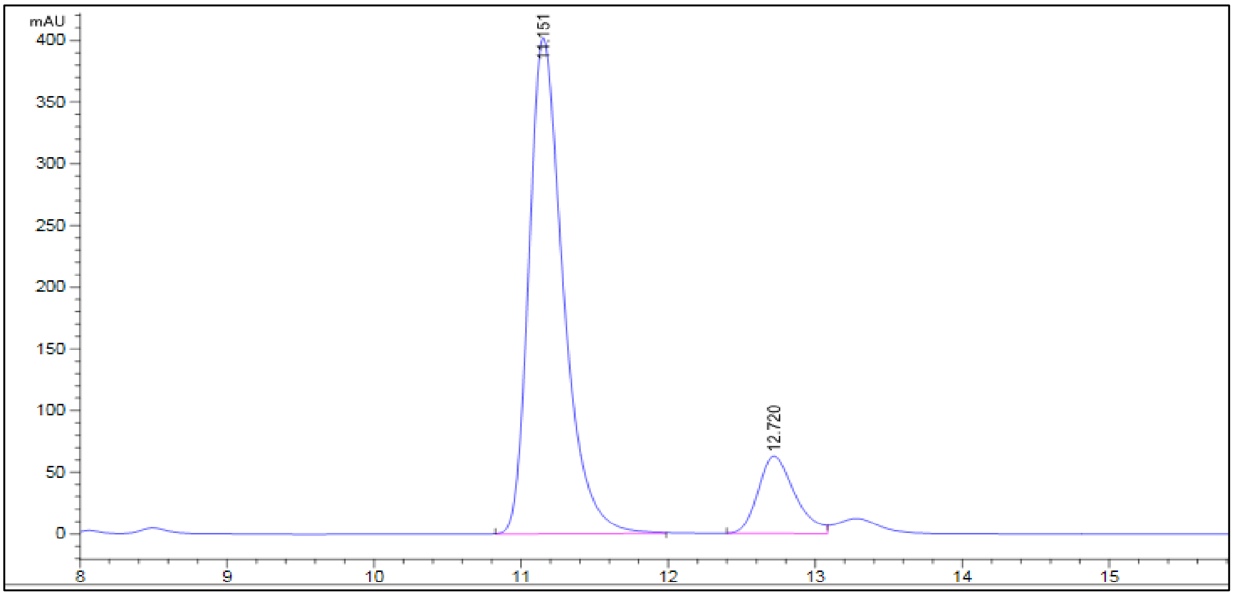

Entry #	RetTime [min]	Width [min]	Area [mAU*s]	Height [mAU]	Area %
1	11.151	0.2449	6510.06934	402.02893	85.7853
2	12.720	0.2634	1078.72424	62.47613	14.2147

HPLC Spectra of 2-(4-(benzyloxy)naphthalen-1-yl)-3-methyl-*N*-phenylbenzamide (3r)

(The top one is racemic, and the bottom one is chiral)

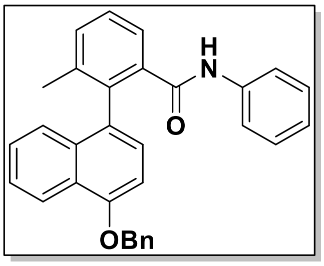

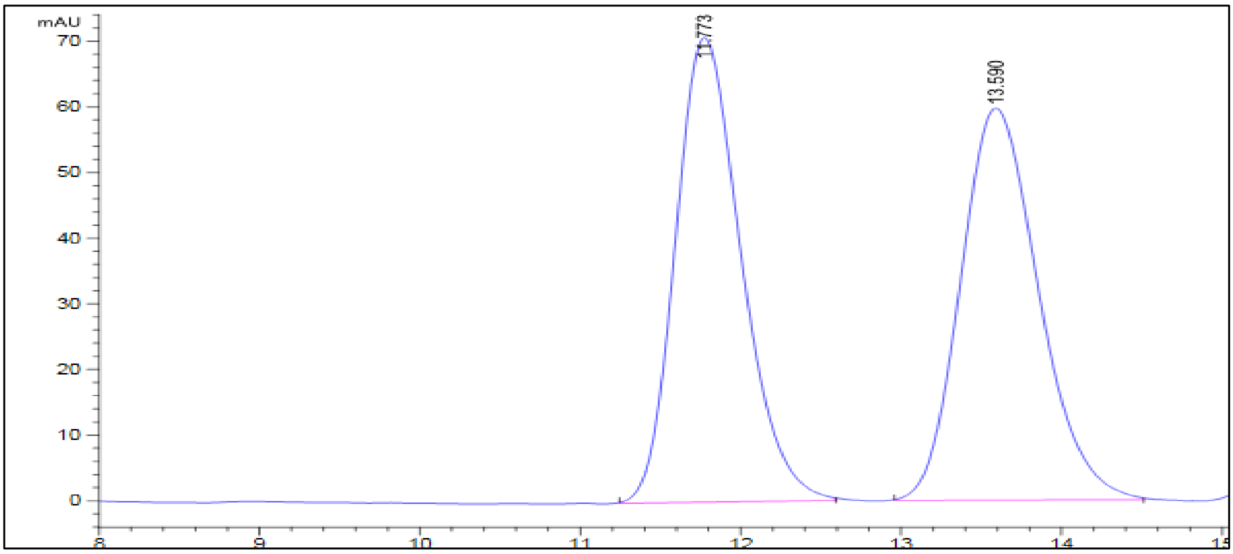

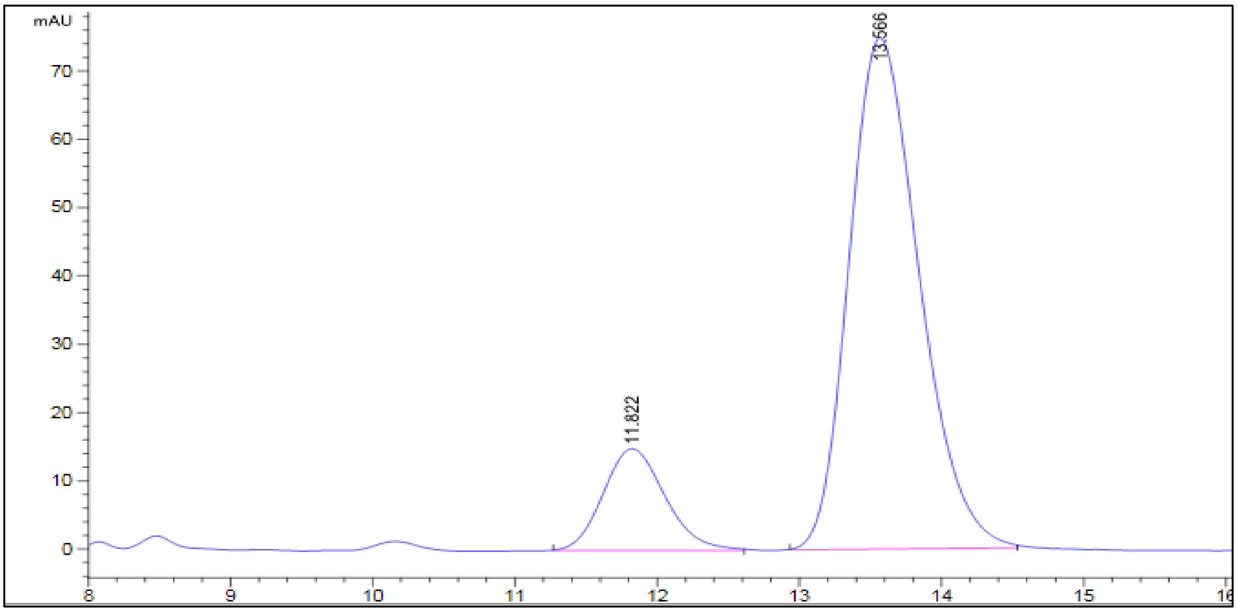

Entry #	RetTime [min]	Width [min]	Area [mAU*s]	Height [mAU]	Area %
1	11.822	0.4706	450.67667	14.91067	14.9458
2	13.566	0.5336	2564.73096	74.88159	85.0542

HPLC Spectra of *N*-isopropyl-3-methyl-2-(naphthalen-1-yl)aniline (5b)

(The top one is racemic, and the bottom one is chiral)

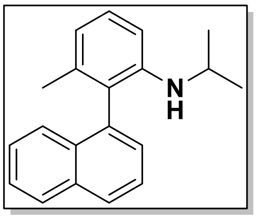

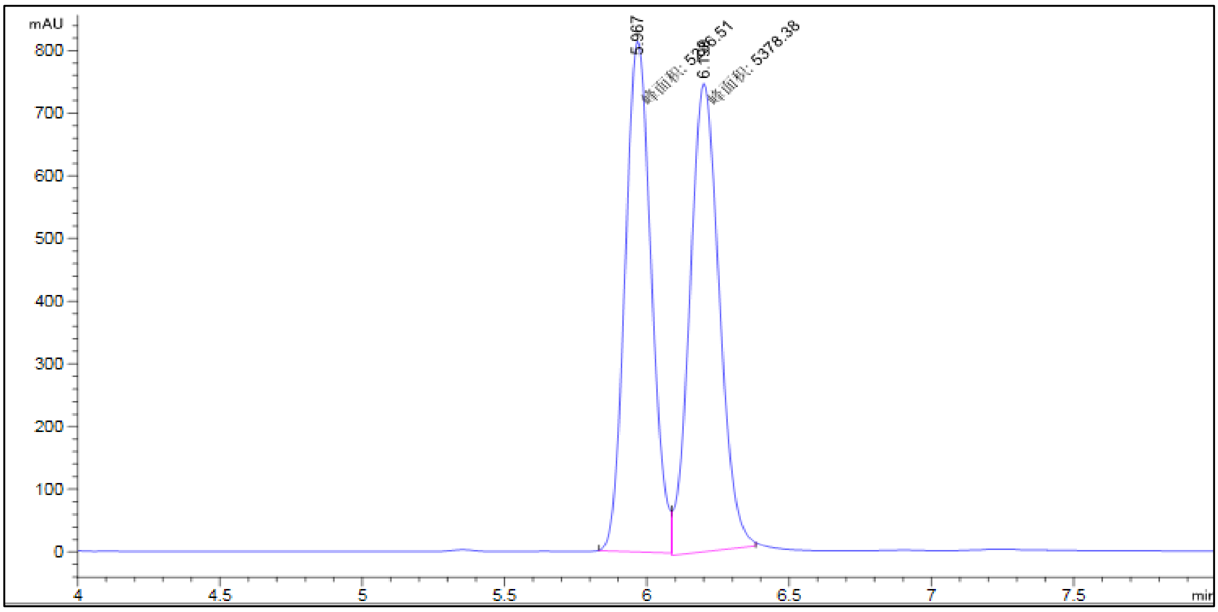

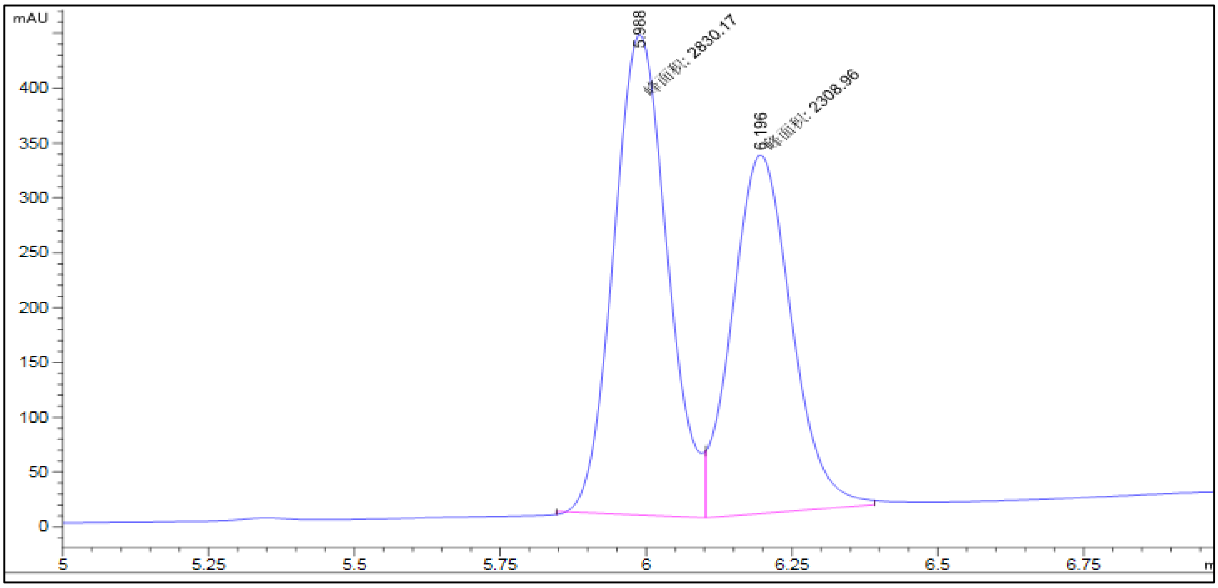

Entry #	RetTime [min]	Width [min]	Area [mAU*s]	Height [mAU]	Area %
1	5.988	0.1075	2830.17041	438.96976	55.0710
2	6.196	0.1174	2308.96436	327.85175	44.9290

HPLC Spectra of *N*-benzyl-3-methyl-2-(naphthalen-1-yl)aniline (5c)

(The top one is racemic, and the bottom one is chiral)

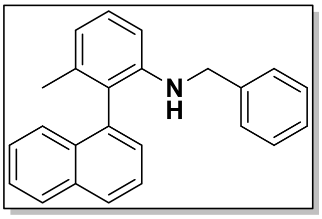

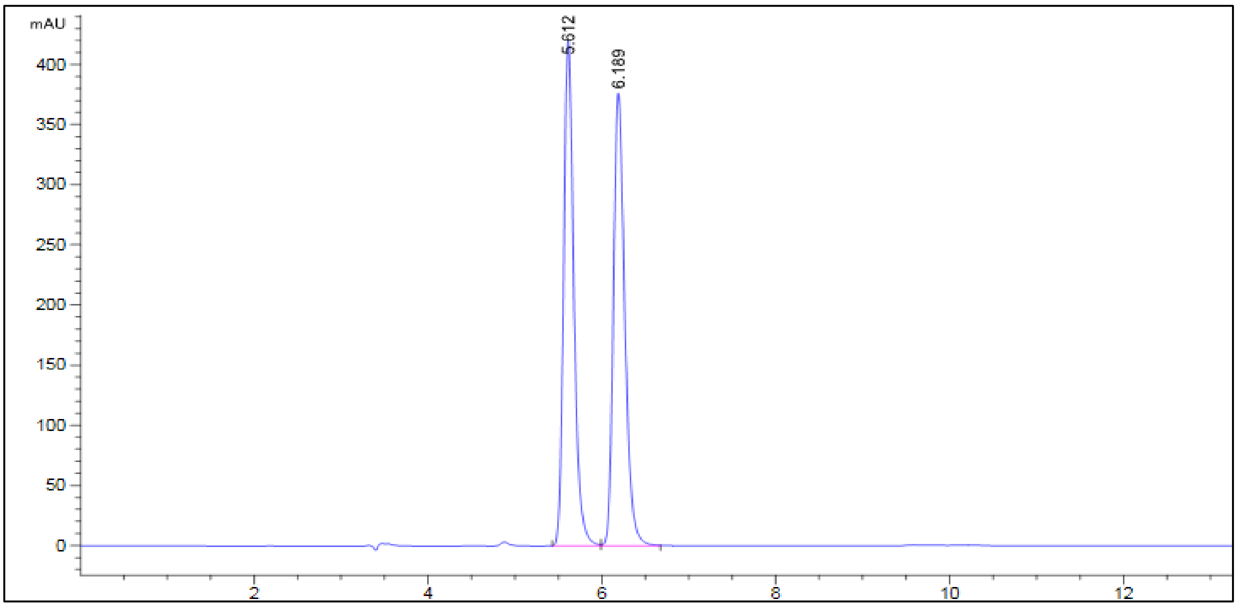

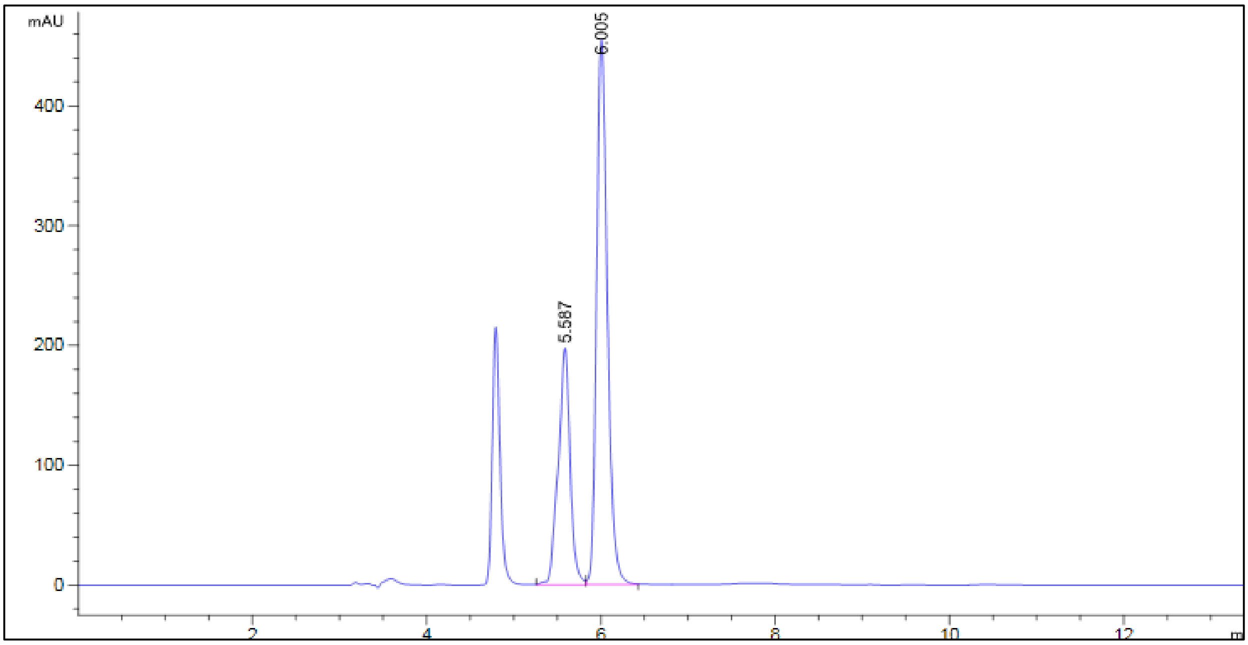

Entry #	RetTime [min]	Width [min]	Area [mAU*s]	Height [mAU]	Area %
1	5.587	0.1368	1878.45886	197.77565	32.4563
2	6.005	0.1321	3909.20239	455.41733	67.5437

HPLC Spectra of *N*-(3-methyl-2-(naphthalen-1-yl)phenyl)benzamide (5d)

(The top one is racemic, and the bottom one is chiral)

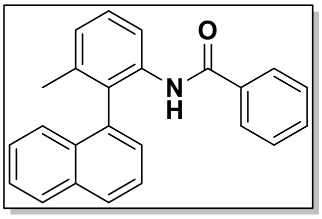

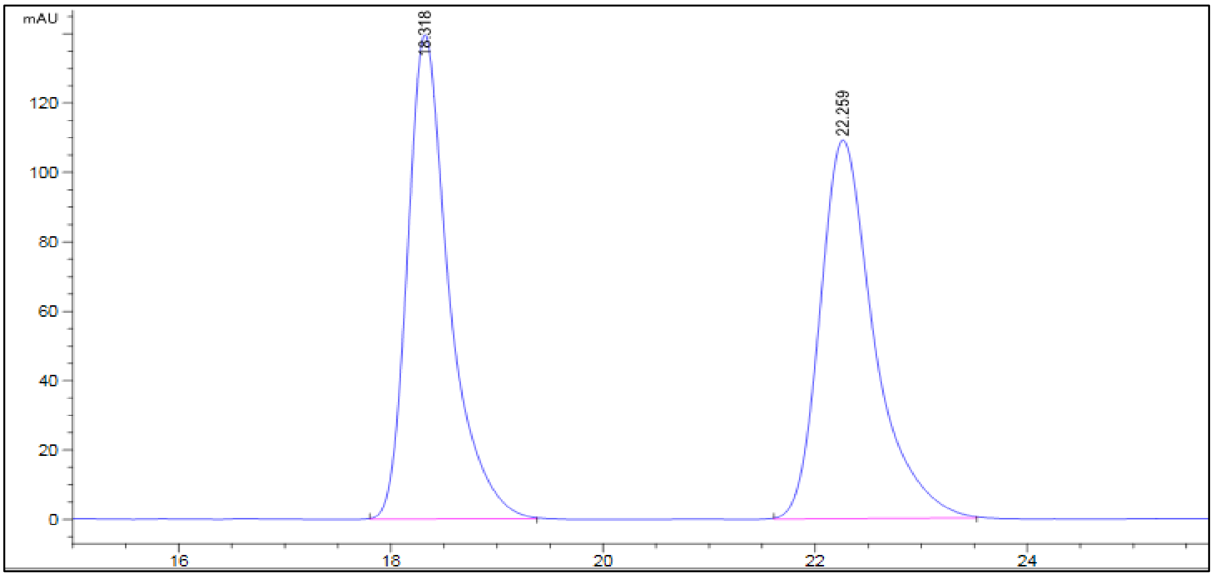

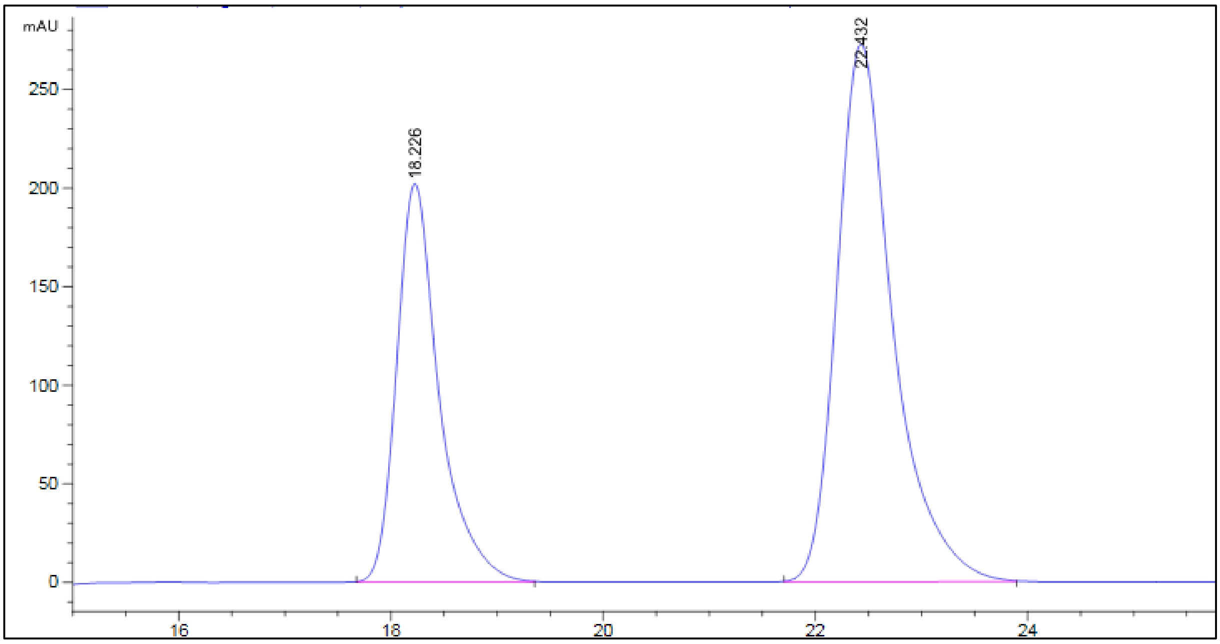

Entry #	RetTime [min]	Width [min]	Area [mAU*s]	Height [mAU]	Area %
1	18.226	0.4180	5650.18506	202.18347	36.0583
2	22.432	0.5526	1.00194e4	272.70181	63.9417

HPLC Spectra of methyl 3-methyl-2-(naphthalen-1-yl)benzoate (5e)

(The top one is racemic, and the bottom one is chiral)

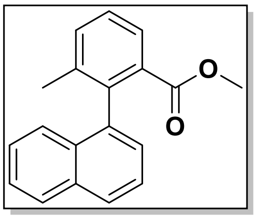

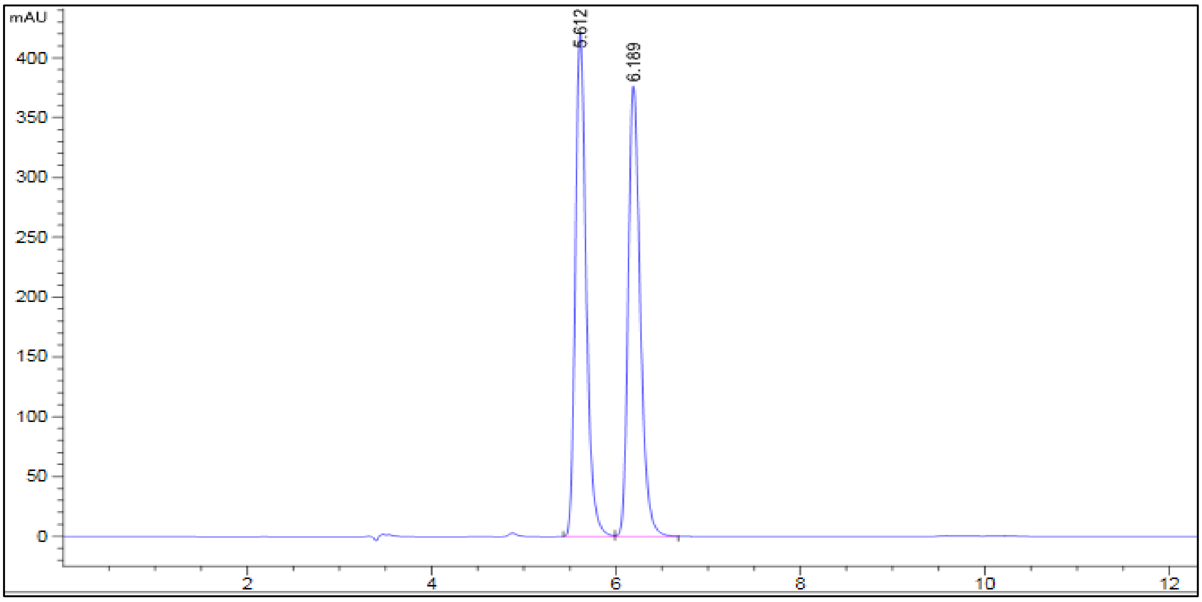

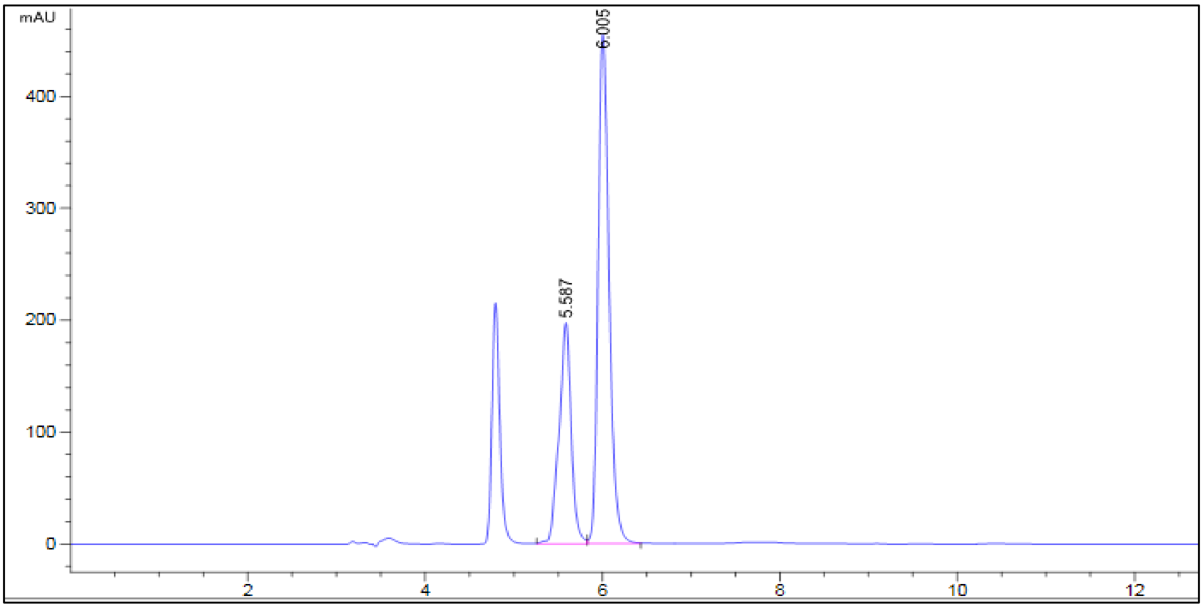

Entry #	RetTime [min]	Width [min]	Area [mAU*s]	Height [mAU]	Area %
1	5.587	0.1368	1878.45886	197.77565	32.4563
2	6.005	0.1321	3909.20239	455.41733	67.5437

HPLC Spectra of *tert*-butyl 3-methyl-2-(naphthalen-1-yl)benzoate (5f)

(The top one is racemic, and the bottom one is chiral)

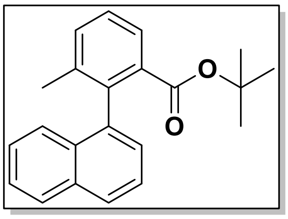

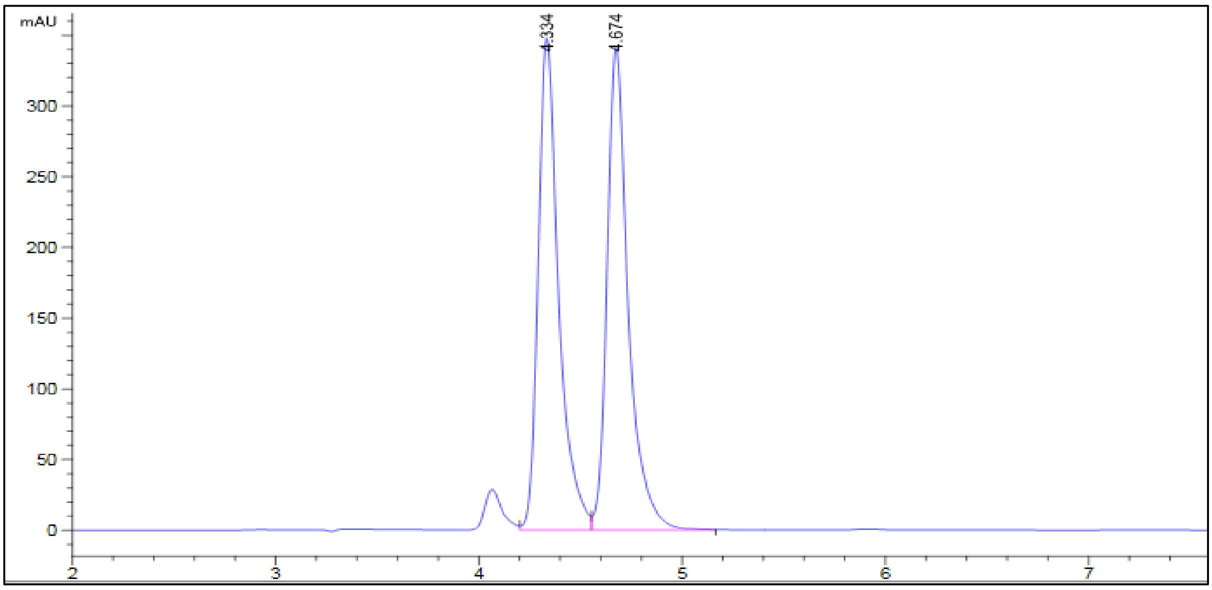

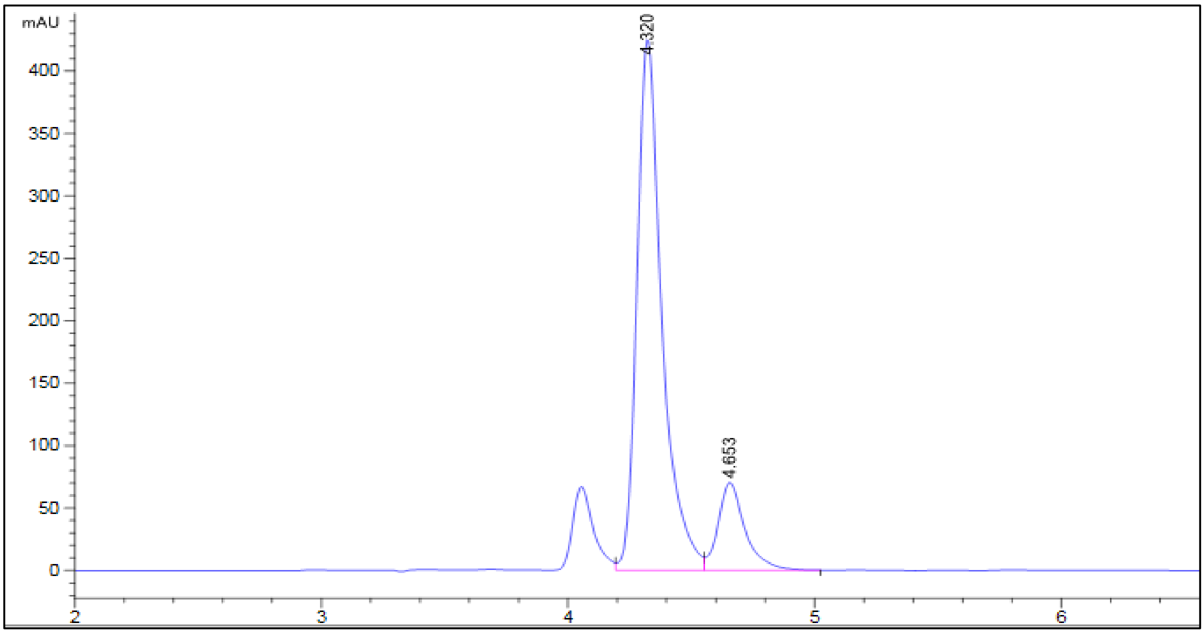

Entry #	RetTime [min]	Width [min]	Area [mAU*s]	Height [mAU]	Area %
1	4.320	0.1044	3004.78467	425.55936	84.9588
2	4.653	0.1124	531.96991	70.23435	15.0412

HPLC Spectra of 1-(2-methyl-6-nitrophenyl)naphthalene (5g)

(The top one is racemic, and the bottom one is chiral)

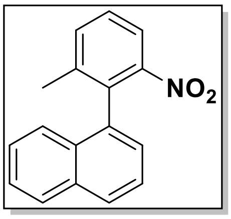

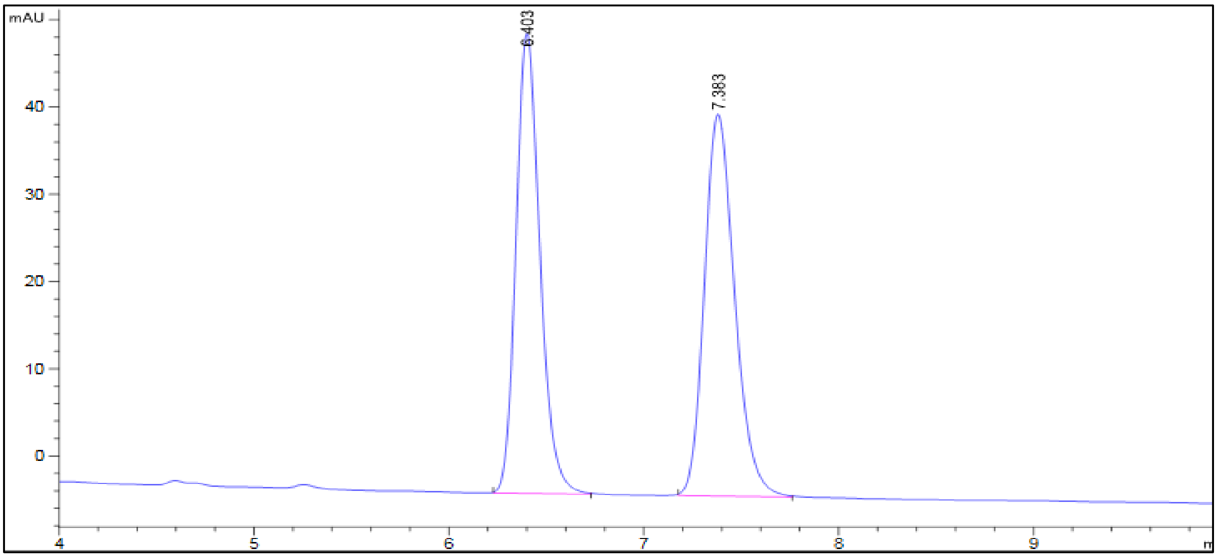

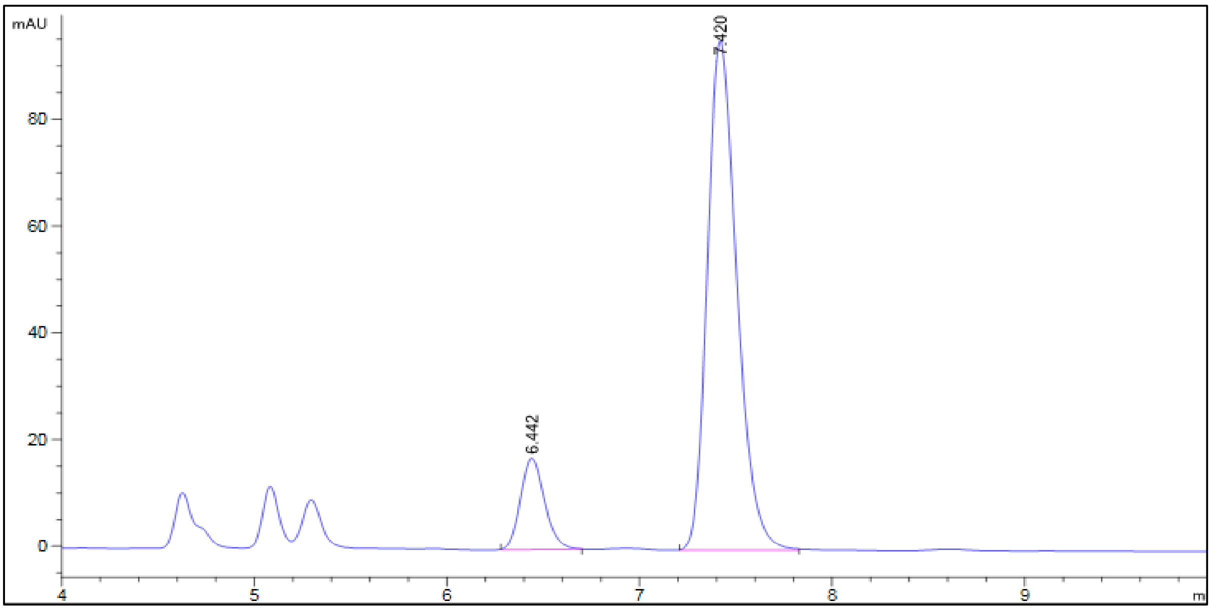

Entry #	RetTime [min]	Width [min]	Area [mAU*s]	Height [mAU]	Area %
1	6.442	0.1358	149.11588	17.09181	12.7700
2	7.420	0.1625	1018.58496	95.43012	87.2300

HPLC Spectra of 1-methyl-4-(2-methyl-6-nitrophenyl)naphthalene (5h)

(The top one is racemic, and the bottom one is chiral)

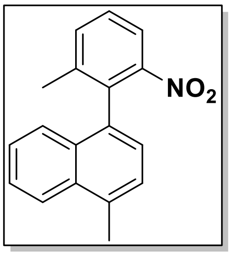

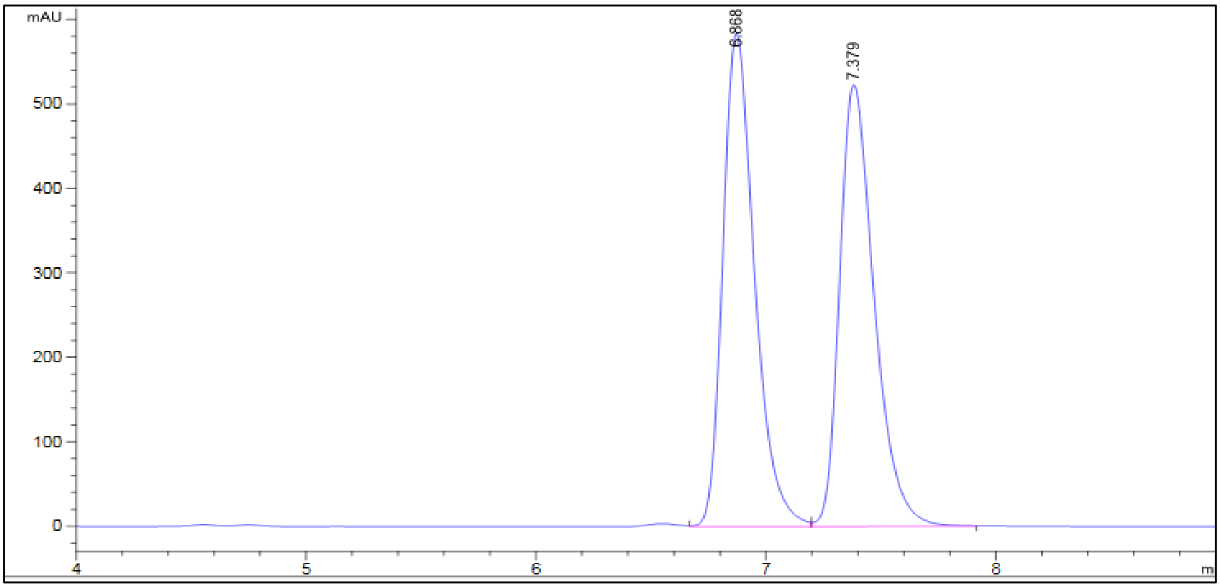

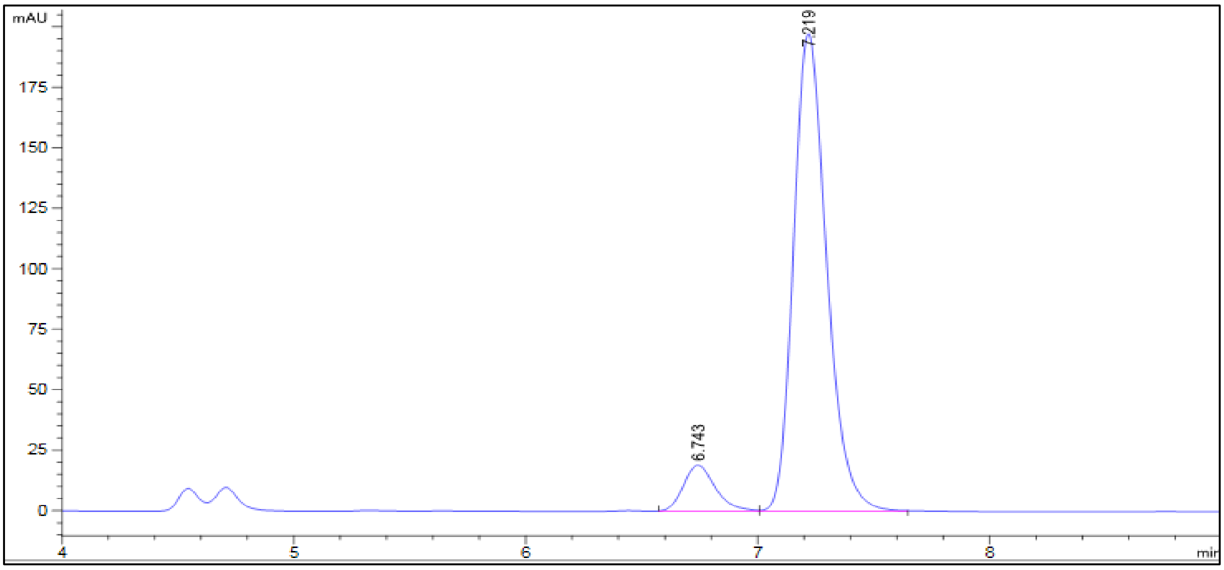

Entry #	RetTime [min]	Width [min]	Area [mAU*s]	Height [mAU]	Area %
1	6.743	0.1454	180.80318	18.94144	8.4327
2	7.219	0.1541	1963.27869	197.36748	91.5673

HPLC Spectra of 1-methoxy-4-(2-methyl-6-nitrophenyl)naphthalene (5i)

(The top one is racemic, and the bottom one is chiral)

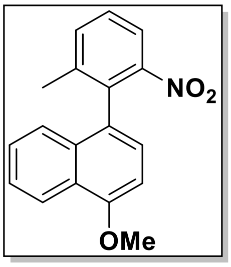

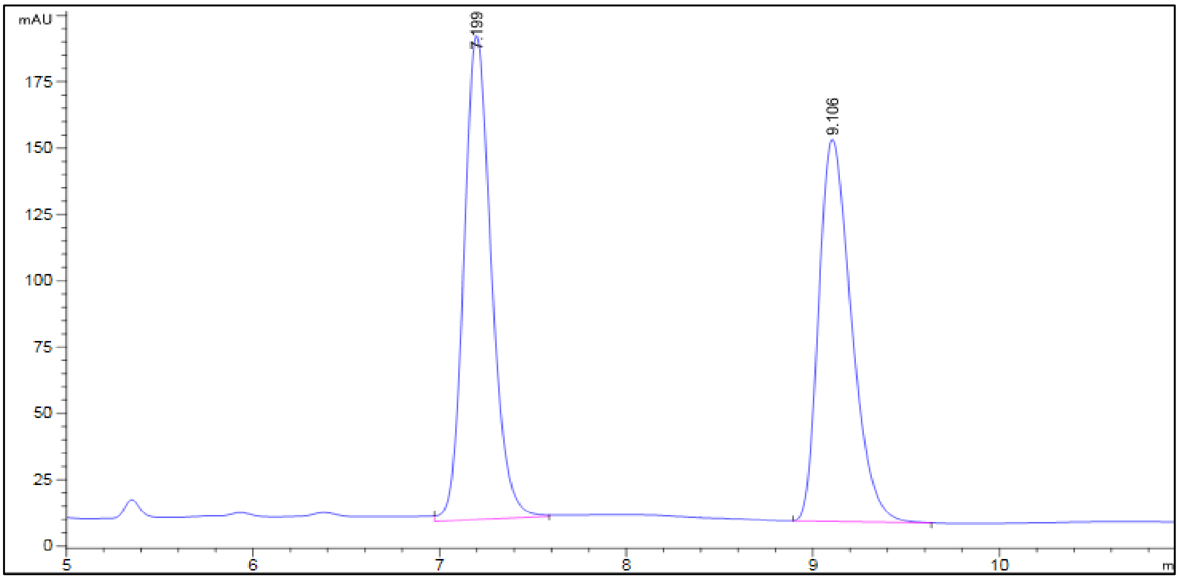

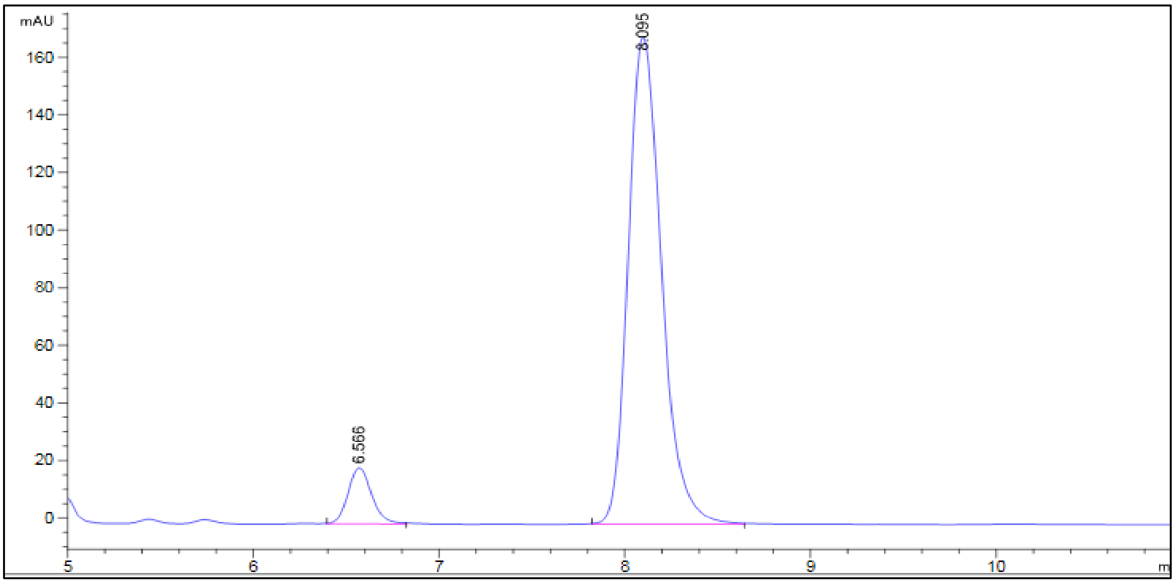

Entry #	RetTime [min]	Width [min]	Area [mAU*s]	Height [mAU]	Area %
1	6.566	0.1351	171.92560	19.46371	7.5375
2	8.095	0.1955	2109.00220	169.15469	92.4625

HPLC Spectra of 3-methoxy-2-(naphthalen-1-yl)-*N*-phenylbenzamide (3s)

(The top one is racemic, and the bottom one is chiral)

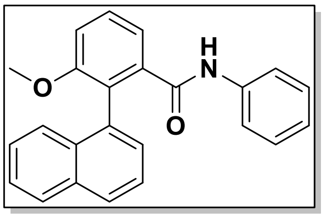

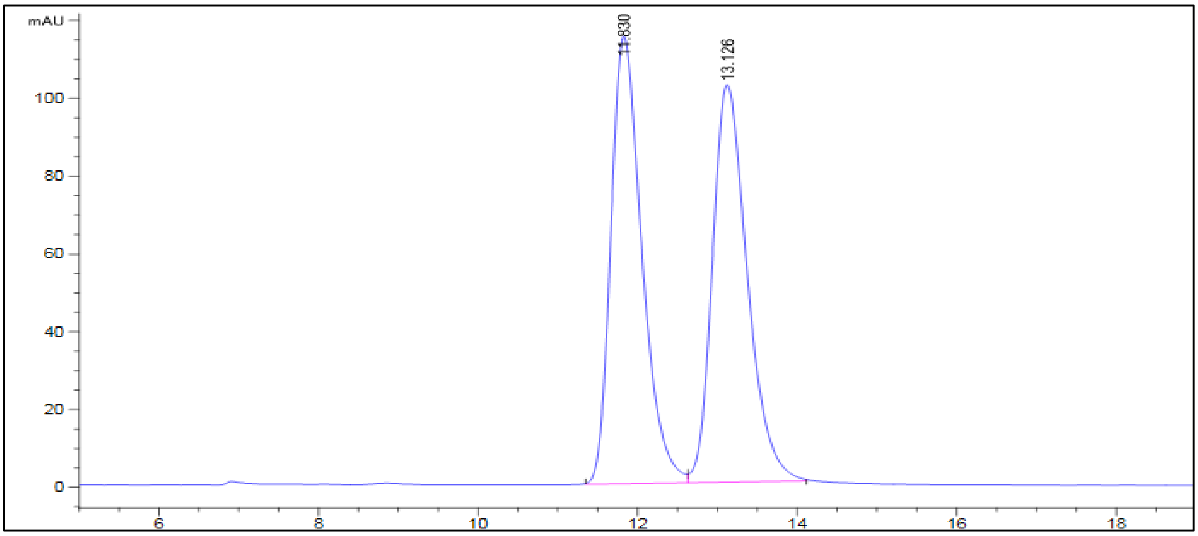

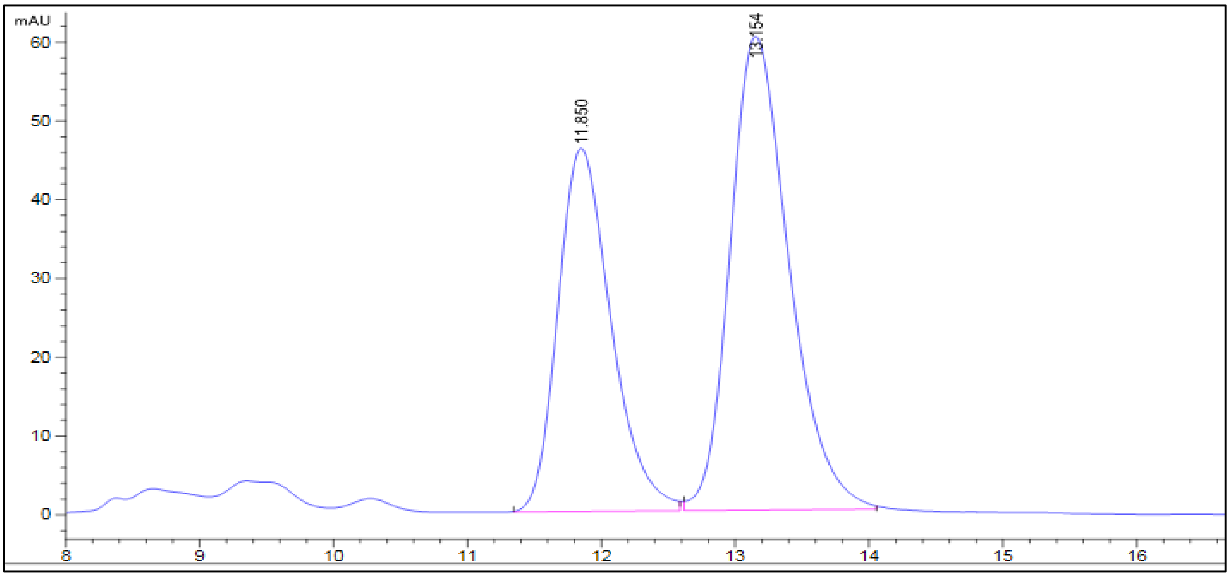

Entry #	RetTime [min]	Width [min]	Area [mAU*s]	Height [mAU]	Area %
1	11.850	0.4242	1272.67139	46.08847	40.9130
2	13.154	0.4668	1838.00745	60.09356	59.0870

HPLC Spectra of 3-(benzyloxy)-2-(naphthalen-1-yl)-*N*-phenylbenzamide (3t)

(The top one is racemic, and the bottom one is chiral)

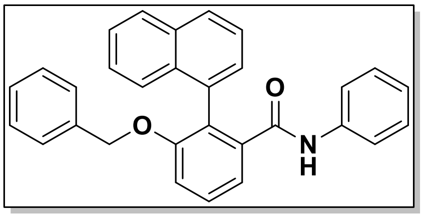

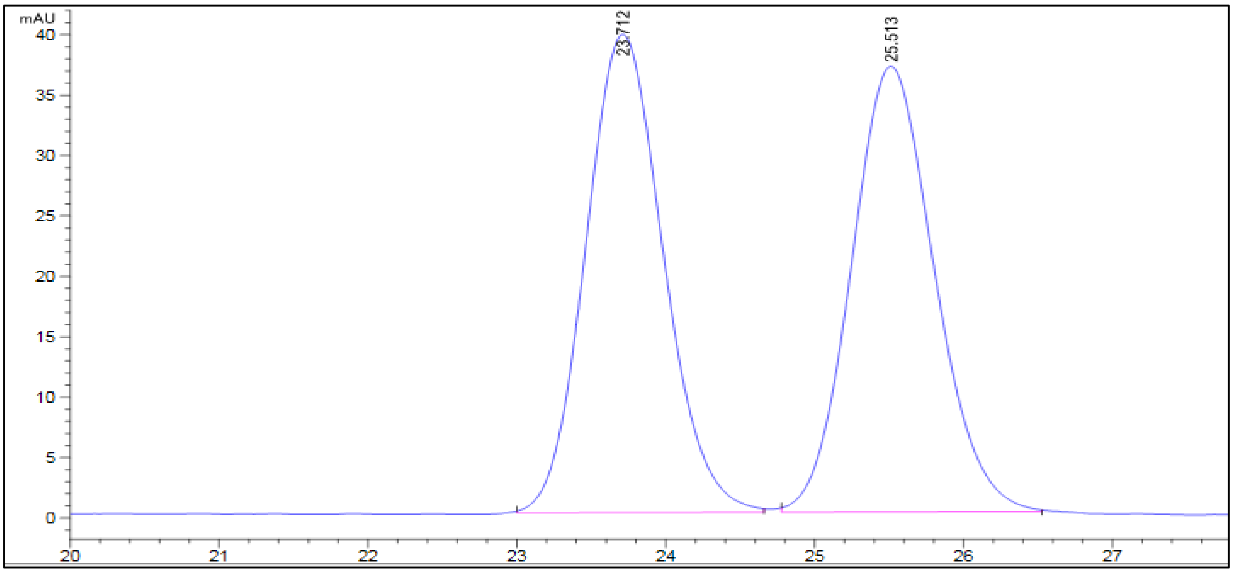

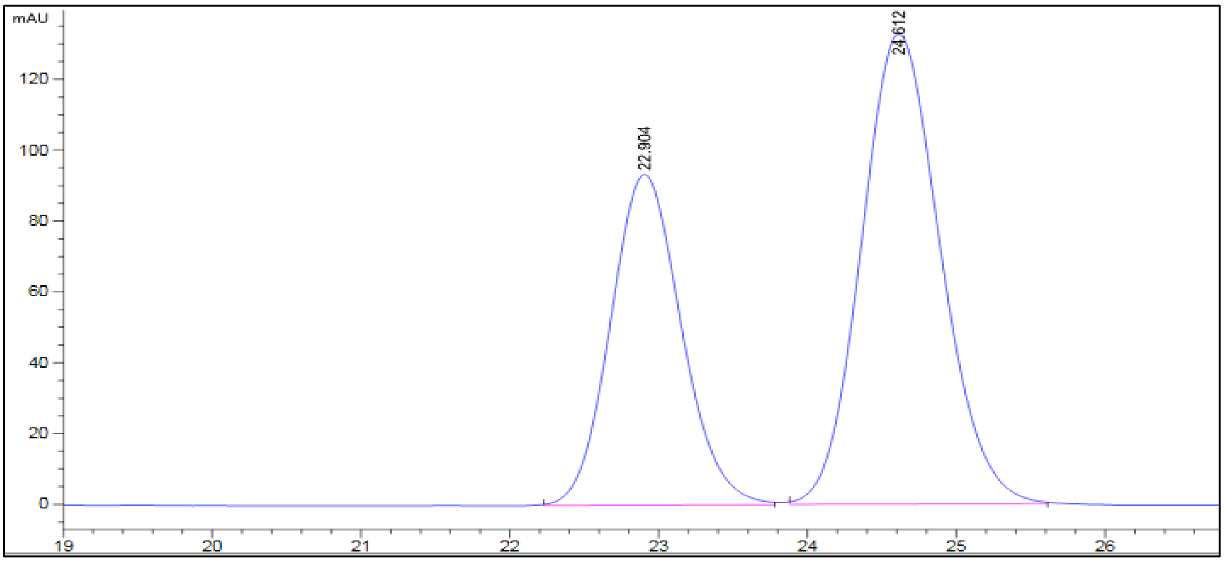

Entry #	RetTime [min]	Width [min]	Area [mAU*s]	Height [mAU]	Area %
1	22.904	0.5138	3086.27539	93.33580	38.6796
2	24.612	0.5700	4892.80029	132.73547	61.3204

HPLC Spectra of 2',6-dimethyl-*N*-phenyl-[1,1'-biphenyl]-2-carboxamide (3u)

(The top one is racemic, and the bottom one is chiral)

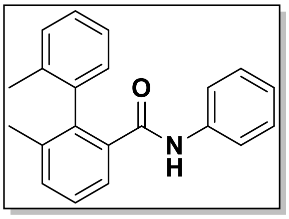

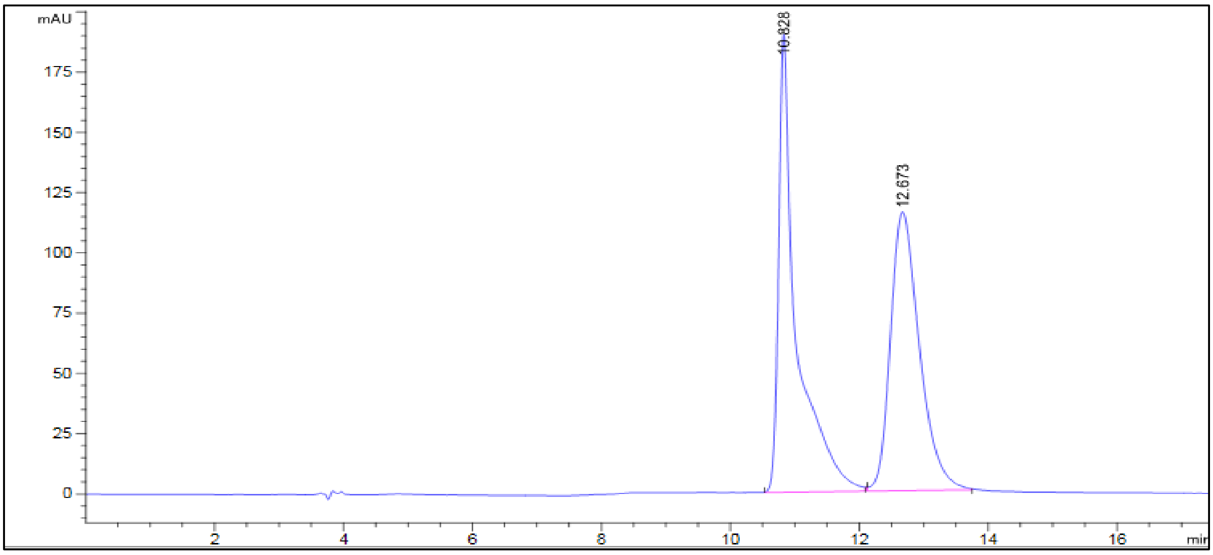

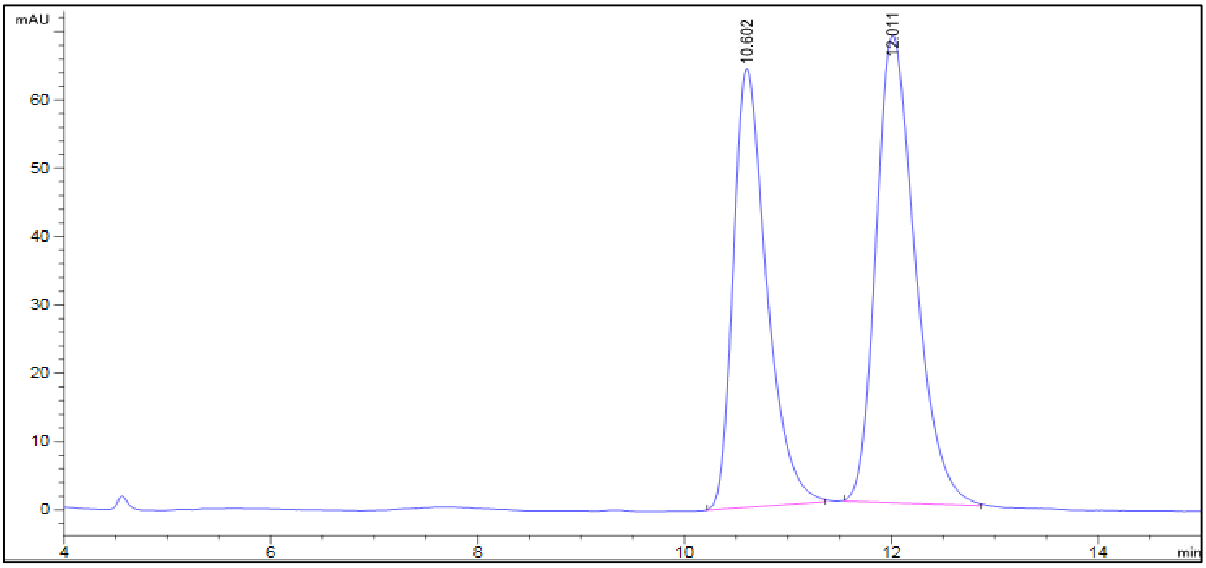

Entry #	RetTime [min]	Width [min]	Area [mAU*s]	Height [mAU]	Area %
1	10.602	0.3395	1439.70154	64.22195	44.3652
2	12.011	0.4019	1805.41638	68.40852	55.6348

HPLC Spectra of 2'-methoxy-6-methyl-*N*-phenyl-[1,1'-biphenyl]-2-carboxamide (3v)

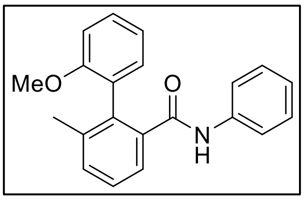

(The top one is racemic, and the bottom one is chiral)

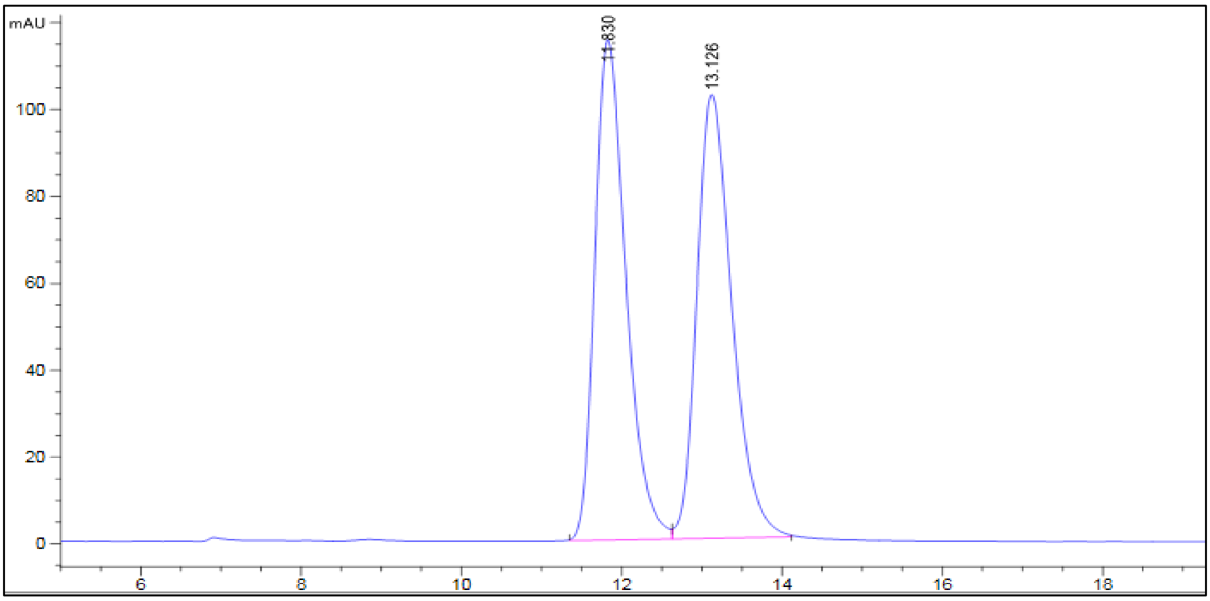

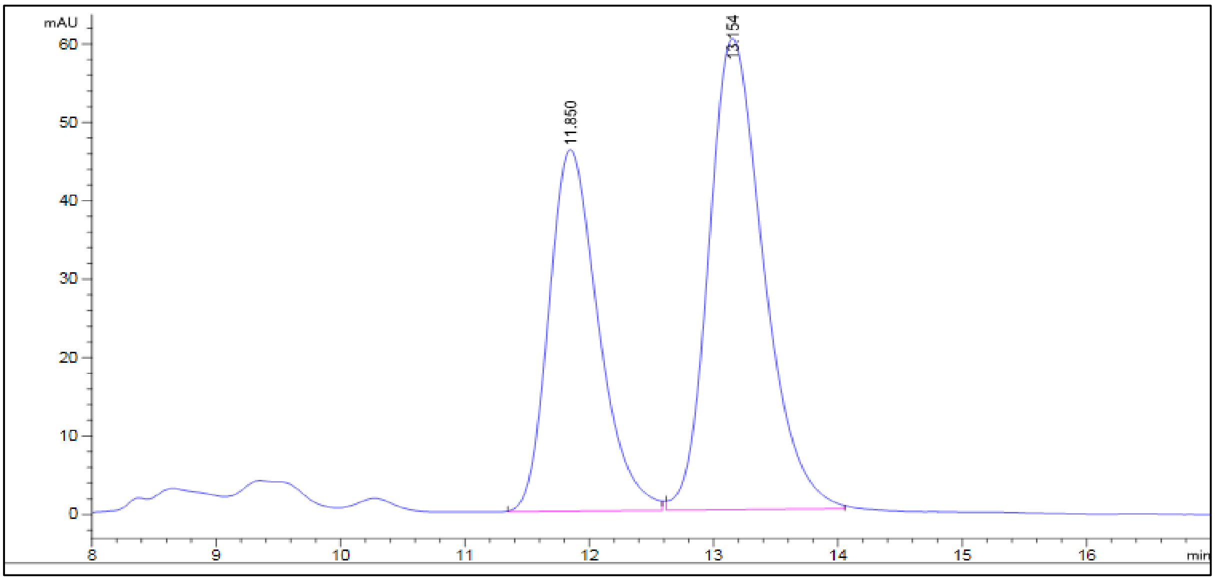

Entry #	RetTime [min]	Width [min]	Area [mAU*s]	Height [mAU]	Area %
1	11.850	0.4242	1272.67139	46.08847	40.9130
2	13.154	0.4668	1838.00745	60.09356	59.0870
